# The signaling helix: a common functional theme in diverse signaling proteins

**DOI:** 10.1186/1745-6150-1-25

**Published:** 2006-09-05

**Authors:** Vivek Anantharaman, S Balaji, L Aravind

**Affiliations:** 1 National Center for Biotechnology Information, National Library of Medicine, National Institutes of Health, Bethesda, MD 20894, USA

## Abstract

**Background:**

The mechanism by which the signals are transmitted between receptor and effector domains in multi-domain signaling proteins is poorly understood.

**Results:**

Using sensitive sequence analysis methods we identify a conserved helical segment of around 40 residues in a wide range of signaling proteins, including numerous sensor histidine kinases such as Sln1p, and receptor guanylyl cyclases such as the atrial natriuretic peptide receptor and nitric oxide receptors. We term this helical segment the signaling (S)-helix and present evidence that it forms a novel parallel coiled-coil element, distinct from previously known helical segments in signaling proteins, such as the Dimerization-Histidine phosphotransfer module of histidine kinases, the intra-cellular domains of the chemotaxis receptors, inter-GAF domain helical linkers and the α-helical HAMP module. Analysis of domain architectures allowed us to reconstruct the domain-neighborhood graph for the S-helix, which showed that the S-helix almost always occurs between two signaling domains. Several striking patterns in the domain neighborhood of the S-helix also became evident from the graph. It most often separates diverse N-terminal sensory domains from various C-terminal catalytic signaling domains such as histidine kinases, cNMP cyclase, PP2C phosphatases, NtrC-like AAA+ ATPases and diguanylate cyclases. It might also occur between two sensory domains such as PAS domains and occasionally between a DNA-binding HTH domain and a sensory domain. The sequence conservation pattern of the S-helix revealed the presence of a unique constellation of polar residues in the dimer-interface positions within the central heptad of the coiled-coil formed by the S-helix.

**Conclusion:**

Combining these observations with previously reported mutagenesis studies on different S-helix-containing proteins we suggest that it functions as a switch that prevents constitutive activation of linked downstream signaling domains. However, upon occurrence of specific conformational changes due to binding of ligand or other sensory inputs in a linked upstream domain it transmits the signal to the downstream domain. Thus, the S-helix represents one of the most prevalent functional themes involved in the flow of signals between modules in diverse prokaryote-type multi-domain signaling proteins.

**Reviewers:**

This article was reviewed by Frank Eisenhaber, Arcady Mushegian and Sandor Pongor.

## Open peer review

Reviewed by Frank Eisenhaber, Arcady Mushegian and Sandor Pongor. For the full reviews, please go to the Reviewers' comments section.

## Background

Comparative genomics has revealed several similarities as well as differences between signaling systems of prokaryotes and eukaryotes [1,2]. At the core of these signaling systems there are different catalytic domains, each having its own functional role and distinctive phyletic pattern [1]. Histidine kinases (H-kinases) and receiver domains (Rec), which form the two-component signaling systems [3], are dominant in most prokaryotes, but are relatively few or absent in most eukaryotes. In contrast, kinases phosphorylating serine, threonine or tyrosine are dominant in eukaryotes (S/T/Y-kinase) [1]. Eukaryotes and several bacteria share adenylyl and guanylyl cyclases (NMP cyclases) and cNMP signaling pathways initiated by them [4,5]. Signaling via chemotactic (Methyl-Accepting/MA) receptors is seen only in the prokaryotes; whereas diguanylate signaling, mediated by the diguanylate cyclase and two types of cyclic diguanylate phosphodiesterases (EAL and HD-GYP domains), is exclusively seen in bacteria [6]. Both prokaryotic and eukaryotic signaling systems respond to external stimuli by utilizing a variety of extracellular sensor domains, such as the CACHE, CHASE, periplasmic binding protein I/II (PBP-I/II), the helical MCP-N and 7-transmembrane receptor (7TM) domains [7-13]. Most extracellular globular domains from the above group are linked via a membrane-spanning helix to intracellular domains that transmit signals downstream. Studies in the past decade have also shown that signaling systems contain specialized intracellular domains that are typically involved in binding small molecules, like PAS and GAF domains. Such small molecule-binding domains are typically more abundant in prokaryotic signaling systems and appear to be major sensory components of signaling systems that respond to redox potential and light sensed via flavin-derivative ligands, cyclic nucleotides generated by NMP cyclases and a variety of other small molecules [14].

The distinctness of prokaryote-type signaling systems (i.e. signaling systems dominant in prokaryotes) is underscored by the deployment of a unique bi-helical module, the HAMP domain, which usually occurs immediately C-terminal to a TM segment of prokaryotic signaling receptors. It is central to transmission of sensory inputs from the extracellular sensor domains to the downstream intracellular domains [15-17]. The conventional eukaryote-type signaling proteins, like the STY-kinases or eukaryotic 7 TM receptors, do not have an equivalent of the HAMP domain. The only HAMP-domain containing signaling proteins from eukaryotes appear to be relatively late lateral transfers from bacteria and operate in manner very similar to their prokaryotic counterparts [16]. These observations indicated that many prokaryotic membrane-associated signaling molecules, irrespective of their intracellular signaling domains, depend on a common mechanism of transmission of conformational change for signal transduction. Prokaryotic signaling proteins are also highly enriched in coiled-coil (CC) segments, which are believed to be critical for dimerization. A well-known example is the intracellular signaling domain of the chemotaxis receptor that largely consists of long CC stretches in both parallel and anti-parallel configurations [18]. Computational surveys for CC have also reported the presence of such structures upstream of several histidine kinases [19]. While these observations suggested a major role for CCs in prokaryotic signal transduction, their structural diversity, their precise functional roles, phyletic spread and their interactions with other globular domains in signaling proteins are not fully appreciated.

The CC is a simple yet versatile structure, which has been widely used as a protein-protein interaction interface throughout the evolutionary history of life [20]. Well-studied examples of CCs include the basic-leucine zippers (bZIP) [21], which bind DNA via a 'scissors-grip' formed by two parallel dimerizing helices and the SNAREs, which play a major role in endoplasmic vesicle formation and fusion in the eukaryotes [22]. The CC is a long helix consisting multiple copies of a heptad (7-amino-acid) repeating unit, originally recognized by McLachlan and Stewart, with each heptad containing a similar configuration of residues [23]. As a result of bulky hydrophobic side-chains from each heptad, as well as some polar interactions, two such helices intertwine each other, to form an obligate dimeric higher-order double helical coil [20]. The resulting CCs may either be parallel or anti-parallel depending on the orientation of the dimerization partners. While generic CC stretches are encountered in a very wide range of proteins, there are certain well-conserved versions of the CC with characteristic sequence features, which mediate distinct types of interactions [20]. One such, identified in the context of histidine kinase signaling, is the dimerization and histidine phosphotransfer (DHp) module that contains a conserved histidine, which is autophosphorylated by the histidine kinase catalytic domain usually occurring immediately downstream of it [24-26]. While the DHp overlaps with regions identified in a previous study on CCs in H-kinases [19], this study did not attempt to delineate this DHp CC segment from any other CC regions that might occur in H-kinases.

Survey of previous experimental studies and our own anecdotal observations gathered in course of systematic analysis of signaling proteins indicated that there might be other conserved CC modules, distinct from other classes of CC segments including the DHp, in prokaryotic signaling proteins and their eukaryotic relatives [27-30] which might play an important role in signal transduction. We were interested in determining if any of these CC segments might define novel conserved classes of modules with a specific role in signal transduction. Accordingly, we carried out a systematic sequence analysis of signaling proteins, and as a result identified a novel conserved class of CC modules with a potentially critical role in signaling. In this article we present the evidence that this CC module might define a common paradigm in signal transmission across diverse signaling proteins.

## Results and discussion

### Identification of the signaling helix motif

In course of our systematic surveys of signaling proteins we observed a conserved sequence motif present upstream of several histidine kinases, including several previously characterized sensors such as Sln1p [27], BarA [31], TorS [32], GacS [34], LetS [35] and NarQ [33] from various bacteria and yeast. Interestingly, we also detected a similar sequence motif, independently of histidine kinases, upstream of the catalytic domains of two distinct groups of animal guanylyl cyclases, namely intracellular nitric receptors and membrane-associated receptor guanylyl cyclases, such as the vertebrate atrial natriuretic peptide receptor [28,30]. Using PSI-BLAST searches, initiated with different representatives of this motif, we also detected related sequences in close proximity of other signaling domains such as the PAS, GAF, HD-GYP and GGDEF domains with significant e-values (expect (e) value < 0.01). For example, a search initiated with the region corresponding to this motif from the *Pseudomonas aeruginosa *two-component signaling protein (PA3271; gi: 15598467, region 751–794) recovered at least 1000 significant hits within 10 iterations, wherein it was found combined to the above mentioned globular signaling domains. Aided by the boundaries of associated globular signaling domains, which were precisely established using several recently available structures or structure predictions, we delineated the region associated with the conserved motif as potentially spanning a stretch of 40–45 residues. Preliminary structural predictions for this region using the JPRED and COILS program [36,37] strongly suggested that it is predominantly α-helical. Precise alignments for the HAMP domain [16,17] and several recently available structures of the H-kinase catalytic domain and the associated DHp module [24-26] showed that this region was often closely associated, but distinct from both the CC segment of the DHp module and also the helices of the HAMP domain.

In order to investigate the distribution of this motif and define it more precisely we adopted an iterative search procedure with the HMMER package [38]. We prepared an alignment of all representatives of this motif that were unambiguously and consistently recovered with significant e-values in the above PSI-BLAST searches and constructed a hidden Markov model (HMM) from it. This HMM was then used to query a database of 255 completely sequenced genomes with the HMMSEARCH program [38] to identify potential occurrences of this motif. All hits with e-value < .001 were selected and analyzed further for domain composition. Almost all of these hits were proteins with a previously known signaling domain, including H-kinase, NMP cyclase, GGDEF, EAL, PP2C protein phosphatase, PAS, GAF or NtrC-like AAA+ domains. These observations suggested that the motif was present in the specific functional context of signaling and was likely to represent a specialized feature of these proteins rather than a generic CC. We included the cognate regions from all these newly detected proteins in the original alignment of the motif and iterated the HMM searches till no major new set of proteins was recovered.

**Figure 1:**
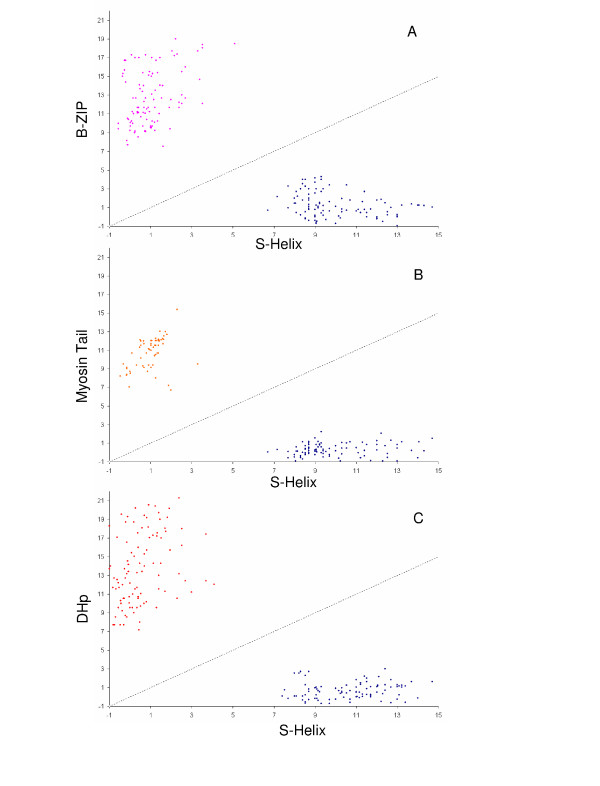
**"Cross-hit" plots for the S-helix vis-à-vis examples of various parallel and anti-parallel CCs**. The axes indicate the negative log of E-values from RPS-BLAST searches as a measure of significance. Typical S-helix and b-ZIP, Myosin tail domain or DHp domains are evident as two separated clusters, with no sequences having significant scores with both profiles. The blue dots in all the three graphs are plots of negative log of e-values from RPS-BLAST of S-helix database with S-Helix profile (x1) and of negative log of e-values from RPS-BLAST of S-helix database with bZIP, Myosin Tail domain or DHp profiles (y1). A) The pink dots are plots of negative log of e-values from RPS-BLAST of bZIP database with S-Helix profile (x2) and of negative log of e-values from RPS-BLAST of bZIP database with bZIP profiles (y2). B) The orange dots are plots of negative log of e-values from RPS-BLAST of Myosin Tail domain database with S-Helix profile (x2) and of negative log of e-values from RPS-BLAST of Myosin Tail domain database with Myosin Tail domain profiles (y2). C) The red dots are plots of negative log of e-values from RPS-BLAST of DHp domain database with S-Helix profile (x2) and of negative log of e-values from RPS-BLAST of DHp domain database with DHp domain profiles (y2).

This expanded alignment confirmed the structure prediction for this motif and suggested that it consisted of a single long α-helix, which would form a CC (See additional file 1 for complete alignment). α-helical modules are prone to attracting other such helical regions in sequence profile searches, despite sharing no specific relationship with them. However, we noted that our searches (for example, the search reported above with the *Pseudomonas aeruginosa *protein as the seed) did not draw in any commonly encountered attractors such as myosin tails, CCs of cytoskeletal filament proteins or Rad50/SMC-like ATPases with significant e-values. To further test the distinctness of this motif, we generated "cross-hit" plots using position specific score matrices (PSSMs) for this motif and several other CC regions. To generate these plots, proteins containing a given module are queried against PSSMs for the same module as well as a PSSM for another test module. Then e-values for self-hits (protein with a given module against their own PSSM) and cross-hits (protein with a given module against the PSSM of test module) for both this motif and test modules were plotted as X-Y scatters. In these plots, we noted a strong segregation of this motif from several other tested CC regions like the DHp module, myosin and bZIPs, supporting the distinctness of the motif from both generic CCs and well-conserved CC regions (Fig. 1). Given its almost exclusive co-occurrence with some major signaling domain we named this motif the Signaling helix (S-helix).

### Sequence conservation pattern of the signaling helix

**Figure 2:**
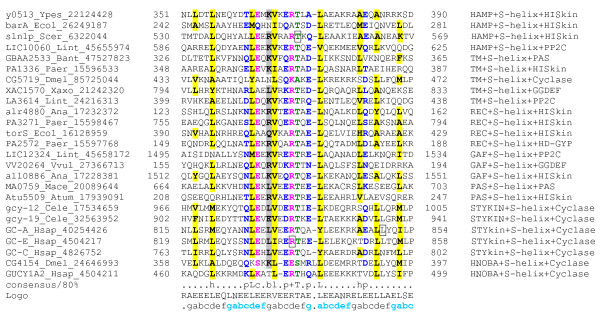
**Multiple alignment of representative examples of the S-helix**. Representatives from a multiple alignment of the S-Helix domain, generated using the MUSCLE program [49] and corrected using PSI-BLAST [47] search results, are shown. The logo and the heptad notations are shown. The 80% consensus shown below the alignment was derived from an alignment of all the members using the following amino acid classes: consensus from the logo is also shown and colored using the following amino acid classes: hydrophobic (h: ACFILMVWY, yellow shading); aliphatic subset of the hydrophobic class (l; ILV, yellow shading); the aromatic subset of the hydrophobic class (a; FHWY, yellow shading); small (s: ACDGNPSTV, green); the tiny subclass of small (u; GAS, Green shading); polar (p: CDEHKNQRST, blue); the charged subclass of polar (c: DEHKR, pink); the positive subclass of charged (+: HKR, pink); the negative subclass of charged (-: DE, pink); alcohol (o: ST, Blue); and big (b: KFILMQRWYE, grey). A 'L', or 'T' show the completely conserved amino acid in that group. The limits of the domains are indicated by the residue positions, on each side. The domain architecture is shown to the right. The domain abbreviations are as in section 2 Materials and Methods and legend to Fig. 4. The mutations discussed in the paper are marked with boxes. The sequences are denoted by their gene name followed by the species abbreviation and GeneBank Identifier. The species abbreviations are: Ana: *Nostoc *sp.; Atum: *Agrobacterium tumefaciens*; Bant: *Bacillus anthracis*; Cele: *Caenorhabditis elegans*; Dmel: *Drosophila melanogaster*; Ecol: *Escherichia coli*; Hsap: *Homo sapiens*; Iloi: *Idiomarina loihiensis*; Lint: *Leptospira interrogans*; Mace: *Methanosarcina acetivorans*; Paer: *Pseudomonas aeruginosa*; Scer: *Saccharomyces cerevisiae*; Vvul: *Vibrio vulnificus*; Xaxo: *Xanthomonas axonopodis*; Ypes: *Yersinia pestis*

**Figure 3:**
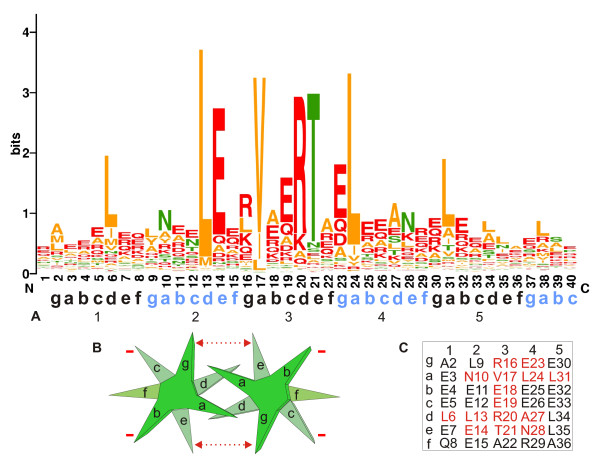
**Sequence logo and interaction models for theS-helix**. A) The sequence logo generated using the Weblogo program [50] is shown. The 'a' and 'd' positions of each of the 5 heptads, as per the notation of MacLachlan and Stewart [23], is also shown below the logo. B) the heptad interaction between two parallel helices is shown. The dotted red arrow indicates the 'g'-'e' interaction. The red negative sign ("-") indicates that most prevalent residues at the b and c positions are negatively charged. C) The most prevalent residue of each position on the S-Helix is shown as a table with the rows showing the positions in each heptad, and the columns showing the five heptads. The residues in red indicate the highly conserved positions.

A multiple alignment of 1000 distinct S-helix representatives detected in our searches from across the three superkingdoms of Life were used to construct a comprehensive multiple alignment (Fig. 2; see additional file 1 for complete alignment) and a sequence logo quantifying the conservation at each position was derived from this alignment (Fig 3) [39]. The logo revealed the telltale feature of CCs in the form of two periodic series of positions dominated by conserved hydrophobic residues, which form the principal interface for dimerization through hydrophobic interactions (Fig. 3). This enabled us to anchor the 'a' and 'd' positions of each heptad as per the notation of McLachlan and Stewart [23] (Fig. 3). About 5 conserved 'a' and 'd' positions were detected suggesting the majority of S-helices contain 5 heptad units, which is consistent with the size of 40 residues that was determined through delineation of domain boundaries. However, it should be noted that in families of CC modules, such as the b-Zip module, the length of the CC segment can be variable, and differ in the number of heptad repeats they span. Thus, some S-helix modules could be potentially shorter or longer. Furthermore, given the proximity to other helical segments such as the DHp and HAMP, the S-helix could merge with them at its termini, without a clear demarcation of their respective helical elements. Given that the S-helix occurs in the cytoplasmic side of numerous TM receptors with intracellular dimeric signaling domains, it is clear that it forms a parallel CC. This also clearly distinguishes the S-helix from certain CC segments found between GAF domains, which run in the anti-parallel configuration [40].

The S-helix is typified by a strongly conserved 'ERT' signature seen in the central heptad unit in positions corresponding to 'c', 'd' and 'e' (Fig. 3). There is a notable discrimination against most other residues, especially non-polar residues, in the positions corresponding to the R and T of this signature (Fig. 3). Beyond these, and the residues forming the hydrophobic zipper, there are several other strongly conserved features that stand out in the S-helix (Fig. 2, 3). These include a conserved glutamate 14 and arginine 16, respectively corresponding to 'e' and 'g' positions of the heptad and a glutamate 23 corresponding to a 'g' position (Fig. 2, 3). While similar residues in equivalent positions are occasionally encountered in heptads from various CC regions [21,23], the specific constellation of strongly conserved residues in the S-helix is not the defining characteristic of any other class of CCs. Studies on parallel CCs have shown that the positions 'g', 'a', 'd' and 'e' in the heptad are critical for dimer interactions [21]. We observed that these positions are about 4–5 times more conserved on an average in heptads of the S-helix than the 'b', 'c' and 'f' positions. It has also been seen that the 'g' and 'e' positions of CCs, which lie on the periphery of the hydrophobic interface play an additional role in stabilizing the dimer interactions [21]. We noted that typically oppositely charged residues, or residues that are unlikely to form any disruptive repulsive interactions are conserved at the 'g' and 'e' positions (see below). This suggests that there is strong selection to favoring specific dimerization between S-helices. The most commonly found residues at the external 'b' and 'c' positions are acidic residues suggesting that S-helices tend to have negatively charged outer surfaces (Fig 3).

### Phyletic patterns and architectural contexts of the S-helix

The S-helix is found in all major bacterial lineages, few euryarchaea, including halophiles and some thermophiles occupying the lower end of the thermophilic temperature range. In eukaryotes S-helix containing proteins are to date seen only in animals and fungi. All fungal versions are receptor kinases prototyped by Sln1p, with previously known intracellular HAMP, DHp and histidine kinase modules. In animals the S-helix is found in three different classes of guanylyl cyclases, two of which are the previously characterized forms mentioned above. The third version is seen in insects and sea urchins and combines a distinct predicted extracellular region with an intracellular segment with the S-helix and the cyclase catalytic domain. PSI-BLAST searches with the extracellular region revealed that it contains a previously unknown version of the NIT domain [41], which has been predicted to sense nitrite and nitrate in a variety of bacteria. This implies that the NIT domain-containing cNMP cyclases are likely to be receptors for extracellular nitrogen oxides in these animals, distinct from the intracellular nitric oxide receptors with sensory HNOB and HNOBA domains [30].

Bacteria show the greatest diversity of domain architectures in proteins containing the S-helix, and in several cases eukaryotic or archaeal proteins with an S-helix can be clearly demonstrated to be related to a specific bacterial form (for an example see reference [30]). These observations suggest that the module arose in bacteria and was laterally transferred to archaea and eukaryotes along with various genes encoding various signaling proteins, which were transferred to these lineages [2]. In several bacteria, like *Geobacter*, *Bradyrhizobium*, *Bdellovibrio*, *Vibrio*, *Pseudomonas*, *Leptospira*, *Synechocystis *and *Nostoc *and the archaeon *Methanosarcina *there are expansions of signaling proteins containing the S-helix. In most cases these appear to arise from lineage-specific expansions of particular specialized sensors, such as the MEDS and PocR domain proteins in *Methanosarcina*, which have been predicted to play a role in sensing growth substrates [9], and 7-TM receptors with different intracellular signaling domains in *Leptospira *with a possible role in sensing carbohydrates [9] and PAS domain containing receptor histidine kinases in *Geobacter*, with a possible role in redox potential sensing [42]. Likewise, in the nematode *C. elegans*, there are two independent expansions of the S-helix containing NMP cyclases [28,30]. These expansions suggest a widespread utility for the S-helix in receptors receiving diverse types of sensory inputs and delivering signaling outputs via diverse catalytic domains.

**Figure 4:**
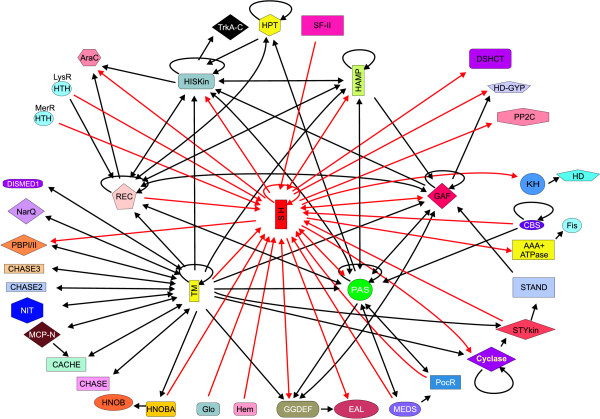
**Domain architecture graph for the S-helix**. The ordered graph for the contextual information contained in domain fusions, drawn using Pajek [71] and modified with CorelDraw, is shown. The direction of the edge denotes the order of the fusion of domain in the polypeptide. If a domain is found on either side of another domain in different architectures, the edge points in both the direction. Domains with tandem repeats have loops pointing to themselves. The loop on TM includes bacterial 7TM receptors, 9TM receptors and 12TM Na+/proline symporters (found fused to bacterial histidine kinases in proteobacteria) with multiple successive TM segments separated by short hydrophilic loops. All connections to the S-helix are shown in red, while the other connections are in black. Domain abbreviations are as shown in section 2 Materials and Method or: SH – S-helix, Cyclase-NMP cyclase, HisKin – Histidine Kinase (including the DHp module), DISMED1 (for 7TMR-DISM extracellular domains 1); STYKIN – S/T/Y Kinase; NarQ – Extracellular nitrate sensing domain domain found in NarQ family of proteins; Glo – Globin domain and Hem – Hemerythrin.

**Figure 5:**
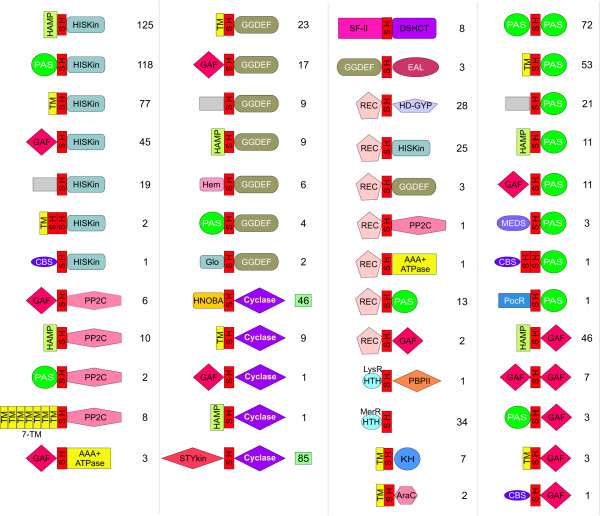
**Domain neighborhoods for the S-helix**. The architectural context in which the S-helix occurs along with its immediately adjacent domains is shown here. The lower bound of numbers of such contexts in different proteins from 255 completely sequenced domains is shown to the right. The two contexts with the numbers in the green boxes are seen in animals. The domain abbreviations are as in section 2 Materials and Methods and Figure 4. The grey boxes are uncharacterized domains.

To understand the functional significance of the S-helix we carried out a systematic analysis of the domain architecture contexts in which it is found. To do this we firstly used sensitive PSI-BLAST-derived PSSMs and HMMs for a range of domains that are known to occur in signaling proteins (refer Methods) and systematically detected all their occurrences in proteins containing the S-helix. We then collated substantial regions in these proteins that did not map to any of these known domains and scanned them for transmembrane regions, signal peptides, and compositionally biased stretches (refer Methods for details). Any regions that did not contain these compositional features were used as seeds in PSI-BLAST searches to determine if there were any divergent copies of previously known domains or uncharacterized protein domains. We randomly checked the architectures of several proteins determined through the above semi-automatic procedure using manual case by case analysis and found at least 90% recovery of correct domain architectures. This procedure allowed us to arrive at reasonably reliable domain architectures for all the S-helix proteins detected in a search of a database of 255 organisms with completely sequenced genomes. We represented this information in the form of an ordered graph (Fig. 4), where the nodes are domains and the edges represent the direction of the connection between domains in the same polypeptide (N-terminal->C-terminal or vice versa). The information is also represented as individual architecture diagrams showing all domain contexts in which the S-helix is found (Fig. 5).

The domain architecture graph of the S-helix underscores its general significance to prokaryote-type signaling systems, because most major signaling domains prevalent in prokaryotes are connected to it within two-degrees of the graph (Fig. 4). Some important syntactical features of the domain architectures involving the S-helix are also immediately apparent in the graph. The S-helix is almost always found between two signaling domains: typically it separates a range of N-terminal sensory domains, like the PAS, GAF, MEDS, PocR or CBS domains, or extra-cellular sensor domains connected via transmembrane helices, or conformational-change-transducing domains like the HAMP, from C-terminal catalytic domains such as histidine kinase, cyclic diguanylate phosphodiesterases like HD-GYP and EAL, PP2C protein phosphatase, NMP and diguanylate cyclases and NtrC-like AAA+ ATPase. The next most prevalent architectural type is one where the S-helix separates two small-molecule-binding domains of same or different types (Fig. 5). For example, it might occur between two PAS domains or between a GAF and a PAS domain. At low frequency, the S-helix also connects signaling domains to DNA-binding Helix-Turn-Helix (HTH) domains. Just as the histidine kinase module only occurs downstream of the S-helix, the receiver domain of the two-component system is only seen upstream of the S-helix (Fig. 5). More generally the S-helix always connects an upstream receiver domain to a downstream catalytic domain like histidine kinase, HD-GYP phosphodiesterase or diguanylate cyclase, or a ligand-binding domain such as PAS or GAF (Fig. 5).

The S-helix occurs most commonly upstream of a histidine kinase but is never observed upstream of a eukaryote-type S/T kinase. About 10% of the histidine kinases from completely sequenced genomes show an S-helix upstream of the DHp and catalytic domains. Only in animal receptor guanylyl cyclases such as Atrial natriuretic peptide receptors and Sea-urchin sperm peptide receptors the S-helix occurs downstream of the S/T kinase domain (Fig. 5); however, this version of the kinase domain appears to be inactive and probably only serves as an allosteric nucleotide binding domain. Another architecture that is typically avoided by the S-helix is a fusion to the methyl-accepting chemotaxis receptor domain. These domains are themselves entirely composed of CC stretches that dimerize in specific fashion, probably rendering a structure like the S-helix superfluous [18].

These observations imply that the S-helix is relevant across numerous prokaryote-type signaling contexts, both membrane-associated and soluble, irrespective of the two signaling domains it links. Often these domains may sense and deliver very different kinds of signals. However, it appears to be entirely incompatible with most *bona fide *'eukaryote-type' signaling systems [1], especially those involving S/T kinases, GTPases or eukaryotic 7 TM receptors. This suggests that the function of S-helix is likely to be a general one that fundamentally distinguishes many of the 'prokaryote-type' signaling systems from the 'eukaryote type' signaling proteins, just as with the HAMP domain. Furthermore, patterns in S-helix domain architectures strongly indicate a certain positional polarity in the function of the S-helix, potentially indicating a role in transmitting signals from one domain to another.

### Structural analysis, mutational data and function of the S-helix

To obtain a clearer picture of the actual role of the S-helix in signaling we combined the inferences drawn from sequence conservation and domain architectures with previously available experimental data on proteins containing the S-helix. The best experimental leads for the possible function of the S-helix is available from mutational data on the yeast Sln1p kinase and human receptor guanylyl cyclases [27-29]. In the case of yeast Sln1p, deletion of the region mapping to the S-helix results in total loss of kinase activity [29]. Its replacement by an unrelated parallel CC from the bZIP proteins results in a partial rescue of the phenotype, with the kinase monomers interacting comparable to the wild type enzyme. However, the hybrid Sln1p apparently has a defect in turning off the kinase [29]. A similar defect in turning off the kinase activity was also observed in a Sln1p mutation substituting the conserved T of the ERT signature with an isoleucine [27,29]. Deletion of the region corresponding to the S-helix in human receptor guanylyl cyclases results in loss of activity and the polypeptides migrating as monomers, suggesting that the S-helix is critical for dimerization [43]. Another mutation targeting the S-helix has been observed in the terminal heptad of the receptor guanylyl cyclase GC-A [28], wherein a leucine is substituted by an arginine. This position shows a clear preference for a hydrophobic residue and strongly discriminates against a positively charged residue. Thus, it is possible that the substitution observed in GC-A disrupts the assembly of a functional dimer, consistent with the observed loss of catalytic activity. Substitution of the conserved R of the ERT signature by C in the human receptor cyclase GC-E results in the retinal disorder, dominant rod-cone dystrophy [44]. Most interestingly, even in this case the defect arises from failure to turn-off cGMP production. These results, together with sequence conservation and domain architectural patterns, suggest that the S-helix is not merely a generic CC required solely for dimerization, but probably plays a specific role in preventing constitutive activation of downstream signaling domains in the absence of a stimulus from upstream domains. Nevertheless, given the vast diversity of proteins in which it is present, and a degree of sequence divergence, it is possible that versions of the S-helix different proteins might have acquired distinct function from those suggested by the available mutational data on the proteins discussed above.

**Figure 6:**
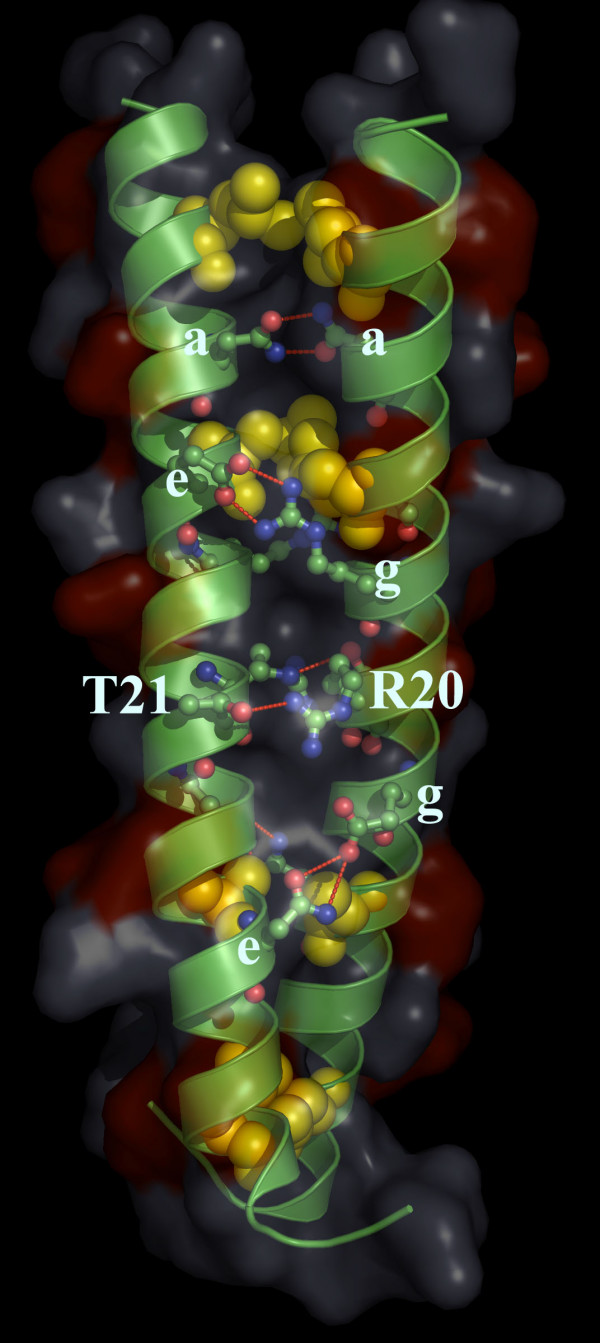
**Approximate structural model for the S-helix**. A model of the S-Helix domain was constructed using other parallel CCs as templates (e.g. PDB: 1YSA). The sequence that was model was derived from the the logo shown in Fig. 2 and represents an idealized S-helix. The hydrophobic residues at position 'd' are shown as yellow spheres. The surface view is shown and the negatively charged ridges on the surface formed by 'b' and 'c' positions are shown in red. The hydrogen bonds, red dots, show the 'a'-'a' interaction of N10-N10; the R20-T21 interaction; the 'g'-'e' interactions of R16-E14 (Top) and E23-N28 (bottom).

To better explore this functional proposal we constructed a model of the S-helix using other parallel CCs as a template (e.g. pdb 1ysa [45]). While it should be stressed that such models are only an approximate guide and no substitute for an actual structure, they do provide a means to appreciate certain key features (Fig. 6). Firstly, the model shows the expected hydrophobic interactions at the interface mediated by the 'a' and 'd' positions of a heptad. Likewise the model also supports the idea that residues in 'g' and 'e' form stabilizing interactions via oppositely charged residues or through hydrogen-bonding between polar residues. However, there are some notable variations on the general CC theme. The 10th residue, corresponding to an 'a' position, is most often an asparagine rather than a hydrophobic residue. This N is predicted to form stabilizing hydrogen-bonding interactions with its cognate from the adjacent monomer, and is similar to asparagines located in the 'a' or 'd' positions of bZIP proteins [21]. More importantly, the arginine of the conserved ERT signature lies in a 'd' position that is typically hydrophobic. Given its size it is likely that the charged head of the R projects to the exterior, where it could potentially form a polar interaction with the T at the flanking 'e' position (Fig. 6). Such interactions are likely to be critical for the function of S-helix as suggested by mutational data, and consistent with such a proposal, mutation of the T to other polar residues, like an acidic residue, does not disrupt function, unlike a hydrophobic substitution [29]. These observations suggest that the conserved RT signature of the linker forms a distinctive structural feature that functions as a switch within the CC. It is likely that the arginine owing to the length of its side chain can form alternative interactions that respectively prevent or allow downstream domains from "firing". Given that the RT signature lies in the key 'd' position of the central heptad of the S-helix its interactions are likely to affect the conformation of the entire CC. This proposal is consistent with the observed polarity in the domain architecture graph, where catalytic domains are typically downstream of the S-helix, with various sensory domains or the receiver domain that gets phosphorylated on a conserved aspartate being upstream. Thus, due to its central position, the switch in the CC could respond to a conformational alternation in the upstream domain (the stimulus), only then undergo an appropriate conformational alteration itself, and thereby transmit a signal to allow action of the downstream domains.

The model also suggests that the acidic residues preferred in the external 'b' and 'c' positions are likely to form external ridges of negative charge along the surface of the S-helix (Fig. 6). These negatively charged ridges could possibly repel other such S-helix dimers and might regulate the spacing or modulate higher-order multimer formation by signaling protein dimers.

## Conclusion

Identification of the S-helix helps in delineating the distribution and specific roles of different CCs in prokaryote-type signaling proteins. An earlier computational study detected CC regions in upstream of numerous histidine kinases [19], but at that point in time their diversity and functions were not completely understood. Subsequent structural studies revealed that the principal CC associated with most histidine kinases is the DHp module that contains the autophosphorylated histidine [24-26]. This module might encompass CCs of widely different lengths, as suggested by the recent structures, but are unified by the formation of a C-terminal 4-helical bundle to which the kinase catalytic domain is connected. Likewise the structure of the intracellular signaling domain of the chemotaxis receptors [18], showed that it forms a distinctive long CC-structure. Structural studies on tandem pairs of GAF domains revealed the presence of CC regions between them [40,46]. The prokaryotic versions of such inter-GAF CCs are anti-parallel in configuration, whereas, the eukaryotic versions from cNMP phosphodiesterases are believed to form parallel dimers. In sequence searches these show poor sequence conservation, and apparently display no unusual pattern of residues as seen in the S-helix. These might represent the extended dimerization interface unique to certain GAF- and PAS-containing proteins. Our current study shows that a subset of histidine kinases and a numerous other prokaryotic signaling proteins contain a distinctive CC motif displaying a specific sequence conservation pattern, which is different from the other characterized CC regions of signaling proteins.

The S-helix from different proteins is typically embedded between two flanking globular domains, of which one or both domains found in other proteins similarly associated with an S-helix. Hence, it is likely that the corresponding S-helices are duplicated and evolutionarily mobile along with their flanking homologous globular domain/s. This is a more parsimonious explanation for the proliferation of the S-helix than extensive multiple sequence convergences of connector segments between globular signaling domains. Due to the frequent presence upstream of histidine kinases, it is possible that the S-helix originally arose as an N-terminal extension of the same coiled-coil segment that contains the DHp module, and acquired a distinct regulatory function. The advantage conferred by this regulatory function resulted in natural selection operating to preserve it as a distinct module. Due to the rampant domain swapping of catalytic and sensor domains of signaling proteins that occurred in course of their evolution [14], the S-helix spread across a range of proteins, where it provided a similar regulatory role different signaling contexts. Regions mapping to the S-helix have previously been extensively experimentally investigated in yeast Sln1p and human receptor guanylyl cyclases. Utilizing information from domain architectures and sequence analysis, we were able to generalize these results to propose a unified role for the S-helix in prokaryotic signaling. It appears that a number of α-helical modules, namely HAMP, S-Helix and DHp are used in prokaryotic-type signaling transduction, presumably as peptide analogs of a system of mechanical levers to appropriately convey the sensory input from a receptor domain to a signal transmitting domain (Fig. 5). These features differentiate prokaryote-type signaling systems from eukaryote-type systems, and might also explain the general tendency and ability of prokaryote-type signaling proteins to function as dimers.

In summary, we present evidence for a special structural feature shared by numerous prokaryote-type signaling proteins, which might function as a switch in the communication between two globular domains that prevents constitutive activation of signal transduction in the absence of an appropriate stimulus from an adjacent domain.

## Methods

The non-redundant (NR) database of protein sequences (National Center for Biotechnology Information, NIH, Bethesda) was searched using the BLASTP and PSI-BLAST programs [47]. Profile searches using the PSI-BLAST program were conducted either with a single sequence or an alignment used as the query, with a profile inclusion expectation (E) value threshold of 0.01, and were iterated until convergence [48]. Multiple alignments were constructed using the Muscle program [49], followed by manual correction based on the PSI-BLAST results. The JPRED program [36] and the COILS program [37] were used to predict secondary structure. The logo was generated using the WebLogo program [50]. All completely sequenced and assembled microbial genomes that were submitted to the NCBI GenBank database as of August 2005 were used in this analysis (see Additional file 1 for the list). A complete list of these genomes and the predicted proteomes in fasta format can be downloaded from the NCBI Genomes division of GenBank

The multiple alignment was used create a HMM using the Hmmbuild program of the HMMER package [38]. It was then optimized with Hmmcaliberate and the resulting profile was used to search a database of completely sequenced genomes using the Hmmsearch program of the HMMER package. Proteins from the search with an e-value > .001 were collected to get the S-helix database. Signal peptides were predicted using the SIGNALP program [51,52]. Transmembrane regions were predicted in individual proteins using the TMPRED, TMHMM2.0 and TOPRED1.0 program with default parameters [53-55].

To determine the domain architecture of the S-helix containing proteins query sequences and profiles of the following domains were used to search the proteins: NMP cyclase, CACHE (extracellular domain found in Calcium channel and Chemotaxis proteins) [7], CHASE (Cyclase/Histidine kinase-Associated Sensing Extracellular domain) [8,10], CHASE2, CHASE3 [11], cNMPBD (cNMP binding domain) [4], EAL (EAL motif containing cyclic nucleotide phosphodiesterases) [6], GAF (cGMP phosphodiesterase, Adenylate cyclase, FhlA domain) [56], GGDEF-motif-containing nucleotide cyclase domains (GGDEF) [6], HAMP (domain present in Histidine kinases, Adenylyl cyclases, Methyl-accepting proteins and Phosphatases) [16], HD-GYP (cyclic diaguanylate phosphodiesterases of the HD-GYP variety) [6], Histidine Kinase [3], HNOB (Heme NO Binding domain), HNOBA (HNOB Associated domain) [30], HPT (The histidine-containing phosphotransfer (HPT) domain), Methyl Acceptor (MA) domain [18], MCP-N [7], MEDS [57], PAS (Per-Arnt Sim domain; Ligand binding domain found in *Drosophila *Period clock proteins, vertebrate Aryl hydrocarbon receptor nuclear translocator and *Drosophila *Single minded proteins) [58,59], PBPI, PBPII (Periplasmic Binding Protein type I and II) [12,60], PocR [57], Receiver domain (REC) [3], S/T/Y Kinase, and NtrC-like AAA+ ATPase [61]. The boundaries of domains obtained from these searches were used to generate domain architectures using the in-house TASS package (VA, SB and LA unpublished). Globular regions without any hits in these proteins were isolated and tested for the presence of other domains using a combination of BLASTCLUST (protein clustering program with empirically determined length and score threshold cut off values; for documentation see ), PSI-Blast searches and Pfam searches [62]. Any new domains which were detected by this procedure were then used to update previously determined domain architectures. Iterations of these steps were used to detect Bacterial 7TMR-DISM (for 7TMreceptors with diverse intracellular signaling modules) [9], 7TMR-DISMED1 (for 7TMR-DISM extracellular domains 1) [9], TrkA-C (TrkA C terminal domain) [14], CBS (Cystathionine β-Synthase) [63,64], PP2C (Sigma factor PP2C-like phosphatases) [65], AraC, Fis, MerR and LysR varieties of HTH (Helix-Turn-Helix DNA binding domain) [66], TARH, Hemerythrin [67], Globin [68], SF-II helicase, DSHCT (C terminal domain is found in DOB1/SK12/helY-like DEAD box helicases) [69], STAND ATPase (Signal Transduction ATPases with Numerous Domains) [70], KH (K-Homology RNA binding domain), and NIT (A nitrate- and nitrite-sensing domain found animal receptor cyclases) domains [41].

Domain linkages and domain context were obtained from the domain architectures using the TASS package. The domain linkage data was converted into a network graph of domain architectures using the Pajek software [71] and then manually modified using CorelDraw. To generate the "cross-plot" graph PSSMs were generated with S-helix, B-Zip, Myosin Tail domain and the DHp domain using PSI-BLAST search against nr. Each profile was first converted to matrices using makemat (with S set to 1) and copymat (with r set to true) programs of the IMPALA package and then the database was formatted for RPS-BLAST [72]. Each profile was also used to generate a query database of 100 random proteins collected from the third iteration of a PSI-BLAST search against nr. RPS-BLAST searches were conducted using each profile first with the S-Helix query database then with the target domain query database and the values were used in a X-Y scatter plot. For example, RPS-BLAST search was conducted with the S-helix profile first on the S-helix database and then the bZIP database, generating values x1 and y1. Then RPS-BLAST search was conducted with the bZIP profile first on the S-helix database and then the bZIP database, generating values x2 and y2. Any significant hits to both the profiles would show up near the diagonal. Absence of such overlap suggests the clear demarcation between the domains. The Swiss-PDB viewer [73] and Pymol programs [74] were used to carry out manipulations of PDB files. The model was generated using SWISS-MODEL [75]. Briefly, this process consisted of constructing a consensus sequence from the sequence Logo for the S-helix monomer. The two protomers of the S-helix were individuyally threaded on to the respective b-ZIP protomers as templates as recommended for the SwissModel oligomer modeling procedure. The layers were then merged and the residues making clashes were fixed, and submitted for oligomeric modelling by the SwissModel server. Energy minimization of the modeled protomers was carried out using the GROMOS 43B1 force field incorporated in SwissModel. Figures were rendered using PyMOL [74].

## Abbreviations

S-helix – Signaling Helix; CC – Coiled Coil; H-Kinases – Histidine Kinases; DHp-dimerization and histidine phosphotransfer; bZIP-basic-leucine zipper; PSSMs-position specific score matrices; HTH – Helix-Turn-Helix; HNOB – Heme NO Binding domain; HNOBA – HNOB Associated domain; HPT – histidine-containing phosphotransfer domain, MA-Methyl Acceptor domain; PAS-Per-Arnt Sim domain; MCPN-Methyl Acceptor Chemotaxis protein N-terminal domain; KH-K-Homology RNA binding domain

## Authors' contributions

VA contributed to the discovery process, programming and preparation of the manuscript and figures. SB contributed to writing programs for this study. LA conceived the study, contributed to the discovery process and preparation of the manuscript. All the authors read and approved the final manuscript.

## Reviewers' comments

### Reviewer's report 1

#### Arcady Mushiegan, Stowers Institute, Kansas City, USA

This is a report of discovery of a novel conserved protein module, widely distributed in many classes of signal transduction proteins. Despite relatively small size (40–45 amino acids) and simple secondary structure (paired alpha-helices), the S-helix domain appears to be amenable to sensitive and specific detection using probabilistic sequence comparisons and database searches.

In Results and discussion/*Phyletic patterns and architectural contexts of the S-helix/para 4 *and elsewhere: what is 'syntactical' – is there such a thing as agreed-upon definition of grammar of protein domains? Maybe 'positional' or 'domain order' would be less pretentious. Also, obviously, biological function occurs in 3-D, where perhaps it does not matter much whether the domain fusion is N-terminal or C-terminal. The order of fused domains might be a useful synapomorphy, however, but this section does not seem to be focused on that.

##### Author response

*Currently we are using the term syntax only in one place and specifically as a descriptor for the general rules in domain architecture, which are seen in the S-helix proteins. While there are no universal grammatical principles for protein domains, we do observe that several domains show strong rules in terms of the positions in the primary structure and we are merely using the term syntax for that. The S-helix is clearly one such domain – for example it occurs rather strictly N-terminal to the histidine kinase and nucleotide cyclase domains (like a "preposition"). While we do agree that biological function occurs in 3D, there is a strong polarity in terms of 3D domain arrangement of many of the signaling proteins. For example, there are specific regions located outside the cell linked to intracellular parts by means of a relatively rigid TM helix. Likewise, the S-helix and DHp are relatively rigid helical segments that impart the protein an "extended" configuration wherein the location at the N- or C-terminus of a structurally rigid segment would matter*.

### Reviewer's report 2

#### Frank Eisenhaber, Institute of Molecular Pathology, Vienna, Austria

The homology concept is traditionally applied to protein sequence segments having no bias towards few amino acid types or short, simple, repetitive patterns and representing complete globular domains. The requirement of matching hydrophobic patterns and the larger number of alignment positions ensured significance of annotation transfer within such segment families. In this context, the existence of a common ancestor can be reliably postulated even for some cases of sequence pairs that have extremely diverged to essentially zero sequence identity.

These insurances are no longer valid if shorter sequence motifs or repetitive patterns such as in coiled coils are the subject for sequence similarity searches. Both concerns are valid in this study. I find it interesting what kind of additional arguments have been brought up by the authors to support the significance of the relatedness of sequence segments within the S-helix family (distinctness from other CCs, sequence architecture with signaling domains, biological context of well-studied examples). The comparison with major families of CCs is instructive, although this argument is not exhaustive as well as the architecture consideration. I agree with the authors that the sequence similarity within the family might indicate similarity of function. I am not so sure whether it also indicates evolutionary relatedness in all instances since it is not improbable that short functional motifs originate de novo from diverse ancestors (in this case, quite frequently occurring alpha-helical precursors).

##### Author response

*We admit that in the case of sequences with lower entropy than typical globular domains the possibility of convergent sequence similarity exists. However, the sequence profile searches seeded with different starting sequences (e.g. in "Identification of the Signaling Helix motif" section) show that despite being a coiled-coil the S-helix does not tend to promiscuously recover diverse functionally unrelated CC segments. It is also seen that the S-helix is typically embedded in the context of two flanking domains, of which one or both domains are homologous across different proteins with S-helices. The globular domains, such as the histidine kinase, PAS, GAF and the like, with the S-helix is closely associated in these architectures are clearly the product of divergent evolution following duplication. Hence, the explanation that the S-helices of the proteins are homologous and evolutionarily mobile, along with their flanking homologous domain/s is clearly a more parsimonious explanation than extensive multiple convergences of a connector or flanking region associated with such domains*.

I find this work of great interest and of importance for understanding protein evolution also beyond the specific functional module S-helix that is considered here.

It would be convenient for the reader if the authors could make available some additional information via an FTP site, in the text or on request. The exact starting sequence segments of the searches (beginning of Results section) would be helpful for people who wish to reproduce the data. The alignments and RPS-BLAST libraries of signaling domains listed in the Methods section would be of interest to other researchers.

##### Author response

*We have added a few new sentences to the section "Identification of the Signaling Helix motif" providing an example of the starting point for the searches. We are also providing in *additional file 1*the entire list of S-helix proteins detected in large-scale searches across various complete genomes along with their alignment from which the RPS-BLAST profiles and HMMs were prepared*.

### Reviewer's report 3

#### Sandor Pongor, International Centre for Genetic Engineering and Biotechnology, Trieste, Italy

This is an interesting piece of work and a well-written paper that I suggest to be published in its present form.

## Supplementary Material

Additional File 1 Comprehensive alignment and architectures of S-Helix. Click here for file
