# Supplementary material for: The signaling helix: a common functional theme in diverse signaling proteins
Source: Biol Direct. 2006 Sep 5;1:25. doi: 10.1186/1745-6150-1-25 (PMC1592074; doi:10.1186/1745-6150-1-25)
Supplement: Additional File 1 — Comprehensive alignment and architectures of S-Helix. [file 1745-6150-1-25-S1.html]

The signaling helix: a common functional theme in diverse signaling proteins


```
The signaling helix: a common functional theme in diverse signaling proteins


Vivek Anantharaman, S. Balaji and L. Aravind*


National Center for Biotechnology Information, National Library of Medicine,
National Institutes of Health, Bethesda, MD 20894, USA


 

*For correspondence:
Email: aravind@ncbi.nlm.nih.gov
Telephone: (301) 594-2445
Fax: (301) 480-9241


This file contains a comprehensive alignment of the S-helix segments detected in 134 complete genomes. 
This list of genomes with S-helix proteins is provided at the end of the file. The column at the extreme 
left provide the Genbank gi numbers of the proteins for their retrieval and extreme right columns 
contain the **automatically** generated domain architectures, protein size, organism name and 
Genbank definition lines.


# 70                                                                            # 70
26247013 4.9e-05: _1_[66..105]     SSVHALNRHREQLAAQ---VKARTA---ELQELVIEHRQARAEAEK        26247013       HAMP+REC+SHELIX+HISKIN+REC+HPT                                                                               576    Escherichia coli CFT073                                              Sensor protein torS [Escherichia coli CFT073]                                    
27365916 0.00013: _1_[178..217]    ERAVIDIENKEKLQRI---VELRTR---ELALAREESERANRAKSE        27365916       SHELIX+HISKIN+REC                                                                                            574    Vibrio vulnificus CMCP6                                              Signal transduction histidine kinase [Vibrio vulnificus CMCP6]                   
37679852 0.00022: _1_[173..212]    ERAVIDIENKEKLQRI---VELRTR---ELALAREESERANRAKSE        37679852       SHELIX+HISKIN+REC                                                                                            569    Vibrio vulnificus YJ016                                              signal transduction histidine kinase [Vibrio vulnificus YJ016]                   
15641456 1.7e-09: _1_[175..214]    ERAISDIEHKEELQKL---VEIRTR---ELRLAQKLAEQANQSKSN        15641456       SHELIX+HISKIN+REC                                                                                            572    Vibrio cholerae O1 biovar eltor str. N16961                          sensor histidine kinase/response regulator [Vibrio cholerae O1 biovar eltor str. N16961]
27375440 0.00016: _1_[40..79]      LNAFSLFETAIALDHK---VRDRTH---QLREALHSVERANEGLYR        27375440       SHELIX+HISKIN+REC                                                                                            452    Bradyrhizobium japonicum USDA 110                                    two-component hybrid sensor and regulator [Bradyrhizobium japonicum USDA 110]    
13471220   3e-18: _1_[31..70]      GNAFSLFQTAISLENR---VRTRTE---ELHSTLRRLEQSNIDLSA        13471220       SHELIX+HISKIN+REC                                                                                            446    Mesorhizobium loti MAFF303099                                        probable two-component sensor [Mesorhizobium loti MAFF303099]                    
39997670 0.00053: _1_[177..216]    ELYSMVRDTLASLEQR---VQERTR---DLAAAREQAESASRAKSD        39997670       SHELIX+HISKIN+REC+REC+HPT                                                                                    850    Geobacter sulfurreducens PCA                                         sensor histidine kinase/response regulator [Geobacter sulfurreducens PCA]        
27366536 0.00016: _1_[134..172]    AKNQELAVHQAGLEKE---IELRTA---DL-KASEELSRTIIDGAP        27366536       SHELIX+PAS+HISKIN+REC+REC+HPT                                                                                1072   Vibrio vulnificus CMCP6                                              FOG: CheY-like receiver [Vibrio vulnificus CMCP6]                                
37676244 0.00022: _1_[134..172]    AKNQELAVHQAGLEKE---IELRTA---DL-KASEELSRTIIDGAP        37676244       SHELIX+PAS+HISKIN+REC+REC+HPT                                                                                1072   Vibrio vulnificus YJ016                                              hypothetical protein VVA0584 [Vibrio vulnificus YJ016]                           
34496337 0.00022: _1_[2..40]       ASNAALSRYQAGLEAE---IVRRTE---ALRQS-EKLSQSIIAGAP        34496337       SHELIX+PAS+HISKIN+REC+REC+HPT                                                                                902    Chromobacterium violaceum ATCC 12472                                 probable sensor/response regulator hybrid protein [Chromobacterium violaceum ATCC 12472]
26989383  0.0001: _1_[233..272]    NTAQALHQAYQHLEQR---VRERTA---ELTSLNDQLLREIEERSQ        26989383       SHELIX+PAS+SHELIX+HISKIN+REC                                                                              655    Pseudomonas putida KT2440                                            sensory box histidine kinase/response regulator [Pseudomonas putida KT2440]      
26989402  0.0001: _1_[170..209]    LAQQRLQQLNDELEQR---VAARTD---ELLEARDAAEAANRSKDK        26989402       PAS+SHELIX+HISKIN+REC                                                                                        567    Pseudomonas putida KT2440                                            sensory box histidine kinase/response regulator [Pseudomonas putida KT2440]      
15837575 7.3e-09: _1_[143..182]    NATRELRLLADTLEHR---VAERTR---DLDQARLDAENANRYKTR        15837575       PAS+SHELIX+HISKIN+REC                                                                                        545    Xylella fastidiosa 9a5c                                              two-component system, sensor protein [Xylella fastidiosa 9a5c]                   
28198192 0.00016: _1_[143..182]    NATRELRLLADTLEHR---VAERTR---DLDQARLEAENANRYKTR        28198192       PAS+SHELIX+HISKIN+REC                                                                                        545    Xylella fastidiosa Temecula1                                         sensor histidine kinase [Xylella fastidiosa Temecula1]                           
15597188 6.1e-11: _1_[159..198]    QAQQRLQTMNEELERR---VAERTH---ELEELNRQLRQARDAAEA        15597188       PAS+SHELIX+HISKIN+REC                                                                                        564    Pseudomonas aeruginosa PAO1                                          probable two-component sensor [Pseudomonas aeruginosa PAO1]                      
42523042  0.0057: _1_[151..190]    ATESKLRSLYQDLEHR---VQERTQ---ELALANKQLKEDASIRQR        42523042       PAS+SHELIX+HISKIN+REC                                                                                        588    Bdellovibrio bacteriovorus HD100                                     two-component hybrid sensor and regulator [Bdellovibrio bacteriovorus HD100]     
16330477 1.8e-08: _1_[284..323]    MIQGQSLEMERRMRQQ---LAEQNQ---ELVAATTAAEAANRSKSE        16330477       REC+PAS+SHELIX+HISKIN+REC+REC+HPT                                                                            1014   Synechocystis sp. PCC 6803                                           hybrid sensory kinase [Synechocystis sp. PCC 6803]                               
42524788  0.0063: _1_[203..242]    VMTEFEGRQRRQLEKQL--VEARNE---ALQAS--HMKSSFLANMS        42524788       TM+CHASE3+TM+SHELIX+HISKIN+REC+HPT                                                                           734    Bdellovibrio bacteriovorus HD100                                     sensory transduction histidine kinase [Bdellovibrio bacteriovorus HD100]         
39996404 0.00053: _1_[291..330]    VMSTELHTFIHNLEEK---VEEKTA---ELILARKDAEEASRAKSD        39996404       TM+HAMP+SHELIX+HISKIN+REC+HPT                                                                                835    Geobacter sulfurreducens PCA                                         sensor histidine kinase/response regulator [Geobacter sulfurreducens PCA]        
24374963 2.7e-05: _1_[244..283]    AMAGSLSEYHDEMQQN---IDQATS---DLRETLEQIEIQNVELDL        24374963       TM+HAMP+SHELIX+HISKIN+REC+HPT                                                                                929    Shewanella oneidensis MR-1                                           sensor histidine kinase/response regulator [Shewanella oneidensis MR-1]          
39997026 0.00053: _1_[235..274]    ESDANLKRNREDLGEE---VARRTE---DLSRTNEELERAVADLSK        39997026       TM+HAMP+SHELIX+HISKIN+REC+HPT                                                                                793    Geobacter sulfurreducens PCA                                         sensor histidine kinase/response regulator [Geobacter sulfurreducens PCA]        
28868897 0.00018: _1_[234..273]    RMAATLQNAQEELQMS---IDQATE---DVRQNLETIEIQNIELDL        28868897       TM+HAMP+SHELIX+HISKIN+REC+REC+HPT                                                                            917    Pseudomonas syringae pv. tomato str. DC3000                          sensor histidine kinase/response regulator GacS [Pseudomonas syringae pv. tomato str. DC3000]
54310182   0.055: _1_[215..254]    AMAISLSEYHIEMQQS---IDQATS---DLRETLEQLEIQNVELDI        54310182       TM+HAMP+SHELIX+HISKIN+REC+REC+HPT                                                                            903    Photobacterium profundum SS9                                         putative sensor histidine kinase/response regulator [Photobacterium profundum SS9]
24112406 4.4e-06: _1_[305..344]    SSVHALNRHREQLAAQ---VKARTA---ELQELVIEHRQARAEAEK        24112406       TM+HAMP+REC+SHELIX+HISKIN+REC+HPT                                                                            809    Shigella flexneri 2a str. 301                                        similar to histidine protein kinase/phosphatase, aerobic respiration sensor-response protein, sensor for arcA [Shigella flexneri 2a str. 301]
26988605  0.0001: _1_[255..294]    TAENRLTQYLNELEDI---VSARTN---ELKASNSSLSLSNQELEQ        26988605       TM+SHELIX+HISKIN+REC+REC                                                                                     785    Pseudomonas putida KT2440                                            sensor histidine kinase/response regulator [Pseudomonas putida KT2440]           
50086083   0.027: _1_[58..97]      ALLTRYEHTASNLLEL---VERRPE---QYDQAQRIMQNMFNERYL        50086083       TM+SHELIX+TM+HISKIN+REC+HPT                                                                                  932    Acinetobacter sp. ADP1                                               GacS-like sensor kinase protein [Acinetobacter sp. ADP1]                         
56477575    0.82: _1_[232..271]    QMAAKLASAHEDMSRQ---IADATA---ELRARKDEAERATLSKSR        56477575       TM+TM+HAMP+SHELIX+HISKIN+REC                                                                                 632    Azoarcus sp. EbN1                                                    complex two-component hybrid sensor component containing histidine kinase and response receiver domain [Azoarcus sp. EbN1]
16767111 2.8e-07: _1_[401..440]    ANVRKLNRHREDLAEQ---VRSQTA---ELHALVLEHRQARAEAEK        16767111       TM+TM+HAMP+SHELIX+HISKIN+REC+HPT                                                                             911    Salmonella typhimurium LT2                                           sensor kinase [Salmonella typhimurium LT2]                                       
37681007 0.00048: _1_[240..279]    AMAISLSEYHVEMQHS---IDQATS---DLRETLEQLEIQNVELDI        37681007       TM+TM+HAMP+SHELIX+HISKIN+REC+HPT                                                                             928    Vibrio vulnificus YJ016                                              signal transduction histidine kinase [Vibrio vulnificus YJ016]                   
15803307 2.5e-09: _1_[242..281]    SMAMSLAAYHEEMQHN---IDQATS---DLRETLEQMEIQNVELDL        15803307       TM+TM+HAMP+SHELIX+HISKIN+REC+HPT                                                                             918    Escherichia coli O157:H7 EDL933                                      sensor-regulator, activates OmpR by phophorylation [Escherichia coli O157:H7 EDL933]
16762498 1.1e-07: _1_[401..440]    ANVRKLNRHREDLAEQ---VRSQTA---ELHALVLEHRQARAEAEK        16762498       TM+TM+HAMP+SHELIX+HISKIN+REC+HPT                                                                             911    Salmonella enterica subsp. enterica serovar Typhi str. CT18          Two-component sensor protein histidine protein kinase. [Salmonella enterica subsp. enterica serovar Typhi str. CT18]
15596125 1.1e-11: _1_[233..272]    RMAETLQSAQEEMQHN---IDQATE---DVRQNLETIEIQNIELDL        15596125       TM+TM+HAMP+SHELIX+HISKIN+REC+HPT                                                                             925    Pseudomonas aeruginosa PAO1                                          sensor/response regulator hybrid [Pseudomonas aeruginosa PAO1]                   
24372811   5e-06: _1_[457..496]    QVADELTEHKKALEQT---VATRTQ---ELAETNLRLDAEAKGHAK        24372811       TM+TM+HAMP+SHELIX+HISKIN+REC+HPT                                                                             1025   Shewanella oneidensis MR-1                                           sensor histidine kinase/response regulator TorS [Shewanella oneidensis MR-1]     
27364942 0.00013: _1_[240..279]    AMAISLSEYHVEMQHS---IDQATS---DLRETLEQLEIQNVELDI        27364942       TM+TM+HAMP+SHELIX+HISKIN+REC+HPT                                                                             928    Vibrio vulnificus CMCP6                                              Signal transduction histidine kinase [Vibrio vulnificus CMCP6]                   
50122492   0.027: _1_[242..281]    SMAMSLTAYHEEMQQN---IDQATY---DLRETLEQMEIQNVELDL        50122492       TM+TM+HAMP+SHELIX+HISKIN+REC+HPT                                                                             933    Erwinia carotovora subsp. atroseptica SCRI1043                       two-component sensor kinase and response regulator [Erwinia carotovora subsp. atroseptica SCRI1043]
37676294 0.00022: _1_[434..473]    KAKKELQEHKEHLEEL---VTERTR---QLQLTNEKLNHEVLNHAK        37676294       TM+TM+HAMP+SHELIX+HISKIN+REC+HPT                                                                             984    Vibrio vulnificus YJ016                                              signal transduction histidine kinase [Vibrio vulnificus YJ016]                   
27366581 0.00016: _1_[420..459]    KAKKELQEHKEHLEEL---VTERTR---QLQLTNEKLNHEVLNHAK        27366581       TM+TM+HAMP+SHELIX+HISKIN+REC+HPT                                                                             970    Vibrio vulnificus CMCP6                                              FOG: CheY-like receiver [Vibrio vulnificus CMCP6]                                
15601465 5.3e-10: _1_[455..494]    LAKRELQQHKEHLEEL---VEQRTC---QLSEMNHKLNQEVLNHAK        15601465       TM+TM+HAMP+SHELIX+HISKIN+REC+HPT                                                                             1020   Vibrio cholerae O1 biovar eltor str. N16961                          sensor protein TorS [Vibrio cholerae O1 biovar eltor str. N16961]                
54308425   0.055: _1_[419..458]    KIRQELQLHKESLERL---VAQRTS---ELEKTNSRLNEEVINHEK        54308425       TM+TM+HAMP+SHELIX+HISKIN+REC+HPT                                                                             983    Photobacterium profundum SS9                                         Hypothetical sensor protein TorS [Photobacterium profundum SS9]                  
56697871    0.92: _1_[240..279]    RTVTELEASRDNLQEL---VDERTR---ELLEARNEAVEASQAKSI        56697871       TM+TM+HAMP+SHELIX+HISKIN+REC+HPT                                                                             898    Silicibacter pomeroyi DSS-3                                          sensor histidine kinase/response regulator [Silicibacter pomeroyi DSS-3]         
26990435  0.0001: _1_[231..270]    RDRLELQDKRDELQAM---VERRTA---SLARAKDEAEAANLAKSR        26990435       TM+TM+HAMP+SHELIX+HISKIN+REC+HPT                                                                             750    Pseudomonas putida KT2440                                            sensor histidine kinase/response regulator [Pseudomonas putida KT2440]           
37524900 0.00022: _1_[241..280]    AMAKSLSSYKEEMQND---IDQATS---DLRETMEQFEIQNVELAI        37524900       TM+TM+HAMP+SHELIX+HISKIN+REC+HPT                                                                             775    Photorhabdus luminescens subsp. laumondii TTO1                       Sensor protein BarA [Photorhabdus luminescens subsp. laumondii TTO1]             
28900530 0.00022: _1_[419..458]    KAKRELEEHKEHLEEL---IEQRTS---QLRQANLRLNEEVVNHAQ        28900530       TM+TM+HAMP+SHELIX+HISKIN+REC+HPT                                                                             988    Vibrio parahaemolyticus RIMD 2210633                                 sensor protein TorS [Vibrio parahaemolyticus RIMD 2210633]                       
15600175 4.4e-10: _1_[470..509]    TDRRRRDADRDELRRQ---VERRTA---SLRRAKDQAEAADRAKSR        15600175       TM+TM+HAMP+SHELIX+HISKIN+REC+HPT                                                                             998    Pseudomonas aeruginosa PAO1                                          probable two-component sensor [Pseudomonas aeruginosa PAO1]                      
29143986 0.00022: _1_[401..440]    ANVRKLNRHREDLAEQ---VRSQTA---ELHALVLEHRQARAEAEK        29143986       TM+TM+HAMP+SHELIX+HISKIN+REC+HPT                                                                             911    Salmonella enterica subsp. enterica serovar Typhi Ty2                two-component sensor protein histidine protein kinase. [Salmonella enterica subsp. enterica serovar Typhi Ty2]
56415706    0.24: _1_[401..440]    ANVRKLNRHREDLAEQ---VRSQTA---ELHALVLEHRQARAEAEK        56415706       TM+TM+HAMP+SHELIX+HISKIN+REC+HPT                                                                             911    Salmonella enterica subsp. enterica serovar Paratyphi A str. ATCC    two-component sensor protein histidine protein kinase. [Salmonella enterica subsp. enterica serovar Paratyphi A str. ATCC 9150]
29143221 0.00022: _1_[242..281]    SMAMSLAAYHEEMQHN---IDQATS---DLRETLEQMEIQNVELDL        29143221       TM+TM+HAMP+SHELIX+HISKIN+REC+REC+HPT                                                                         918    Salmonella enterica subsp. enterica serovar Typhi Ty2                sensor protein [Salmonella enterica subsp. enterica serovar Typhi Ty2]           
28899341 0.00022: _1_[240..279]    AMAVSLSEYHVEMQHS---IDQATS---DLRETLEQLEIQNVELDI        28899341       TM+TM+HAMP+SHELIX+HISKIN+REC+REC+HPT                                                                         932    Vibrio parahaemolyticus RIMD 2210633                                 sensor histidine kinase/response regulator [Vibrio parahaemolyticus RIMD 2210633]
56414910    0.12: _1_[242..281]    SMAMSLAAYHEEMQHN---IDQATS---DLRETLEQMEIQNVELDL        56414910       TM+TM+HAMP+SHELIX+HISKIN+REC+REC+HPT                                                                         918    Salmonella enterica subsp. enterica serovar Paratyphi A str. ATCC    sensor protein [Salmonella enterica subsp. enterica serovar Paratyphi A str. ATCC 9150]
15642449 1.7e-09: _1_[240..279]    AMAVSLSEYHVEMQHS---IDQATS---DLRETLEQLEIQNVELDI        15642449       TM+TM+HAMP+SHELIX+HISKIN+REC+REC+HPT                                                                         927    Vibrio cholerae O1 biovar eltor str. N16961                          sensor histidine kinase/response regulator [Vibrio cholerae O1 biovar eltor str. N16961]
15832900 3.4e-09: _1_[242..281]    SMAMSLAAYHEEMQHN---IDQATS---DLRETLEQMEIQNVELDL        15832900       TM+TM+HAMP+SHELIX+HISKIN+REC+REC+HPT                                                                         918    Escherichia coli O157:H7                                             BarA [Escherichia coli O157:H7]                                                  
26988382   8e-05: _1_[235..274]    RMAETLQNAHEELQHS---IDQATE---DVRQNLETIEIQNIELDM        26988382       TM+TM+HAMP+SHELIX+HISKIN+REC+REC+HPT                                                                         917    Pseudomonas putida KT2440                                            sensor histidine kinase/response regulator GacS [Pseudomonas putida KT2440]      
26249187 5.1e-05: _1_[242..281]    SMAMSLAAYHEEMQHN---IDQATS---DLRETLEQMEIQNVELDL        26249187       TM+TM+HAMP+SHELIX+HISKIN+REC+REC+HPT                                                                         918    Escherichia coli CFT073                                              Sensor protein barA [Escherichia coli CFT073]                                    
24114070 4.4e-06: _1_[242..281]    SMAMSLAAYHEEMQHN---IDQATS---DLRETLEQMEIQNVELDL        24114070       TM+TM+HAMP+SHELIX+HISKIN+REC+REC+HPT                                                                         918    Shigella flexneri 2a str. 301                                        sensor-regulator [Shigella flexneri 2a str. 301]                                 
22124721 8.7e-07: _1_[241..280]    SMAMSLAAYHEEMQQN---IDQATS---DLRETLEQMEIQNVELGL        22124721       TM+TM+HAMP+SHELIX+HISKIN+REC+REC+HPT                                                                         933    Yersinia pestis KIM                                                  sensor-regulator, activates OmpR by phophorylation [Yersinia pestis KIM]         
16766264 2.1e-07: _1_[242..281]    SMAMSLAAYHEEMQHN---IDQATS---DLRETLEQMEIQNVELDL        16766264       TM+TM+HAMP+SHELIX+HISKIN+REC+REC+HPT                                                                         918    Salmonella typhimurium LT2                                           sensor histidine kinase [Salmonella typhimurium LT2]                             
16761737 8.6e-08: _1_[242..281]    SMAMSLAAYHEEMQHN---IDQATS---DLRETLEQMEIQNVELDL        16761737       TM+TM+HAMP+SHELIX+HISKIN+REC+REC+HPT                                                                         918    Salmonella enterica subsp. enterica serovar Typhi str. CT18          sensor protein [Salmonella enterica subsp. enterica serovar Typhi str. CT18]     
45440163  0.0063: _1_[241..280]    SMAMSLAAYHEEMQQN---IDQATS---DLRETLEQMEIQNVELGL        45440163       TM+TM+HAMP+SHELIX+HISKIN+REC+REC+HPT                                                                         921    Yersinia pestis biovar Medievalis str. 91001                         two-component regulatory protein [Yersinia pestis biovar Medievalis str. 91001]  
16130693 1.2e-08: _1_[242..281]    SMAMSLAAYHEEMQHN---IDQATS---DLRETLEQMEIQNVELDL        16130693       TM+TM+HAMP+SHELIX+HISKIN+REC+REC+HPT                                                                         918    Escherichia coli K12                                                 hybrid sensory histidine kinase in two-component regulatory system with UvrY [Escherichia coli K12]
16123530 9.7e-09: _1_[241..280]    SMAMSLAAYHEEMQQN---IDQATS---DLRETLEQMEIQNVELGL        16123530       TM+TM+HAMP+SHELIX+HISKIN+REC+REC+HPT                                                                         930    Yersinia pestis CO92                                                 two-component regulatory protein [Yersinia pestis CO92]                          
51595100   0.027: _1_[241..280]    SMAMSLAAYHEEMQQN---IDQATS---DLRETLEQMEIQNVELGL        51595100       TM+TM+HAMP+SHELIX+HISKIN+REC+REC+HPT                                                                         942    Yersinia pseudotuberculosis IP 32953                                 bifunctional multimodular BarA: sensory histidine kinase in two component regulatory system, activates OmpR [Yersinia pseudotuberculosis IP 32953]
52842130   0.027: _1_[247..286]    HLQRQYLNTVRDLNHH---IEIATA---DLQQSLELLEEKNIELSL        52842130       TM+TM+HAMP+SHELIX+HISKIN+REC+REC+HPT                                                                         910    Legionella pneumophila subsp. pneumophila str. Philadelphia 1        sensory box histidine kinase/response regulator [Legionella pneumophila subsp. pneumophila str. Philadelphia 1]
54294799    0.05: _1_[247..286]    HLQRQYLNTVRDLNHH---IEIATA---DLQQSLELLEEKNIELSL        54294799       TM+TM+HAMP+SHELIX+HISKIN+REC+REC+HPT                                                                         910    Legionella pneumophila str. Lens                                     Legionella transmission sensor LetS [Legionella pneumophila str. Lens]           
54297835   0.055: _1_[247..286]    HLQRQYLNTVRDLNHH---IEIATA---DLQQSLELLEEKNIELSL        54297835       TM+TM+HAMP+SHELIX+HISKIN+REC+REC+HPT                                                                         910    Legionella pneumophila str. Paris                                    Legionella transmission sensor LetS [Legionella pneumophila str. Paris]          
30064131 0.00022: _1_[242..281]    SMAMSLAAYHEEMQHN---IDQATS---DLRETLEQMEIQNVELDL        30064131       TM+TM+HAMP+SHELIX+HISKIN+REC+REC+HPT                                                                         918    Shigella flexneri 2a str. 2457T                                      sensor-regulator [Shigella flexneri 2a str. 2457T]                               
15830402 3.1e-09: _1_[390..429]    SSVHALNRHREQLAAQ---VKARTA---ELQELVIEHRQARAEAEK        15830402       TM+TM+HAMP+REC+SHELIX+HISKIN+REC+HPT                                                                         904    Escherichia coli O157:H7                                             TorS [Escherichia coli O157:H7]                                                  
15800913 2.1e-09: _1_[390..429]    SSVHALNRHREQLAAQ---VKARTA---ELQELVIEHRQARAEAEK        15800913       TM+TM+HAMP+REC+SHELIX+HISKIN+REC+HPT                                                                         904    Escherichia coli O157:H7 EDL933                                      sensor protein torS (regulator TorR) [Escherichia coli O157:H7 EDL933]           
16128959 9.7e-09: _1_[390..429]    SNVHALNRHREQLAAQ---VKARTA---ELQELVIEHRQARAEAEK        16128959       TM+TM+HAMP+REC+SHELIX+HISKIN+REC+HPT                                                                         904    Escherichia coli K12                                                 hybrid sensory histidine kinase in two-component regulatory system with TorR, regulates TMAO reductase regulatory system I, senses TMAO [Escherichia coli K12]
15598020 4.3e-10: _1_[255..294]    EAENRLTQYLEELESI---VAARTA---ELKAANARLTLSNQELEE        15598020       TM+TM+SHELIX+HISKIN+REC+REC                                                                                  786    Pseudomonas aeruginosa PAO1                                          probable sensor/response regulator hybrid [Pseudomonas aeruginosa PAO1]          
28868988 0.00018: _1_[241..280]    AAEDRLTDHLNELENI---VSARTN---ELKSSNMRLRESNEELQI        28868988       TM+TM+TM+SHELIX+HISKIN+REC                                                                                   783    Pseudomonas syringae pv. tomato str. DC3000                          sensor histidine kinase/response regulator [Pseudomonas syringae pv. tomato str. DC3000]
# 42     :                                                                        # 42
37676848 2.4e-08: _1_[218..257]    NMAADLGKLYRGLEQA---VNEKTH---KLQHANQSLQVLYHSSQE        37676848       TM+NarQ+TM+TM+HAMP+SHELIX+HISKIN                                                                             571    Vibrio vulnificus YJ016                                              nitrate/nitrite sensor protein NarQ [Vibrio vulnificus YJ016]                    
15601432 6.8e-07: _1_[227..266]    SMAGDLGKLYRGLEHA---VNEKTN---KLQMANQSLQVLYHSSQE        15601432       TM+NarQ+TM+HAMP+SHELIX+GAF+HISKIN                                                                            583    Vibrio cholerae O1 biovar eltor str. N16961                          nitrate/nitrite sensor protein NarQ [Vibrio cholerae O1 biovar eltor str. N16961]
15603456 1.3e-08: _1_[217..256]    QMSSELEKLYSSLEES---VNEKTQ---KLRQTNRSLTMLYESSQQ        15603456       TM+NarQ+TM+HAMP+SHELIX+GAF+HISKIN                                                                            568    Pasteurella multocida subsp. multocida str. Pm70                     NarQ [Pasteurella multocida subsp. multocida str. Pm70]                          
15677120 8.9e-09: _1_[228..267]    QMGGRLKILYDDLEGQ---VAEQTR---SLEKQNQNLTLLYQTTRD        15677120       TM+NarQ+TM+HAMP+SHELIX+GAF+HISKIN                                                                            590    Neisseria meningitidis MC58                                          putative nitrate/nitrite sensory protein NarX [Neisseria meningitidis MC58]      
15794330 8.9e-09: _1_[228..267]    QMGGRLKILYDDLEGQ---VAEQTR---SLEKQNQNLTLLYQTTRD        15794330       TM+NarQ+TM+HAMP+SHELIX+GAF+HISKIN                                                                            590    Neisseria meningitidis Z2491                                         two-component system sensor kinase [Neisseria meningitidis Z2491]                
56477413   3e-11: _1_[256..295]    AMADELSTVYRDLEQR---VEQKTA---DLTRSNRALELLYHVIAR        56477413       TM+NarQ+TM+HAMP+SHELIX+GAF+HISKIN                                                                            648    Azoarcus sp. EbN1                                                    nitrate/nitrite TWO-component sensor NARX [Azoarcus sp. EbN1]                    
53724170 2.6e-10: _1_[241..280]    RMASELQEVYAGLEER---VQQKTA---QLAAQNRELSALYEITAF        53724170       TM+NarQ+TM+HAMP+SHELIX+GAF+HISKIN                                                                            640    Burkholderia mallei ATCC 23344                                       nitrate/nitrite sensory protein NarX, putative [Burkholderia mallei ATCC 23344]  
53719923 2.6e-10: _1_[241..280]    RMASELQEVYAGLEER---VQQKTA---QLAAQNRELSALYEITAF        53719923       TM+NarQ+TM+HAMP+SHELIX+GAF+HISKIN                                                                            644    Burkholderia pseudomallei K96243                                     putative nitrate/nitrite sensor protein [Burkholderia pseudomallei K96243]       
50120830 4.8e-08: _1_[217..256]    SMTDELQKLYRSLEDK---VRQKTL---RLQEVNRMLKVLYNCSQA        50120830       TM+NarQ+TM+HAMP+SHELIX+GAF+HISKIN                                                                            573    Erwinia carotovora subsp. atroseptica SCRI1043                       nitrate/nitrite sensor protein [Erwinia carotovora subsp. atroseptica SCRI1043]  
15599073 1.3e-10: _1_[234..273]    QMAGELKSIYGDLEER---VEDKTR---ALSQSHQRLELLYASARR        15599073       TM+NarQ+TM+HAMP+SHELIX+GAF+HISKIN                                                                            622    Pseudomonas aeruginosa PAO1                                          two-component sensor NarX [Pseudomonas aeruginosa PAO1]                          
34497989 6.8e-10: _1_[230..269]    QMAERAQDLYQNLESK---VAQKTR---EVEEQNLRLKTLYDMTSF        34497989       TM+NarQ+TM+HAMP+SHELIX+GAF+HISKIN                                                                            632    Chromobacterium violaceum ATCC 12472                                 transmembrane nitrate/nitrite sensor kinase transcription regulator protein [Chromobacterium violaceum ATCC 12472]
28901051 6.7e-09: _1_[218..257]    RMATDLGKLYRGLEQA---VDEKTR---KLQHANQSLEVLYDSSKE        28901051       TM+NarQ+TM+HAMP+SHELIX+GAF+HISKIN                                                                            578    Vibrio parahaemolyticus RIMD 2210633                                 nitrate/nitrite sensor protein NarQ [Vibrio parahaemolyticus RIMD 2210633]       
16272225 3.6e-08: _1_[218..257]    QMSTELGQLYSRLEEA---VNEKTQ---KLRQTNRTLSTLYQSAQL        16272225       TM+NarQ+TM+HAMP+SHELIX+GAF+HISKIN                                                                            567    Haemophilus influenzae Rd KW20                                       nitrate/nitrite sensor protein [Haemophilus influenzae Rd KW20]                  
56479028 7.1e-09: _1_[243..282]    RMAVHLQGLYATLEER---VAAKTR---SLEGKNRELEILYDIGAF        56479028       TM+NarQ+TM+HAMP+SHELIX+GAF+HISKIN                                                                            651    Azoarcus sp. EbN1                                                    nitrate/nitrite sensor kinase [Azoarcus sp. EbN1]                                
17549200 1.4e-09: _1_[228..267]    RMAEELQGLYADLASR---VARKTE---ELAEQNRELSALYDITAF        17549200       TM+NarQ+TM+HAMP+SHELIX+GAF+HISKIN                                                                            637    Ralstonia solanacearum GMI1000                                       PUTATIVE TRANSMEMBRANE NITRATE/NITRITE SENSOR KINASE TRANSCRIPTION REGULATOR PROTEIN [Ralstonia solanacearum GMI1000]
16129185 2.5e-08: _1_[218..257]    NMSAELAESYAVLEQR---VQEKTA---GLEHKNQILSFLWQANRR        16129185       TM+NarQ+TM+HAMP+SHELIX+GAF+HISKIN                                                                                598    Escherichia coli K12                                                 nitrate/nitrate sensor, histidine protein kinase acts on NarL regulator [Escherichia coli K12]
50120957   6e-11: _1_[218..257]    SMSDELSAMYHSLEQR---VAEKTA---DLQQKNDLLSFLYRASRR        50120957       TM+NarQ+TM+HAMP+SHELIX+GAF+HISKIN                                                                                602    Erwinia carotovora subsp. atroseptica SCRI1043                       nitrate/nitrite sensor kinase [Erwinia carotovora subsp. atroseptica SCRI1043]   
26248838 3.9e-09: _1_[222..261]    QMSSELHKLYRSLEAS---VEEKTR---DLHEAKRRLEVLYQCSQA        26248838       TM+NarQ+TM+HAMP+SHELIX+GAF+HISKIN                                                                                571    Escherichia coli CFT073                                              Nitrate/nitrite sensor protein narQ [Escherichia coli CFT073]                    
26247547 2.5e-08: _1_[218..257]    NMSAELAESYAVLEQR---VQEKTA---GLEHKNQILSFLWQANRR        26247547       TM+NarQ+TM+HAMP+SHELIX+GAF+HISKIN                                                                                598    Escherichia coli CFT073                                              Nitrate/nitrite sensor protein narX [Escherichia coli CFT073]                    
16130394 3.9e-09: _1_[217..256]    QMSSELHKLYRSLEAS---VEEKTR---DLHEAKRRLEVLYQCSQA        16130394       TM+NarQ+TM+HAMP+SHELIX+GAF+HISKIN                                                                                566    Escherichia coli K12                                                 sensor for nitrate reductase system, protein histidine kinase (acts on NarP and narL) [Escherichia coli K12]
15830981 2.5e-08: _1_[218..257]    NMSAELAESYAVLEQR---VQEKTA---GLEHKNQILSFLWQANRR        15830981       TM+NarQ+TM+HAMP+SHELIX+GAF+HISKIN                                                                                598    Escherichia coli O157:H7                                             NarX [Escherichia coli O157:H7]                                                  
15832585 3.9e-09: _1_[217..256]    QMSSELHKLYRSLEAS---VEEKTR---DLHEAKRRLEVLYQCSQA        15832585       TM+NarQ+TM+HAMP+SHELIX+GAF+HISKIN                                                                                566    Escherichia coli O157:H7                                             NarQ [Escherichia coli O157:H7]                                                  
15802991 3.9e-09: _1_[217..256]    QMSSELHKLYRSLEAS---VEEKTR---DLHEAKRRLEVLYQCSQA        15802991       TM+NarQ+TM+HAMP+SHELIX+GAF+HISKIN                                                                                566    Escherichia coli O157:H7 EDL933                                      sensor for nitrate reductase system, protein histidine kinase (acts on NarP and narL) [Escherichia coli O157:H7 EDL933]
15801453 2.5e-08: _1_[218..257]    NMSAELAESYAVLEQR---VQEKTA---GLEHKNQILSFLWQANRR        15801453       TM+NarQ+TM+HAMP+SHELIX+GAF+HISKIN                                                                                598    Escherichia coli O157:H7 EDL933                                      nitrate/nitrate sensor, histidine protein kinase acts on NarL regulator [Escherichia coli O157:H7 EDL933]
56459295 3.3e-06: _1_[220..259]    KMSETVSYFYGGLERR---VEQQTQ---ELSRKNKVLSFLYDTARS        56459295       TM+NarQ+TM+HAMP+SHELIX+GAF+HISKIN                                                                                598    Idiomarina loihiensis L2TR                                           Nitrate/nitrite sensor histidine kinase [Idiomarina loihiensis L2TR]             
52426343 3.7e-08: _1_[236..275]    HMSNELHKLYANLEEK---VTEKTQ---KINQVNRSLAMLYYCSQE        52426343       TM+NarQ+TM+HAMP+SHELIX+GAF+HISKIN                                                                                585    Mannheimia succiniciproducens MBEL55E                                BaeS protein [Mannheimia succiniciproducens MBEL55E]                             
54308045 1.5e-09: _1_[218..257]    KMSSELGKLYRGLEQK---VDEKTR---RLRQANESLQVLYNCSQE        54308045       TM+NarQ+TM+HAMP+SHELIX+GAF+HISKIN                                                                                565    Photobacterium profundum SS9                                         hypothetical nitrate/nitrite sensor protein NarQ [Photobacterium profundum SS9]  
16761397 3.6e-09: _1_[217..256]    QMSSELHKLYRSLEAS---VEEKTH---DLHEAHRRLEVLYQCSQA        16761397       TM+NarQ+TM+HAMP+SHELIX+GAF+HISKIN                                                                                566    Salmonella enterica subsp. enterica serovar Typhi str. CT18          nitrate/nitrite sensor protein NarQ [Salmonella enterica subsp. enterica serovar Typhi str. CT18]
56413312   2e-08: _1_[218..257]    NMSEELAESYAVLEQR---VQEKTA---GLEHKNQILSFLWQANRR        56413312       TM+NarQ+TM+HAMP+SHELIX+GAF+HISKIN                                                                                598    Salmonella enterica subsp. enterica serovar Paratyphi A str. ATCC    nitrate/nitrite sensor protein NarX [Salmonella enterica subsp. enterica serovar Paratyphi A str. ATCC 9150]
16760120   2e-08: _1_[218..257]    NMSEELAESYAVLEQR---VQEKTA---GLEHKNQILSFLWQANRR        16760120       TM+NarQ+TM+HAMP+SHELIX+GAF+HISKIN                                                                                598    Salmonella enterica subsp. enterica serovar Typhi str. CT18          nitrate/nitrite sensor protein NarX [Salmonella enterica subsp. enterica serovar Typhi str. CT18]
29140906 3.6e-09: _1_[217..256]    QMSSELHKLYRSLEAS---VEEKTH---DLHEAHRRLEVLYQCSQA        29140906       TM+NarQ+TM+HAMP+SHELIX+GAF+HISKIN                                                                                566    Salmonella enterica subsp. enterica serovar Typhi Ty2                nitrate/nitrite sensor protein [Salmonella enterica subsp. enterica serovar Typhi Ty2]
29142109   2e-08: _1_[218..257]    NMSEELAESYAVLEQR---VQEKTA---GLEHKNQILSFLWQANRR        29142109       TM+NarQ+TM+HAMP+SHELIX+GAF+HISKIN                                                                                598    Salmonella enterica subsp. enterica serovar Typhi Ty2                nitrate/nitrite sensor protein NarX [Salmonella enterica subsp. enterica serovar Typhi Ty2]
16765800 3.6e-09: _1_[217..256]    QMSSELHKLYRSLEAS---VEEKTH---DLHEAHRRLEVLYQCSQA        16765800       TM+NarQ+TM+HAMP+SHELIX+GAF+HISKIN                                                                                566    Salmonella typhimurium LT2                                           sensory histidine kinase [Salmonella typhimurium LT2]                            
16765107   2e-08: _1_[218..257]    NMSEELAESYAVLEQR---VQEKTA---GLEHKNQILSFLWQANRR        16765107       TM+NarQ+TM+HAMP+SHELIX+GAF+HISKIN                                                                                598    Salmonella typhimurium LT2                                           sensory histidine kinase [Salmonella typhimurium LT2]                            
24375468 1.5e-08: _1_[202..241]    KTARELATLYGNLESQ---VAEKTL---ALTRANNELAFLYDTLLT        24375468       TM+NarQ+TM+HAMP+SHELIX+GAF+HISKIN                                                                                585    Shewanella oneidensis MR-1                                           nitrate/nitrite sensor protein NarQ [Shewanella oneidensis MR-1]                 
30062746 2.5e-08: _1_[218..257]    NMSAELAESYAVLEQR---VQEKTA---GLEHKNQILSFLWQANRR        30062746       TM+NarQ+TM+HAMP+SHELIX+GAF+HISKIN                                                                                598    Shigella flexneri 2a str. 2457T                                      nitrate/nitrite sensor protein NarX [Shigella flexneri 2a str. 2457T]            
24112621 2.5e-08: _1_[218..257]    NMSAELAESYAVLEQR---VQEKTA---GLEHKNQILSFLWQANRR        24112621       TM+NarQ+TM+HAMP+SHELIX+GAF+HISKIN                                                                                598    Shigella flexneri 2a str. 301                                        nitrate/nitrite sensor protein NarX [Shigella flexneri 2a str. 301]              
27367137 2.4e-08: _1_[202..241]    NMAADLGKLYRGLEQA---VNEKTH---KLQHANQSLQVLYHSSQE        27367137       TM+NarQ+TM+HAMP+SHELIX+GAF+HISKIN                                                                                555    Vibrio vulnificus CMCP6                                              Signal transduction histidine kinase, nitrate/nitrite-specific [Vibrio vulnificus CMCP6]
45441517 1.2e-07: _1_[224..263]    RMSQELSLIYGDLEQR---VVNKTA---DLQQKNQVLAFLYHSSRQ        45441517       TM+NarQ+TM+HAMP+SHELIX+GAF+HISKIN                                                                                601    Yersinia pestis biovar Medievalis str. 91001                         nitrate/nitrite sensor protein [Yersinia pestis biovar Medievalis str. 91001]    
16122205 1.2e-07: _1_[216..255]    RMSQELSLIYGDLEQR---VVNKTA---DLQQKNQVLAFLYHSSRQ        16122205       TM+NarQ+TM+HAMP+SHELIX+GAF+HISKIN                                                                                593    Yersinia pestis CO92                                                 nitrate/nitrite sensor protein [Yersinia pestis CO92]                            
22126235 1.2e-07: _1_[224..263]    RMSQELSLIYGDLEQR---VVNKTA---DLQQKNQVLAFLYHSSRQ        22126235       TM+NarQ+TM+HAMP+SHELIX+GAF+HISKIN                                                                                601    Yersinia pestis KIM                                                  nitrate/nitrite sensor [Yersinia pestis KIM]                                     
51596283 1.2e-07: _1_[216..255]    RMSQELSLIYGDLEQR---VVNKTA---DLQQKNQVLAFLYHSSRQ        51596283       TM+NarQ+TM+HAMP+SHELIX+GAF+HISKIN                                                                                593    Yersinia pseudotuberculosis IP 32953                                 nitrate/nitrite sensor protein [Yersinia pseudotuberculosis IP 32953]            
# 41            :                                                                        # 41
15805493 3.3e-10: _1_[317..356]    RTQEALRELNAQLEER---VEARTA---ELEALSRQLQHDALHDSL        15805493       PAS+PAS+SHELIX+GGDEF+EAL                                                                                     805    Deinococcus radiodurans R1                                           sensory box/GGDEF family protein [Deinococcus radiodurans R1]                    
28867764    0.57: _1_[531..570]    NADAAMYRSKAQGRNR---VESYTR---DLTEQASERIALEQELRR        28867764       PAS+PAS+PAS+GGDEF+SHELIX+EAL                                                                                 829    Pseudomonas syringae pv. tomato str. DC3000                          sensory box/GGDEF domain/EAL domain protein [Pseudomonas syringae pv. tomato str. DC3000]
52840532 2.3e-06: _1_[160..199]    VLARETQKHTEFLNQL---VEERTE---SLQQSFSLLRATIESSSD        52840532       REC+SHELIX+PAS+GGDEF+EAL                                                                                     740    Legionella pneumophila subsp. pneumophila str. Philadelphia 1        sensory box protein, GGDEF family protein, LssE [Legionella pneumophila subsp. pneumophila str. Philadelphia 1]
54293281 2.3e-06: _1_[160..199]    VLARETQKHTEFLNQL---VEERTE---SLQQSFSLLRATIESSSD        54293281       REC+SHELIX+PAS+GGDEF+EAL                                                                                     740    Legionella pneumophila str. Lens                                     hypothetical protein lpl0329 [Legionella pneumophila str. Lens]                  
46578824 1.9e-08: _1_[126..165]    RLLDERQRYQERLEFE---VEARTR---ELRVSNEQLRSTQSELED        46578824       REC+SHELIX+PAS+GGDEF+EAL                                                                                     715    Desulfovibrio vulgaris subsp. vulgaris str. Hildenborough            response regulator/sensory box/GGDEF domain/EAL domain protein [Desulfovibrio vulgaris subsp. vulgaris str. Hildenborough]
34497033 0.00011: _2_[428..466]    KSARRVQVYEEELGQQ---MRERV----ELEKSLRHAIDHGELVLH        34497033       REC+SHELIX+PAS+GGDEF+SHELIX+EAL                                                                           712    Chromobacterium violaceum ATCC 12472                                 probable sensory box/GGDEF family protein [Chromobacterium violaceum ATCC 12472] 
17232389 4.6e-11: _1_[213..252]    RIEKSLQQAHDQLENK---VKERTS---EIIKTHDKLKSEIHEHKQ        17232389       TM+CHASE3+PAS+SHELIX+PAS+GGDEF+EAL                                                                           821    Nostoc sp. PCC 7120                                                  hypothetical protein all4897 [Nostoc sp. PCC 7120]                               
56459429  0.0032: _1_[263..302]    LLFLTVVGFNLLLRRK---VTEKTQ---ALAESKYRLNTILDSVEA        56459429       TM+SHELIX+PAS+GGDEF+EAL                                                                                      850    Idiomarina loihiensis L2TR                                           Signaling protein with a periplasmic amino acid-binding domain, GGDEF and EAL domains [Idiomarina loihiensis L2TR]
53804274    0.21: _1_[122..157]    PSPRQLLELNQHLASE---VSER-------KQAEQRLRMLIDAEPE        53804274       TM+TM+SHELIX+PAS+PAS+PAS+GGDEF+EAL                                                                           819    Methylococcus capsulatus str. Bath                                   sensory box protein/GGDEF domain protein [Methylococcus capsulatus str. Bath]    
56476956 0.00028: _1_[63..102]     NQMAQLRAHRADLQRL---VAARSA---EVRAREAELDRAQSVAHV        56476956       TM+TM+SHELIX+PAS+PAS+PAS+PAS+GGDEF+EAL                                                                       1039   Azoarcus sp. EbN1                                                    hypothetical protein ebA2702 [Azoarcus sp. EbN1]                                 
26987128    0.68: _1_[949..988]    NADAAMYRSKAKGRNR---VEAYTR---DLTAQASERIALEHELRR        26987128       TM+TM+PAS+PAS+PAS+PAS+GGDEF+SHELIX+EAL                                                                       1247   Pseudomonas putida KT2440                                            sensory box protein [Pseudomonas putida KT2440]                                  
56750182   0.016: _1_[122..161]    LQYAELRRQRDHLQAT---INEQTN---YLIDEIEALDKTQKELED        56750182       TM+TM+TM+SHELIX+PAS+GGDEF+EAL                                                                                712    Synechococcus elongatus PCC 6301                                     hypothetical protein syc0173_c [Synechococcus elongatus PCC 6301]                
15600635 2.5e-07: _1_[242..281]    LVERELRAARDALQRR---VEARTE---ELGATSQALSQSEARLAM        15600635       TM+TM+TM+TM+TM+SHELIX+PAS+PAS+GGDEF+EAL                                                                      951    Pseudomonas aeruginosa PAO1                                          hypothetical protein PA5442 [Pseudomonas aeruginosa PAO1]                        
54297266 0.00025: _1_[207..246]    FKISNLNSTKDILEKK---VVDRTK---ELEKILALTKSTLESTAD        54297266       TM+TM+TM+TM+TM+TM+SHELIX+PAS+GGDEF+EAL                                                                       792    Legionella pneumophila str. Paris                                    hypothetical protein lpp1311 [Legionella pneumophila str. Paris]                 
52841587 1.6e-05: _1_[207..246]    FKISNLNSTKDILEKK---VADRTK---ELEKILALTKSTLESTAD        52841587       TM+TM+TM+TM+TM+TM+SHELIX+PAS+GGDEF+EAL                                                                       792    Legionella pneumophila subsp. pneumophila str. Philadelphia 1        inner membrane protein PLUS sensory box protein LssE [Legionella pneumophila subsp. pneumophila str. Philadelphia 1]
54294243 0.00025: _1_[207..246]    FKISNLNSTKDILEKK---VVDRTK---ELEKILALTKSTLESTAD        54294243       TM+TM+TM+TM+TM+TM+SHELIX+PAS+GGDEF+EAL                                                                       792    Legionella pneumophila str. Lens                                     hypothetical protein lpl1308 [Legionella pneumophila str. Lens]                  
47527823 1.4e-07: _1_[326..365]    DLTETLKVFNNQLEQK---VFQRTA---DLIKKSNDLVKNQERFKS        47527823       TM+TM+TM+TM+TM+TM+TM+TM+TM+TM+SHELIX+PAS+TM+GGDEF+EAL                                                        912    Bacillus anthracis str. 'Ames Ancestor'                              sensory box/ggdef family protein [Bacillus anthracis str. 'Ames Ancestor']       
47529169 3.8e-08: _1_[323..362]    SLTKRLQAFNAQLEEK---VTLRTS---DLVHKSEALSQKQQKFKS        47529169       TM+TM+TM+TM+TM+TM+TM+TM+TM+TM+SHELIX+PAS+TM+GGDEF+EAL                                                        911    Bacillus anthracis str. 'Ames Ancestor'                              sensory box/ggdef family protein [Bacillus anthracis str. 'Ames Ancestor']       
47530863 4.6e-06: _1_[321..360]    LLLLAQMQFNSELEKQ---IELRTE---DLVEQKNELYHNQQMFKS        47530863       TM+TM+TM+TM+TM+TM+TM+TM+TM+TM+SHELIX+PAS+TM+GGDEF+EAL                                                        909    Bacillus anthracis str. 'Ames Ancestor'                              sensory box/ggdef family protein [Bacillus anthracis str. 'Ames Ancestor']       
47530915  0.0021: _1_[313..352]    ALIDTYEQLTTQLEGK---VEEGAS---ALSKSEQRYKSLFEDHPD        47530915       TM+TM+TM+TM+TM+TM+TM+TM+TM+TM+SHELIX+PAS+TM+GGDEF+EAL                                                        892    Bacillus anthracis str. 'Ames Ancestor'                              sensory box/ggdef family protein [Bacillus anthracis str. 'Ames Ancestor']       
30265372  0.0021: _1_[313..352]    ALIDTYEQLTTQLEGK---VEEGAS---ALSKSEQRYKSLFEDHPD        30265372       TM+TM+TM+TM+TM+TM+TM+TM+TM+TM+SHELIX+PAS+TM+GGDEF+EAL                                                        892    Bacillus anthracis str. Ames                                         sensory box/GGDEF family protein [Bacillus anthracis str. Ames]                  
49186595 3.8e-08: _1_[323..362]    SLTKRLQAFNAQLEEK---VTLRTS---DLVHKSEALSQKQQKFKS        49186595       TM+TM+TM+TM+TM+TM+TM+TM+TM+TM+SHELIX+PAS+TM+GGDEF+EAL                                                        911    Bacillus anthracis str. Sterne                                       sensory box/GGDEF family protein [Bacillus anthracis str. Sterne]                
49188137 4.6e-06: _1_[321..360]    LLLLAQMQFNSELEKQ---IELRTE---DLVEQKNELYHNQQMFKS        49188137       TM+TM+TM+TM+TM+TM+TM+TM+TM+TM+SHELIX+PAS+TM+GGDEF+EAL                                                        909    Bacillus anthracis str. Sterne                                       sensory box/GGDEF family protein [Bacillus anthracis str. Sterne]                
49188183  0.0021: _1_[313..352]    ALIDTYEQLTTQLEGK---VEEGAS---ALSKSEQRYKSLFEDHPD        49188183       TM+TM+TM+TM+TM+TM+TM+TM+TM+TM+SHELIX+PAS+TM+GGDEF+EAL                                                        892    Bacillus anthracis str. Sterne                                       sensory box/GGDEF family protein [Bacillus anthracis str. Sterne]                
49477748 3.6e-07: _1_[326..365]    DLTETLKVFNSQLEQK---VSQRTA---DLITKSNDLVINQERFKS        49477748       TM+TM+TM+TM+TM+TM+TM+TM+TM+TM+SHELIX+PAS+TM+GGDEF+EAL                                                        912    Bacillus thuringiensis serovar konkukian str. 97-27                  sensory box/GGDEF family protein [Bacillus thuringiensis serovar konkukian str. 97-27]
42782831   7e-08: _1_[323..362]    SLTKRLQVFNAQLEEK---VTLRTS---DLVHKSEALSQKQQKFKS        42782831       TM+TM+TM+TM+TM+TM+TM+TM+TM+TM+SHELIX+PAS+TM+GGDEF+EAL                                                        910    Bacillus cereus ATCC 10987                                           sensory box/GGDEF family protein [Bacillus cereus ATCC 10987]                    
49478974  0.0021: _1_[313..352]    ALIDTYEQLTTQLEGK---VEEGAS---ALSKSEQRYKSLFEDHPD        49478974       TM+TM+TM+TM+TM+TM+TM+TM+TM+TM+SHELIX+PAS+TM+GGDEF+EAL                                                        892    Bacillus thuringiensis serovar konkukian str. 97-27                  conserved hypothetical protein, PAS/PAC domain, EAL domain, GGDEF domain [Bacillus thuringiensis serovar konkukian str. 97-27]
49481140   3e-06: _1_[321..360]    ILLLAQMQFNSELEKQ---IELRTE---DLVEQKNELYHNQQMFKS        49481140       TM+TM+TM+TM+TM+TM+TM+TM+TM+TM+SHELIX+PAS+TM+GGDEF+EAL                                                        909    Bacillus thuringiensis serovar konkukian str. 97-27                  sensory box/GGDEF family protein [Bacillus thuringiensis serovar konkukian str. 97-27]
52140213  0.0021: _1_[313..352]    ALIDTYEQLTTQLEGK---VEEGAS---ALSKSEQRYKSLFEDHPD        52140213       TM+TM+TM+TM+TM+TM+TM+TM+TM+TM+SHELIX+PAS+TM+GGDEF+EAL                                                        892    Bacillus cereus E33L                                                 sensory box/GGDEF family protein [Bacillus cereus E33L]                          
52140259   3e-06: _1_[321..360]    ILLLAQMQFNSELEKQ---IELRTE---DLVEQKNELYHNQQMFKS        52140259       TM+TM+TM+TM+TM+TM+TM+TM+TM+TM+SHELIX+PAS+TM+GGDEF+EAL                                                        909    Bacillus cereus E33L                                                 sensory box/GGDEF family protein [Bacillus cereus E33L]                          
52141742 3.8e-08: _1_[323..362]    SLTKRLQAFNAQLEEK---VTLRTS---DLVHKSEALSQKQQKFKS        52141742       TM+TM+TM+TM+TM+TM+TM+TM+TM+TM+SHELIX+PAS+TM+GGDEF+EAL                                                        911    Bacillus cereus E33L                                                 sensory box/GGDEF family [Bacillus cereus E33L]                                  
52142967 1.4e-07: _1_[326..365]    DLTETLKVFNNQLEQK---VFQRTA---DLIKKSNDLVKNQERFKS        52142967       TM+TM+TM+TM+TM+TM+TM+TM+TM+TM+SHELIX+PAS+TM+GGDEF+EAL                                                        912    Bacillus cereus E33L                                                 sensory box/GGDEF family protein [Bacillus cereus E33L]                          
30265324 4.6e-06: _1_[321..360]    LLLLAQMQFNSELEKQ---IELRTE---DLVEQKNELYHNQQMFKS        30265324       TM+TM+TM+TM+TM+TM+TM+TM+TM+TM+SHELIX+PAS+TM+GGDEF+EAL                                                        909    Bacillus anthracis str. Ames                                         sensory box/GGDEF family protein [Bacillus anthracis str. Ames]                  
42784524 0.00069: _1_[313..352]    ALIDTYEQLTTQLEDK---VEEGAS---ALSKSEQRYKSLFEDHPD        42784524       TM+TM+TM+TM+TM+TM+TM+TM+TM+TM+SHELIX+PAS+TM+GGDEF+EAL                                                        892    Bacillus cereus ATCC 10987                                           sensory box/GGDEF family protein [Bacillus cereus ATCC 10987]                    
30263750 3.8e-08: _1_[323..362]    SLTKRLQAFNAQLEEK---VTLRTS---DLVHKSEALSQKQQKFKS        30263750       TM+TM+TM+TM+TM+TM+TM+TM+TM+TM+SHELIX+PAS+TM+GGDEF+EAL                                                        911    Bacillus anthracis str. Ames                                         sensory box/GGDEF family protein [Bacillus anthracis str. Ames]                  
30262524 1.4e-07: _1_[326..365]    DLTETLKVFNNQLEQK---VFQRTA---DLIKKSNDLVKNQERFKS        30262524       TM+TM+TM+TM+TM+TM+TM+TM+TM+TM+SHELIX+PAS+TM+GGDEF+EAL                                                        912    Bacillus anthracis str. Ames                                         sensory box/GGDEF family protein [Bacillus anthracis str. Ames]                  
49478361 3.8e-08: _1_[323..362]    SLTKRLQAFNAQLEEK---VTLRTS---DLVHKSEALSQKQQKFKS        49478361       TM+TM+TM+TM+TM+TM+TM+TM+TM+TM+SHELIX+PAS+TM+GGDEF+EAL                                                        911    Bacillus thuringiensis serovar konkukian str. 97-27                  sensory box/GGDEF family [Bacillus thuringiensis serovar konkukian str. 97-27]   
42784471   3e-06: _1_[321..360]    ILLLAQMQFNSELEKQ---IELRTE---DLVEQKNELYHNQQMFKS        42784471       TM+TM+TM+TM+TM+TM+TM+TM+TM+TM+SHELIX+PAS+TM+GGDEF+EAL                                                        909    Bacillus cereus ATCC 10987                                           sensory box/GGDEF family protein [Bacillus cereus ATCC 10987]                    
30023334 2.3e-06: _1_[321..360]    ILLIAQMQFNSELEKQ---IKLRTE---DLVEQKNELYHNQQMFKS        30023334       TM+TM+TM+TM+TM+TM+TM+TM+TM+TM+SHELIX+PAS+TM+GGDEF+EAL                                                        909    Bacillus cereus ATCC 14579                                           Sensory box/GGDEF family protein [Bacillus cereus ATCC 14579]                    
30021841 3.8e-08: _1_[323..362]    SLTKRLQAFNAQLEEK---VTLRTS---DLVHKSEALSQKQQKFKS        30021841       TM+TM+TM+TM+TM+TM+TM+TM+TM+TM+SHELIX+PAS+TM+GGDEF+EAL                                                        909    Bacillus cereus ATCC 14579                                           Sensory box/GGDEF family protein [Bacillus cereus ATCC 14579]                    
49185366 1.4e-07: _1_[326..365]    DLTETLKVFNNQLEQK---VFQRTA---DLIKKSNDLVKNQERFKS        49185366       TM+TM+TM+TM+TM+TM+TM+TM+TM+TM+SHELIX+PAS+TM+GGDEF+EAL                                                        912    Bacillus anthracis str. Sterne                                       sensory box/GGDEF family protein [Bacillus anthracis str. Sterne]                
# 33            :                                                                        # 33
13474865 2.1e-09: _1_[345..384]    QRQEVLVLLNAELEHR---VELRTA---ELQSSNAALAGEIAEREN        13474865       TM+MCP-N+CACHE+TM+SHELIX+HISKIN                                                                              623    Mesorhizobium loti MAFF303099                                        dicarboxylate sensor protein [Mesorhizobium loti MAFF303099]                     
13476030 4.4e-09: _1_[324..363]    LRQEALARMNAELESR---VSTRTA---ELTRSNIALAGEIAEREN        13476030       TM+MCP-N+CACHE+TM+SHELIX+HISKIN                                                                              601    Mesorhizobium loti MAFF303099                                        two-component C4-dicarboxylate transport system, sensor protein [Mesorhizobium loti MAFF303099]
15596533 5.2e-11: _1_[348..387]    AAREALQRANGELEVK---IAERTA---DLQASNARLTAEIHERQQ        15596533       TM+MCP-N+CACHE+TM+SHELIX+HISKIN                                                                              633    Pseudomonas aeruginosa PAO1                                          probable two-component sensor [Pseudomonas aeruginosa PAO1]                      
15600358 1.1e-08: _1_[319..358]    DRIALEAEAKRQLEER---VLERTR---ELENANAQLQQEVHEREQ        15600358       TM+MCP-N+CACHE+TM+SHELIX+HISKIN                                                                              612    Pseudomonas aeruginosa PAO1                                          probable two-component sensor [Pseudomonas aeruginosa PAO1]                      
15600705   1e-06: _1_[324..363]    RIARLRQRSREELERL---VEQRTA---DLRTAQDGLVQAAKLAAL        15600705       MCP-N+CACHE+SHELIX+HISKIN                                                                                    588    Pseudomonas aeruginosa PAO1                                          probable two-component sensor [Pseudomonas aeruginosa PAO1]                      
15641927  0.0014: _1_[321..360]    QIEQLQQETKQKLEFE---VMERTA---KLHAEIAERIKTEHALRQ        15641927       TM+MCP-N+TM+CACHE+TM+SHELIX+HISKIN                                                                           597    Vibrio cholerae O1 biovar eltor str. N16961                          C4-dicarboxylate transport sensor protein [Vibrio cholerae O1 biovar eltor str. N16961]
15887468 1.4e-09: _1_[369..408]    ERLTHQAEAQAELERR---VEERTA---DLARVNQEIKHEIAERRQ        15887468       TM+MCP-N+CACHE+TM+SHELIX+HISKIN                                                                              656    Agrobacterium tumefaciens str. C58                                   hypothetical protein AGR_C_174 [Agrobacterium tumefaciens str. C58]              
15891636 2.8e-07: _1_[358..397]    REKEEEQRARAELEMK---VQERTR---DLTKTRDHLQAEITLHEK        15891636       TM+MCP-N+CACHE+SHELIX+HISKIN                                                                                 633    Agrobacterium tumefaciens str. C58                                   hypothetical protein AGR_L_3043 [Agrobacterium tumefaciens str. C58]             
16264614 1.5e-09: _1_[320..359]    ERILMQEQAQEELERR---VEERTA---DLARVNSQIEAEIAERRL        16264614       TM+MCP-N+CACHE+TM+SHELIX+HISKIN                                                                              606    Sinorhizobium meliloti 1021                                          putative two-component sensor histidine kinase protein [Sinorhizobium meliloti 1021]
16265272 2.8e-06: _1_[346..385]    MRSAEERLARNALEAS---VEERTR---DLRMARDRLETEIADHRQ        16265272       TM+MCP-N+CACHE+SHELIX+HISKIN                                                                                 621    Sinorhizobium meliloti 1021                                          C4-dicarboxylate transport sensor protein [Sinorhizobium meliloti 1021]          
17545050   5e-11: _1_[373..412]    RGRRLLEAAYDQLERR---VEDRTA---DLMATNEQLQHEIVDRTR        17545050       TM+MCP-N+CACHE+TM+SHELIX+HISKIN                                                                              665    Ralstonia solanacearum GMI1000                                       PROBABLE C4-DICARBOXYLATE TRANSPORT SENSOR KINASE TRANSCRIPTION REGULATOR PROTEIN [Ralstonia solanacearum GMI1000]
17548229 2.3e-09: _1_[346..385]    RARAALREVQRDLEAR---IAQRTA---ELTSANAALASKVEALDT        17548229       TM+MCP-N+CACHE+TM+SHELIX+HISKIN                                                                              634    Ralstonia solanacearum GMI1000                                       PROBABLE C4-DICARBOXYLATE TRANSPORT SENSOR KINASE TRANSCRIPTION REGULATOR PROTEIN [Ralstonia solanacearum GMI1000]
17934032 1.4e-09: _1_[321..360]    ERLTHQAEAQAELERR---VEERTA---DLARVNQEIKHEIAERRQ        17934032       TM+MCP-N+CACHE+TM+SHELIX+HISKIN                                                                              608    Agrobacterium tumefaciens str. C58                                   two component sensor kinase [Agrobacterium tumefaciens str. C58]                 
17937008 2.8e-07: _1_[338..377]    REKEEEQRARAELEMK---VQERTR---DLTKTRDHLQAEITLHEK        17937008       TM+MCP-N+CACHE+SHELIX+HISKIN                                                                                 613    Agrobacterium tumefaciens str. C58                                   two component sensor kinase for C4-dicarboxylate transport [Agrobacterium tumefaciens str. C58]
26250853 6.7e-11: _1_[325..364]    HAQQLLTLANETLEKQ---VKERTS---ALELINQKLIQEIKERSQ        26250853       TM+MCP-N+CACHE+TM+SHELIX+HISKIN                                                                              606    Escherichia coli CFT073                                              Putative transport sensor protein [Escherichia coli CFT073]                      
26987006 5.8e-08: _1_[311..350]    DRIDFEARGRQELEKR---VAERTA---DLEGLNTRLKNAVLEREN        26987006       TM+MCP-N+CACHE+TM+SHELIX+HISKIN                                                                              604    Pseudomonas putida KT2440                                            sensor histidine kinase [Pseudomonas putida KT2440]                              
26987803 9.3e-12: _1_[350..389]    AAREALEEANSQLERR---IAERTA---DLRASNERLKGQIRERRH        26987803       TM+MCP-N+CACHE+TM+SHELIX+HISKIN                                                                              635    Pseudomonas putida KT2440                                            sensor histidine kinase [Pseudomonas putida KT2440]                              
26988136 2.4e-06: _1_[324..363]    RLARLRQRSRQELERQ---VEERTR---ELRTAQEGLVQSAKLAAL        26988136       TM+MCP-N+CACHE+TM+SHELIX+HISKIN                                                                              585    Pseudomonas putida KT2440                                            C4-dicarboxylate transport sensor protein [Pseudomonas putida KT2440]            
27363514   5e-05: _1_[320..359]    QIERVQQEAKQKLEFL---VMERTA---ELQAEIAQRTKTEQALRL        27363514       TM+MCP-N+CACHE+TM+SHELIX+HISKIN                                                                              595    Vibrio vulnificus CMCP6                                              Signal transduction histidine kinase regulating C4-dicarboxylate transport system [Vibrio vulnificus CMCP6]
27367507 2.8e-09: _1_[299..338]    NNKRQLALLNKQLESK---VKQRTE---VLSESNQRLQQTIIQYEK        27367507       MCP-N+CACHE+TM+SHELIX+HISKIN                                                                                 588    Vibrio vulnificus CMCP6                                              Signal transduction histidine kinase regulating C4-dicarboxylate transport system [Vibrio vulnificus CMCP6]
27378841 3.6e-10: _1_[363..402]    ASQAALQAANDMLETR---VQERTA---ELRAAQDELVHAGKLAAL        27378841       TM+MCP-N+CACHE+TM+SHELIX+HISKIN                                                                              631    Bradyrhizobium japonicum USDA 110                                    two-component hybrid sensor and regulator [Bradyrhizobium japonicum USDA 110]    
28275254 7.9e-10: _1_[333..372]    RNLQRMQLAQSLLEQR---VEERTY---DLQQANDRLKDTQDELIQ        28275254       TM+MCP-N+CACHE+TM+SHELIX+HISKIN                                                                              555    Shewanella oneidensis MR-1                                           hypothetical two-component sensor histidine kinase protein [Shewanella oneidensis MR-1]
28871317 1.4e-09: _1_[350..389]    AAREALQAANDELERK---ITERTE---HLRASNERLKAQIRERRL        28871317       TM+MCP-N+CACHE+SHELIX+HISKIN                                                                                 643    Pseudomonas syringae pv. tomato str. DC3000                          sensor histidine kinase [Pseudomonas syringae pv. tomato str. DC3000]            
28871428 2.9e-06: _1_[325..364]    RLARLRERNRDELERL---VQERTR---DLQTAQDGLVQSAKLAAL        28871428       TM+MCP-N+CACHE+TM+SHELIX+HISKIN                                                                              585    Pseudomonas syringae pv. tomato str. DC3000                          sensor histidine kinase [Pseudomonas syringae pv. tomato str. DC3000]            
28872506 3.5e-08: _1_[309..348]    DRIAFEAKARRELEMR---VIERTS---DLEGLNSRLRQEVLEREQ        28872506       MCP-N+CACHE+TM+SHELIX+HISKIN                                                                                 602    Pseudomonas syringae pv. tomato str. DC3000                          sensor histidine kinase [Pseudomonas syringae pv. tomato str. DC3000]            
28897689 2.5e-05: _1_[320..359]    QLERIQQEANQKLEFQ---VMARTA---ELQAEIAQRTETEQTLRL        28897689       TM+MCP-N+TM+CACHE+TM+SHELIX+HISKIN                                                                           600    Vibrio parahaemolyticus RIMD 2210633                                 C4-dicarboxylate transport sensor protein [Vibrio parahaemolyticus RIMD 2210633] 
28901586 1.6e-08: _1_[317..356]    VNKARLANLNVSLEEK---VMQRTV---VLSEANHKLQQTVRQYEQ        28901586       TM+MCP-N+CACHE+TM+SHELIX+HISKIN                                                                              616    Vibrio parahaemolyticus RIMD 2210633                                 putative C4-dicarboxylate transport sensor protein [Vibrio parahaemolyticus RIMD 2210633]
37677309 2.8e-09: _1_[314..353]    NNKRQLALLNKQLESK---VKQRTE---VLSESNQRLQQTIIQYEK        37677309       TM+MCP-N+CACHE+TM+SHELIX+HISKIN                                                                              603    Vibrio vulnificus YJ016                                              signal transduction histidine kinase [Vibrio vulnificus YJ016]                   
37679285   5e-05: _1_[330..369]    QIERVQQEAKQKLEFL---VMERTA---ELQAEIAQRTKTEQALRL        37679285       TM+MCP-N+CACHE+TM+SHELIX+HISKIN                                                                              605    Vibrio vulnificus YJ016                                              signal transduction histidine kinase regulating C4-dicarboxylate transport system [Vibrio vulnificus YJ016]
53718065 6.7e-10: _1_[373..412]    RSRVLLQSAYAELNRR---VEERTA---DLSQANARLKKEVGERIR        53718065       TM+MCP-N+CACHE+SHELIX+HISKIN                                                                                 677    Burkholderia pseudomallei K96243                                     C4-dicarboxylate transport sensor protein [Burkholderia pseudomallei K96243]     
53721102 4.4e-10: _1_[348..387]    RARTALLAAQRDLERR---IAERTA---ELTAANAALEEKVDALDA        53721102       TM+MCP-N+CACHE+TM+SHELIX+HISKIN                                                                              643    Burkholderia pseudomallei K96243                                     putative C4-dicarboxylate transport sensor kinase [Burkholderia pseudomallei K96243]
54303668 2.6e-07: _1_[337..376]    LTKKELAQLNDKLEQL---VVERTH---NLLETNQQLRDTIEQYER        54303668       TM+MCP-N+CACHE+TM+SHELIX+HISKIN                                                                              619    Photobacterium profundum SS9                                         hypothetical Signal transduction histidine kinase regulating C4-dicarboxylate transport system [Photobacterium profundum SS9]
56697472 1.8e-09: _1_[295..334]    ERRRTLSEANQVLESR---VAQRTR---ALTAANSALRREVAERQE        56697472       TM+MCP-N+TM+SHELIX+HISKIN                                                                                    578    Silicibacter pomeroyi DSS-3                                          C4-dicarboxylate transport sensor protein [Silicibacter pomeroyi DSS-3]          
# 32            :                                                                        # 32
58582187    0.65: _1_[109..148]    LALFHNIEGQQDYERE---LQQRHA---ELRQAYLRLNGAQDKLLQ        58582187       SHELIX+HISKIN                                                                                                408    Xanthomonas oryzae pv. oryzae KACC10331                              histidine kinase [Xanthomonas oryzae pv. oryzae KACC10331]                       
24373703    0.03: _1_[10..49]      VAYLREKKARQEVEQL---LEDSTR---QLYEKNVLLQQQIDLIKQ        24373703       SHELIX+HISKIN                                                                                                311    Shewanella oneidensis MR-1                                           sensor histidine kinase [Shewanella oneidensis MR-1]                             
21242735    0.65: _1_[109..148]    LALFHNIEGQQDYERE---LQQRHA---ELRQAYLRLNGAQDKLLQ        21242735       SHELIX+HISKIN                                                                                                408    Xanthomonas axonopodis pv. citri str. 306                            histidine kinase [Xanthomonas axonopodis pv. citri str. 306]                     
15598078 7.2e-05: _1_[60..99]      RGLGTVLDLLKAMEAR---IAQRNR---VLRKALVDLKESQAQLVQ        15598078       SHELIX+HISKIN                                                                                                371    Pseudomonas aeruginosa PAO1                                          probable two-component sensor [Pseudomonas aeruginosa PAO1]                      
53722667 4.8e-06: _1_[43..82]      GSDFSLFQMAVTLEDQ---VRHRTR---ELEAALHENQKIMHALQR        53722667       SHELIX+HISKIN                                                                                                389    Burkholderia pseudomallei K96243                                     probable two-component sensor kinase [Burkholderia pseudomallei K96243]          
21231405    0.65: _1_[109..148]    LALFHNIEGQQDYERE---LQQRHA---ELRQAYLRLNGAQDKLLQ        21231405       SHELIX+HISKIN                                                                                                408    Xanthomonas campestris pv. campestris str. ATCC 33913                histidine kinase [Xanthomonas campestris pv. campestris str. ATCC 33913]         
53716381 4.8e-06: _1_[12..51]      GSDFSLFQMAVTLEDQ---VRHRTR---ELEAALHENQKIMHALQR        53716381       SHELIX+HISKIN                                                                                                358    Burkholderia mallei ATCC 23344                                       sensory box sensor histidine kinase [Burkholderia mallei ATCC 23344]             
39997660 3.1e-08: _1_[7..46]       TGEEELRCRVQELEHR---VTELTE---ALDASNRELQTFSYSVSH        39997660       SHELIX+HISKIN                                                                                                255    Geobacter sulfurreducens PCA                                         sensor histidine kinase [Geobacter sulfurreducens PCA]                           
34498900 2.4e-05: _1_[16..55]      AVSGQLIDAYRQLEVQ---VNVLNA---QLDEANSQLRKQRDENAE        34498900       SHELIX+PAS+HISKIN                                                                                            381    Chromobacterium violaceum ATCC 12472                                 probable two-component sensor [Chromobacterium violaceum ATCC 12472]             
30250022 5.3e-06: _1_[25..64]      QASSQLSGIYRELQQQ---VFRLTE---ELALANGELQRELAAKEA        30250022       SHELIX+PAS+HISKIN                                                                                            411    Nitrosomonas europaea ATCC 19718                                     putative two-component sensor [Nitrosomonas europaea ATCC 19718]                 
26991061   0.014: _1_[36..75]      QVSSQLSQSYSLLEAR---VSELKG---ELAVVSAQRIDELSEKER        26991061       SHELIX+PAS+HISKIN                                                                                            405    Pseudomonas putida KT2440                                            sensory box histidine kinase FleS [Pseudomonas putida KT2440]                    
15596295  0.0083: _1_[33..72]      QMSSQLSESYSLLEER---VTELKG---QLALVSAQRMEELAEKER        15596295       SHELIX+PAS+HISKIN                                                                                            402    Pseudomonas aeruginosa PAO1                                          two-component sensor [Pseudomonas aeruginosa PAO1]                               
28870284 1.6e-11: _1_[140..179]    NAEEALRELNESLELR---VAERTR---ELAKANELLQVEIAERAQ        28870284       PAS+SHELIX+HISKIN                                                                                            423    Pseudomonas syringae pv. tomato str. DC3000                          sensory box histidine kinase [Pseudomonas syringae pv. tomato str. DC3000]       
32473302 8.8e-06: _1_[165..204]    AAEAELKQLNATLDQR---VKAQAA---ELQQAQLELVEKEKFATL        32473302       PAS+SHELIX+HISKIN                                                                                            418    Rhodopirellula baltica SH 1                                          sensor protein fixL [Rhodopirellula baltica SH 1]                                
32472620 0.00016: _1_[140..179]    QMREQLREQNAELEHL---VQMRTA---KIAEL-EKHRLKMEKLAA        32472620       PAS+SHELIX+HISKIN                                                                                            410    Rhodopirellula baltica SH 1                                          sensor protein atoS [Rhodopirellula baltica SH 1]                                
17229615 1.5e-09: _1_[142..181]    HAEGLLKQTNEELEAR---VEQRNI---ELIQANHDLKVTLDKLKY        17229615       PAS+SHELIX+HISKIN                                                                                            460    Nostoc sp. PCC 7120                                                  two-component sensor histidine kinase [Nostoc sp. PCC 7120]                      
26989664   1e-07: _1_[197..236]    EMTQRASLKMHQLEQR---VERRTQ---QLTQASEALQQEIEERKQ        26989664       REC+SHELIX+HISKIN                                                                                            505    Pseudomonas putida KT2440                                            sensor histidine kinase/response regulator [Pseudomonas putida KT2440]           
17232372 2.1e-10: _1_[123..162]    SSHLQLRSLNKNLEKR---VTERTA---ELNQALKDLQDYQLQLVQ        17232372       REC+SHELIX+HISKIN                                                                                            443    Nostoc sp. PCC 7120                                                  two-component hybrid sensor and regulator [Nostoc sp. PCC 7120]                  
27378097 1.6e-09: _1_[123..162]    LAVRRLHLENAELALR---VRERTL---ELEAANRELEAFAYSVAH        27378097       REC+SHELIX+HISKIN                                                                                            371    Bradyrhizobium japonicum USDA 110                                    two-component hybrid sensor and regulator [Bradyrhizobium japonicum USDA 110]    
17232800 5.5e-11: _1_[124..163]    KLHLKISHLTRTLEQK---VQERTA---ELTQSIQQLKQTQIQLIQ        17232800       REC+SHELIX+HISKIN                                                                                            437    Nostoc sp. PCC 7120                                                  two-component hybrid sensor and regulator [Nostoc sp. PCC 7120]                  
17228672 5.7e-07: _1_[131..170]    FLTKELEIKNQELDKL---VEERTQ---TLSLTLDKLQQSQLQLVQ        17228672       REC+SHELIX+HISKIN                                                                                            440    Nostoc sp. PCC 7120                                                  two-component hybrid sensor and regulator [Nostoc sp. PCC 7120]                  
24214446    0.46: _1_[127..162]    LQDAALHELNESL-RQ------KNL---ELEYSNKELDAFTYSVSH        24214446       REC+SHELIX+HISKIN                                                                                            371    Leptospira interrogans serovar Lai str. 56601                        two-component hybrid sensor and regulator [Leptospira interrogans serovar Lai str. 56601]
15597767   0.021: _1_[156..195]    NVTAHLQLQMSRMEAA---IQEITR---EVGQAKEALEKEIGERKE        15597767       REC+SHELIX+HISKIN                                                                                            470    Pseudomonas aeruginosa PAO1                                          probable two-component sensor [Pseudomonas aeruginosa PAO1]                      
17230651 3.3e-09: _1_[129..167]    QALETLRTMNTTLENR---VEERTA---ELVKA-QEINQFKSEFVS        17230651       REC+SHELIX+HISKIN                                                                                            385    Nostoc sp. PCC 7120                                                  two-component hybrid sensor and regulator [Nostoc sp. PCC 7120]                  
39996221 4.8e-07: _1_[168..207]    LLLAEIEAWNQELERR---VQQKSE---ALQQAQAEIVQSEKLASL        39996221       REC+SHELIX+HISKIN                                                                                            420    Geobacter sulfurreducens PCA                                         sensor histidine kinase/response regulator [Geobacter sulfurreducens PCA]        
45657906    0.46: _1_[127..162]    LQDAALHELNESL-RQ------KNL---ELEYSNKELDAFTYSVSH        45657906       REC+SHELIX+HISKIN                                                                                            371    Leptospira interrogans serovar Copenhageni str. Fiocruz L1-130       histidine kinase response regulator hybrid protein [Leptospira interrogans serovar Copenhageni str. Fiocruz L1-130]
17228133 2.7e-05: _1_[138..177]    STHLQLSQLTQNLAAQ---VAQKAA---ELEASQLQLIQNEKMSAL        17228133       REC+SHELIX+HISKIN                                                                                            438    Nostoc sp. PCC 7120                                                  two-component hybrid sensor and regulator [Nostoc sp. PCC 7120]                  
10957450 5.6e-09: _1_[145..184]    FAEAELRQLNTQLEEK---VARRTV---ELQERSAALEAQSAELAR        10957450       REC+SHELIX+HISKIN                                                                                            416    Deinococcus radiodurans R1                                           sensor histidine kinase/response regulator [Deinococcus radiodurans R1]          
39996351 1.3e-09: _1_[90..129]     EAETRLRRYQSNLESL---VEDRTA---RLTEAIEELEREIVERKR        39996351       TM+TM+SHELIX+PAS+PAS+HISKIN                                                                                  524    Geobacter sulfurreducens PCA                                         sensory box histidine kinase [Geobacter sulfurreducens PCA]                      
15678472    0.64: _1_[122..161]    ERIEKVRSLRE-LNLE---LKKQAE---KLEDANKELEAFAYSVSH        15678472       TM+TM+TM+SHELIX+HISKIN                                                                                       373    Methanothermobacter thermautotrophicus str. Delta H                  sensory transduction histidine kinase [Methanothermobacter thermautotrophicus str. Delta H]
17232208 7.2e-07: _1_[144..183]    TAEAALLQIKADLENR---VEERTN---QLQQSMLASLATAAKAKD        17232208       TM+TM+TM+SHELIX+HISKIN                                                                                       468    Nostoc sp. PCC 7120                                                  two-component sensor histidine kinase [Nostoc sp. PCC 7120]                      
39997763 7.7e-13: _1_[96..135]     QAEQETRAINENLELL---IAERTA---ALEASNRELESFCYSVSH        39997763       TM+TM+TM+SHELIX+HISKIN                                                                                       343    Geobacter sulfurreducens PCA                                         sensor histidine kinase [Geobacter sulfurreducens PCA]                           
# 28            :                                                                        # 28
27379387 1.8e-06: _1_[156..195]    STLLETTAAKEQLEAA---VAERTE---HLVTAHDELRLSVNVLQS        27379387       chase3+TM+SHELIX+PAS+HISKIN+REC                                                                              698    Bradyrhizobium japonicum USDA 110                                    two-component hybrid sensor and regulator [Bradyrhizobium japonicum USDA 110]    
21242421 1.5e-06: _1_[129..167]    RARQKQFVIRDQLAQL---AESRTA----LAQSEAELRRVTDALPV        21242421       SHELIX+PAS+REC+SHELIX+HISKIN+REC                                                                          690    Xanthomonas axonopodis pv. citri str. 306                            histidine kinase-response regulator hybrid protein [Xanthomonas axonopodis pv. citri str. 306]
26990362 1.2e-07: _1_[323..362]    EAMAALEALNTTLEHR---VEERTT---QLLHTEAVLRQAQKLEAI        26990362       PAS+GAF+GAF+SHELIX+HISKIN+REC                                                                                727    Pseudomonas putida KT2440                                            sensor histidine kinase/response regulator [Pseudomonas putida KT2440]           
21231108 7.2e-08: _1_[281..320]    VAMESIQQQATLLEAK---VAERTA---ELEQQMQARESSEAALRQ        21231108       PAS+SHELIX+HISKIN+REC                                                                                        700    Xanthomonas campestris pv. campestris str. ATCC 33913                histidine kinase/response regulator hybrid protein [Xanthomonas campestris pv. campestris str. ATCC 33913]
53716772 0.00018: _1_[176..215]    ETNAELRRLTETLEQR---VADAIT---ERASIEEQLRQAQKMEAI        53716772       PAS+SHELIX+HISKIN+REC                                                                                        611    Burkholderia mallei ATCC 23344                                       sensory box histidine kinase/response regulator [Burkholderia mallei ATCC 23344] 
53722961 0.00018: _1_[176..215]    ETNAELRRLTETLEQR---VADAIT---ERASIEEQLRQAQKMEAI        53722961       PAS+SHELIX+HISKIN+REC                                                                                        611    Burkholderia pseudomallei K96243                                     putative sensor kinase/response regulator fusion protein [Burkholderia pseudomallei K96243]
26990258  0.0042: _1_[153..192]    RAEQALQRLNEELEQR---VNAALA---ERRLFAELVDHSVVNVHV        26990258       PAS+SHELIX+PAS+HISKIN+REC                                                                                    688    Pseudomonas putida KT2440                                            sensory box histidine kinase/response regulator [Pseudomonas putida KT2440]      
28870744    0.73: _1_[109..148]    RAERALQQLNETLEAR---IVEAVA---ERNVLANVVNGTSAFIHV        28870744       PAS+SHELIX+PAS+HISKIN+REC                                                                                    646    Pseudomonas syringae pv. tomato str. DC3000                          sensory box histidine kinase/response regulator [Pseudomonas syringae pv. tomato str. DC3000]
28869074 3.3e-05: _1_[273..312]    KAEEAVRELNQTLEQR---IEQAVF---EREQIEDALRHSQKMDAV        28869074       PAS+PAS+SHELIX+HISKIN+REC                                                                                    691    Pseudomonas syringae pv. tomato str. DC3000                          sensory box histidine kinase/response regulator [Pseudomonas syringae pv. tomato str. DC3000]
39936279 1.6e-07: _1_[276..315]    RAIAELRALNETLEQR---VAEARA---ELLRSAEQLRQSQKMEAV        39936279       PAS+PAS+SHELIX+HISKIN+REC                                                                                    686    Rhodopseudomonas palustris CGA009                                    sensor histidine kinase with multiple PAS and a response regulator receiver domain [Rhodopseudomonas palustris CGA009]
16127455 9.1e-06: _1_[321..360]    RAEADLRGLNETLESR---IAQALA---ERERTEEALRQAQKMEAV        16127455       PAS+PAS+SHELIX+HISKIN+REC                                                                                    731    Caulobacter crescentus CB15                                          sensory box histidine kinase/response regulator [Caulobacter crescentus CB15]    
17937743 4.4e-06: _1_[263..302]    RAEEQLRQLNEGLEAR---VEMEMA---ERRQTEKALQQAQKMESI        17937743       PAS+PAS+SHELIX+HISKIN+REC+REC                                                                                822    Agrobacterium tumefaciens str. C58                                   two component sensor kinase/response regulator hybrid [Agrobacterium tumefaciens str. C58]
39934056 7.2e-09: _1_[264..303]    QAEIDLRNVNETLERL---IAQRTA---QLHSSESQLRAILETTNQ        39934056       PAS+PAS+SHELIX+PAS+HISKIN+REC                                                                                795    Rhodopseudomonas palustris CGA009                                    sensor histidine kinase with multiple PAS and a response regulator receiver domain [Rhodopseudomonas palustris CGA009]
28871430 2.6e-05: _1_[270..308]    NAEEALRTLNETLEQR---VVERTQ---DRDRI-WRLSTDLMLVAQ        28871430       PAS+PAS+SHELIX+PAS+HISKIN+REC                                                                                793    Pseudomonas syringae pv. tomato str. DC3000                          sensory box histidine kinase/response regulator [Pseudomonas syringae pv. tomato str. DC3000]
27380380 3.8e-09: _1_[294..333]    QAEIELRRLNETLEER---IVERTA---ELESNEARLRAILETSNQ        27380380       PAS+PAS+SHELIX+PAS+HISKIN+REC                                                                                825    Bradyrhizobium japonicum USDA 110                                    two-component hybrid sensor and regulator [Bradyrhizobium japonicum USDA 110]    
21242418 1.4e-07: _2_[262..301]    VAEEQLRQLTETLEER---VRERSA---ALLLAEEKLRQSQKMEAV        21242418       PAS+PAS+SHELIX+PAS+SHELIX+HISKIN+REC                                                                      676    Xanthomonas axonopodis pv. citri str. 306                            histidine kinase-response regulator hybrid protein [Xanthomonas axonopodis pv. citri str. 306]
28870851 2.3e-11: _1_[417..456]    RATAALRTLNETLEQR---VAARTA---ELMQAEEKLRQSQKMEAV        28870851       PAS+PAS+PAS+SHELIX+HISKIN+REC                                                                                827    Pseudomonas syringae pv. tomato str. DC3000                          sensory box histidine kinase/response regulator [Pseudomonas syringae pv. tomato str. DC3000]
15890924 4.4e-06: _1_[432..471]    RAEEQLRQLNEGLEAR---VEMEMA---ERRQTEKALQQAQKMESI        15890924       PAS+PAS+PAS+SHELIX+HISKIN+REC+REC                                                                            991    Agrobacterium tumefaciens str. C58                                   hypothetical protein AGR_L_1617 [Agrobacterium tumefaciens str. C58]             
21231863 0.00026: _1_[468..507]    RVEQQLRDLNETLEQR---VVEALA---ERRLFADFVDSTDAAVLA        21231863       PAS+PAS+PAS+SHELIX+PAS+HISKIN+REC                                                                            1002   Xanthomonas campestris pv. campestris str. ATCC 33913                histidine kinase/response regulator hybrid protein [Xanthomonas campestris pv. campestris str. ATCC 33913]
21231105 5.7e-10: _1_[551..590]    QAEAQLRQLTDTLEER---VRERTA---ELLLAEEKLRQSQKMEAV        21231105       PAS+PAS+PAS+PAS+SHELIX+HISKIN+REC                                                                            968    Xanthomonas campestris pv. campestris str. ATCC 33913                histidine kinase/response regulator hybrid protein [Xanthomonas campestris pv. campestris str. ATCC 33913]
27377289 2.5e-11: _1_[142..181]    AYLEAQRGASAELERL---VAERTA---DLERANSQLREEIAGRER        27377289       REC+SHELIX+HISKIN+REC                                                                                        560    Bradyrhizobium japonicum USDA 110                                    two-component hybrid sensor and regulator [Bradyrhizobium japonicum USDA 110]    
17939091 1.6e-09: _1_[208..247]    QSEEQQRHLNETLEER---VAERTR---ELEEAHRLVLAEVSQRER        17939091       REC+PAS+SHELIX+HISKIN+REC                                                                                    620    Agrobacterium tumefaciens str. C58                                   two component sensor kinase/response regulator hybrid [Agrobacterium tumefaciens str. C58]
16119738 1.6e-09: _1_[287..326]    QSEEQQRHLNETLEER---VAERTR---ELEEAHRLVLAEVSQRER        16119738       REC+PAS+SHELIX+HISKIN+REC                                                                                    699    Agrobacterium tumefaciens str. C58                                   hypothetical protein AGR_pAT_755 [Agrobacterium tumefaciens str. C58]            
26989078 5.2e-06: _2_[393..431]    EAALSLADANARLREQ---INERE----RIEAALQQMQRLEAVGQL        26989078       REC+PAS+SHELIX+PAS+SHELIX+HISKIN+REC                                                                      795    Pseudomonas putida KT2440                                            sensory box histidine kinase/response regulator [Pseudomonas putida KT2440]      
28869904   4e-10: _1_[267..306]    QAESQLRRLNETLEHQ---VEERTS---QLRQKEEILRQSQKMEAV        28869904       REC+PAS+REC+SHELIX+HISKIN+REC                                                                                676    Pseudomonas syringae pv. tomato str. DC3000                          sensory box histidine kinase/response regulator [Pseudomonas syringae pv. tomato str. DC3000]
39997909  0.0046: _1_[224..263]    VILAGLFFFNTRLRQR---VRQSTE---ALAESEENYRHFTTLTSD        39997909       TM+SHELIX+PAS+PAS+HISKIN+REC                                                                                 882    Geobacter sulfurreducens PCA                                         sensory box histidine kinase/response regulator [Geobacter sulfurreducens PCA]   
51245370  0.0096: _1_[276..315]    GLVFFFVGAALLLKKT---VRSRTR---ELAESEASLLEAQDIARI        51245370       TM+REC+TM+SHELIX+PAS+PAS+HISKIN+REC                                                                          930    Desulfotalea psychrophila LSv54                                      similar to two-component system sensory/regulatory protein (Ntr family) [Desulfotalea psychrophila LSv54]
16127106  0.0025: _1_[393..432]    QAEAKLTGLNELLEER---VTAALA---ERDAAQAALLQSQKLEAI        16127106       TM+TM+TM+PAS+PAS+SHELIX+HISKIN+REC                                                                           794    Caulobacter crescentus CB15                                          sensory box histidine kinase/response regulator [Caulobacter crescentus CB15]    
# 28            :                                                                        # 28
15597768 5.2e-10: _1_[149..188]    EQNDRLQQLNATLEKR---VQARTA---ELQQTADMLDLAYEELKR        15597768       REC+SHELIX+HDGYP                                                                                             447    Pseudomonas aeruginosa PAO1                                          probable two-component response regulator [Pseudomonas aeruginosa PAO1]          
15599975 3.2e-06: _1_[132..171]    ANADFLRDKSEYLELE---VRRRTR---QLQQLQDAVIEALATLGD        15599975       REC+SHELIX+HDGYP                                                                                             393    Pseudomonas aeruginosa PAO1                                          probable two-component response regulator [Pseudomonas aeruginosa PAO1]          
15641100 9.3e-11: _1_[158..197]    ENNHKLAQFNQELEAK---VQERTV---ALQESNKRLESLLASRNK        15641100       REC+SHELIX+HDGYP                                                                                             447    Vibrio cholerae O1 biovar eltor str. N16961                          response regulator [Vibrio cholerae O1 biovar eltor str. N16961]                 
15641360   0.013: _1_[191..230]    RSKDILLNQNDYLETE---VLRRSG---ELDRMQDAVVFALASLAE        15641360       REC+SHELIX+HDGYP                                                                                             441    Vibrio cholerae O1 biovar eltor str. N16961                          response regulator [Vibrio cholerae O1 biovar eltor str. N16961]                 
16330586  0.0019: _1_[132..171]    AAADFLLDKNAFLEQE---VARRTQ---EMMAIQDVTIQVMASLAE        16330586       REC+SHELIX+HDGYP                                                                                             368    Synechocystis sp. PCC 6803                                           regulatory components of sensory transduction system [Synechocystis sp. PCC 6803]
24372449 9.1e-07: _1_[118..157]    KTHLALYDQKRLLEQQ---VKERTQ---ELEETRFEIIRRLGRAAE        24372449       REC+SHELIX+HDGYP                                                                                             339    Shewanella oneidensis MR-1                                           response regulator [Shewanella oneidensis MR-1]                                  
24374084 8.6e-07: _1_[144..183]    TQNKQLKELNSSLEET---VLKRTE---QLKQTLLKFKHLAGERQK        24374084       REC+SHELIX+HDGYP                                                                                             441    Shewanella oneidensis MR-1                                           response regulator [Shewanella oneidensis MR-1]                                  
24375490 3.6e-06: _1_[122..161]    KTHLTLASRANQLESL---VQLRTQ---ELESARYKIIHKLGRAAE        24375490       REC+SHELIX+HDGYP                                                                                             357    Shewanella oneidensis MR-1                                           response regulator [Shewanella oneidensis MR-1]                                  
26989661   2e-10: _1_[147..186]    RQNAQLRLLNNDLEKR---VAARTA---ELQQTADMLDLAYEELKH        26989661       REC+SHELIX+HDGYP                                                                                             456    Pseudomonas putida KT2440                                            response regulator [Pseudomonas putida KT2440]                                   
27366537   9e-09: _1_[126..165]    HAMEELAQQNTVLEDK---VKARTH---ELESLQDATIGAMASLAE        27366537       REC+SHELIX+HDGYP                                                                                             378    Vibrio vulnificus CMCP6                                              Response regulator [Vibrio vulnificus CMCP6]                                     
27367376  0.0011: _1_[129..168]    RSKDFLRDQNGYLEKE---VLKRSH---ELDRMQDAVVFALASLAE        27367376       REC+SHELIX+HDGYP                                                                                             363    Vibrio vulnificus CMCP6                                              Response regulator [Vibrio vulnificus CMCP6]                                     
32470755 2.4e-08: _1_[161..200]    SFQDRLQHHAAELELK---VEQRTR---ELEASRREVIYCLARAAE        32470755       REC+SHELIX+HDGYP                                                                                             418    Rhodopirellula baltica SH 1                                          response regulator [Rhodopirellula baltica SH 1]                                 
34496338  0.0004: _1_[118..157]    AARDILKNQNDYLEEE---VRRRTR---ETETVQNVAIWALASLAE        34496338       REC+SHELIX+HDGYP                                                                                             360    Chromobacterium violaceum ATCC 12472                                 probable two component system, transcriptional regulatory protein [Chromobacterium violaceum ATCC 12472]
34497952   0.097: _1_[131..170]    AVADFLEDKAAFLQTE---VERRTR---EVQVIQDVTIMALASLAE        34497952       REC+SHELIX+HDGYP                                                                                             378    Chromobacterium violaceum ATCC 12472                                 probable two component system, transcriptional regulatory protein [Chromobacterium violaceum ATCC 12472]
34498701   0.015: _1_[125..164]    QARDRLSDQNRWLEVE---VSRRMA---ENDLTQRMAIHALARLAE        34498701       REC+SHELIX+HDGYP                                                                                             366    Chromobacterium violaceum ATCC 12472                                 probable two component system, transcriptional regulatory protein [Chromobacterium violaceum ATCC 12472]
34558036 1.3e-08: _1_[115..154]    RTHLELRRYQNHLERL---VSERTQ---EVEKLRNAVIEALGGMAE        34558036       REC+SHELIX+HDGYP                                                                                             349    Wolinella succinogenes DSM 1740                                      SIGNAL TRANSDUCTION RESPONSE REGULATOR [Wolinella succinogenes DSM 1740]         
37676245   9e-09: _1_[126..165]    HAMEELAQQNTVLEDK---VKARTH---ELESLQDATIGAMASLAE        37676245       REC+SHELIX+HDGYP                                                                                             378    Vibrio vulnificus YJ016                                              response regulator [Vibrio vulnificus YJ016]                                     
37677133  0.0011: _1_[129..168]    RSKDFLRDQNGYLEKE---VLKRSH---ELDRMQDAVVFALASLAE        37677133       REC+SHELIX+HDGYP                                                                                             364    Vibrio vulnificus YJ016                                              response regulator [Vibrio vulnificus YJ016]                                     
39996754 6.4e-07: _1_[122..161]    RLLIENREYQANLEKK---VLEQTG---QIRSAMEELNLTYDHTLT        39996754       REC+SHELIX+HDGYP                                                                                             349    Geobacter sulfurreducens PCA                                         response regulator, putative [Geobacter sulfurreducens PCA]                      
39996989 3.4e-08: _1_[126..165]    RLLKIEKNYKAELENT---VKQRTG---ELADTLQMLKNMSKEVVQ        39996989       REC+SHELIX+HDGYP                                                                                             379    Geobacter sulfurreducens PCA                                         response regulator [Geobacter sulfurreducens PCA]                                
39997669 7.5e-05: _1_[124..163]    KLVDLSQDVNGILERE---VARKTA---EVRNALAMAREAEYEITL        39997669       REC+SHELIX+HDGYP                                                                                             368    Geobacter sulfurreducens PCA                                         response regulator [Geobacter sulfurreducens PCA]                                
46562169   1e-07: _1_[123..162]    RLLRENRMHRENLECL---VRQRTE---ELEETRRQVMQRLSRAAE        46562169       REC+SHELIX+HDGYP                                                                                             363    Desulfovibrio vulgaris subsp. vulgaris str. Hildenborough            response regulator [Desulfovibrio vulgaris subsp. vulgaris str. Hildenborough]   
46579592 9.3e-10: _1_[128..167]    ALIAENTAYSNGLEQM---VRQRTA---ELEAASSRLHTTLFATVE        46579592       REC+SHELIX+HDGYP                                                                                             356    Desulfovibrio vulgaris subsp. vulgaris str. Hildenborough            response regulator [Desulfovibrio vulgaris subsp. vulgaris str. Hildenborough]   
51244497 7.9e-07: _1_[131..170]    AYILREQEYKKSLEHE---IRIKTA---QLEKQNKLLTSYNKSIKN        51244497       REC+SHELIX+HDGYP                                                                                             374    Desulfotalea psychrophila LSv54                                      similar to two-component system response regulator (Ntr family) [Desulfotalea psychrophila LSv54]
53716379 2.5e-07: _1_[173..212]    KQNDALRAFNAGLEAQ---VQARVE---EIRQTMLFLEDAQRDLTH        53716379       REC+SHELIX+HDGYP                                                                                             475    Burkholderia mallei ATCC 23344                                       response regulator [Burkholderia mallei ATCC 23344]                              
53718347 2.2e-06: _1_[134..173]    KQNEELVAFSTELEAQ---VHARTE---EIRQTVMFLEDAQRDLKR        53718347       REC+SHELIX+HDGYP                                                                                             439    Burkholderia pseudomallei K96243                                     putative two-component response regulator protein [Burkholderia pseudomallei K96243]
53722669 2.5e-07: _1_[173..212]    KQNDALRAFNAGLEAQ---VQARVE---EIRQTMLFLEDAQRDLTH        53722669       REC+SHELIX+HDGYP                                                                                             475    Burkholderia pseudomallei K96243                                     probable two-component response regulator [Burkholderia pseudomallei K96243]     
53724711 2.2e-06: _1_[134..173]    KQNEELVAFSTELEAQ---VHARTE---EIRQTVMFLEDAQRDLKR        53724711       REC+SHELIX+HDGYP                                                                                             439    Burkholderia mallei ATCC 23344                                       response regulator [Burkholderia mallei ATCC 23344]                              
# 24            :                                                                        # 24
13474737 1.2e-09: _1_[756..795]    EQDLALKRANESLEQR---VKTRTI---ELTSVNEELTRVNEELAQ        13474737       TM+TM+TM+TM+TM+TM+TM+TM+TM+TM+TM+TM+PAS+SHELIX+HISKIN+REC                                                    1169   Mesorhizobium loti MAFF303099                                        two-component sensor histidine kinase [Mesorhizobium loti MAFF303099]            
15598467 1.8e-11: _1_[755..794]    EAEQGLKGANESLEQR---VQERTQ---ELSQLNQELSEAKSNAEA        15598467       TM+TM+TM+TM+TM+TM+TM+TM+TM+TM+TM+TM+PAS+SHELIX+HISKIN+REC                                                1159   Pseudomonas aeruginosa PAO1                                          probable two-component sensor [Pseudomonas aeruginosa PAO1]                      
15640331 2.9e-09: _1_[745..784]    EAEQSLKMANETLEER---VLQRTL---ELEKLNKQLVTATQRSER        15640331       TM+TM+TM+TM+TM+TM+TM+TM+TM+TM+TM+TM+PAS+SHELIX+HISKIN+REC                                                    1147   Vibrio cholerae O1 biovar eltor str. N16961                          sensor histidine kinase [Vibrio cholerae O1 biovar eltor str. N16961]            
15887876   1e-10: _1_[758..797]    ASDQALKQANETLEQR---VEERTV---ELTRVNRALAEARASADE        15887876       TM+TM+TM+TM+TM+TM+TM+TM+TM+TM+TM+TM+PAS+SHELIX+HISKIN+REC                                                    1169   Agrobacterium tumefaciens str. C58                                   hypothetical protein AGR_C_932 [Agrobacterium tumefaciens str. C58]              
15964306   3e-10: _1_[757..796]    AADMALKQANETLELR---VAERTG---ELTRVNRELGEARAAAEE        15964306       TM+TM+TM+TM+TM+TM+TM+TM+TM+TM+TM+TM+PAS+SHELIX+HISKIN+REC                                                    1168   Sinorhizobium meliloti 1021                                          PUTATIVE SENSORY TRANSDUCTION HISTIDINE KINASE TRANSMEMBRANE PROTEIN [Sinorhizobium meliloti 1021]
17934439   1e-10: _1_[758..797]    ASDQALKQANETLEQR---VEERTV---ELTRVNRALAEARASADE        17934439       TM+TM+TM+TM+TM+TM+TM+TM+TM+TM+TM+TM+PAS+SHELIX+HISKIN+REC                                                    1169   Agrobacterium tumefaciens str. C58                                   two component sensor kinase/response regulator hybrid [Agrobacterium tumefaciens str. C58]
17987889 3.7e-08: _1_[755..794]    EADLMRQKASEMLEQR---VIDRTA---ELMHVNDELARAQAAAEE        17987889       TM+TM+TM+TM+TM+TM+TM+TM+TM+TM+TM+TM+PAS+SHELIX+HISKIN+REC                                                    1305   Brucella melitensis 16M                                              SENSORY TRANSDUCTION HISTIDINE KINASE [Brucella melitensis 16M]                  
21233498   1e-08: _1_[754..793]    RAERALLDANENLEQR---VADRSR---EAELAQQSKTRFLAAISH        21233498       TM+TM+TM+TM+TM+TM+TM+TM+TM+TM+TM+TM+PAS+SHELIX+HISKIN+REC                                                    1147   Xanthomonas campestris pv. campestris str. ATCC 33913                two-component system sensor protein [Xanthomonas campestris pv. campestris str. ATCC 33913]
21244910   1e-08: _1_[755..794]    RAERALLDANENLEQR---VADRSR---EAELAQQSKTRFLAAISH        21244910       TM+TM+TM+TM+TM+TM+TM+TM+TM+TM+TM+TM+PAS+SHELIX+HISKIN+REC                                                    1150   Xanthomonas axonopodis pv. citri str. 306                            two-component system sensor protein [Xanthomonas axonopodis pv. citri str. 306]  
23501223 3.7e-08: _1_[758..797]    EADLMRQKASEMLEQR---VIDRTA---ELMHVNDELARAQAAAEE        23501223       TM+TM+TM+TM+TM+TM+TM+TM+TM+TM+TM+TM+PAS+SHELIX+HISKIN+REC                                                    1174   Brucella suis 1330                                                   sensor histidine kinase/response regulator [Brucella suis 1330]                  
24374283 1.3e-08: _1_[740..779]    QQERALLEANETLESR---VKERTF---ELAMLNSELLEAKAQEEM        24374283       TM+TM+TM+TM+TM+TM+TM+TM+TM+TM+TM+TM+PAS+SHELIX+HISKIN+REC                                                    1145   Shewanella oneidensis MR-1                                           sensor histidine kinase/response regulator [Shewanella oneidensis MR-1]          
26988427 9.2e-12: _1_[753..792]    EAEQALRDANERLEQR---VAERTH---ELSQLNQALSEAKSQAEA        26988427       TM+TM+TM+TM+TM+TM+TM+TM+TM+TM+TM+TM+PAS+SHELIX+HISKIN+REC                                                    1159   Pseudomonas putida KT2440                                            sodium-solute symporter/sensory box histidine kinase/response regulator, putative [Pseudomonas putida KT2440]
27364643 1.5e-09: _1_[745..784]    DAEQALKEANETLEER---VHLRTR---ELEQLNKQLVVATQRSEL        27364643       TM+TM+TM+TM+TM+TM+TM+TM+TM+TM+TM+TM+PAS+SHELIX+HISKIN+REC                                                    1143   Vibrio vulnificus CMCP6                                              Signal transduction histidine kinase [Vibrio vulnificus CMCP6]                   
27376310 5.1e-10: _1_[760..799]    EAAEALERANATLEKR---VRDRTE---ELTRLNSELALAKSTAED        27376310       TM+TM+TM+TM+TM+TM+TM+TM+TM+TM+TM+TM+PAS+SHELIX+HISKIN+REC                                                    1169   Bradyrhizobium japonicum USDA 110                                    two-component hybrid sensor and regulator [Bradyrhizobium japonicum USDA 110]    
27381000 3.4e-10: _1_[778..817]    RAAEALRRANEDLELR---VRERTE---ALERAKAEAEQANLGKTR        27381000       TM+TM+TM+TM+TM+TM+TM+TM+TM+TM+TM+TM+PAS+SHELIX+HISKIN+REC                                                    1190   Bradyrhizobium japonicum USDA 110                                    two-component hybrid sensor and regulator [Bradyrhizobium japonicum USDA 110]    
28871052   3e-11: _1_[753..792]    AAEQALKGVNESLEQR---VAARTH---ELSQLNQALTEAKGTAEA        28871052       TM+TM+TM+TM+TM+TM+TM+TM+TM+TM+TM+TM+PAS+SHELIX+HISKIN+REC                                                    1157   Pseudomonas syringae pv. tomato str. DC3000                          sensory box histidine kinase/response regulator [Pseudomonas syringae pv. tomato str. DC3000]
28899648 1.9e-11: _1_[745..784]    DAEQALKDANETLEER---VRARTR---ELEQLNKQLVAATQRSDL        28899648       TM+TM+TM+TM+TM+TM+TM+TM+TM+TM+TM+TM+PAS+SHELIX+HISKIN+REC                                                    1143   Vibrio parahaemolyticus RIMD 2210633                                 sensor histidine kinase [Vibrio parahaemolyticus RIMD 2210633]                   
37681313 1.5e-09: _1_[758..797]    DAEQALKEANETLEER---VHLRTR---ELEQLNKQLVVATQRSEL        37681313       TM+TM+TM+TM+TM+TM+TM+TM+TM+TM+TM+TM+PAS+SHELIX+HISKIN+REC                                                    1156   Vibrio vulnificus YJ016                                              sensor histidine kinase [Vibrio vulnificus YJ016]                                
39933930 5.4e-10: _1_[759..798]    EAAEALERANATLERR---VRERTE---ELTRLNSQLALAKSTAEE        39933930       TM+TM+TM+TM+TM+TM+TM+TM+TM+TM+TM+TM+PAS+SHELIX+HISKIN+REC                                                    1169   Rhodopseudomonas palustris CGA009                                    sensor histidine kinase, possible proline sensor PrlS [Rhodopseudomonas palustris CGA009]
50086428 2.6e-05: _1_[773..812]    ADITAFRENEAILEAR---VSDRTQ---QLENALSEQQLAREQADK        50086428       TM+TM+TM+TM+TM+TM+TM+TM+TM+TM+TM+TM+PAS+SHELIX+HISKIN+REC                                                    1176   Acinetobacter sp. ADP1                                               putative two-component sensor [Acinetobacter sp. ADP1]                           
54310465 3.5e-11: _1_[747..786]    QAEQALKDANEHLEAR---VQKRTQ---ELEQLNRRLVNATQQAEQ        54310465       TM+TM+TM+TM+TM+TM+TM+TM+TM+TM+TM+TM+PAS+SHELIX+HISKIN+REC                                                    1154   Photobacterium profundum SS9                                         putative Signal transduction histidinekinase [Photobacterium profundum SS9]      
56461105 3.9e-10: _1_[735..774]    ETQQALEDANIDLEQR---VDVRTQ---EIREINQELTAEIDRRRQ        56461105       TM+TM+TM+TM+TM+TM+TM+TM+TM+TM+TM+TM+PAS+SHELIX+HISKIN+REC                                                 1157   Idiomarina loihiensis L2TR                                           Multidomain protein contains Na+/proline symporter PutP-like domain, sensory histidine kinase and receiver domain [Idiomarina loihiensis L2TR]
56461252 2.8e-08: _1_[724..763]    EAQRQLQKINQELEER---VDERTQ---QLLSAKEAEEKAHESKSR        56461252       TM+TM+TM+TM+TM+TM+TM+TM+TM+TM+TM+TM+PAS+SHELIX+HISKIN+REC                                                 1115   Idiomarina loihiensis L2TR                                           Multidomain protein, contains Na+/proline symporter PutP-like domain, sensory histidine kinase and receiver domain [Idiomarina loihiensis L2TR]
58579959   1e-08: _1_[822..861]    RAERALLDANENLEQR---VADRSR---EAELAQQSKTRFLAAISH        58579959       TM+TM+TM+TM+TM+TM+TM+TM+TM+TM+TM+TM+PAS+SHELIX+HISKIN+REC                                                    1217   Xanthomonas oryzae pv. oryzae KACC10331                              two-component system sensor protein [Xanthomonas oryzae pv. oryzae KACC10331]    
# 18            :                                                                        # 18
20092820 1.1e-09: _1_[224..263]    GPEVRSKEAYDHLEEL---VKERTI---QLKKAYIQLKESEKDLAE        20092820       MEDS+SHELIX+PAS+PAS+HISKIN                                                                                   729    Methanosarcina acetivorans C2A                                       sensory transduction histidine kinase [Methanosarcina acetivorans C2A]           
20089379 1.5e-12: _1_[472..511]    KAEKALKKAHDNLEKL---VEERTG---QLEKAYNSLKESEKSLSE        20089379       PocR+PAS+PAS+SHELIX+PAS+HISKIN                                                                               848    Methanosarcina acetivorans C2A                                       sensory transduction histidine kinase [Methanosarcina acetivorans C2A]           
20090483   4e-12: _1_[265..304]    KAEEALKEAYDSLEET---VKERTA---ELEKSYISLKESESSLTE        20090483       PAS+GAF+PAS+SHELIX+PAS+PAS+PAS+HISKIN                                                                        899    Methanosarcina acetivorans C2A                                       sensory transduction histidine kinase [Methanosarcina acetivorans C2A]           
20090482 3.6e-11: _1_[580..619]    KAEEDLKKVHGNLEKL---VEERTA---ELENAYKSLKESEEGLAE        20090482       PAS+PocR+PAS+PAS+SHELIX+PAS+HISKIN                                                                           962    Methanosarcina acetivorans C2A                                       sensory transduction histidine kinase [Methanosarcina acetivorans C2A]           
20091607 1.2e-17: _2_[547..586]    EVETKLKETLDNLENL---VKVRTE---ELEIAFNSLKESERGLAE        20091607       PAS+PAS+SHELIX+PAS+PAS+SHELIX+PAS+PAS+HISKIN                                                              1061   Methanosarcina acetivorans C2A                                       sensory transduction histidine kinase [Methanosarcina acetivorans C2A]           
20090329 7.6e-07: _1_[135..174]    RAEDKLRSQHERLENL---VELRTL---EYIEANDKLAQEVTERKN        20090329       PAS+SHELIX+PAS+PAS+PAS+PAS+PAS+HISKIN                                                                        1066   Methanosarcina acetivorans C2A                                       sensory transduction histidine kinase [Methanosarcina acetivorans C2A]           
20092184 5.8e-11: _1_[462..501]    RAEEALMKIHKDLEEK---IKERTA---ELEKSYNSLKESERRLSE        20092184       GAF+PAS+PAS+SHELIX+PAS+PAS+PAS+HISKIN                                                                        1126   Methanosarcina acetivorans C2A                                       sensory transduction histidine kinase [Methanosarcina acetivorans C2A]           
20089644   1e-11: _1_[664..703]    KAELALKKVHDNLEKL---VEERTE---ELEKACRSLKESEEGLAK        20089644       MEDS+PocR+PAS+PAS+SHELIX+PAS+PAS+PAS+HISKIN                                                                  1281   Methanosarcina acetivorans C2A                                       sensory transduction histidine kinase [Methanosarcina acetivorans C2A]           
20089441 2.5e-11: _1_[448..487]    HMISQLGYSNIKMAQS---LAERDA---LLDAVRESEERFRSVLEH        20089441       MEDS+PAS+PocR+SHELIX+PAS+PAS+SHELIX+PAS+PAS+PAS+HISKIN                                                    1348   Methanosarcina acetivorans C2A                                       sensory transduction histidine kinase [Methanosarcina acetivorans C2A]           
21226271 1.7e-10: _1_[380..419]    QAEEDLNKVHYNLEKL---VEERTS---ELEKAYISLKESEKGLAE        21226271       PAS+PAS+PAS+SHELIX+PAS+HISKIN                                                                                762    Methanosarcina mazei Go1                                             hypothetical sensory transduction histidine kinase [Methanosarcina mazei Go1]    
21226620   3e-10: _1_[269..308]    KAEEALKEAHDSLEET---VTERTS---ELEKSYISLKESESRLAE        21226620       PAS+GAF+PAS+SHELIX+PAS+PAS+PAS+HISKIN                                                                        901    Methanosarcina mazei Go1                                             hypothetical sensory transduction histidine kinase [Methanosarcina mazei Go1]    
21227773 2.2e-11: _1_[472..511]    KAEDALKKAHDNLEKL---VEERTV---QLESAYESLKENEISLSE        21227773       PocR+PAS+PAS+SHELIX+PAS+HISKIN                                                                               848    Methanosarcina mazei Go1                                             hypothetical sensory transduction histidine kinase [Methanosarcina mazei Go1]    
21228377 2.1e-07: _1_[787..826]    EAESRLKNTLENLGNL---VKERTV---ELEKAYVLLKEREESLAE        21228377       MEDS+PAS+PocR+PAS+PAS+SHELIX+PAS+HISKIN                                                                      1171   Methanosarcina mazei Go1                                             hypothetical sensory transduction histidine kinase [Methanosarcina mazei Go1]    
21228378 6.9e-10: _1_[583..622]    EAETKLKETLENLEKL---VEDRTL---ELEKAYKSLKESETRLSE        21228378       MEDS+PAS+PAS+PAS+SHELIX+PAS+PAS+HISKIN                                                                       1107   Methanosarcina mazei Go1                                             hypothetical sensory transduction histidine kinase [Methanosarcina mazei Go1]    
21228537   1e-10: _1_[254..293]    KAEAKLKETLDNLEEL---VKQRTE---ELEKAYDSLKESEEHLRL        21228537       PAS+PAS+SHELIX+PAS+PAS+PAS+HISKIN                                                                            885    Methanosarcina mazei Go1                                             hypothetical sensory transduction histidine kinase [Methanosarcina mazei Go1]    
21228617 2.5e-05: _1_[120..159]    QAEEELGSRYKRLEEL---VRLKSA---ECMEVNERLAKEVIERQR        21228617       PAS+SHELIX+PAS+PAS+PAS+PAS+PAS+HISKIN                                                                        1050   Methanosarcina mazei Go1                                             hypothetical sensory transduction histidine kinase [Methanosarcina mazei Go1]    
21228748 1.6e-10: _1_[475..514]    IAEAKLKETLENLERL---VRKRTA---ELEKAYYSLKESEKGLAE        21228748       PocR+PAS+PAS+SHELIX+PAS+HISKIN                                                                               883    Methanosarcina mazei Go1                                             sensory transduction protein kinase [Methanosarcina mazei Go1]                   
21228875 6.6e-10: _1_[165..204]    KTESTLKETLDNLEEM---IKKRTE---EIEKAYRSLKESEKCLAE        21228875       PAS+SHELIX+PAS+PAS+PAS+HISKIN                                                                                807    Methanosarcina mazei Go1                                             Two component system histidine kinase [Methanosarcina mazei Go1]                 
# 11            :                                                                        # 11
17227819 2.1e-09: _1_[1495..1534]  AISLENTRLYLTLEAR---VQERTQ---ELEDKNYQLQNILGELQR        17227819       STYkin+AAAatpase+GAF+SHELIX+HISKIN                                                                           1808   Nostoc sp. PCC 7120                                                  serine/threonine kinase with two-component sensor domain [Nostoc sp. PCC 7120]   
17228381 6.8e-11: _1_[1512..1551]  QLYGQLAEYSQNLELK---VEQRTQ---ELKEKANQLESALQKLSS        17228381       STYkin+AAAatpase+GAF+SHELIX+HISKIN                                                                           1850   Nostoc sp. PCC 7120                                                  serine/threonine kinase with two-component sensor domain [Nostoc sp. PCC 7120]   
17229750 1.4e-08: _1_[1459..1498]  AISLENARLYANLEEK---IEERTR---ELNENNLRLRQTLHELKL        17229750       STYkin+AAAatpase+GAF+SHELIX+HISKIN                                                                           1777   Nostoc sp. PCC 7120                                                  serine/threonine kinase with two-component sensor domain [Nostoc sp. PCC 7120]   
17229774 9.6e-09: _1_[1480..1519]  LLYEQLEYYSRNLEIK---VEERTS---QLKAAQNKIIAQEKLASL        17229774       STYkin+AAAatpase+GAF+SHELIX+HISKIN                                                                           1783   Nostoc sp. PCC 7120                                                  serine/threonine kinase with two-component sensor domain [Nostoc sp. PCC 7120]   
17232179 2.1e-11: _1_[1577..1616]  QANQNLEDYNHRLEEK---VEARTQ---ELNHKNHRLQQTLQELQR        17232179       STYkin+AAAatpase+GAF+SHELIX+HISKIN                                                                           1900   Nostoc sp. PCC 7120                                                  serine/threonine kinase with two-component sensor domain [Nostoc sp. PCC 7120]   
24213864   1e-07: _1_[1493..1532]  KLYEDITSLNAELEKK---VELRTQ---ELMQSLKIIRKDLLYSKK        24213864       STYkin+AAAatpase+GAF+SHELIX+pp2cSIG                                                                          1759   Leptospira interrogans serovar Lai str. 56601                        Probable serine/threonine-protein kinase [Leptospira interrogans serovar Lai str. 56601]
24214122 5.1e-10: _1_[1495..1534]  AISIDNALLYSNMEEK---VRERTR---ELAQANADLELKNQRITD        24214122       STYkin+AAAatpase+GAF+SHELIX+pp2cSIG                                                                          1780   Leptospira interrogans serovar Lai str. 56601                        Serine/threonine protein kinase [Leptospira interrogans serovar Lai str. 56601]  
24215812 4.8e-08: _1_[1462..1501]  AIFIENANLYAELEEK---VKQRTS---ELDNTIHLLRKDLLYAQK        24215812       STYkin+AAAatpase+GAF+SHELIX+pp2cSIG                                                                          1731   Leptospira interrogans serovar Lai str. 56601                        Serine/threonine protein kinase [Leptospira interrogans serovar Lai str. 56601]  
45656877 4.8e-08: _1_[1462..1501]  AIFIENANLYAELEEK---VKQRTS---ELDNTIHLLRKDLLYAQK        45656877       STYkin+AAAatpase+GAF+SHELIX+pp2cSIG                                                                          1731   Leptospira interrogans serovar Copenhageni str. Fiocruz L1-130       serine/threonine kinase with GAF domain [Leptospira interrogans serovar Copenhageni str. Fiocruz L1-130]
45658172 5.1e-10: _1_[1495..1534]  AISIDNALLYSNMEEK---VRERTR---ELAQANADLELKNQRITD        45658172       STYkin+AAAatpase+GAF+SHELIX+pp2cSIG                                                                          1780   Leptospira interrogans serovar Copenhageni str. Fiocruz L1-130       serine/threonine kinase with GAF domain [Leptospira interrogans serovar Copenhageni str. Fiocruz L1-130]
45658363 9.8e-08: _1_[1493..1532]  KLYEDITSLNAELERK---VELRTQ---ELMQSLKIIRKDLLYSKK        45658363       STYkin+AAAatpase+GAF+SHELIX+pp2cSIG                                                                          1759   Leptospira interrogans serovar Copenhageni str. Fiocruz L1-130       serine/threonine kinase [Leptospira interrogans serovar Copenhageni str. Fiocruz L1-130]
32472478   1e-06: _1_[1374..1413]  NSFQQLQDLNLNLEKK---VHERTE---SVVRHSKELELTAQQLTA        32472478       STYkin+AAAatpase+GAF+SHELIX+HISKIN+REC+HPT                                                                   1998   Rhodopirellula baltica SH 1                                          receptor-like histidine kinase BpdS [Rhodopirellula baltica SH 1]                
# 11            :                                                                        # 11
21230974   2e-06: _1_[835..874]    FLLRRYKTHNARLAEL---VRKRTE---DLQRQAQRLLQANQEKSE        21230974       TM+SHELIX+GGDEF                                                                                              1050   Xanthomonas campestris pv. campestris str. ATCC 33913                hypothetical protein XCC1520 [Xanthomonas campestris pv. campestris str. ATCC 33913]
21231360 2.3e-05: _1_[779..818]    WRHRHLVARERMLAEL---VAERTY---QLEREKRELETARAALAL        21231360       TM+TM+SHELIX+GGDEF                                                                                           987    Xanthomonas campestris pv. campestris str. ATCC 33913                GGDEF family protein [Xanthomonas campestris pv. campestris str. ATCC 33913]     
21231361 1.6e-09: _1_[779..818]    WRTRKLLARKQELEHL---IAERTA---ELEQDKRDLEAARAALTH        21231361       TM+SHELIX+GGDEF                                                                                              996    Xanthomonas campestris pv. campestris str. ATCC 33913                GGDEF family protein [Xanthomonas campestris pv. campestris str. ATCC 33913]     
21232762   5e-05: _1_[791..830]    WRTASYRRRQRELNVV---IDKRTR---ELSDKNLALQQASQEREA        21232762       TM+SHELIX+GGDEF                                                                                              999    Xanthomonas campestris pv. campestris str. ATCC 33913                hypothetical protein XCC3333 [Xanthomonas campestris pv. campestris str. ATCC 33913]
21242320 1.8e-06: _1_[794..833]    VLLHRYKTHNARLAEL---VRKRTE---DLQRQAQRLLQANQEKSE        21242320       TM+SHELIX+GGDEF                                                                                              1009   Xanthomonas axonopodis pv. citri str. 306                            hypothetical protein XAC1570 [Xanthomonas axonopodis pv. citri str. 306]         
21242683 0.00011: _1_[740..779]    WRHRHLVARERMLAEL---VSERTY---QLEREKRELENARAALAL        21242683       TM+SHELIX+GGDEF                                                                                              948    Xanthomonas axonopodis pv. citri str. 306                            GGDEF family protein [Xanthomonas axonopodis pv. citri str. 306]                 
21242684 1.4e-09: _1_[771..810]    WRTAKLLRRKRELEQL---VAKRTA---ELEQDKRDLEAARAELSL        21242684       TM+SHELIX+GGDEF                                                                                              980    Xanthomonas axonopodis pv. citri str. 306                            GGDEF family protein [Xanthomonas axonopodis pv. citri str. 306]                 
21244185 0.00021: _1_[775..814]    WRTASYRLRQRQLNTV---IDRRTR---ELSDKNLALQQASQEREA        21244185       TM+TM+SHELIX+GGDEF                                                                                           983    Xanthomonas axonopodis pv. citri str. 306                            hypothetical protein XAC3460 [Xanthomonas axonopodis pv. citri str. 306]         
56460549   0.006: _1_[782..821]    ARLNQYNRTRLQLEQM---VKEKTA---DL-ETMARQDPLTNLGNR        56460549       TM+SHELIX+GGDEF                                                                                              970    Idiomarina loihiensis L2TR                                           Signaling protein with a ligand-binding sensor domain and GGDEF domain [Idiomarina loihiensis L2TR]
58582237 4.2e-10: _1_[772..811]    WRTAKLLRRKHELEQL---VAERTA---ELEQDKRDLEAARAELSL        58582237       TM+SHELIX+GGDEF                                                                                              990    Xanthomonas oryzae pv. oryzae KACC10331                              GGDEF family protein [Xanthomonas oryzae pv. oryzae KACC10331]                   
58582238 0.00011: _1_[765..804]    WRHRHLVARERMLAEL---VSERTY---QLEREKRELENARAALAL        58582238       TM+TM+SHELIX+GGDEF                                                                                           973    Xanthomonas oryzae pv. oryzae KACC10331                              GGDEF family protein [Xanthomonas oryzae pv. oryzae KACC10331]                   
# 11            :                                                                        # 11
13471756 0.00054: _1_[162..199]    RTMTMQVEAAETLERR---VEQRTA---QIQHLATH--DILTGLPN        13471756       CACHE+TM+SHELIX+GGDEF+EAL                                                                                    614    Mesorhizobium loti MAFF303099                                        hypothetical protein mlr1828 [Mesorhizobium loti MAFF303099]                     
15601540  0.0059: _1_[155..194]    YQRETLLQMNQDLHFK---VRHKMQ---SIASLNQSLHQEIDKRRA        15601540       GAF+SHELIX+GGDEF+EAL                                                                                         627    Vibrio cholerae O1 biovar eltor str. N16961                          GGDEF family protein [Vibrio cholerae O1 biovar eltor str. N16961]               
17229798 8.4e-11: _1_[132..171]    TARIEILTLNAELEQR---VKQRTW---ELEKALQKLQIEVSARQK        17229798       REC+SHELIX+GGDEF+EAL                                                                                         611    Nostoc sp. PCC 7120                                                  two-component response regulator [Nostoc sp. PCC 7120]                           
27366597 3.1e-08: _1_[155..194]    YRKEELAQLNQELEQK---VAERTK---TLADLSSRLLTEIEQRTA        27366597       GAF+SHELIX+GGDEF+EAL                                                                                         630    Vibrio vulnificus CMCP6                                              Predicted signal transduction protein [Vibrio vulnificus CMCP6]                  
27366713 5.1e-05: _1_[155..194]    YQHQKLLRLNEQLKQR---VDKRTT---NLAQLSFSLNQEIDRRKA        27366713       GAF+SHELIX+GGDEF+EAL                                                                                         638    Vibrio vulnificus CMCP6                                              Predicted signal transduction protein [Vibrio vulnificus CMCP6]                  
28900724   4e-05: _1_[155..194]    YQQYKLLRLNSELKNR---VENRTT---DLAQLSFSLTQEIDRRKA        28900724       GAF+SHELIX+GGDEF+EAL                                                                                         636    Vibrio parahaemolyticus RIMD 2210633                                 GGDEF family protein [Vibrio parahaemolyticus RIMD 2210633]                      
32472612  0.0077: _1_[161..198]    DSARAAGLKHRELEEL---VRVRTA---ELERV--ALHDGLTGLAN        32472612       REC+SHELIX+GGDEF+EAL                                                                                         614    Rhodopirellula baltica SH 1                                          conserved hypothetical protein-putative response regulator [Rhodopirellula baltica SH 1]
34497763 6.6e-10: _1_[198..237]    RQAAALSETNRELDSR---VAARTW---ELANANARLEDEIQERRR        34497763       TM+SHELIX+GGDEF+EAL                                                                                          679    Chromobacterium violaceum ATCC 12472                                 hypothetical protein CV2308 [Chromobacterium violaceum ATCC 12472]               
37676311 3.1e-08: _1_[167..206]    YRKEELAQLNQELEQK---VAERTK---TLADLSSRLLTEIEQRTA        37676311       GAF+SHELIX+GGDEF+EAL                                                                                         642    Vibrio vulnificus YJ016                                              GGDEF family protein [Vibrio vulnificus YJ016]                                   
37676430 5.1e-05: _1_[177..216]    YQHQKLLRLNEQLKQR---VDKRTT---NLAQLSFSLNQEIDRRKA        37676430       GAF+SHELIX+GGDEF+EAL                                                                                         660    Vibrio vulnificus YJ016                                              GGDEF family protein [Vibrio vulnificus YJ016]                                   
52841291    0.48: _1_[20..59]      RSYYRERKAREEAELL---LENKTR---ELHLLNQDLEKKNAELLK        52841291       SHELIX+GGDEF+EAL                                                                                             506    Legionella pneumophila subsp. pneumophila str. Philadelphia 1        sensory box/GGDEF family [Legionella pneumophila subsp. pneumophila str. Philadelphia 1]
# 9             :                                                                        # 9
16120593 9.7e-10: _1_[361..400]    NLLDTLNEQYDTLEMK---VKERTL---ALAEAKRAAEQANRRKSD        16120593       TM+TM+TM+HAMP+SHELIX+HISKIN+REC+HPT                                                                          939    Yersinia pestis CO92                                                 two-component sensor/regulator [Yersinia pestis CO92]                            
16760519 4.8e-11: _1_[359..398]    QLLDTLQVQYDNLENK---VAERTQ---ALNEAKKRAEQANKRKSI        16760519       TM+HAMP+SHELIX+HISKIN+REC+HPT                                                                                920    Salmonella enterica subsp. enterica serovar Typhi str. CT18          putative two-component sensor kinase [Salmonella enterica subsp. enterica serovar Typhi str. CT18]
16764742   7e-11: _1_[359..398]    QLLDTLQVQYDNLENK---VAERTQ---ALNEAKKRAERANKRKSI        16764742       TM+HAMP+SHELIX+HISKIN+REC+HPT                                                                                920    Salmonella typhimurium LT2                                           sensor kinase [Salmonella typhimurium LT2]                                       
22124428 9.7e-10: _1_[351..390]    NLLDTLNEQYDTLEMK---VKERTL---ALAEAKRAAEQANRRKSD        22124428       TM+TM+HAMP+SHELIX+HISKIN+REC+HPT                                                                             929    Yersinia pestis KIM                                                  putative response regulator [Yersinia pestis KIM]                                
29141721 4.8e-11: _1_[359..398]    QLLDTLQVQYDNLENK---VAERTQ---ALNEAKKRAEQANKRKSI        29141721       TM+HAMP+SHELIX+HISKIN+REC+HPT                                                                                920    Salmonella enterica subsp. enterica serovar Typhi Ty2                putative two-component sensor kinase [Salmonella enterica subsp. enterica serovar Typhi Ty2]
34498055 3.8e-08: _1_[360..399]    KLLATIRSHYRTLEAR---VHKRTQ---ELDEA-RKIAELMSRRKS        34498055       TM+TM+HAMP+SHELIX+HISKIN+REC+HPT                                                                             924    Chromobacterium violaceum ATCC 12472                                 probable two-component sensor/regulator [Chromobacterium violaceum ATCC 12472]   
45440264 9.7e-10: _1_[351..390]    NLLDTLNEQYDTLEMK---VKERTL---ALAEAKRAAEQANRRKSD        45440264       TM+TM+HAMP+SHELIX+HISKIN+REC+HPT                                                                             929    Yersinia pestis biovar Medievalis str. 91001                         two-component sensor/regulator [Yersinia pestis biovar Medievalis str. 91001]    
51594666 6.2e-10: _1_[361..400]    NLLDTLNEQYDTLEMK---VKERTL---ALAKAKRAAEQANRRKSD        51594666       TM+TM+TM+HAMP+SHELIX+HISKIN+REC+HPT                                                                          939    Yersinia pseudotuberculosis IP 32953                                 two-component sensor/regulator [Yersinia pseudotuberculosis IP 32953]            
56413637 4.8e-11: _1_[359..398]    QLLDTLQVQYDNLENK---VAERTQ---ALNEAKKRAEQANKRKSI        56413637       TM+HAMP+SHELIX+HISKIN+REC+HPT                                                                                920    Salmonella enterica subsp. enterica serovar Paratyphi A str. ATCC    putative two-component sensor kinase [Salmonella enterica subsp. enterica serovar Paratyphi A str. ATCC 9150]
# 7             :                                                                        # 7
15609229   0.001: _1_[681..720]    ERYLRIERDNAQLERK---VAAATN---SLARTFDRFVGLLTEREF        15609229       rhel+SHELIX                                                                                                  906    Mycobacterium tuberculosis H37Rv                                     PROBABLE ATP-DEPENDENT DNA HELICASE HELY [Mycobacterium tuberculosis H37Rv]      
15827694 0.00012: _1_[695..734]    ERYLRIERYNAQLQRK---VAAATN---SLARTFDRIVGLLIERDF        15827694       rhel+SHELIX                                                                                                  920    Mycobacterium leprae TN                                              probable helicase, Ski2 subfamily [Mycobacterium leprae TN]                      
15841583   0.001: _1_[681..720]    ERYLRIERDNAQLERK---VAAATN---SLARTFDRFVGLLTEREF        15841583       rhel+SHELIX                                                                                                  906    Mycobacterium tuberculosis CDC1551                                   ATP-dependent RNA helicase, DEAD/DEAH box family [Mycobacterium tuberculosis CDC1551]
21220127  0.0038: _1_[731..770]    ERYHRLLRDTSQLERR---IEGRTN---TIARTFDRIVALLTELDY        21220127       rhel+SHELIX                                                                                                  950    Streptomyces coelicolor A3(2)                                        putative helicase [Streptomyces coelicolor A3(2)]                                
29833237   0.031: _1_[716..755]    ERYYRLMRDTSQLERR---IEGRTN---TIARTFDRIVALLTELDY        29833237       rhel+SHELIX                                                                                                  937    Streptomyces avermitilis MA-4680                                     putative ATP-dependent RNA helicase [Streptomyces avermitilis MA-4680]           
31793275   0.001: _1_[681..720]    ERYLRIERDNAQLERK---VAAATN---SLARTFDRFVGLLTEREF        31793275       rhel+SHELIX                                                                                                  906    Mycobacterium bovis AF2122/97                                        PROBABLE ATP-DEPENDENT DNA HELICASE HELY [Mycobacterium bovis AF2122/97]         
41407926  0.0021: _1_[710..749]    ERYLRIERDNAQLEKK---VATATN---SLARTFDRIVGLLTERGF        41407926       rhel+SHELIX                                                                                                  821    Mycobacterium avium subsp. paratuberculosis K-10                     HelY [Mycobacterium avium subsp. paratuberculosis K-10]                          
32477939   0.039: _1_[481..520]    AGRGRWRLIADELEHR---VADRLE---EATRVRSEDRQVALREMV        32477939       rhel+SHELIX                                                                                                  560    Rhodopirellula baltica SH 1                                          rhel+SHELIX ATP-dependent DNA helicase RecQ [Rhodopirellula baltica SH 1]     
55376574   0.013: _1_[693..732]    AIKQSLQRRMEKLDEK---VEDMTE---EVSELSRDAGVTEATIDE        55376574       SHELIX+rhel                                                                                                  1212   Haloarcula marismortui ATCC 43049                                    putative DEAD/H helicase [Haloarcula marismortui ATCC 43049]                     
# 7             :                                                                        # 7
15641529 3.4e-07: _1_[393..432]    QRNEEIVQAAHELEAK---VQSRTA---SLQEKTAQLEHHIALLNQ        15641529       TM+TM+HAMP+SHELIX+HISKIN                                                                                     677    Vibrio cholerae O1 biovar eltor str. N16961                          sensor histidine kinase [Vibrio cholerae O1 biovar eltor str. N16961]            
24376189 2.6e-06: _1_[415..454]    RRNAQIQAAAEQLEMK---VEERTR---SLQDKTLELQRNVALLNE        24376189       TM+TM+HAMP+SHELIX+HISKIN                                                                                     709    Shewanella oneidensis MR-1                                           sensor histidine kinase [Shewanella oneidensis MR-1]                             
27365874 2.9e-07: _1_[376..415]    TREEQIKQAANELEQK---VLDRTA---SLNEKTEQLEHHIRLLNQ        27365874       TM+HAMP+SHELIX+HISKIN                                                                                        660    Vibrio vulnificus CMCP6                                              Signal transduction histidine kinase [Vibrio vulnificus CMCP6]                   
28898277   1e-07: _1_[396..435]    DRKIELKNAAAQLECK---VQERTA---SLREKTEELELHIQLLNQ        28898277       TM+HAMP+SHELIX+HISKIN                                                                                        679    Vibrio parahaemolyticus RIMD 2210633                                 sensor histidine kinase [Vibrio parahaemolyticus RIMD 2210633]                   
37679895 1.7e-07: _1_[424..463]    TREQQIKQAANELEQK---VLERTA---SLNEKTEQLEHHIRLLNQ        37679895       TM+HAMP+SHELIX+HISKIN                                                                                        708    Vibrio vulnificus YJ016                                              signal transduction histidine kinase [Vibrio vulnificus YJ016]                   
39995881 1.3e-08: _1_[401..440]    QREEDIRKLNRELEQK---VLDRTA---QLEEKNLLLVKTQEDLVR        39995881       TM+TM+HAMP+SHELIX+HISKIN                                                                                     674    Geobacter sulfurreducens PCA                                         sensor histidine kinase [Geobacter sulfurreducens PCA]                           
54309073 2.4e-06: _1_[394..433]    QRNEDIKKASLELEDK---VQIRTR---SLHEKTEELEQHIKLLNQ        54309073       TM+HAMP+SHELIX+HISKIN                                                                                        681    Photobacterium profundum SS9                                         putative sensor histidine kinase [Photobacterium profundum SS9]                  
# 7             :                                                                        # 7
15641229 2.9e-10: _1_[172..211]    ERNRELVKLNQSLEEK---VIERTQ---QLYQVNRQLEALSMTDSL        15641229       SHELIX+GGDEF                                                                                                 372    Vibrio cholerae O1 biovar eltor str. N16961                          GGDEF family protein [Vibrio cholerae O1 biovar eltor str. N16961]               
16329623 1.4e-11: _1_[251..290]    QYYETIKNYSQTLEIR---VKERTM---ELEFAKQQLEQVNQRLKH        16329623       GAF+SHELIX+GGDEF                                                                                             464    Synechocystis sp. PCC 6803                                           PleD [Synechocystis sp. PCC 6803]                                                
27365688 4.2e-11: _1_[172..211]    ERNRELLSLNQHLEEK---VEERTR---QLVEANKQLEVLSYTDAL        27365688       SHELIX+GGDEF                                                                                                 371    Vibrio vulnificus CMCP6                                              GGDEF family protein [Vibrio vulnificus CMCP6]                                   
28898063 2.2e-08: _1_[172..211]    ERNRDLLKLNLELEEK---VEARTN---QLLTANKQLEALSLTDSL        28898063       SHELIX+GGDEF                                                                                                 381    Vibrio parahaemolyticus RIMD 2210633                                 GGDEF family protein [Vibrio parahaemolyticus RIMD 2210633]                      
37680156 4.2e-11: _1_[172..211]    ERNRELLSLNQHLEEK---VEERTR---QLVEANKQLEVLSYTDAL        37680156       SHELIX+GGDEF                                                                                                 371    Vibrio vulnificus YJ016                                              GGDEF family protein [Vibrio vulnificus YJ016]                                   
51244824 1.1e-11: _1_[172..211]    QRNKELNLLNQDLEQK---VRERTE---ELQQANRGLEKISLTDPL        51244824       SHELIX+GGDEF                                                                                                 370    Desulfotalea psychrophila LSv54                                      hypothetical protein DP0972 [Desulfotalea psychrophila LSv54]                    
51245891 5.7e-12: _1_[174..213]    LRNNELSQLNRNLEQQ---VQERTT---ELRKANRDLEKISQTDPL        51245891       SHELIX+GGDEF                                                                                                 374    Desulfotalea psychrophila LSv54                                      hypothetical protein DP2039 [Desulfotalea psychrophila LSv54]                    
# 6             :                                                                        # 6
30261120   0.031: _1_[150..189]    SLQSQLTDFRSTEEQL---TKKRAA---QLDTKNNKLELWLYSLLF        30261120       TM+CHASE3+SHELIX+TM+HAMP+SHELIX+HISKIN+REC                                                                896    Bacillus anthracis str. Ames                                         sensor histidine kinase/response regulator [Bacillus anthracis str. Ames]        
42780166    0.37: _1_[449..491]    MQAEELRTTNEQLESRTEEAEQKTA---DLQITKLELEEKASELLR        42780166       TM+CHASE3+TM+HAMP+SHELIX+HISKIN+REC                                                                          896    Bacillus cereus ATCC 10987                                           sensor histidine kinase/response regulator [Bacillus cereus ATCC 10987]          
47526275   0.031: _1_[150..189]    SLQSQLTDFRSTEEQL---TKKRAA---QLDTKNNKLELWLYSLLF        47526275       TM+CHASE3+SHELIX+TM+HAMP+SHELIX+HISKIN+REC                                                                896    Bacillus anthracis str. 'Ames Ancestor'                              sensor histidine kinase/response regulator [Bacillus anthracis str. 'Ames Ancestor']
49183954   0.031: _1_[150..189]    SLQSQLTDFRSTEEQL---TKKRAA---QLDTKNNKLELWLYSLLF        49183954       TM+CHASE3+SHELIX+TM+HAMP+SHELIX+HISKIN+REC                                                                896    Bacillus anthracis str. Sterne                                       sensor histidine kinase/response regulator [Bacillus anthracis str. Sterne]      
49480178   0.045: _1_[150..189]    SLQSQLTDFRSTEEQL---TKKRAA---QLDTKNNKLELWLYSLLF        49480178       TM+CHASE3+SHELIX+TM+HAMP+SHELIX+HISKIN+REC                                                                896    Bacillus thuringiensis serovar konkukian str. 97-27                  sensor histidine kinase [Bacillus thuringiensis serovar konkukian str. 97-27]    
52144326   0.012: _1_[150..189]    SLQSQLTDFRSTEEQL---TKKRSA---QLDTKNNKLELWLYSLLF        52144326       TM+CHASE3+SHELIX+TM+HAMP+SHELIX+HISKIN+REC                                                                896    Bacillus cereus E33L                                                 sensor histidine kinase [Bacillus cereus E33L]                                   
# 6             :                                                                        # 6
20807819  0.0013: _1_[71..110]     EAKEEIHRLRSDFEKE---VRDRRG---ELQRLEKRLLQKEEILEK        20807819       TM+SHELIX+HDGYP                                                                                              523    Thermoanaerobacter tengcongensis MB4                                 predicted HD superfamily hydrolase [Thermoanaerobacter tengcongensis MB4]        
29349825 0.00034: _1_[52..91]      EVKEKFLNKKADLEKE---VALRNQ---KIQQAENKLKQREMVLSQ        29349825       TM+SHELIX+HDGYP                                                                                              511    Bacteroides thetaiotaomicron VPI-5482                                hypothetical protein BT4417 [Bacteroides thetaiotaomicron VPI-5482]              
34540228 1.6e-05: _1_[54..93]      EVKEKFLQLKGDLEKQ---VAQRNS---KLQSVESKLKSREQTLNQ        34540228       TM+SHELIX+HDGYP                                                                                              513    Porphyromonas gingivalis W83                                         hypothetical protein PG0401 [Porphyromonas gingivalis W83]                       
46581075  0.0077: _1_[120..158]    RKERRLATLEEELEGK---IAEQDH---RLEEV-SGLTAEEARARI        46581075       TM+SHELIX+SHELIX+HDGYP                                                                                    519    Desulfovibrio vulgaris subsp. vulgaris str. Hildenborough            HDIG/HD/KH domain protein [Desulfovibrio vulgaris subsp. vulgaris str. Hildenborough]
53714887 0.00034: _1_[52..91]      EVKEKFLNKKADLEKE---VALRNQ---KIQQAENKLKQREMVLSQ        53714887       TM+SHELIX+HDGYP                                                                                              511    Bacteroides fragilis YCH46                                           hypothetical protein BF3602 [Bacteroides fragilis YCH46]                         
57233607   0.057: _1_[86..128]     SAEAELKERRSELAKQENRVMQKTE---ALDRKLENLEQREQSLTN        57233607       TM+SHELIX+HDGYP                                                                                              527    Dehalococcoides ethenogenes 195                                      KH domain/HDIG domain protein [Dehalococcoides ethenogenes 195]                  
# 5             :                                                                        # 5
24214896 2.6e-07: _1_[385..424]    KMKEKLKSWNSVLETA---IRERTK---ELSSSLEEVKNLKEQQDG        24214896       TM+TM+TM+TM+TM+TM+TM+SHELIX+pp2cSIG                                                                          789    Leptospira interrogans serovar Lai str. 56601                        hypothetical protein LA2196 [Leptospira interrogans serovar Lai str. 56601]      
24215376 8.3e-07: _1_[459..498]    RLNEEVEELNLGLEKK---VEQRTE---ELRLSLEQVNRLKVQQDA        24215376       TM+TM+TM+TM+TM+TM+TM+SHELIX+pp2cSIG                                                                          762    Leptospira interrogans serovar Lai str. 56601                        hypothetical protein LA2676 [Leptospira interrogans serovar Lai str. 56601]      
24216313 1.6e-07: _1_[399..438]    RVHKEAEELNLDLDQK---VKERTR---QLENTLEQVRELKIQQDG        24216313       TM+TM+TM+TM+TM+TM+TM+SHELIX+pp2cSIG                                                                          869    Leptospira interrogans serovar Lai str. 56601                        TPR-repeat-containing proteins [Leptospira interrogans serovar Lai str. 56601]   
45656491 1.6e-07: _1_[399..438]    RVHKEAEELNLDLDQK---VKERTR---QLENTLEQVRELKIQQDG        45656491       TM+TM+TM+TM+TM+TM+TM+SHELIX+pp2cSIG                                                                          869    Leptospira interrogans serovar Copenhageni str. Fiocruz L1-130       hypothetical protein LIC10593 [Leptospira interrogans serovar Copenhageni str. Fiocruz L1-130]
45657197 8.3e-07: _1_[459..498]    RLNEEVEELNLGLEKK---VEQRTE---ELRLSLEQVNRLKVQQDA        45657197       TM+TM+TM+TM+TM+TM+TM+SHELIX+pp2cSIG                                                                          762    Leptospira interrogans serovar Copenhageni str. Fiocruz L1-130       hypothetical protein LIC11317 [Leptospira interrogans serovar Copenhageni str. Fiocruz L1-130]
# 5             :                                                                        # 5
24214922 0.00078: _1_[151..190]    KITEELKNLTVSMETE---IYQRAQ---EIQKNNKELIRLNEELTQ        24214922       PAS+SHELIX+HISKIN+REC+HISKIN                                                                                 832    Leptospira interrogans serovar Lai str. 56601                        two-component hybrid sensor and regulator [Leptospira interrogans serovar Lai str. 56601]
45657575 0.00078: _1_[151..190]    KITEELKNLTVSMETE---IYQRAQ---EIQKNNKELIRLNEELTQ        45657575       PAS+SHELIX+HISKIN+REC+HISKIN                                                                                 832    Leptospira interrogans serovar Copenhageni str. Fiocruz L1-130       histidine kinase response regulator hybrid protein [Leptospira interrogans serovar Copenhageni str. Fiocruz L1-130]
52842360 0.00031: _1_[151..190]    KLMEELRTKAGEMEIE---IYQRAQ---EIQEANKQLEEANKNLAR        52842360       PAS+SHELIX+HISKIN+REC+HISKIN                                                                                 816    Legionella pneumophila subsp. pneumophila str. Philadelphia 1        response regulator TutC [Legionella pneumophila subsp. pneumophila str. Philadelphia 1]
54294995 0.00031: _1_[151..190]    KLMEELRTKAGEMEIE---IYQRAQ---EIQEANKQLEEANKNLAR        54294995       PAS+SHELIX+HISKIN+REC+HISKIN                                                                                 816    Legionella pneumophila str. Lens                                     sensor histidine kinase [Legionella pneumophila str. Lens]                       
54298027 0.00031: _1_[151..190]    KLMEELRTKAGEMEIE---IYQRAQ---EIQEANKQLEEANKNLAR        54298027       PAS+SHELIX+HISKIN+REC+HISKIN                                                                                 816    Legionella pneumophila str. Paris                                    sensor histidine kinase [Legionella pneumophila str. Paris]                      
# 5             :                                                                        # 5
15601461 2.7e-08: _1_[504..543]    TDVTDQEHANRLLEQR---VKEKTQ---SLREKNRELQAEVEERQR        15601461       TM+HAMP+PAS+SHELIX+HISKIN                                                                                    789    Vibrio cholerae O1 biovar eltor str. N16961                          phosphoglycerate transport regulatory protein PgtB, putative [Vibrio cholerae O1 biovar eltor str. N16961]
27366664 2.3e-08: _1_[506..545]    TDVTEQIQANRILEQR---VAEKTR---DVTERNRQLKLEIKERQK        27366664       TM+TM+HAMP+TM+PAS+SHELIX+HISKIN                                                                              787    Vibrio vulnificus CMCP6                                              Signal transduction histidine kinase [Vibrio vulnificus CMCP6]                   
28900681 2.2e-06: _1_[509..548]    TDVTHQEMTTRTLESR---VLEKTQ---DLLLKNQQLENEIEERIR        28900681       TM+HAMP+PAS+SHELIX+HISKIN                                                                                    791    Vibrio parahaemolyticus RIMD 2210633                                 putative phosphoglycerate transport regulatory protein PgtB [Vibrio parahaemolyticus RIMD 2210633]
37676376 2.3e-08: _1_[506..545]    TDVTEQIQANRILEQR---VAEKTR---DVTERNRQLKLEIKERQK        37676376       TM+TM+HAMP+TM+PAS+SHELIX+HISKIN                                                                              787    Vibrio vulnificus YJ016                                              signal transduction histidine kinase regulating phosphoglycerate transport system [Vibrio vulnificus YJ016]
54309924 4.9e-06: _1_[283..322]    RIQNRLRDQEELIQTM---AEQATT---SLKQLNDETRHSITVSNF        54309924       TM+SHELIX+HISKIN+TM+HAMP+PAS+SHELIX+HISKIN                                                                786    Photobacterium profundum SS9                                         hypothetical phosphoglycerate transport regulatory protein PgtB [Photobacterium profundum SS9]
# 5             :                                                                        # 5
39995365 3.9e-14: _1_[368..407]    NAEDELSRLNAELEQR---VAQRTS---ELAALNRELEAFSYTVSH        39995365       TM+PAS+PAS+SHELIX+HISKIN                                                                                     616    Geobacter sulfurreducens PCA                                         sensory box histidine kinase [Geobacter sulfurreducens PCA]                      
39995799 7.5e-10: _1_[362..401]    AVEEEIRRLNADLEAR---VRERTA---DLESFCHSVSHDLRAPLR        39995799       PAS+PAS+PAS+SHELIX+HISKIN                                                                                    602    Geobacter sulfurreducens PCA                                         sensory box histidine kinase [Geobacter sulfurreducens PCA]                      
39997882 4.8e-13: _1_[513..552]    NAELGMRQLNEELERR---VGERTR---ELELSNRELDSFCSAVSH        39997882       PAS+PAS+PAS+PAS+SHELIX+HISKIN                                                                                770    Geobacter sulfurreducens PCA                                         sensory box histidine kinase [Geobacter sulfurreducens PCA]                      
39997908 2.4e-10: _1_[501..540]    QTEETIGKLNRELDRR---VMERTG---QLEEAIREQESFSYSVSH        39997908       PAS+PAS+PAS+PAS+SHELIX+HISKIN                                                                                749    Geobacter sulfurreducens PCA                                         sensory box histidine kinase [Geobacter sulfurreducens PCA]                      
56479336 3.5e-13: _1_[363..402]    IAEMTLRNINRELEAR---VRARTV---ELEAANRELKAFSYSVSH        56479336       TM+PAS+PAS+SHELIX+HISKIN                                                                                     611    Azoarcus sp. EbN1                                                    sensory box histidine kinase [Azoarcus sp. EbN1]                                 
# 5             :                                                                        # 5
13474773 7.4e-12: _1_[209..248]    AARRNLEALNSGLETR---VRERTA---DLVRANEEVQRFAYIVTH        13474773       TM+CHASE3+TM+SHELIX+HISKIN                                                                                   492    Mesorhizobium loti MAFF303099                                        two-component sensor protein [Mesorhizobium loti MAFF303099]                     
15889278 6.5e-12: _1_[215..254]    RSREDVGELNRHLEER---VEERTR---DLRRANQEIQRFAYIVTH        15889278       TM+CHASE3+TM+SHELIX+HISKIN                                                                                   497    Agrobacterium tumefaciens str. C58                                   hypothetical protein AGR_C_3614 [Agrobacterium tumefaciens str. C58]             
17935875 6.5e-12: _1_[202..241]    RSREDVGELNRHLEER---VEERTR---DLRRANQEIQRFAYIVTH        17935875       TM+CHASE3+TM+SHELIX+HISKIN                                                                                   484    Agrobacterium tumefaciens str. C58                                   two component sensor kinase [Agrobacterium tumefaciens str. C58]                 
27379400 3.7e-12: _1_[217..256]    EAEMRLRDANVNLETV---VDERTA---DLREANNEIQRFAYIVSH        27379400       TM+CHASE3+TM+SHELIX+HISKIN                                                                                   505    Bradyrhizobium japonicum USDA 110                                    two-component sensor histidine kinase [Bradyrhizobium japonicum USDA 110]        
39935500 1.4e-11: _1_[217..256]    EAENQLRENNLNLESA---VQERTA---DLREANEEIQRFAYIVSH        39935500       TM+CHASE3+TM+SHELIX+HISKIN                                                                                   510    Rhodopseudomonas palustris CGA009                                    sensor histidine kinase [Rhodopseudomonas palustris CGA009]                      
# 5             :                                                                        # 5
21232824    0.66: _1_[272..311]    RAADSFNAMQARLNRL---INERTH---MVAAIAHDLRTPLARLSF        21232824       TM+TM+HAMP+SHELIX+HISKIN                                                                                     498    Xanthomonas campestris pv. campestris str. ATCC 33913                two-component system sensor protein [Xanthomonas campestris pv. campestris str. ATCC 33913]
21241500    0.66: _1_[278..317]    RAADSFNAMQARLNRL---INERTH---MVAAIAHDLRTPLARLSF        21241500       TM+TM+HAMP+SHELIX+HISKIN                                                                                     504    Xanthomonas axonopodis pv. citri str. 306                            two-component system sensor protein [Xanthomonas axonopodis pv. citri str. 306]  
46199530     0.7: _1_[202..241]    AELRPVVAALNALFAR---VGRLLA---ELAEKEEAARRFARHASH        46199530       TM+TM+TM+HAMP+SHELIX+HISKIN                                                                                  434    Thermus thermophilus HB27                                            two-component system histidine kinase [Thermus thermophilus HB27]                
55981561     0.7: _1_[202..241]    AELRPVVAALNALFAR---VGRLLA---ELAEKEEAARRFARHASH        55981561       TM+TM+TM+HAMP+SHELIX+HISKIN                                                                                  434    Thermus thermophilus HB8                                             sensor histidine kinase [Thermus thermophilus HB8]                               
58583494    0.66: _1_[274..313]    RAADSFNAMQARLNRL---INERTH---MVAAIAHDLRTPLARLSF        58583494       TM+TM+HAMP+SHELIX+HISKIN                                                                                     499    Xanthomonas oryzae pv. oryzae KACC10331                              two-component system sensor protein [Xanthomonas oryzae pv. oryzae KACC10331]    
# 4             :                                                                        # 4
15642080  0.0015: _1_[758..797]    WRTRMILAANRKLKAQ---VELKTS---QLRHQSKIVLSNNHQLRK        15642080       TM+TM+SHELIX+arac                                                                                            1121   Vibrio cholerae O1 biovar eltor str. N16961                          transcriptional regulator, AraC/XylS family [Vibrio cholerae O1 biovar eltor str. N16961]
27363633   0.015: _1_[751..790]    WRSRYVSVQNRELTAL---VALKTN---QLRHQSRVLLTSNQQLRK        27363633       TM+SHELIX+HISKIN+arac                                                                                        1134   Vibrio vulnificus CMCP6                                              Signal transduction histidine kinase [Vibrio vulnificus CMCP6]                   
28897628  0.0035: _1_[758..797]    WRSRYVMQANRELAAQ---VTLKTN---QLRHQSRVLLTSNQQLRK        28897628       TM+TM+SHELIX+arac                                                                                            1130   Vibrio parahaemolyticus RIMD 2210633                                 putative transcriptional regulator, AraC/XylS family [Vibrio parahaemolyticus RIMD 2210633]
37679223   0.015: _1_[767..806]    WRSRYVSVQNRELTAL---VALKTN---QLRHQSRVLLTSNQQLRK        37679223       TM+TM+SHELIX+HISKIN+arac                                                                                     1150   Vibrio vulnificus YJ016                                              putative transcriptional regulator, AraC/XylS family [Vibrio vulnificus YJ016]   
# 4             :                                                                        # 4
24212767 1.5e-09: _1_[586..625]    QARRELQDYAENLEEK---VKERTQ---EVQEKMEEVQRLKVQQDG        24212767       TM+TM+TM+TM+TM+TM+TM+TM+HAMP+SHELIX+pp2cSIG                                                                  1061   Leptospira interrogans serovar Lai str. 56601                        hypothetical protein LA0067 [Leptospira interrogans serovar Lai str. 56601]      
24212770 4.2e-07: _1_[588..627]    DARKELQDYAEHLATK---VRLRTE---ELSEKIEEFQRLKIQQDG        24212770       TM+TM+TM+TM+TM+TM+TM+TM+TM+TM+HAMP+SHELIX+pp2cSIG                                                            1068   Leptospira interrogans serovar Lai str. 56601                        hypothetical protein LA0070 [Leptospira interrogans serovar Lai str. 56601]      
45655974 1.5e-09: _1_[586..625]    QARRELQDYAENLEEK---VKERTQ---EVQEKMEEVQRLKVQQDG        45655974       TM+TM+TM+TM+TM+TM+TM+TM+HAMP+SHELIX+pp2cSIG                                                                  1061   Leptospira interrogans serovar Copenhageni str. Fiocruz L1-130       hypothetical protein LIC10060 [Leptospira interrogans serovar Copenhageni str. Fiocruz L1-130]
45655977 4.2e-07: _1_[588..627]    DARKELQDYAEHLATK---VRLRTE---ELSEKIEEFQRLKIQQDG        45655977       TM+TM+TM+TM+TM+TM+TM+TM+TM+TM+HAMP+SHELIX+pp2cSIG                                                            1068   Leptospira interrogans serovar Copenhageni str. Fiocruz L1-130       histidine kinase sensor protein [Leptospira interrogans serovar Copenhageni str. Fiocruz L1-130]
# 4             :                                                                        # 4
24375806 2.2e-18: _1_[197..236]    TGVMERLKHQEQLEQA---IQQRTK---ELSQAYDKLKQEVYERRR        24375806       GAF+SHELIX+GAF+SHELIX+GGDEF+EAL                                                                           876    Shewanella oneidensis MR-1                                           GGDEF domain protein [Shewanella oneidensis MR-1]                                
28869887 4.9e-19: _1_[156..195]    AMALDRLLRRTDLEQI---VALRTQ---QLSAANDALRQEVKDRER        28869887       GAF+SHELIX+GAF+SHELIX+GGDEF+EAL                                                                           819    Pseudomonas syringae pv. tomato str. DC3000                          GAF domain/GGDEF domain/EAL domain protein [Pseudomonas syringae pv. tomato str. DC3000]
34499834   7e-21: _1_[277..316]    GFALDRVLYHRRLERQ---VALRTS---ELEGANARLRAEVASRKR        34499834       GAF+SHELIX+GAF+SHELIX+GGDEF+EAL                                                                           946    Chromobacterium violaceum ATCC 12472                                 hypothetical protein CV4379 [Chromobacterium violaceum ATCC 12472]               
56459317 1.1e-09: _1_[196..235]    VNALERLKQRDLMQSE---IEHQTA---ELRHANESLMSEITIRER        56459317       GAF+SHELIX+GAF+SHELIX+GGDEF+EAL                                                                           866    Idiomarina loihiensis L2TR                                           Intracellular signaling protein (GAF,GGDEF,EAL domains) [Idiomarina loihiensis L2TR]
# 4             :                                                                        # 4
17227678 2.1e-09: _1_[144..183]    KVEAELRKYQNHLEEL---VTIRTN---EITNANEKLQQQINERQH        17227678       TM+TM+TM+SHELIX+PAS+HISKIN+REC                                                                               715    Nostoc sp. PCC 7120                                                  two-component hybrid sensor and regulator [Nostoc sp. PCC 7120]                  
17230767    0.89: _1_[347..386]    QLNESLVAINDCLEES---HREK-D---ELLQREQALRSRLSNILS        17230767       (7TM)TM+TM+TM+TM+TM+TM+TM+SHELIX+PAS+HISKIN+REC                                                                   889    Nostoc sp. PCC 7120                                                  two-component hybrid sensor and regulator [Nostoc sp. PCC 7120]                  
37523781 3.5e-11: _1_[135..174]    RVEEALRQAKDELERR---VAERTD---DLSTANARLHGELAERRR        37523781       PAS+SHELIX+PAS+HISKIN+REC                                                                                    686    Gloeobacter violaceus PCC 7421                                       two-component hybrid sensor and regulator [Gloeobacter violaceus PCC 7421]       
54309013 0.00017: _1_[175..214]    ERAIIDIDYRERLQSL---VTARTQ---ELTHSQQRFKDFAKTVGD        54309013       SHELIX+PAS+HISKIN+REC                                                                                        693    Photobacterium profundum SS9                                         hypothetical protein PBPRA1827 [Photobacterium profundum SS9]                    
# 4             :                                                                        # 4
15600911 9.9e-11: _1_[428..467]    KAEIALQNANRDLQQR---VEERTA---ALKSAQQELIEASKMAAL        15600911       TM+CACHE+PAS+SHELIX+HISKIN                                                                                   684    Vibrio cholerae O1 biovar eltor str. N16961                          C4-dicarboxylate transport sensor protein, putative [Vibrio cholerae O1 biovar eltor str. N16961]
27367382 6.6e-10: _1_[426..465]    KAEQALQKANQELAER---VEERTA---ALRSAQQELIESSKLAAL        27367382       TM+TM+CACHE+TM+PAS+SHELIX+HISKIN                                                                             686    Vibrio vulnificus CMCP6                                              Signal transduction histidine kinase [Vibrio vulnificus CMCP6]                   
28900037 4.6e-10: _1_[431..470]    KAEQSLQLINQELAQR---VEERTQ---ALRQAQEQLIESSKMAAL        28900037       TM+CACHE+TM+PAS+SHELIX+HISKIN                                                                                690    Vibrio parahaemolyticus RIMD 2210633                                 putative C4-dicarboxylate transport sensor protein [Vibrio parahaemolyticus RIMD 2210633]
37677139 6.1e-10: _1_[566..605]    KAEQALQKANHELAER---VEERTA---ALRSAQQELIESSKLAAL        37677139       TM+TM+CACHE+TM+PAS+SHELIX+HISKIN                                                                             826    Vibrio vulnificus YJ016                                              signal transduction histidine kinase [Vibrio vulnificus YJ016]                   
# 4             :                                                                        # 4
16330789 1.9e-07: _1_[342..381]    LLYSEVQKQKNNFEKR---VIERTK---ELRDTLMAAQAANLLKSQ        16330789       GAF+SHELIX+HISKIN+REC                                                                                        750    Synechocystis sp. PCC 6803                                           hybrid sensory kinase [Synechocystis sp. PCC 6803]                               
17229180   1e-06: _1_[409..448]    DSAQRLTQEKQTLEQR---VIERTM---ALRDALLAAEAASRLRSE        17229180       GAF+SHELIX+HISKIN                                                                                            676    Nostoc sp. PCC 7120                                                  two-component sensor histidine kinase [Nostoc sp. PCC 7120]                      
22298442 1.8e-09: _1_[323..362]    QLYSELQQQKATLEQR---VNERTQ---ALREALSAMEAAHRIKND        22298442       GAF+REC+SHELIX+HISKIN+REC                                                                                    729    Thermosynechococcus elongatus BP-1                                   two-component sensor histidine kinase [Thermosynechococcus elongatus BP-1]       
56750891 3.3e-09: _1_[347..386]    QLYGQLQDQTQTLENR---VLERTQ---ELIDA-LALAQAANAAKG        56750891       GAF+SHELIX+HISKIN+REC                                                                                        754    Synechococcus elongatus PCC 6301                                     two-component sensor kinase [Synechococcus elongatus PCC 6301]                   
# 4             :                                                                        # 4
15600935 1.3e-08: _1_[498..537]    KAQEAISVLNETLELE---VRNRTR---DLQKANEKLDLMAKLDPL        15600935       GAF+GAF+SHELIX+GGDEF                                                                                         710    Vibrio cholerae O1 biovar eltor str. N16961                          GGDEF family protein [Vibrio cholerae O1 biovar eltor str. N16961]               
27367906 5.7e-12: _1_[492..531]    QAQEAIRELNENLEKE---VRKRTQ---ELEEANRQLEVLSKLDPL        27367906       GAF+GAF+SHELIX+GGDEF                                                                                         700    Vibrio vulnificus CMCP6                                              GGDEF family protein [Vibrio vulnificus CMCP6]                                   
28901312 2.4e-10: _1_[498..537]    QAQEAIRELNENLELE---VQNRTK---ELEEANRQLEVLSKLDPL        28901312       GAF+GAF+SHELIX+GGDEF                                                                                         710    Vibrio parahaemolyticus RIMD 2210633                                 GGDEF family protein [Vibrio parahaemolyticus RIMD 2210633]                      
37676033 5.7e-12: _1_[498..537]    QAQEAIRELNENLEKE---VRKRTQ---ELEEANRQLEVLSKLDPL        37676033       GAF+GAF+SHELIX+GGDEF                                                                                         706    Vibrio vulnificus YJ016                                              GGDEF family protein [Vibrio vulnificus YJ016]                                   
# 4             :                                                                        # 4
17230231    0.65: _1_[253..295]    MKNTELTNMNQRLELE---IAERKQAELELQKALQELQQTQAQLIQ        17230231       TM+TM+HAMP+SHELIX+HISKIN                                                                                     591    Nostoc sp. PCC 7120                                                  two-component sensor histidine kinase [Nostoc sp. PCC 7120]                      
24375382 2.1e-08: _1_[246..285]    RLLKLERSYKEELARQ---VKERTQ---ELEQSLAALKRAQQVLVE        24375382       TM+TM+HAMP+SHELIX+HISKIN                                                                                     543    Shewanella oneidensis MR-1                                           sensor histidine kinase [Shewanella oneidensis MR-1]                             
42525078 1.7e-10: _1_[232..271]    EANQKLESLNQALEQK---VAERTR---QLTTKNESLEKAMMQIKR        42525078       TM+HAMP+SHELIX+HISKIN                                                                                        515    Bdellovibrio bacteriovorus HD100                                     serine/threonine kinase with two-component sensor domain [Bdellovibrio bacteriovorus HD100]
56461220 3.6e-06: _1_[250..289]    AANRQLDEQNAILEQE---VARKTS---SLSQVMLDLEQRKNELEM        56461220       TM+TM+HAMP+SHELIX+HISKIN                                                                                     577    Idiomarina loihiensis L2TR                                           Signal transduction histidine kinase [Idiomarina loihiensis L2TR]                
# 4             :                                                                        # 4
34498714    0.62: _1_[258..297]    VDITARKQTQRQLETA---LQEKTT---LLNEVHHRVKNNLQVIIS        34498714       PAS+PAS+SHELIX+HISKIN                                                                                        484    Chromobacterium violaceum ATCC 12472                                 probable sensory transduction histidine kinase [Chromobacterium violaceum ATCC 12472]
37521102 2.4e-11: _1_[243..282]    EDISARKQNSEQLERR---VRERTV---QLEAANQELEAFTYSVSH        37521102       REC+PAS+SHELIX+HISKIN                                                                                        500    Gloeobacter violaceus PCC 7421                                       two-component hybrid sensor and regulator [Gloeobacter violaceus PCC 7421]       
56479140    0.68: _1_[234..273]    MSAIRDVSERRGFEQA---LQEKNL---ELLAANRAKDHFLASMSH        56479140       PAS+PAS+SHELIX+HISKIN                                                                                        491    Azoarcus sp. EbN1                                                    sensory transduction histidine kinase [Azoarcus sp. EbN1]                        
57234840 6.8e-07: _1_[108..147]    QDVTDPVNLRKQLQIQ---VAERTK---ELAEINSKLVQEIKNHEK        57234840       PAS+SHELIX+PAS+HISKIN                                                                                        534    Dehalococcoides ethenogenes 195                                      sensory box sensor histidine kinase [Dehalococcoides ethenogenes 195]            
# 3             :                                                                        # 3
21225602   0.058: _1_[716..758]    TQQEELQHSNAELEDKASLLAAQNR---DIEAKNLQIEQARQELEA        21225602       HAMP+HAMP+HAMP+HAMP+HAMP+HAMP+HAMP+GAF+SHELIX+HISKIN+REC+REC                                                 1331   Streptomyces coelicolor A3(2)                                        putative two-component system sensory histidine kinase. [Streptomyces coelicolor A3(2)]
29827626    0.17: _1_[808..850]    VQQDELQRSNAELEEKATLLATQNR---DIEAKNLQIEQARQELEA        29827626       HAMP+HAMP+HAMP+HAMP+HAMP+HAMP+HAMP+HAMP+GAF+SHELIX+HISKIN+REC                                                1424   Streptomyces avermitilis MA-4680                                     putative two-component system sensor kinase [Streptomyces avermitilis MA-4680]   
29832107    0.29: _1_[887..926]    SRTESLLGESQRLAIQ---LQDRSD---ELQSQQAELQRSNAELEE        29832107       HAMP+HAMP+HAMP+HAMP+HAMP+HAMP+HAMP+HAMP+HAMP+HAMP+HAMP+GAF+SHELIX+HISKIN+REC                                 1383   Streptomyces avermitilis MA-4680                                     putative two-component system sensor kinase [Streptomyces avermitilis MA-4680]   
# 3             :                                                                        # 3
15599307   6e-10: _1_[462..501]    AAEARIRELNTGLEHQ---IAERTA---ELRRLNVLLGSVLQAASE        15599307       TM+CHASE+PAS+SHELIX+PAS+PAS+HISKIN+REC+REC+HPT                                                               1417   Pseudomonas aeruginosa PAO1                                          probable sensor/response regulator hybrid [Pseudomonas aeruginosa PAO1]          
34496645   8e-06: _1_[85..123]     RLHERIRQMNASLESQ---VRQGTQ---EVRRYAA-LQRAILNDAS        34496645       SHELIX+PAS+PAS+HISKIN+REC+REC+HPT                                                                            1105   Chromobacterium violaceum ATCC 12472                                 probable sensor/response regulator hybrid [Chromobacterium violaceum ATCC 12472] 
34498958 6.6e-07: _1_[196..234]    ANERDIRKLNASLEQQ---VEQRTA---EI-RVFSALQNAILANAG        34498958       TM+PAS+SHELIX+PAS+PAS+HISKIN+REC+REC+HPT                                                                     1229   Chromobacterium violaceum ATCC 12472                                 probable sensor/response regulator hybrid [Chromobacterium violaceum ATCC 12472] 
# 3             :                                                                        # 3
21231797 4.4e-09: _1_[1240..1278]  LALPALAGASGDLESR---VEQGH----DLTAALLALKDTMAQTTA        21231797       TM+CHASE+TM+PAS+SHELIX+PAS+HISKIN+REC+REC+HPT+SHELIX                                                      1364   Xanthomonas campestris pv. campestris str. ATCC 33913                two-component system sensor protein [Xanthomonas campestris pv. campestris str. ATCC 33913]
21243225 1.3e-07: _1_[465..503]    RSHQRIIDLNASLEHQ---VAERTS---ELVKFSV-LQRAILAHAG        21243225       TM+PAS+CHASE+TM+PAS+SHELIX+PAS+HISKIN+REC+REC+HPT                                                            1364   Xanthomonas axonopodis pv. citri str. 306                            two-component system sensor protein [Xanthomonas axonopodis pv. citri str. 306]  
58582420 1.5e-06: _1_[503..541]    RSHQRSIDLNASLEHQ---VAERTG---ELVKFSV-LQRAILAHAG        58582420       TM+PAS+CHASE+TM+PAS+SHELIX+TM+PAS+HISKIN+REC+REC+HPT                                                         1402   Xanthomonas oryzae pv. oryzae KACC10331                              two-component system sensor protein [Xanthomonas oryzae pv. oryzae KACC10331]    
# 3             :                                                                        # 3
21231592   0.075: _1_[715..754]    LREQAMARSASHLAGI---VIERTR---VDAKLKASLQAETAARNQ        21231592       GAF+GAF+PAS+PAS+GAF+SHELIX+HISKIN+REC                                                                        1146   Xanthomonas campestris pv. campestris str. ATCC 33913                two-component system sensor protein [Xanthomonas campestris pv. campestris str. ATCC 33913]
21242798    0.23: _1_[700..739]    LREQAMARSASHLAGI---VIERTR---VDAKLKLSLQAETAARAQ        21242798       GAF+GAF+PAS+PAS+GAF+SHELIX+HISKIN+REC                                                                        1127   Xanthomonas axonopodis pv. citri str. 306                            two-component system sensor protein [Xanthomonas axonopodis pv. citri str. 306]  
58581946    0.23: _1_[719..758]    LREQAMARSASHLAGI---VIERTR---VDAKLKLSLQAETAARAQ        58581946       GAF+PAS+PAS+GAF+SHELIX+HISKIN+REC                                                                            1150   Xanthomonas oryzae pv. oryzae KACC10331                              two-component system sensor protein [Xanthomonas oryzae pv. oryzae KACC10331]    
# 3             :                                                                        # 3
17232370 3.1e-10: _1_[437..476]    DSFAELEKTNQELENR---VAERTA---EITAAKEVADAANRAKSE        17232370       TM+MCP-N+CACHE+TM+HAMP+SHELIX+HISKIN+REC                                                                     941    Nostoc sp. PCC 7120                                                  two-component hybrid sensor and regulator [Nostoc sp. PCC 7120]                  
27378360 1.2e-09: _1_[355..394]    RMAAHLRESYATLERK---VIERTS---ELEKARDHALAEHDAAER        27378360       TM+CACHE+HAMP+SHELIX+HISKIN+REC+HPT                                                                          897    Bradyrhizobium japonicum USDA 110                                    two-component hybrid sensor and regulator [Bradyrhizobium japonicum USDA 110]    
56752287 1.1e-09: _1_[394..433]    RMAVTLRELLVSLEHR---VQERTA---ALRETNDRLEQAREQAEA        56752287       CACHE+TM+HAMP+SHELIX+HISKIN+REC+REC                                                                          937    Synechococcus elongatus PCC 6301                                     two-component system sensory histidine kinase [Synechococcus elongatus PCC 6301] 
# 3             :                                                                        # 3
21232777 2.2e-10: _1_[480..519]    NAARELRSLADALEHR---VAERTR---DLAAAKREAESANRYKTR        21232777       TM+MCP-N+PAS+CACHE+PAS+SHELIX+HISKIN+REC                                                                     881    Xanthomonas campestris pv. campestris str. ATCC 33913                sensor histidine kinase [Xanthomonas campestris pv. campestris str. ATCC 33913]  
21244198 2.2e-10: _1_[479..518]    NAARELRSLADALEHR---VAERTR---DLAAAKREAESANRYKTR        21244198       TM+MCP-N+CACHE+PAS+SHELIX+HISKIN+REC                                                                         880    Xanthomonas axonopodis pv. citri str. 306                            sensor histidine kinase [Xanthomonas axonopodis pv. citri str. 306]              
58580738 1.7e-10: _1_[480..519]    NAARELRSLADALEHR---VAERTR---DLAEAKREAESANRYKTR        58580738       TM+MCP-N+CACHE+PAS+SHELIX+HISKIN+REC                                                                         881    Xanthomonas oryzae pv. oryzae KACC10331                              sensor histidine kinase [Xanthomonas oryzae pv. oryzae KACC10331]                
# 3             :                                                                        # 3
39995928 1.8e-14: _1_[482..521]    RAEEEILRLNSDLEQR---VADRTA---ALESANRELEAFSYTVSH        39995928       TM+CACHE+HAMP+PAS+SHELIX+HISKIN                                                                              729    Geobacter sulfurreducens PCA                                         sensory box histidine kinase [Geobacter sulfurreducens PCA]                      
39997395 1.2e-12: _1_[482..521]    GAEEEVRRLNAGLEQR---VAERTA---ELESVNIRLMAEIEQRAQ        39997395       TM+CACHE+HAMP+PAS+SHELIX+HISKIN                                                                              762    Geobacter sulfurreducens PCA                                         sensory box histidine kinase [Geobacter sulfurreducens PCA]                      
39997483 1.6e-16: _1_[310..349]    REIDELGDGFRSMADA---VKTRES---ELKERNEELAMTEEELRQ        39997483       CACHE+HAMP+SHELIX+PAS+SHELIX+HISKIN                                                                       733    Geobacter sulfurreducens PCA                                         sensory box histidine kinase [Geobacter sulfurreducens PCA]                      
# 3             :                                                                        # 3
15599594    0.08: _1_[217..256]    DEIGRLADAFNTMLTR---IEAREQ---QLKRARDDAQEAVEQAQS        15599594       TM+TM+HAMP+SHELIX+PAS+HISKIN+PAS                                                                             698    Pseudomonas aeruginosa PAO1                                          probable two-component sensor [Pseudomonas aeruginosa PAO1]                      
26988084   0.086: _1_[217..256]    DEIGSLAEAFNTMLSR---IEAREQ---QLKSARDEFQSAYDQAQG        26988084       TM+TM+HAMP+SHELIX+PAS+HISKIN                                                                                 676    Pseudomonas putida KT2440                                            sensory box histidine kinase/response regulator [Pseudomonas putida KT2440]      
28871530    0.16: _1_[217..256]    DEIGSLAEAFNTMLSR---IEAREQ---QLKRARDDSQEAYDQAQG        28871530       TM+TM+HAMP+SHELIX+PAS+HISKIN                                                                                 678    Pseudomonas syringae pv. tomato str. DC3000                          sensory box histidine kinase [Pseudomonas syringae pv. tomato str. DC3000]       
# 3             :                                                                        # 3
15600122 8.6e-06: _1_[455..494]    ARLALLHSAQRDLARA---VEVRTN---ELSEANRQLQMREAQLQY        15600122       TM+TM+TM+TM+TM+TM+TM+SHELIX+GGDEF                                                                            680    Pseudomonas aeruginosa PAO1                                          hypothetical protein PA4929 [Pseudomonas aeruginosa PAO1]                        
24375692 2.4e-07: _1_[413..452]    EALKVEAETNERLEQK---VQERTL---ELEITLRELHEVNQKLTE        24375692       TM+TM+TM+TM+TM+TM+TM+TM+SHELIX+GGDEF                                                                         625    Shewanella oneidensis MR-1                                           GGDEF domain protein [Shewanella oneidensis MR-1]                                
56461466   4e-07: _1_[388..427]    ERLQAEQRHNRELEES---VDERTF---ELQVALRELQDVNTELEQ        56461466       TM+TM+TM+TM+TM+TM+TM+TM+SHELIX+GGDEF                                                                         592    Idiomarina loihiensis L2TR                                           Signaling protein with a acyltransferase and GGDEF domains [Idiomarina loihiensis L2TR]
# 3             :                                                                        # 3
15678922    0.86: _1_[247..286]    QDITEDKRIRESLEAS---IREKEF---LLSEIHHRVKNNLQLISS        15678922       REC+PAS+SHELIX+HISKIN                                                                                        462    Methanothermobacter thermautotrophicus str. Delta H                  sensory transduction histidine kinase [Methanothermobacter thermautotrophicus str. Delta H]
20091104 4.9e-11: _1_[272..311]    QAVEELNKHREHLEEL---VKERTS---ELTKTNRQLFNEISVRKQ        20091104       PAS+GAF+SHELIX+PAS+GAF+HISKIN                                                                                680    Methanosarcina acetivorans C2A                                       sensory transduction histidine kinase [Methanosarcina acetivorans C2A]           
21228988 2.6e-11: _1_[265..304]    YAEEELHQYKEKLEEI---VKERTI---ELTRANEQLSMEISRRKK        21228988       PAS+GAF+SHELIX+PAS+GAF+HISKIN                                                                                668    Methanosarcina mazei Go1                                             hypothetical sensory transduction histidine kinase [Methanosarcina mazei Go1]    
# 3             :                                                                        # 3
42779508 3.1e-07: _1_[313..352]    APTFSNLLYMDQLESM---VEERTR---ELAVANEKVTSVIESITD        42779508       GAF+GAF+SHELIX+PAS+HISKIN                                                                                    676    Bacillus cereus ATCC 10987                                           sensor histidine kinase, putative [Bacillus cereus ATCC 10987]                   
49476786   5e-08: _1_[314..353]    APTFSNLLYMDQLESM---VEERTR---ELAAANEKVTSVIESITD        49476786       GAF+GAF+SHELIX+PAS+HISKIN                                                                                    677    Bacillus thuringiensis serovar konkukian str. 97-27                  sensor histidine kinase; possible sporulation kinase [Bacillus thuringiensis serovar konkukian str. 97-27]
52144913   5e-08: _1_[314..353]    APTFSNLLYMDQLESM---VEERTR---ELAAANEKVTSVIESITD        52144913       GAF+GAF+SHELIX+PAS+HISKIN                                                                                    677    Bacillus cereus E33L                                                 sensor histidine kinase (sporulation kinase), C-terminal region [Bacillus cereus E33L]
# 3             :                                                                        # 3
15595370 2.9e-11: _1_[379..418]    SMADQVRRHTAELEDK---VQERTQ---ALEEANREMAAAQKKIGD        15595370       TM+TM+HAMP+SHELIX+pp2cSIG                                                                                    663    Pseudomonas aeruginosa PAO1                                          hypothetical protein PA0172 [Pseudomonas aeruginosa PAO1]                        
39996651 5.1e-10: _1_[379..418]    AMSAMVLDHTTNLEAR---VRERTD---ELSAANRLLEDSQRRIME        39996651       TM+TM+TM+HAMP+SHELIX+pp2cSIG                                                                                 661    Geobacter sulfurreducens PCA                                         bacterial signal domain protein [Geobacter sulfurreducens PCA]                   
56477009 1.2e-08: _1_[374..413]    GMARQIDAHTSELESR---VTARTR---DLAQARDRIAATHRQIQD        56477009       TM+TM+TM+HAMP+SHELIX+pp2cSIG                                                                                 670    Azoarcus sp. EbN1                                                    hypothetical protein ebA2797 [Azoarcus sp. EbN1]                                 
# 3             :                                                                        # 3
15600980 1.6e-07: _1_[355..394]    LAQLSLEIMNQDLEQQ---VANRTQ---DLALSLTRLQETQTQLIE        15600980       TM+TM+PAS+SHELIX+HISKIN                                                                                      648    Vibrio cholerae O1 biovar eltor str. N16961                          sensory box sensor histidine kinase [Vibrio cholerae O1 biovar eltor str. N16961]
27366900 1.1e-09: _1_[371..410]    LTQIELQQLNQNLEHE---VAVRTL---DLKQSMEQLKATQEQLVE        27366900       TM+TM+PAS+SHELIX+HISKIN                                                                                      665    Vibrio vulnificus CMCP6                                              Signal transduction histidine kinase [Vibrio vulnificus CMCP6]                   
37676675 1.1e-09: _1_[371..410]    LTQIELQQLNQNLEHE---VAVRTL---DLKQSMEQLKATQEQLVE        37676675       TM+TM+PAS+SHELIX+HISKIN                                                                                      665    Vibrio vulnificus YJ016                                              signal transduction histidine kinase [Vibrio vulnificus YJ016]                   
# 3             :                                                                        # 3
21232865  0.0033: _1_[343..378]    VSQQRIQELNRQLEGK---VEQVS----EVNREL---EAFSYSVSH        21232865       TM+CHASE3+TM+PAS+SHELIX+HISKIN                                                                               603    Xanthomonas campestris pv. campestris str. ATCC 33913                two-component system sensor protein [Xanthomonas campestris pv. campestris str. ATCC 33913]
21241454   0.014: _1_[359..394]    TAQLRIHELNRQMEGK---VEQVS----EVNREL---EAFSYSVSH        21241454       TM+CHASE3+TM+PAS+SHELIX+HISKIN                                                                               619    Xanthomonas axonopodis pv. citri str. 306                            two-component system sensor protein [Xanthomonas axonopodis pv. citri str. 306]  
58583559  0.0098: _1_[359..394]    TAQMRIQELNRQMEGK---VEQVS----EVNREL---EAFSYSVSH        58583559       TM+CHASE3+TM+PAS+SHELIX+HISKIN                                                                               619    Xanthomonas oryzae pv. oryzae KACC10331                              two-component system sensor protein [Xanthomonas oryzae pv. oryzae KACC10331]    
# 3  7TM           :                                                                        # 3
16329416   2e-07: _1_[373..412]    IAQQSLSRAVKNLENE---VQKRTD---KLAAANKSLELANIELNK        16329416       TM+TM+TM+TM+TM+TM+TM+SHELIX+GGDEF                                                                            585    Synechocystis sp. PCC 6803                                           PleD gene product [Synechocystis sp. PCC 6803]                                   
34495501 2.2e-07: _1_[305..344]    RIKQELGRQQEQLEQQ---VRIRTL---ALEQSHAEVVALSRVDSV        34495501       TM+TM+TM+TM+TM+TM+TM+TM+SHELIX+GGDEF                                                                         514    Chromobacterium violaceum ATCC 12472                                 pleD gene product [Chromobacterium violaceum ATCC 12472]                         
53804085 7.4e-05: _1_[290..328]    AAEEKLRRMNETLERR---VARRTE---ELHYRATR-DGLTGMANR        53804085       TM+TM+TM+TM+TM+TM+TM+TM+SHELIX+GGDEF                                                                         472    Methylococcus capsulatus str. Bath                                   GGDEF domain protein [Methylococcus capsulatus str. Bath]                        
# 3             :                                                                        # 3
15601006 1.5e-05: _1_[276..315]    QAQQKERMSRQLIERE---VADRTQ---ELAQALQTLEKAKHQQKE        15601006       TM+HAMP+SHELIX+HISKIN                                                                                        549    Vibrio cholerae O1 biovar eltor str. N16961                          sensor histidine kinase [Vibrio cholerae O1 biovar eltor str. N16961]            
16125543 1.3e-06: _1_[253..292]    ARREQEAELRASLEVQ---VATRTE---ELEAALAELRQVEARRRQ        16125543       TM+HAMP+SHELIX+HISKIN                                                                                        524    Caulobacter crescentus CB15                                          sensor histidine kinase [Caulobacter crescentus CB15]                            
17549660 2.4e-05: _1_[277..316]    QHRNDADAARRRLEDA---VQARTG---ELRTAHDALQRIDQRRRQ        17549660       TM+HAMP+SHELIX+HISKIN                                                                                        551    Ralstonia solanacearum GMI1000                                       PROBABLE TWO-COMPONENT SENSOR KINASE TRANSCRIPTION REGULATOR PROTEIN [Ralstonia solanacearum GMI1000]
# 3             :                                                                        # 3
23502518 5.2e-06: _1_[219..258]    EGQSVLRSEKELLEQR---VRERTA---ELEQERSIAERERKRVEV        23502518       TM+CHASE3+TM+SHELIX+HISKIN                                                                                   476    Brucella suis 1330                                                   sensor histidine kinase [Brucella suis 1330]                                     
49474606 5.4e-07: _1_[237..276]    MYQLLLHSENIALEAR---VKERTQ---ELEKARNHAEKERQRVEM        49474606       TM+TM+SHELIX+HISKIN                                                                                          485    Bartonella quintana str. Toulouse                                    hypothetical protein BQ10950 [Bartonella quintana str. Toulouse]                 
49476048 9.9e-07: _1_[237..276]    MYQLLLHSENIALEAR---VKERTQ---ELERARNHAEKERQRVEM        49476048       TM+TM+SHELIX+HISKIN                                                                                          485    Bartonella henselae str. Houston-1                                   hypothetical protein BH13820 [Bartonella henselae str. Houston-1]                
# 3             :                                                                        # 3
21232536  0.0034: _1_[196..235]    RGATELLLTKPNLDEK---VLQRLQ---RIQRAEQQCSDLIGSLLL        21232536       TM+HAMP+SHELIX+HISKIN                                                                                        385    Xanthomonas campestris pv. campestris str. ATCC 33913                two-component system sensor protein [Xanthomonas campestris pv. campestris str. ATCC 33913]
21243975  0.0034: _1_[196..235]    RGATELLLTKPNLDEK---VLQRLQ---RIQRAEQQCSDLIGSLLL        21243975       TM+HAMP+SHELIX+HISKIN                                                                                        385    Xanthomonas axonopodis pv. citri str. 306                            two-component system sensor protein [Xanthomonas axonopodis pv. citri str. 306]  
58580830   0.004: _1_[261..300]    RGATELLLTRPNLDEK---VLQRLQ---RIQRAEQQCSDLIGSLLL        58580830       TM+TM+HAMP+SHELIX+HISKIN                                                                                     450    Xanthomonas oryzae pv. oryzae KACC10331                              two-component system sensor protein [Xanthomonas oryzae pv. oryzae KACC10331]    
# 3             :                                                                        # 3
15641382 0.00015: _1_[236..275]    ASLQSTTVTKDELQAE---VARKTA---KLERQKAELRFLSERDSL        15641382       TM+TM+HAMP+SHELIX+GGDEF                                                                                      443    Vibrio cholerae O1 biovar eltor str. N16961                          GGDEF family protein [Vibrio cholerae O1 biovar eltor str. N16961]               
27365636  0.0013: _1_[227..266]    TSLRQITVTREQLELE---VLRQTA---ELRAQKEQLVYLSERDPL        27365636       TM+TM+HAMP+SHELIX+GGDEF                                                                                      437    Vibrio vulnificus CMCP6                                              GGDEF family protein [Vibrio vulnificus CMCP6]                                   
37680214  0.0013: _1_[230..269]    TSLRQITVTREQLELE---VLRQTA---ELRAQKEQLVYLSERDPL        37680214       TM+TM+HAMP+SHELIX+GGDEF                                                                                      440    Vibrio vulnificus YJ016                                              GGDEF family protein [Vibrio vulnificus YJ016]                                   
# 3             :                                                                        # 3
16761322   0.031: _1_[406..444]    HTLGQLNMQRRQLEQE---VAERK----EIEADLRAMQDELIQTAK        16761322       TM+TM+HAMP+SHELIX+HISKIN                                                                                     668    Salmonella enterica subsp. enterica serovar Typhi str. CT18          phosphoglycerate transport system sensor protein PgtB [Salmonella enterica subsp. enterica serovar Typhi str. CT18]
16765723   0.031: _1_[406..444]    HTLGQLNMQRRQLEQE---VAERK----EIEADLRAMQDELIQTAK        16765723       TM+TM+HAMP+SHELIX+HISKIN                                                                                     668    Salmonella typhimurium LT2                                           phosphoglycerate transport system sensor protein [Salmonella typhimurium LT2]    
29140982   0.031: _1_[406..444]    HTLGQLNMQRRQLEQE---VAERK----EIEADLRAMQDELIQTAK        29140982       TM+TM+HAMP+SHELIX+HISKIN                                                                                     668    Salmonella enterica subsp. enterica serovar Typhi Ty2                TM+TM+HAMP+SHELIX+HISKIN+ phosphoglycerate transport system sensor protein PgtB [Salmonella enterica subsp. enterica serovar Typhi Ty2]
# 2             :                                                                        # 2
18310494    0.31: _1_[803..842]    IVIFLMWSYVTALEKT---VKKRAE---QLKKEAKEKEKLYIEKEK        18310494       TM+TM+SHELIX+HISKIN                                                                                          1093   Clostridium perfringens str. 13                                      two-component sensor histidine kinase/response regulator [Clostridium perfringens str. 13]
29345776 4.1e-07: _1_[831..870]    WRIRNLKRQKEYLHRT---VEERTH---ELEQQKHLLENQTDELSR        29345776       SHELIX+HISKIN+REC+arac                                                                                       1420   Bacteroides thetaiotaomicron VPI-5482                                two-component system sensor histidine kinase/response regulator, hybrid ('one component system') [Bacteroides thetaiotaomicron VPI-5482]
# 2             :                                                                        # 2
24215505   7e-07: _1_[832..871]    LLNAERKEAKEELEKA---VKVRTS---ELAEALENLQKTQEQLIH        24215505       PAS+PAS+PAS+PAS+PAS+GAF+PAS+SHELIX+HISKIN                                                                    1191   Leptospira interrogans serovar Lai str. 56601                        two-component hybrid sensor and regulator [Leptospira interrogans serovar Lai str. 56601]
45657104   7e-07: _1_[832..871]    LLNAERKEAKEELEKA---VKVRTS---ELAEALENLQKTQEQLIH        45657104       PAS+PAS+PAS+PAS+PAS+GAF+PAS+SHELIX+HISKIN                                                                    1191   Leptospira interrogans serovar Copenhageni str. Fiocruz L1-130       histidine kinase sensor protein [Leptospira interrogans serovar Copenhageni str. Fiocruz L1-130]
# 2             :                                                                        # 2
15640642    0.56: _1_[475..517]    EKISLIKSQNKELESNLLIIRKQND---ELQLANKLKDEFLATTSH        15640642       TM+TM+HAMP+SHELIX+HISKIN+REC+PAS                                                                             1177   Vibrio cholerae O1 biovar eltor str. N16961                          sensory box sensor histidine kinase/response regulator [Vibrio cholerae O1 biovar eltor str. N16961]
28899252    0.34: _1_[427..469]    EKIQTIKQQNEELESNIKLIQKQND---ELQLA-DKLKDEFLATTS        28899252       TM+TM+HAMP+SHELIX+HISKIN+REC+PAS                                                                             1128   Vibrio parahaemolyticus RIMD 2210633                                 sensory box sensor histidine kinase/response regulator [Vibrio parahaemolyticus RIMD 2210633]
# 2             :                                                                        # 2
24215906 9.8e-10: _1_[789..828]    EAEETILDLNANLERK---IDLRTE---ELRKSNSDLRDAVNNLEK        24215906       TM+TM+TM+TM+TM+TM+TM+CACHE+TM+HAMP+PAS+PAS+SHELIX+HISKIN                                                     1165   Leptospira interrogans serovar Lai str. 56601                        two-component hybrid sensor and regulator [Leptospira interrogans serovar Lai str. 56601]
45656811 9.8e-10: _1_[789..828]    EAEETILDLNANLERK---IDLRTE---ELRKSNSDLRDAVNNLEK        45656811       TM+TM+TM+TM+TM+TM+TM+CACHE+TM+HAMP+PAS+PAS+SHELIX+HISKIN                                                     1165   Leptospira interrogans serovar Copenhageni str. Fiocruz L1-130       histidine kinase sensor protein [Leptospira interrogans serovar Copenhageni str. Fiocruz L1-130]
# 2             :                                                                        # 2
20090837  0.0011: _1_[706..745]    DLIKGITESKGEVDER---VSALMK---ELQTKEEDLRAANEELET        20090837       SHELIX+SHELIX+PAS+PAS                                                                                     1052   Methanosarcina acetivorans C2A                                       hypothetical protein MA1989 [Methanosarcina acetivorans C2A]                     
20092349   0.032: _2_[772..814]    SINEELQSTNEELETSKEELQSTNE---ELVTVNAELQNKVTELSQ        20092349       SHELIX+SHELIX+PAS+PAS                                                                                     1060   Methanosarcina acetivorans C2A                                       conserved hypothetical chemotaxis protein [Methanosarcina acetivorans C2A]       
# 2             :                                                                        # 2
20089747   0.018: _1_[482..521]    QGMWQLIQRKQ-LEDA---LKAYSE---DLSKANDELRSLNRIKAE        20089747       PAS+GAF+PAS+GAF+SHELIX+HISKIN                                                                                749    Methanosarcina acetivorans C2A                                       sensory transduction histidine kinase [Methanosarcina acetivorans C2A]           
20093345    0.14: _1_[744..783]    QGMWQLIQRKQ-LEEA---LRTYSG---ELSRANEELRSVNMMKTE        20093345       PAS+PAS+GAF+PAS+PAS+GAF+SHELIX+HISKIN                                                                        998    Methanosarcina acetivorans C2A                                       sensory transduction histidine kinase [Methanosarcina acetivorans C2A]           
# 2             :                                                                        # 2
16330352 4.6e-09: _1_[592..631]    RIEAELEKLNAKLEQR---VLERTQ---ALAESQEQVNRVNEQLAQ        16330352       PAS+PAS+PAS+PAS+SHELIX+GAF+GGDEF                                                                             970    Synechocystis sp. PCC 6803                                           hypothetical protein sll1687 [Synechocystis sp. PCC 6803]                        
16331870   4e-08: _3_[563..601]    FKGEILPDIRQQLAKT---VAEQL----ALAIANLKLREQLEEQSL        16331870       PAS+PAS+SHELIX+PAS+SHELIX+GAF+SHELIX+GGDEF                                                             768    Synechocystis sp. PCC 6803                                           PleD-like protein [Synechocystis sp. PCC 6803]                                   
# 2             :                                                                        # 2
17228673 1.4e-10: _1_[418..457]    KANQQLQDYSRTLEEK---VRDRTQ---ELETAKIAADVANQAKSE        17228673       TM+CHASE2+TM+TM+SHELIX+HISKIN+REC                                                                            922    Nostoc sp. PCC 7120                                                  two-component hybrid sensor and regulator [Nostoc sp. PCC 7120]                  
17232801 4.9e-10: _1_[428..467]    IANSQLLDYSKTLEIK---VQERTH---ELLEAKQAADAANQAKSE        17232801       TM+CHASE2+TM+TM+SHELIX+HISKIN+REC                                                                            935    Nostoc sp. PCC 7120                                                  two-component hybrid sensor and regulator [Nostoc sp. PCC 7120]                  
# 2             :                                                                        # 2
46580484 2.8e-11: _1_[565..604]    EAELALQAMNVSLEAR---VSERTA---ELARANDALQKSMDELRA        46580484       TM+TM+PAS+PAS+SHELIX+HISKIN                                                                                  870    Desulfovibrio vulgaris subsp. vulgaris str. Hildenborough            sensory box histidine kinase [Desulfovibrio vulgaris subsp. vulgaris str. Hildenborough]
51244211 5.5e-09: _1_[492..531]    QLAGELEAYKFHLEEL---VDQKTA---DLQRFQQDLITAKDEAER        51244211       TM+TM+PAS+SHELIX+HISKIN+REC                                                                                  910    Desulfotalea psychrophila LSv54                                      two-component system sensory/regulatory protein (hybrid family) [Desulfotalea psychrophila LSv54]
# 2             :                                                                        # 2
24217302 2.4e-07: _1_[462..501]    ELLALKELQNTNLEFQ---VQSRTR---ELLKARDDAEFANKVKSQ        24217302       TM+TM+TM+TM+TM+TM+TM+TM+SHELIX+HISKIN+REC                                                                    869    Leptospira interrogans serovar Lai str. 56601                        two-component hybrid sensor and regulator [Leptospira interrogans serovar Lai str. 56601]
45655765 2.4e-07: _1_[434..473]    ELLALKELQNTNLEFQ---VQSRTR---ELLKARDDAEFANKVKSQ        45655765       TM+TM+TM+TM+TM+TM+TM+TM+SHELIX+HISKIN+REC                                                                    841    Leptospira interrogans serovar Copenhageni str. Fiocruz L1-130       histidine kinase sensor protein [Leptospira interrogans serovar Copenhageni str. Fiocruz L1-130]
# 2             :                                                                        # 2
46578955 1.8e-07: _1_[300..339]    QARQQLKDYASNLESM---VATRTA---DLQAEADARSADVGLFVE        46578955       TM+TM+SHELIX+GAF+PAS+HISKIN                                                                                  803    Desulfovibrio vulgaris subsp. vulgaris str. Hildenborough            sensor histidine kinase [Desulfovibrio vulgaris subsp. vulgaris str. Hildenborough]
46579358   1e-05: _1_[292..331]    EARNQLQAYADNLRDM---VDARTE---ALSQEAAERRADVSLFVQ        46579358       TM+TM+SHELIX+PAS+HISKIN                                                                                      810    Desulfovibrio vulgaris subsp. vulgaris str. Hildenborough            sensor histidine kinase [Desulfovibrio vulgaris subsp. vulgaris str. Hildenborough]
# 2             :                                                                        # 2
13475593 2.1e-11: _1_[499..538]    RMAEEISFHTENLEQL---VDERTK---ELGDANQEISALNEKLRD        13475593       TM+CACHE+TM+HAMP+SHELIX+pp2cSIG                                                                              788    Mesorhizobium loti MAFF303099                                        hypothetical protein mll6700 [Mesorhizobium loti MAFF303099]                     
16264180 7.7e-11: _1_[497..536]    RMAEEISFHTENLEQL---VEDRTK---EIEEANLQISALNQQLRS        16264180       TM+CACHE+TM+HAMP+SHELIX+pp2cSIG                                                                              786    Sinorhizobium meliloti 1021                                          putative regulatory protein [Sinorhizobium meliloti 1021]                        
# 2             :                                                                        # 2
16263912 2.5e-10: _2_[34..73]      GSAYSLFESAIALQAE---VWEKTK---DLEKALDTLDRASSELEV        16263912       SHELIX+PAS+PAS+SHELIX+HISKIN+REC                                                                          747    Sinorhizobium meliloti 1021                                          putative sensor histidine kinase protein [Sinorhizobium meliloti 1021]           
56696389 1.9e-16: _2_[38..77]      GAAYAQFERAALLEEE---VRNRTR---DLERALDLLNDSNARLAE        56696389       SHELIX+PAS+PAS+SHELIX+HISKIN+REC                                                                          737    Silicibacter pomeroyi DSS-3                                          sensor histidine kinase/response regulator [Silicibacter pomeroyi DSS-3]         
# 2             :                                                                        # 2
24215121 2.6e-06: _1_[306..345]    NILEELKVHREKLEVL---VSQRTE---EL-NLQKEMAEKANKAKS        24215121       PAS+GAF+SHELIX+HISKIN                                                                                        731    Leptospira interrogans serovar Lai str. 56601                        two-component hybrid sensor and regulator [Leptospira interrogans serovar Lai str. 56601]
45657401 2.6e-06: _1_[306..345]    NILEELKVHREKLEVL---VSQRTE---ELNLQKEMAEKANKAKSE        45657401       PAS+GAF+SHELIX+HISKIN                                                                                        731    Leptospira interrogans serovar Copenhageni str. Fiocruz L1-130       histidine kinase sensor protein [Leptospira interrogans serovar Copenhageni str. Fiocruz L1-130]
# 2  7TM           :                                                                        # 2
24212811 1.1e-09: _1_[300..339]    EVEDQLRISHQSLEEK---VEERTH---ELLRSNEILREEIQEKNE        24212811       TM+TM+TM+TM+TM+TM+TM+TM+TM+SHELIX+PAS+HISKIN                                                                 717    Leptospira interrogans serovar Lai str. 56601                        Sensory transduction histidine kinase [Leptospira interrogans serovar Lai str. 56601]
45656010 1.1e-09: _1_[300..339]    EVEDQLRISHQSLEEK---VEERTH---ELLRSNEILREEIQEKNE        45656010       TM+TM+TM+TM+TM+TM+TM+TM+TM+SHELIX+PAS+HISKIN                                                                 717    Leptospira interrogans serovar Copenhageni str. Fiocruz L1-130       histidine kinase sensor protein [Leptospira interrogans serovar Copenhageni str. Fiocruz L1-130]
# 2             :                                                                        # 2
24216056 9.8e-10: _1_[327..366]    DAEQEIRLVNEGLEEI---VAKRTE---ELRTVNQYLISKNEQVIR        24216056       TM+TM+TM+TM+TM+TM+TM+TM+TM+SHELIX+HISKIN                                                                     712    Leptospira interrogans serovar Lai str. 56601                        Sensory transduction histidine kinase [Leptospira interrogans serovar Lai str. 56601]
45656697 9.8e-10: _1_[327..366]    DAEQEIRLVNEGLEEI---VAKRTE---ELRTVNQYLISKNEQVIR        45656697       TM+TM+TM+TM+TM+TM+TM+TM+TM+SHELIX+HISKIN                                                                     712    Leptospira interrogans serovar Copenhageni str. Fiocruz L1-130       histidine kinase sensor protein [Leptospira interrogans serovar Copenhageni str. Fiocruz L1-130]
# 2             :                                                                        # 2
24216308  0.0037: _1_[407..446]    TSNLELFEMKEQLVQK---IEDRTR---VLNDNIIQINRELEIAQN        24216308       TM+TM+TM+TM+TM+TM+TM+SHELIX+pp2cSIG                                                                          684    Leptospira interrogans serovar Lai str. 56601                        Sigma factor sigB regulation protein rsbU [Leptospira interrogans serovar Lai str. 56601]
45656496  0.0037: _1_[407..446]    TSNLELFEMKEQLVQK---IEDRTR---VLNDNIIQINRELEIAQN        45656496       TM+TM+TM+TM+TM+TM+TM+SHELIX+pp2cSIG                                                                          684    Leptospira interrogans serovar Copenhageni str. Fiocruz L1-130       hypothetical protein LIC10598 [Leptospira interrogans serovar Copenhageni str. Fiocruz L1-130]
# 2             :                                                                        # 2
15615511  0.0002: _1_[270..309]    ILFFLGIRWNRQLQKE---VAKKTV---VLHELNRTLEKQIEETRN        15615511       TM+SHELIX+PAS+HISKIN                                                                                         667    Bacillus halodurans C-125                                            two-component sensor histidine kinase [Bacillus halodurans C-125]                
28211918  0.0005: _1_[289..328]    ILLLIFFTWNDILHKE---VQKKTK---ELEIFNKSLISKNSQIQE        28211918       TM+TM+SHELIX+PAS+HISKIN                                                                                      673    Clostridium tetani E88                                               sensory transduction protein kinase [Clostridium tetani E88]                     
# 2             :                                                                        # 2
53718346 6.3e-08: _1_[239..278]    ARTAFLRGMTDALEEL---VAKRTS---ELEGALRQYERTTHVLQR        53718346       TM+TM+TM+TM+TM+TM+TM+SHELIX+HISKIN                                                                           627    Burkholderia pseudomallei K96243                                     putative two-component sensor histidine kinase [Burkholderia pseudomallei K96243]
53724710 6.3e-08: _1_[239..278]    ARTAFLRGMTDALEEL---VAKRTS---ELEGALRQYERTTHVLQR        53724710       TM+TM+TM+TM+TM+TM+TM+SHELIX+HISKIN                                                                           627    Burkholderia mallei ATCC 23344                                       sensor histidine kinase [Burkholderia mallei ATCC 23344]                         
# 2             :                                                                        # 2
24212833 3.4e-10: _1_[244..283]    IFYERLIELTETLEEK---VKIRTQ---ELEETQSQLIMSEKMASL        24212833       TM+TM+GAF+SHELIX+HISKIN                                                                                      609    Leptospira interrogans serovar Lai str. 56601                        Sensory transduction histidine kinase [Leptospira interrogans serovar Lai str. 56601]
45656030 3.4e-10: _1_[244..283]    IFYERLIELTETLEEK---VKIRTQ---ELEETQSQLIMSEKMASL        45656030       TM+TM+GAF+SHELIX+HISKIN                                                                                      609    Leptospira interrogans serovar Copenhageni str. Fiocruz L1-130       histidine kinase sensor protein [Leptospira interrogans serovar Copenhageni str. Fiocruz L1-130]
# 2             :                                                                        # 2
39996250 2.2e-11: _1_[339..378]    VQTEEIRAINEDLERR---VAARTE---ELQAALKDMESFSYSISH        39996250       TM+HAMP+SHELIX+HISKIN                                                                                        604    Geobacter sulfurreducens PCA                                         sensor histidine kinase [Geobacter sulfurreducens PCA]                           
39997662 5.9e-12: _1_[333..372]    ASEETLTALNAELEER---VLQRTA---DLEAMNRELESFSYSVSH        39997662       MCP-N+CACHE+HAMP+SHELIX+HISKIN                                                                               583    Geobacter sulfurreducens PCA                                         sensor histidine kinase [Geobacter sulfurreducens PCA]                           
# 2             :                                                                        # 2
16126563    0.02: _1_[185..224]    RRQHDLAISHEQL------VVERLR---VLEAAERTARSKSDIVAT        16126563       TM+TM+TM+TM+TM+SHELIX+HISKIN+REC                                                                             597    Caulobacter crescentus CB15                                          sensor histidine kinase/response regulator [Caulobacter crescentus CB15]         
27365587    0.94: _1_[9..48]       RKLQRAIASRKEAERL---LEEKSL---ELYQSNQQLKLALKQLEL        27365587       SHELIX+HISKIN+REC                                                                                            571    Vibrio vulnificus CMCP6                                              Probable sensor/response regulator hybrid [Vibrio vulnificus CMCP6]              
# 2             :                                                                        # 2
56551815    0.18: _1_[392..431]    FLYDLISNQIRKLRLM---ITNKTL---ELQETNLRLKELANTDEL        56551815       TM+SHELIX+GGDEF                                                                                              585    Zymomonas mobilis subsp. mobilis ZM4                                 two-component response regulator [Zymomonas mobilis subsp. mobilis ZM4]          
56552261 6.5e-07: _1_[371..410]    FRTFLLERRTRRLQHL---VDDRTH---ELQQANKQLSELANTDVL        56552261       TM+SHELIX+GGDEF                                                                                              563    Zymomonas mobilis subsp. mobilis ZM4                                 hypothetical protein ZMO1365 [Zymomonas mobilis subsp. mobilis ZM4]              
# 2             :                                                                        # 2
51891870 2.5e-11: _1_[318..357]    AMSDELQALYRNLERK---VAERTR---QLQEAHADLEALHRSQTE        51891870       TM+TM+HAMP+SHELIX+HISKIN                                                                                     584    Symbiobacterium thermophilum IAM 14863                               two-component sensor histidine kinase [Symbiobacterium thermophilum IAM 14863]   
51892178 3.8e-14: _1_[262..301]    QMSDRLQELYAGLEQK---VAERTR---ELEEANRELERASRYKSE        51892178       TM+TM+HAMP+SHELIX+HISKIN                                                                                     521    Symbiobacterium thermophilum IAM 14863                               two-component sensor histidine kinase [Symbiobacterium thermophilum IAM 14863]   
# 2             :                                                                        # 2
27376141 2.4e-11: _1_[309..348]    ILYQQLQDANTQLEDR---VAQRTR---ALMQANRRLSAQWLRLQR        27376141       REC+GAF+SHELIX+HISKIN                                                                                        581    Bradyrhizobium japonicum USDA 110                                    two-component hybrid sensor and regulator [Bradyrhizobium japonicum USDA 110]    
39937848 7.3e-11: _1_[278..317]    ILYRQLHEANTQLEER---VAQRTR---ALTQANRRLSTQWLRLQR        39937848       GAF+SHELIX+HISKIN                                                                                            550    Rhodopseudomonas palustris CGA009                                    response regulator receiver:histidine kinase [Rhodopseudomonas palustris CGA009] 
# 2             :                                                                        # 2
56476468 9.8e-05: _1_[134..173]    RLEDELKADVEKLEIL---VQQRTV---ELSRHVASLKQEVVTRKR        56476468       PAS+SHELIX+PAS+HISKIN                                                                                        544    Azoarcus sp. EbN1                                                    two-component sensor [Azoarcus sp. EbN1]                                         
56476750 9.3e-06: _1_[138..177]    LAEDQLRRSKERLAQT---VERRTV---NLQRTNRALKDEVRRRKD        56476750       PAS+SHELIX+PAS+HISKIN                                                                                        568    Azoarcus sp. EbN1                                                    two-component sensor [Azoarcus sp. EbN1]                                         
# 2             :                                                                        # 2
37523001   0.064: _1_[315..353]    QMGIALAQAQL-LEQE---TQQRR----ELARSNTDLEQFAYVASH        37523001       PAS+GAF+SHELIX+HISKIN                                                                                        566    Gloeobacter violaceus PCC 7421                                       two-component sensor histidine kinase [Gloeobacter violaceus PCC 7421]           
39998526 1.3e-11: _1_[280..319]    SVAMENVRVYADLEQR---VRERTE---ALETANRELEAFGFSVSH        39998526       PAS+GAF+SHELIX+HISKIN                                                                                        533    Geobacter sulfurreducens PCA                                         sensory box histidine kinase [Geobacter sulfurreducens PCA]                      
# 2             :                                                                        # 2
27366135   2e-06: _1_[341..380]    LMANEVNRGRKELEFR---VLERTK---ELKSA-ERFARHLASHDS        27366135       TM+CACHE+TM+HAMP+SHELIX+GGDEF                                                                                540    Vibrio vulnificus CMCP6                                              GGDEF domain [Vibrio vulnificus CMCP6]                                           
37679601   2e-06: _1_[343..382]    LMANEVNRGRKELEFR---VLERTK---ELKSA-ERFARHLASHDS        37679601       TM+CACHE+TM+HAMP+SHELIX+GGDEF                                                                                542    Vibrio vulnificus YJ016                                              GGDEF family protein [Vibrio vulnificus YJ016]                                   
# 2             :                                                                        # 2
39995987 2.2e-13: _1_[272..311]    ESERKIRELNVNLEEM---VRRRTA---ELMAANRELESFNYSASH        39995987       TM+TM+HAMP+SHELIX+HISKIN                                                                                     524    Geobacter sulfurreducens PCA                                         sensor histidine kinase [Geobacter sulfurreducens PCA]                           
39997386 6.5e-14: _1_[269..308]    KAQEAISALNNELEQR---VIRRTA---ELEKANREMESFNYSVSH        39997386       TM+TM+HAMP+SHELIX+HISKIN                                                                                     520    Geobacter sulfurreducens PCA                                         sensor histidine kinase [Geobacter sulfurreducens PCA]                           
# 2             :                                                                        # 2
27363777   0.017: _1_[269..307]    TYFEHRQQLNRELEGL---VAKSQA---SLDNA-TELEQLKNNISP        27363777       SHELIX+GGDEF                                                                                                 521    Vibrio vulnificus CMCP6                                              GGDEF domain family protein [Vibrio vulnificus CMCP6]                            
37679069   0.017: _1_[269..307]    TYFEHRQQLNRELEGL---VAKSQA---SLDNA-TELEQLKNNISP        37679069       SHELIX+GGDEF                                                                                                 521    Vibrio vulnificus YJ016                                              GGDEF family protein [Vibrio vulnificus YJ016]                                   
# 2             :                                                                        # 2
27377709 4.1e-10: _1_[128..167]    RKTRELERLNHDLEDR---VRARTA---ELENSTAKLRESEERRSM        27377709       REC+SHELIX+PAS                                                                                               496    Bradyrhizobium japonicum USDA 110                                    two-component response regulator [Bradyrhizobium japonicum USDA 110]             
27383150   0.019: _1_[230..269]    RMTARNAATKEELAET---LSGRLH---ALAQANGLIRRSFGSDVA        27383150       GAF+SHELIX+HISKIN                                                                                            400    Bradyrhizobium japonicum USDA 110                                    hypothetical protein blr8039 [Bradyrhizobium japonicum USDA 110]                 
# 2             :                                                                        # 2
24374675   9e-10: _1_[169..208]    DYQQQLKAINADLEAQ---VELRTQ---ELETALSDLRSTQHHLIE        24374675       GAF+SHELIX+HISKIN                                                                                            453    Shewanella oneidensis MR-1                                           sensor histidine kinase [Shewanella oneidensis MR-1]                             
56459713 1.3e-08: _1_[170..209]    RFEALLIQQNEQLESM---VEERTQ---SLTKALDNFKQAQERLIE        56459713       GAF+SHELIX+HISKIN                                                                                            455    Idiomarina loihiensis L2TR                                           Signal transduction histidine kinase (contains GAF domain) [Idiomarina loihiensis L2TR]
# 2             :                                                                        # 2
24215248 0.00092: _1_[146..185]    KMDREKEELLEKLEEK---VRQRSR---LLANYYKGMKEELNLAKK        24215248       PAS+SHELIX+pp2cSIG                                                                                           418    Leptospira interrogans serovar Lai str. 56601                        two-component response regulator [Leptospira interrogans serovar Lai str. 56601] 
45657302 0.00092: _1_[146..185]    KMDREKEELLEKLEEK---VRQRSR---LLANYYKGMKEELNLAKK        45657302       PAS+SHELIX+pp2cSIG                                                                                           418    Leptospira interrogans serovar Copenhageni str. Fiocruz L1-130       sigma factor regulatory protein [Leptospira interrogans serovar Copenhageni str. Fiocruz L1-130]
# 2             :                                                                        # 2
15595535 7.4e-09: _1_[179..218]    QRNEELFDWNLRLEEL---VAERTE---ALHRVNQALASKMAEAQR        15595535       PAS+SHELIX+GGDEF                                                                                             376    Pseudomonas aeruginosa PAO1                                          hypothetical protein PA0338 [Pseudomonas aeruginosa PAO1]                        
26991094 3.2e-10: _1_[135..174]    RLVSQLEQKNQSLEFQ---VAERTR---ELSWVNQQLQRQLDENRE        26991094       PAS+SHELIX+GGDEF                                                                                             332    Pseudomonas putida KT2440                                            sensory box protein [Pseudomonas putida KT2440]                                  
# 2             :                                                                        # 2
34495694 0.00013: _1_[175..214]    TSRNKSEQYARTLEEK---VATRTR---QLEEL-SRTDPLTGLLNR        34495694       SHELIX+GGDEF                                                                                                 375    Chromobacterium violaceum ATCC 12472                                 hypothetical protein CV0239 [Chromobacterium violaceum ATCC 12472]               
51244053  0.0077: _1_[176..215]    RSKRELEEYAESLEEV---ISERTK---LLKEQ-ARHDGLTKLLNQ        51244053       SHELIX+GGDEF                                                                                                 363    Desulfotalea psychrophila LSv54                                      hypothetical protein DP0201 [Desulfotalea psychrophila LSv54]                    
# 2             :                                                                        # 2
16126093  0.0011: _1_[143..182]    EATRRVHKENQSLEAR---LADSTA---EVERLREHLEQVRRDATT        16126093       SHELIX+GGDEF                                                                                                 353    Caulobacter crescentus CB15                                          GGDEF family protein [Caulobacter crescentus CB15]                               
51245642   0.011: _1_[119..161]    GVFLELYQHKEELEKKSIELDNRLC---ELEELQQQLEETNEQLTL        51245642       REC+SHELIX+GGDEF                                                                                             339    Desulfotalea psychrophila LSv54                                      similar to two-component system response regulator (hybrid family) [Desulfotalea psychrophila LSv54]
# 4 GAF         :                                                                        # 4 GAF + AAA atpase + HTH
15605774  0.0032: _1_[149..188]    SMIATLIANSFSLERK---VQAERK---SLEEEKRALETELKRVYE        15605774       GAF+SHELIX+AAAatpase+fis                                                                                     506    Aquifex aeolicus VF5                                                 transcriptional regulator (NifA family) [Aquifex aeolicus VF5]                   
17549180   0.098: _1_[138..175]    DAQAELRQLAVVIE-----AAARTT---RLEAEIRALRATRGDLPP        17549180       GAF+SHELIX+AAAatpase+fis                                                                                     510    Ralstonia solanacearum GMI1000                                       anaerobic nitric oxide reductase transcription regulator [Ralstonia solanacearum GMI1000]
46562252   0.051: _1_[155..194]    EVVATLVAQFLSLNEQ---VAARER---ALRRENMQLRTRVLDSRG        46562252       GAF+SHELIX+AAAatpase+fis                                                                                     515    Desulfovibrio vulgaris subsp. vulgaris str. Hildenborough            Nif-specific regulatory protein [Desulfovibrio vulgaris subsp. vulgaris str. Hildenborough]
46579093  0.0012: _1_[122..161]    ALVSENATLRSDMEGR---VAQATT---ALRQDLEAARLRLRNVSG        46579093       REC+SHELIX+AAAatpase+fis                                                                                     488    Desulfovibrio vulgaris subsp. vulgaris str. Hildenborough            sigma-54 dependent transcriptional regulator/response regulator [Desulfovibrio vulgaris subsp. vulgaris str. Hildenborough]
# 2 has         :                                                                        # 2 has RT. hth+linker?+inactive ggdef?
52081741   0.049: _1_[108..147]    EDVRLGDIVNHTLSHI---LDKRTA---ELEQAIAAQKKFTSHIMS        52081741       SHELIX                                                                                                       532    Bacillus licheniformis ATCC 14580                                    transcriptional regulator [Bacillus licheniformis ATCC 14580]                    
52787128   0.049: _1_[108..147]    EDVRLGDIVNHTLSHI---LDKRTA---ELEQAIAAQKKFTSHIMS        52787128       SHELIX                                                                                                       532    Bacillus licheniformis ATCC 14580                                    PucR [Bacillus licheniformis ATCC 14580]                                         
# 2 HTH+        :                                                                        # 2 HTH+Linker_divergent div family3 _ other members
53719128   0.054: _1_[96..134]     DIHADLSQQIATLDAR---IAAAQR---ELKEV-RALKQELQHDID        53719128       merr+SHELIX                                                                                                  166    Burkholderia pseudomallei K96243                                     putative MerR-family transcriptional regulator [Burkholderia pseudomallei K96243]
53723585   0.054: _1_[96..134]     DIHADLSQQIATLDAR---IAAAQR---ELKEV-RALKQELQHDID        53723585       merr+SHELIX                                                                                                  166    Burkholderia mallei ATCC 23344                                       transcriptional regulator, MerR family [Burkholderia mallei ATCC 23344]          
53723585                           DIHADLSQQIATLDAR---IAAAQR---ELKEV-RALKQELQHDIDYVERRLAGENADELIAQRRAA
53719128                           DIHADLSQQIATLDAR---IAAAQR---ELKEV-RALKQELQHDIDYVERRLAGENADELIAQRRAA
15807297                           -LESDLSSQLTVLSTR---ITAAEK---ELKDA-RALRRDVQRDLDYVQRRLAGAQVGELLREKDPA
# 2 PA20  merR1      :                                                                        # 2 PA2016 big family HTH+divergen linker. Real
15597212   0.018: _1_[76..114]     -PSSGNQKQLNTMLEK---IAERRA---QLEQQLLDIEQMQLELDT        15597212       merr+SHELIX                                                                                                  134    Pseudomonas aeruginosa PAO1                                          probable transcriptional regulator [Pseudomonas aeruginosa PAO1]                 
17987091   0.075: _1_[73..112]     -ELPGETGQLKLLMKR---VEEKRA---DLRQKRRDIDETLGELDQ        17987091       merr+SHELIX                                                                                                  128    Brucella melitensis 16M                                              TRANSCRIPTIONAL REGULATOR, MERR FAMILY [Brucella melitensis 16M]                 
23502057    0.44: _1_[73..112]     -REPPGETGQLKLLMKR--VEEKRA---DLRQKRRDIDETLGELDQ        23502057       merr+SHELIX                                                                                                  128    Brucella suis 1330                                                   transcriptional regulator, putative [Brucella suis 1330]                         
26990251   0.036: _1_[75..113]     -PTSGNLKQLNSMLAK---IAERRA---QLEQQMLDIHQMQLELDT        26990251       merr+SHELIX                                                                                                  132    Pseudomonas putida KT2440                                            transcriptional regulator, putative [Pseudomonas putida KT2440]                  
15597212                           -PSSGNQKQLNTMLEK---IAERRA---QLEQQLLDIEQMQLELDTAEERCRAALIKSYNKHTVET
28869930                           -PSGGNQKQLQTMLGK---ITERRG---QLEQQLLDIQQMQLELDTAEERCRAELDKTA-------
26990251                           -PTSGNLKQLNSMLAK---IAERRA---QLEQQMLDIHQMQLELDTAQERCEQALAATLNNK----
15888867                           ----GESGQLVLLMKK---VDEKRA---DLRQKRKDIEETLAELDNVEEACLTRLAEIGVG-----
17935445                           ----GESGQLVLLMKK---VDEKRA---DLRQKRKDIEETLAELDNVEEACLTRLAEIGVG-----
15965157                           ----GEMGQLQLLIKR---VEEKRE---DLRQKRKDIEDTLSELDNVEEACLTRLAEIGVG-----
23502057                           ----GETGQLKLLMKR---VEEKRA---DLRQKRRDIDETLGELDQVEEACIERLAEL--------
17987091                           ----GETGQLKLLMKR---VEEKRA---DLRQKRRDIDETLGELDQVEEACIERLAEL--------
13471368                           ----GEVGQLKLMIKR---IEEKRE---DLRQKRRDLEETLAELDQAEESCVERLVEL--------
49475400                           -----DEKKLKELIAG---VNKKRA---DLQQMQRDIDDFLHDLERIEETLFESLAEL--------
24373463                           -D-KSSTTQLNTMLAL---VEEKKS---ALQQQMDDIKVVLMELNSAEQQCRLALEESKVKA----
56459981                           -D-NSSAAQLEKVLEL---VSEKKH---SLKQQMEDINVLLTELSGLEQRCQDELENIQSS-----
27366929                           ----QSETQLVKMLNI---IDEKQA---ILQRQLDDISVVMSELDAAKQRCQHALHELASRSK---
37676705                           ----QSETQLVKMLNI---IDEKQA---ILQRQLDDISVVMSELDAAKQRCQHALHELASRSK---
28900474                           ---QQTDEQLLKMLNI---IEEKQA---VLQQQLNDIGVVMGELNAAKERC---LQTLKNNEK---
54302792                           -T-NQSDNQLNQMLTI---IDDKQV---TLQRQLNDINVVMSELNAARKRCHQALNRSKTS-----
28900979                           -T-DQSTEQLNYMIRL---IEEKKA---ALQQQANDIQAVMMELNAAQLRCQNTLRSMKGEK----
54308322                           -N-KSNKKQQQDMLNA---IKHRQQ---LLIQQQADIDAVMMELNAAERNCYLQLAKFEQ------
33603918                           ---GDTAPQLRHYLTI---LAQHRA---TLEQQQRDIEDTLAEIAQQELQCQRLLAQKQ-------
33591658                           ---GDTAPQLRHYLTI---LAQHRA---TLTQQQRDIEDTLAEIAQQELQCQRLLAQKQ-------
33598843                           ---GDTAPQLRHYLTI---LAQHRA---TLEQQQRDIEDTLAEISQQELQCQRLLAQKQ-------
17544999                           ----DTVPQLERLLTF---LAQHRA---VLTQQLEDLNAQLAEIDQHETQCRHLLDEARGGA----
56477489                           ----NPAAQLVRFLVV---LADRRE---ALEQQREDIEAVLGEIAMLEKQCSDLLGHDSRGA----
34497542                           ----DDAPQLQEFIRI---LARKEQ---QLLAQMEDIKVVLTEIGQLRGQCEKSLGKRANGH----
16124336                           -N-DEGAAQMARSLKK---FRERAT---ALEQQRDDIDNALIELREACNRLERRLTEIR-------
13474681                           -PTGSNTKQLRLALDK---SEKQLA---RLQKQRALIDDAINELSGSMSAVRQMLAER--------


# 2 misc        :                                                                        # 2 misc hth+linker. not a family
15641402    0.01: _1_[70..109]     KEGEQLFHSYAELFAQ---IERKQE---EVEQAKRDYRGEIAISAP        15641402       lysr+SHELIX+PBPII                                                                                            316    Vibrio cholerae O1 biovar eltor str. N16961                          lysR+Linker+lysr_substratebinding transcriptional regulator, LysR family [Vibrio cholerae O1 biovar eltor str. N16961]
18466612    0.05: _1_[76..115]     ADGAQAAAQLAVLRQL---VERRRA---ALAHLDAQLASMPAERAH        18466612       merr+SHELIX                                                                                                  120    Salmonella enterica subsp. enterica serovar Typhi str. CT18          Real. MerD _ other members hth+linker putative mercuric resistance operon coregulator [Salmonella enterica subsp. enterica serovar Typhi str. CT18]
21223398   0.033: _1_[87..126]     ESGEDVAGILERLEET---LAEREA---ELRRQRAAVRRMRTEGSR        21223398       merr+SHELIX                                                                                                  305    Streptomyces coelicolor A3(2)                                        Real. but only one instance putative transcriptional regulator [Streptomyces coelicolor A3(2)]
22536610    0.66: _1_[79..118]     ARLEILKDERDNLEDR---LQGLQE---ALNRLNHKIDNYQNKVVP        22536610       merr+SHELIX                                                                                                  129    Streptococcus agalactiae 2603V/R                                     SoxR div huge family1.hth+linker transcriptional regulator, MerR family [Streptococcus agalactiae 2603V/R]
28901418    0.71: _1_[81..120]     TTLQQKVQELDELERK---IAQSKS---KLKEVLKEIEAKPDDMDC        28901418       merr+SHELIX                                                                                                  154    Vibrio parahaemolyticus RIMD 2210633                                 div family1 (email) _ other members putative transcriptional regulator [Vibrio parahaemolyticus RIMD 2210633]
41409941  0.0024: _1_[76..115]     KRIIELTNQVEALQAR---VKELTE---ELAQVRAGQRRDLAVLPK        41409941       merr+SHELIX                                                                                                  131    Mycobacterium avium subsp. paratuberculosis K-10                     HspR div family1 _ 3 other members hth+linker[Mycobacterium avium subsp. paratuberculosis K-10]
54027344     0.3: _1_[77..116]     ELRGMLMLRRAELEQR---IAADRA---RLAQIETRLRIIEREGVM        54027344       merr+SHELIX                                                                                                  272    Nocardia farcinica IFM 10152                                         div family1 _ other members hth+linker+arac_subsbinding *putative transcriptional regulator [Nocardia farcinica IFM 10152] HTH+Linker?+AraC_E_bind    
# 1             :                                                                        # 1
27382127   1e-10: _1_[341..380]    RGEKRLRRAQVELERK---VAERTQ---ELELANAAKSRFLAMASH        27382127       (7TM) TM+TM+TM+TM+TM+TM+TM+SHELIX+HISKIN+REC                                                                       743    Bradyrhizobium japonicum USDA 110                                    two-component hybrid sensor and regulator [Bradyrhizobium japonicum USDA 110]    
42524257  0.0003: _1_[459..498]    TLLLTIANRIITVEEI---VDEKTQ---HLIDLNVQLKKASETKSE        42524257       (7TM)TM+TM+TM+TM+TM+TM+CHASE+TM+SHELIX+HISKIN+REC                                                                 878    Bdellovibrio bacteriovorus HD100                                     sensor histidine kinase/response regulator [Bdellovibrio bacteriovorus HD100]    
16330325 8.2e-10: _1_[490..529]    QLYQRVQTLNTNLEKQ---VGERTA---QLEDKMVELQDLQQMKAL        16330325       (7TM)TM+TM+TM+TM+TM+TM+TM+TM+GAF+SHELIX+HISKIN                                                                    749    Synechocystis sp. PCC 6803                                           sensory transduction histidine kinase [Synechocystis sp. PCC 6803]               
46581041   5e-09: _1_[315..354]    RLMGNLEAHTSRLEDR---VQERTL---ELTRLNAAYREEIDMRRE        46581041       CACHE+CACHE+HAMP+SHELIX+PAS                                                                                  487    Desulfovibrio vulgaris subsp. vulgaris str. Hildenborough            HAMP domain protein [Desulfovibrio vulgaris subsp. vulgaris str. Hildenborough]  
24372170 5.1e-05: _1_[375..414]    QYTHHLAEQNKRLDKL---VARRTE---RLKDATERREREYALLRS        24372170       CACHE+SHELIX+PAS+PAS+HISKIN+REC+REC                                                                          1188   Shewanella oneidensis MR-1                                           sensory box histidine kinase/response regulator [Shewanella oneidensis MR-1]     
54301706   1e-07: _1_[493..532]    KNALALREHQNELQKL---VDERTT---QLTEANNALNSEVAKHAK        54301706       CACHE+TM+HAMP+HAMP+SHELIX+HISKIN+REC+HPT                                                                     1035   Photobacterium profundum SS9                                         hypothetical sensor protein TorS [Photobacterium profundum SS9]                  
27378217 1.9e-11: _1_[362..401]    ADQAVIAIENARLFNE---VQQRTN---ELTEALEQQTATAEVLSV        27378217       GAF+GAF+SHELIX+GAF+GAF+SHELIX+HISKIN                                                                      1024   Bradyrhizobium japonicum USDA 110                                    two-component sensor histidine kinase [Bradyrhizobium japonicum USDA 110]        
17230191 7.4e-09: _1_[420..459]    ELYQQVQAFNENLEKQ---VQKRTL---ELRHTSEQQQAVFGVISK        17230191       GAF+GAF+SHELIX+GAF+HISKIN                                                                                    920    Nostoc sp. PCC 7120                                                  two-component sensor histidine kinase [Nostoc sp. PCC 7120]                      
17228348    0.27: _1_[400..439]    DERTAKLQSSLELQAK---LHERTR---QYVEQLRELNQLKDEFMS        17228348       GAF+GAF+SHELIX+HISKIN                                                                                        674    Nostoc sp. PCC 7120                                                  two-component sensor histidine kinase [Nostoc sp. PCC 7120]                      
24375463 2.6e-09: _1_[146..185]    FQQAKLQLHNQQLEQK---VQQRTH---ELAELSAKLIREIETRTT        24375463       GAF+SHELIX                                                                                                   259    Shewanella oneidensis MR-1                                           GAF domain protein [Shewanella oneidensis MR-1]                                  
39997479 1.9e-16: _1_[162..200]    ALIFRNAVLYRDLERQ---VVSRTH---DL-LWRAEVDRALAELYP        39997479       GAF+SHELIX+GAF+SHELIX+HISKIN                                                                              614    Geobacter sulfurreducens PCA                                         sensor histidine kinase [Geobacter sulfurreducens PCA]                           
54309750 5.3e-12: _1_[337..376]    RAEESLRNLNKELEQR---VTERTA---ELELVNQKLESLSKLDPL        54309750       GAF+SHELIX+GGDEF                                                                                             540    Photobacterium profundum SS9                                         hypothetical protein PBPRA2589 [Photobacterium profundum SS9]                    
42523608 1.1e-10: _1_[164..203]    TAEDSLKRINESLERL---VNERTS---ELQKAFASLQATQKQLIS        42523608       GAF+SHELIX+HISKIN                                                                                            315    Bdellovibrio bacteriovorus HD100                                     two-component hybrid sensor and regulator [Bdellovibrio bacteriovorus HD100]     
17228686 4.3e-07: _1_[169..208]    RLYKQQCQFNHILEAE---VEKRTA---ELKATQSKLLEKERLAVM        17228686       GAF+SHELIX+HISKIN                                                                                            435    Nostoc sp. PCC 7120                                                  two-component sensor histidine kinase [Nostoc sp. PCC 7120]                      
20090728 2.7e-12: _1_[189..228]    YAEEALKEAYQSLEDR---VRERTA---ELEKAYNSLKESEESLAE        20090728       GAF+SHELIX+PAS+HISKIN                                                                                        562    Methanosarcina acetivorans C2A                                       sensory transduction histidine kinase [Methanosarcina acetivorans C2A]           
16332192 4.6e-11: _1_[215..254]    ELYEQLQQLNKDLENR---VEKRTQ---QLAATNQSLRMEISERQK        16332192       GAF+SHELIX+PAS+PAS                                                                                           481    Synechocystis sp. PCC 6803                                           GAF+SHELIX+PAS+PAS+ putative chromatic adaptation sensor receptor protein [Synechocystis sp. PCC 6803]
53803910 1.8e-09: _1_[105..144]    VFVAELARSNRLLEQR---VEERTR---ALSEKTQELQRECDIRRA        53803910       GAF+SHELIX+PAS+PAS+GAF+HDGYP                                                                                 771    Methylococcus capsulatus str. Bath                                   sensory box protein [Methylococcus capsulatus str. Bath]                         
17229629 1.8e-07: _1_[324..363]    RLYERLSDYSETLERK---VEEQTQ---ALQQEIAERRQTEAALRQ        17229629       GAF+SHELIX+PAS+PAS+PAS+PAS+HISKIN                                                                            944    Nostoc sp. PCC 7120                                                  two-component sensor histidine kinase [Nostoc sp. PCC 7120]                      
22298758 1.3e-10: _1_[464..503]    ELYQQLAALNANLEQQ---IAERTG---QLQQKMSELEELNRLKDL        22298758       GAF+PAS+GAF+SHELIX+HISKIN                                                                                    726    Thermosynechococcus elongatus BP-1                                   two-component sensor histidine kinase [Thermosynechococcus elongatus BP-1]       
34496565 2.9e-11: _1_[320..359]    EAEAELQLLNSHLEER---VAARTA---ELKKAMQQISIAEKQAAL        34496565       GAF+PAS+SHELIX+HISKIN                                                                                        597    Chromobacterium violaceum ATCC 12472                                 hypothetical protein CV1110 [Chromobacterium violaceum ATCC 12472]               
37519602 3.6e-08: _1_[257..296]    VTQEELARLNAELEER---VRERTV---RYKLLNQKLRAEIGERQR        37519602       GAF+PAS+SHELIX+HISKIN+REC                                                                                    692    Gloeobacter violaceus PCC 7421                                       two-component hybrid sensor and regulator [Gloeobacter violaceus PCC 7421]       
27377710 0.00081: _1_[1369..1411]  TQQRELQQTNDQLEQKAQQLAERNV---EVERKNQEIEQARRALEE        27377710       HAMP+HAMP+HAMP+HAMP+HAMP+HAMP+HAMP+HAMP+HAMP+HAMP+HAMP+HAMP+HAMP+GAF+SHELIX+HISKIN+REC+REC+REC               2095   Bradyrhizobium japonicum USDA 110                                    two-component hybrid sensor and regulator [Bradyrhizobium japonicum USDA 110]    
56752288    0.04: _1_[334..373]    DALGQLARMFRQMTRS---VEQRET---QLKQTIRRLELQIDTATV        56752288       HISKIN+HISKIN+REC+HAMP+SHELIX                                                                                411    Synechococcus elongatus PCC 6301                                     two-component response regulator [Synechococcus elongatus PCC 6301]              
30249232    0.69: _1_[1228..1268]  MAVDQQAHLNRRLQQDL--VHIRTV---PFRHYSERLYRVVRQAAK        30249232       HPT+HPT+SHELIX+HISKIN+REC                                                                                    1713   Nitrosomonas europaea ATCC 19718                                     two-component hybrid sensor and regulator [Nitrosomonas europaea ATCC 19718]     
56476385   0.066: _1_[937..976]    GLEHALERLRDTAQAP---TAEQTA---LLKSANDTLDAMLAEVVA        56476385       HPT+HPT+SHELIX+HPT+HISKIN+REC                                                                                1782   Azoarcus sp. EbN1                                                    pili chemotaxis protein similar PilL [Azoarcus sp. EbN1]                         
39997311   0.094: _1_[52..91]      GVAAALAVENRKLAAR---VEELER---EIAEIIGQIRQIEAENND        39997311       SHELIX+GAF                                                                                                   274    Geobacter sulfurreducens PCA                                         GAF domain protein [Geobacter sulfurreducens PCA]                                
54024863    0.68: _1_[19..58]      VIETLAQSRLRELLAE---VQDRIA---EIVNVRDQMDRLIEAMLV        54024863       SHELIX+GAF+GAF+HISKIN                                                                                        583    Nocardia farcinica IFM 10152                                         putative two-component system sensor kinase [Nocardia farcinica IFM 10152]       
17230612 9.9e-14: _1_[320..358]    EKVQLLENRTVELEQL---VADRTA---SL-KAKAGREQIVSDISR        17230612       SHELIX+GAF+SHELIX+PAS+GAF+HISKIN+REC                                                                      1286   Nostoc sp. PCC 7120                                                  two-component hybrid sensor and regulator [Nostoc sp. PCC 7120]                  
42524787 1.2e-06: _1_[129..168]    EQTAQLKRLQIELEER---VQKRTK---FLTEARRKLFLTNSRIEG        42524787       SHELIX+GAF+PAS+HISKIN                                                                                        612    Bdellovibrio bacteriovorus HD100                                     Two-component sensor histidine kinase [Bdellovibrio bacteriovorus HD100]         
24375887 3.7e-08: _1_[139..178]    YPMTSLEERNRELESI---IQARTY---ELAEANNRLEKLSNTDGL        24375887       SHELIX+GGDEF                                                                                                 344    Shewanella oneidensis MR-1                                           GGDEF family protein [Shewanella oneidensis MR-1]                                
54023296   0.036: _1_[114..153]    LQERALAQQEQNLSAK---VSRRVQ---EIAALQHRLRHEATHDAL        54023296       SHELIX+GGDEF                                                                                                 318    Nocardia farcinica IFM 10152                                         hypothetical protein nfa13290 [Nocardia farcinica IFM 10152]                     
34557608   0.046: _1_[274..313]    EITQKIDTHIQNLNKE---VAEKKS---EIEFLQSKIEELTQELCR        34557608       SHELIX+GGDEF                                                                                                 480    Wolinella succinogenes DSM 1740                                      hypothetical protein WS1243 [Wolinella succinogenes DSM 1740]                    
58580556  0.0033: _1_[10..49]      WRAASYRRRHRQLNTV---TDRRTR---ELSDKNLALQQASQEREA        58580556       SHELIX+GGDEF                                                                                                 210    Xanthomonas oryzae pv. oryzae KACC10331                              hypothetical protein XOO0933 [Xanthomonas oryzae pv. oryzae KACC10331]           
15640161   0.032: _1_[390..429]    HAFHDLDAMTDNLAQQ---IRAQTS---LLTASYRRDRRTGLPNRV        15640161       SHELIX+GGDEF+EAL                                                                                             829    Vibrio cholerae O1 biovar eltor str. N16961                          GGDEF family protein [Vibrio cholerae O1 biovar eltor str. N16961]               
51893828     0.4: _1_[80..122]     LLRAQLLSYAEDLNREYQLVRERTV---ALERLLVATVGALANSIE        51893828       SHELIX+HDGYP                                                                                                 309    Symbiobacterium thermophilum IAM 14863                               two-component response regulator variant [Symbiobacterium thermophilum IAM 14863]
56477165   0.013: _1_[342..381]    VLLSAWILYTVRVEHL---VHLRTA---ELHAALAVREALEARMRA        56477165       SHELIX+HISKIN                                                                                                638    Azoarcus sp. EbN1                                                    sensor histidine kinase [Azoarcus sp. EbN1]                                      
17986653 5.2e-06: _1_[3..42]       EGQSVLRSEKELLEQR---VRERTA---ELEQERSIAERERKRVEV        17986653       SHELIX+HISKIN                                                                                                264    Brucella melitensis 16M                                              SENSORY TRANSDUCTION HISTIDINE KINASE [Brucella melitensis 16M]                  
37521628   0.013: _1_[312..351]    EKMELLQGRTLQLERL---VEERTA---QVQEQARREQLARAAAES        37521628       SHELIX+HISKIN                                                                                                682    Gloeobacter violaceus PCC 7421                                       two-component sensor histidine kinase [Gloeobacter violaceus PCC 7421]           
26990126     0.3: _1_[545..584]    LLLAGVVFWNSYLRKL---INQRTE---AQHALQAQLALSRGLLEQ        26990126       SHELIX+HISKIN+REC+HPT                                                                                        1093   Pseudomonas putida KT2440                                            sensor histidine kinase/response regulator [Pseudomonas putida KT2440]           
17229871 8.2e-15: _1_[275..314]    LDPLEIVNVIQALEAK---VAEQTF---QLQQTNQQLEAEIKQRHQ        17229871       SHELIX+SHELIX+PAS+PAS+PAS+PAS+PAS+HISKIN+REC                                                              1344   Nostoc sp. PCC 7120                                                  two-component hybrid sensor and regulator [Nostoc sp. PCC 7120]                  
55377058    0.44: _1_[111..150]    ANLLARRSHTEDLAEN---LREQNN---ELRRFRNAVEHAGHAILI        55377058       SHELIX+PAS+HISKIN                                                                                            465    Haloarcula marismortui ATCC 43049                                    signal-transducing histidine kinase-like [Haloarcula marismortui ATCC 43049]     
20092182 5.4e-24: _1_[1..39]       -MITSKSVAADELESR---LKGRIA---ELEKSNGELRARLLEYKH        20092182       SHELIX+PAS+SHELIX+PAS+MEDS+PAS+PAS+SHELIX+PAS+PAS+PAS+PAS+HISKIN                                       1447   Methanosarcina acetivorans C2A                                       sensory transduction histidine kinase [Methanosarcina acetivorans C2A]           
21244455    0.72: _1_[467..516]    SMIEELESTNEELKSSN--EEVTTVNG-ELAHRVQELAHANSDLKN        21244455       SHELIX+PAS+PAS                                                                                               950    Xanthomonas axonopodis pv. citri str. 306                            SHELIX+PAS+PAS+ methyltransferase [Xanthomonas axonopodis pv. citri str. 306] 
53804418    0.27: _1_[628..670]    QTIEKSRHVNDELRASNEEIMAMNE---ELQSANEELESSREELQS        53804418       SHELIX+PAS+PAS+GGDEF+EAL                                                                                     1378   Methylococcus capsulatus str. Bath                                   SHELIX+PAS+PAS+GGDEF+EAL methyltransferase CheR, putative [Methylococcus capsulatus str. Bath]
56478485 7.2e-06: _1_[195..234]    FQPLNIKDQIDGLETK---VFERTR---ELEASEQRYRNLIEDLPE        56478485       SHELIX+PAS+PAS+HISKIN                                                                                        712    Azoarcus sp. EbN1                                                    hybrid sensor component of two-component regulation system [Azoarcus sp. EbN1]   
16331882 0.00049: _1_[150..189]    RLYERLANYAKTLERK---LEEQTQ---ALQQEIINRERTEDALRQ        16331882       SHELIX+PAS+PAS+PAS+HISKIN                                                                                    759    Synechocystis sp. PCC 6803                                           sensory transduction histidine kinase [Synechocystis sp. PCC 6803]               
15597172   2e-18: _1_[456..495]    RTAEALHQAYQNLEQR---VRERTA---ELTTLNDQFKREIHERSQ        15597172       SHELIX+PAS+PAS+PAS+SHELIX+HISKIN+REC                                                                      881    Pseudomonas aeruginosa PAO1                                          probable two-component sensor [Pseudomonas aeruginosa PAO1]                      
56475524     0.6: _1_[28..70]      SANEELKSVNEDLYILNRELEDRNA---ALANLNRDYDHLLASTKI        56475524       SHELIX+PAS+PAS+PAS+PAS+GGDEF+EAL                                                                             1008   Azoarcus sp. EbN1                                                    sensory box/GGDEF family protein [Azoarcus sp. EbN1]                             
56697870  0.0028: _1_[226..265]    RSHIELRQAQTALEKL---LARRAD---RLEKAEQLLTGICADIGQ        56697870       lysr+REC+SHELIX                                                                                              269    Silicibacter pomeroyi DSS-3                                          REC+SHELIX+ DNA-binding response regulator, LysR family [Silicibacter pomeroyi DSS-3]
21226991 1.1e-11: _1_[22..61]      LCQFELKESYDHLEEL---VQERTV---QLEKAYKRLKESEIDLSE        21226991       MEDS+SHELIX+PAS+PAS+HISKIN                                                                                   527    Methanosarcina mazei Go1                                             hypothetical protein MM0889 [Methanosarcina mazei Go1]                           
21228379 2.6e-15: _2_[743..782]    KTETELKETLDNLENL---VKGRTE---ELEKAYSSLKESEKRLAE        21228379       MEDS+SHELIX+PAS+PAS+PAS+PAS+SHELIX+PAS+PAS+HISKIN                                                         1259   Methanosarcina mazei Go1                                             hypothetical sensory transduction histidine kinase [Methanosarcina mazei Go1]    
20090134   5e-09: _3_[686..725]    EAEIELKEARDNLKKL---VEKRTL---KLRKAYKLLKESEKGLAE        20090134       MEDS+PAS+GAF+PAS+SHELIX+PAS+SHELIX+PAS+PAS+PAS+PAS+SHELIX+HISKIN                                       1456   Methanosarcina acetivorans C2A                                       sensory transduction histidine kinase [Methanosarcina acetivorans C2A]           
20089101 2.3e-10: _1_[639..678]    EAEAKLKETLDNLENL---VKERTA---QLEKAYNLLKENEKGLAE        20089101       MEDS+PAS+GAF+PAS+PAS+SHELIX+PAS+PAS+PAS+HISKIN                                                               1274   Methanosarcina acetivorans C2A                                       signal-transducing histidine kinase [Methanosarcina acetivorans C2A]             
20089440 1.2e-10: _1_[874..913]    KAEEALKEAHDNLEKL---IEERTM---QLEKAYNSLKESEEGLAE        20089440       MEDS+PAS+GAF+PAS+PAS+PAS+PAS+SHELIX+PAS+PAS+HISKIN                                                           1349   Methanosarcina acetivorans C2A                                       sensory transduction histidine kinase [Methanosarcina acetivorans C2A]           
20090927 2.1e-11: _1_[322..361]    RAEEALKKAHECLEEK---VKARTV---ELEEAYKALKENEGRLSE        20090927       MEDS+PAS+SHELIX+PAS+HISKIN                                                                                   698    Methanosarcina acetivorans C2A                                       sensory transduction histidine kinase [Methanosarcina acetivorans C2A]           
20090138 2.1e-10: _1_[711..750]    KTEAKLKDTLDNLENM---VKKRTS---ELEKAYKLLKESERGLAE        20090138       MEDS+PAS+PAS+PAS+PAS+SHELIX+PAS+PAS+PAS+HISKIN                                                               1355   Methanosarcina acetivorans C2A                                       sensory transduction histidine kinase [Methanosarcina acetivorans C2A]           
20090183 3.9e-12: _1_[799..838]    KAEEALKKAHENLEEK---VKERTA---ELEEAYKSLMEEERRLSE        20090183       MEDS+PAS+PAS+PocR+PAS+SHELIX+PAS+PAS+PAS+HISKIN                                                              1428   Methanosarcina acetivorans C2A                                       sensory transduction histidine kinase [Methanosarcina acetivorans C2A]           
20089643 1.7e-12: _1_[525..564]    RAEEALQKAYDNLEEK---VKERTA---ELEEACKALVENERRLSE        20089643       MEDS+PocR+PAS+SHELIX+PAS+PAS+HISKIN                                                                          1016   Methanosarcina acetivorans C2A                                       sensory transduction histidine kinase [Methanosarcina acetivorans C2A]           
13476679 1.2e-06: _1_[976..1015]   RAEAALQTLNSTLEQR---VIDEVA---ERSKAEEQLRQVQKMDAV        13476679       PAS+GAF+GAF+PAS+GAF+PAS+GAF+PAS+SHELIX+PAS+HISKIN+REC                                                        1386   Mesorhizobium loti MAFF303099                                        two-component sensor histidine kinase [Mesorhizobium loti MAFF303099]            
39936994   9e-11: _1_[892..931]    KAEAALRDINEQLEEQ---VAERTA---ELQQKEARLRTIFAASYT        39936994       PAS+GAF+HISKIN+REC+PAS+REC+SHELIX+PAS                                                                        1021   Rhodopseudomonas palustris CGA009                                    sensor histidine kinase with multiple PAS and a response regulator receiver domain [Rhodopseudomonas palustris CGA009]
22299374 1.6e-11: _1_[451..490]    QLYQQVQELNADLERQ---VRARTA---ELEQKMQELERLNAIKDD        22299374       PAS+GAF+SHELIX+HISKIN                                                                                        714    Thermosynechococcus elongatus BP-1                                   two-component sensor histidine kinase [Thermosynechococcus elongatus BP-1]       
15596440 2.6e-05: _1_[458..497]    RAENELRELNESLEER---VATMLA---QRESALAQLHEARKMEMV        15596440       PAS+GAF+PAS+SHELIX+HISKIN+REC                                                                                858    Pseudomonas aeruginosa PAO1                                          probable sensor/response regulator hybrid [Pseudomonas aeruginosa PAO1]          
39995363 3.5e-20: _2_[569..608]    LAEDELHRHRELLEEL---VHQRTA---ELKLRNMELASEIAERRR        39995363       PAS+GAF+PAS+PAS+SHELIX+PAS+SHELIX+HISKIN                                                                  830    Geobacter sulfurreducens PCA                                         sensory box histidine kinase [Geobacter sulfurreducens PCA]                      
17231056   3e-11: _1_[162..201]    LTYQELADLNTNLEHQ---VEERTA---ELQQKMRELEEIQRIKNV        17231056       PAS+SHELIX+HISKIN                                                                                            461    Nostoc sp. PCC 7120                                                  hypothetical protein all3564 [Nostoc sp. PCC 7120]                               
16124540 1.8e-08: _1_[222..261]    VVELRARDHSDGLQQM---VEERTR---ELTEALKQKTALLHEVDH        16124540       PAS+SHELIX+HISKIN                                                                                            449    Caulobacter crescentus CB15                                          sensory box histidine kinase [Caulobacter crescentus CB15]                       
41409331    0.01: _1_[472..514]    TMNEELQSTNDELHTINDMLRERSL---ELDDAKSFLDSLVDSVRM        41409331       PAS+SHELIX+PAS                                                                                               616    Mycobacterium avium subsp. paratuberculosis K-10                     hypothetical protein MAP3233c [Mycobacterium avium subsp. paratuberculosis K-10] 
42522900 2.6e-09: _1_[127..166]    DIERKLEEAKSLLEER---VAERTR---QLVESESFLMAIFENMPT        42522900       PAS+SHELIX+PAS+GAF+HISKIN+REC                                                                                865    Bdellovibrio bacteriovorus HD100                                     two-component hybrid sensor and regulator [Bdellovibrio bacteriovorus HD100]     
56477556 4.9e-11: _1_[144..183]    RYEDVIQRQNAELEAR---VKARTA---ELENANARLAQAHGEVDE        56477556       PAS+SHELIX+PAS+HISKIN                                                                                        408    Azoarcus sp. EbN1                                                    sensory box histidine kinase/response regulator,N-terminal part [Azoarcus sp. EbN1]
42523850 5.1e-10: _1_[137..176]    EAKVQLENYSKNLEQM---VADRTR---ELSRLNTTMSALLDSLGQ        42523850       PAS+SHELIX+PAS+HPT+HISKIN                                                                                    649    Bdellovibrio bacteriovorus HD100                                     chemotaxis histidine kinase [Bdellovibrio bacteriovorus HD100]                   
39995214 5.8e-18: _1_[289..328]    LAEDELQHLVAEMEDR---IRSRTA---ELLDANTRLLEEIEERRR        39995214       PAS+SHELIX+PAS+SHELIX+HISKIN                                                                              703    Geobacter sulfurreducens PCA                                         sensory box histidine kinase [Geobacter sulfurreducens PCA]                      
55377301   0.036: _1_[388..427]    ASTGAAALNRVERETR---LKSKQA---ELERSNEALQQFAYIASH        55377301       PAS+PAS+GAF+SHELIX+HISKIN                                                                                    643    Haloarcula marismortui ATCC 43049                                    sensory transduction histidine kinase [Haloarcula marismortui ATCC 43049]        
21226270   7e-12: _1_[954..993]    KAEEALKKAYDNLDIL---VKERTA---ELEKAYTSLKESEKGLAE        21226270       PAS+PAS+GAF+PAS+PAS+GAF+PAS+SHELIX+PAS+PAS+PAS+HISKIN                                                        1584   Methanosarcina mazei Go1                                             hypothetical sensory transduction histidine kinase [Methanosarcina mazei Go1]    
20089847 4.2e-10: _1_[565..604]    ESELKFRTLSNTLEEK---VKERTV---ELEKAYKFLQEIDIIRKQ        20089847       PAS+PAS+GAF+PAS+PAS+SHELIX+HISKIN                                                                            830    Methanosarcina acetivorans C2A                                       sensory transduction histidine kinase [Methanosarcina acetivorans C2A]           
20090499   6e-11: _1_[552..591]    NTEDALKIANETLEEK---VKERTV---ELEKAYSTLKEKEELLSN        20090499       PAS+PAS+GAF+PAS+PAS+SHELIX+PAS+PAS+HISKIN                                                                    1058   Methanosarcina acetivorans C2A                                       sensory transduction histidine kinase [Methanosarcina acetivorans C2A]           
21233187  0.0093: _1_[269..310]    ALTTALTDISEHLRVQWR-LTKTTE---ALAEANRQLEQANAQLLG        21233187       PAS+PAS+SHELIX+GGDEF                                                                                         483    Xanthomonas campestris pv. campestris str. ATCC 33913                GGDEF family protein [Xanthomonas campestris pv. campestris str. ATCC 33913]     
34557352 4.7e-09: _1_[494..533]    TDVTELEMSKRELEGK---VMARTA---ELSRAMSILEEAQKIARL        34557352       PAS+PAS+SHELIX+PAS+GGDEF                                                                                     804    Wolinella succinogenes DSM 1740                                      hypothetical protein WS0964 [Wolinella succinogenes DSM 1740]                    
16329637 1.9e-06: _1_[274..313]    KSRDEIRQINQELEER---VKLRTK---EVYEQQQLLRLYFDQSII        16329637       PAS+PAS+SHELIX+PAS+HISKIN                                                                                    661    Synechocystis sp. PCC 6803                                           sensory transduction histidine kinase [Synechocystis sp. PCC 6803]               
15807408 4.5e-07: _1_[347..386]    AAERALQTLNADLQLQ---VAEQTQ---EIGQVSRFMALLLTSAGE        15807408       PAS+PAS+SHELIX+PAS+SHELIX+HISKIN                                                                          744    Deinococcus radiodurans R1                                           sensory box sensor histidine kinase [Deinococcus radiodurans R1]                 
17228724 5.3e-11: _1_[412..451]    EAQDALEQANQELERR---VAKRTL---ALQKANRQLLAEISDRQI        17228724       PAS+PAS+SHELIX+PAS+PAS+GAF+PAS+PAS+PAS+PAS+PAS+HISKIN                                                        1749   Nostoc sp. PCC 7120                                                  two-component sensor histidine kinase [Nostoc sp. PCC 7120]                      
46578838 5.1e-07: _1_[357..396]    NTEAGLRQRLTNIEAM---VATRTE---ELRVANEALHRSSTRFRA        46578838       PAS+PAS+SHELIX+PAS+PAS+GGDEF+EAL                                                                             1072   Desulfovibrio vulgaris subsp. vulgaris str. Hildenborough            sensory box/GGDEF domain/EAL domain protein [Desulfovibrio vulgaris subsp. vulgaris str. Hildenborough]
58039114  0.0002: _1_[688..727]    RTQQQIRDLNVMLEER---ILQRTR---ERDRLWNIARDLFIIIDR        58039114       PAS+PAS+PAS+GAF+SHELIX+PAS+HISKIN+REC                                                                        1210   Gluconobacter oxydans 621H                                           Histidine kinase-response regulator hybrid protein [Gluconobacter oxydans 621H]  
20090485 5.8e-12: _1_[816..855]    KVEEALKKAHETLEEK---VKERTA---ELEEAYNSLKESERGLAE        20090485       PAS+PAS+PAS+GAF+PAS+PAS+SHELIX+PAS+HISKIN                                                                    1196   Methanosarcina acetivorans C2A                                       sensory transduction histidine kinase [Methanosarcina acetivorans C2A]           
21227195 5.9e-10: _1_[450..489]    EAEVKLKQTLNNLEKL---VQERTI---ELEKAYGSLKESERGLAE        21227195       PAS+PAS+PAS+SHELIX+PAS+HISKIN                                                                                831    Methanosarcina mazei Go1                                             hypothetical sensory transduction histidine kinase [Methanosarcina mazei Go1]    
34498703   1e-08: _1_[729..768]    RLGLELDNYRHRLEEL---VEARTA---EVREAHARLQITQFAMDS        34498703       PAS+PAS+PAS+PAS+PAS+PAS+SHELIX+PAS+HISKIN+HAMP+REC+HPT                                                       1480   Chromobacterium violaceum ATCC 12472                                 hypothetical protein CV3248 [Chromobacterium violaceum ATCC 12472]               
17228319 3.2e-10: _1_[380..419]    LAEMALRQLNHQLEAR---VAERTA---ALQNTLAEAQGLNAILDN        17228319       PAS+PAS+PAS+REC+SHELIX+PAS+HISKIN+REC+REC                                                                    1002   Nostoc sp. PCC 7120                                                  two-component hybrid sensor and regulator [Nostoc sp. PCC 7120]                  
42524991    0.27: _1_[269..308]    VNTLSIAMSRNALHKE---LEERIQ---QLHTAHRKKNDFLATLSH        42524991       REC+GAF+SHELIX+HISKIN+REC                                                                                    688    Bdellovibrio bacteriovorus HD100                                     two-component hybrid sensor and regulator [Bdellovibrio bacteriovorus HD100]     
17229731   1e-10: _1_[699..738]    RAEQALQKLNQELEAR---VAERTA---ALRESEERWHLALRGSND        17229731       REC+GAF+PAS+PAS+PAS+PAS+REC+SHELIX+PAS+PAS+PAS+PAS+PAS+PAS+HISKIN                                            1707   Nostoc sp. PCC 7120                                                  two-component hybrid sensor and regulator [Nostoc sp. PCC 7120]                  
17229920 2.2e-11: _1_[949..988]    QIEATLQKINNELELR---VAERTA---ELVNVNRQLQSELDERQR        17229920       REC+HPT+REC+PAS+GAF+PAS+SHELIX+PAS+HISKIN+REC+REC                                                            1627   Nostoc sp. PCC 7120                                                  two-component hybrid sensor and regulator [Nostoc sp. PCC 7120]                  
17232743    0.38: _1_[130..169]    GELKAVVQRAAETYDL---LKQRTE---ELRRANAQMSLLTVLVQV        17232743       REC+SHELIX+GAF                                                                                               315    Nostoc sp. PCC 7120                                                  two-component response regulator [Nostoc sp. PCC 7120]                           
51246965   0.029: _1_[126..165]    RLSRENKKYQEALITQ---VAEKEE---ELSQLDRRFRNMVSSGQK        51246965       REC+SHELIX+GAF+HISKIN+REC                                                                                    711    Desulfotalea psychrophila LSv54                                      similar to two-component system sensory/regulatory protein (Ntr family) [Desulfotalea psychrophila LSv54]
46578687 2.1e-06: _1_[123..161]    RLRMALRAHTEHLEAL---VEARTR---ELVEA-ERLAGMGETAAM        46578687       REC+SHELIX+HISKIN                                                                                            496    Desulfovibrio vulgaris subsp. vulgaris str. Hildenborough            response regulator [Desulfovibrio vulgaris subsp. vulgaris str. Hildenborough]   
22299208    0.47: _1_[121..160]    LARVKNQLTIALLQRK---LRQKNA---LLQKQNQQLQEIAEERRE        22299208       REC+SHELIX+PAS                                                                                               288    Thermosynechococcus elongatus BP-1                                   two-component response regulator [Thermosynechococcus elongatus BP-1]            
54296323 5.6e-06: _1_[160..199]    VLARETQKHTKFLNQL---VEERTE---SLQQSFSLLRATIESSSD        54296323       REC+SHELIX+PAS+GGDEF                                                                                         507    Legionella pneumophila str. Paris                                    hypothetical protein lpp0352 [Legionella pneumophila str. Paris]                 
46579301 5.3e-08: _1_[191..230]    RLLRDEGRLREHLEEQ---VLQRSR---ELEEANAQLRREIAERRD        46579301       REC+SHELIX+PAS+HISKIN                                                                                        570    Desulfovibrio vulgaris subsp. vulgaris str. Hildenborough            response regulator [Desulfovibrio vulgaris subsp. vulgaris str. Hildenborough]   
42522211   5e-05: _1_[110..149]    HQNKQLEALTHSLEGM---VEERTQ---YIEMSHHEESEKLTRERQ        42522211       REC+SHELIX+PAS+HISKIN                                                                                        640    Bdellovibrio bacteriovorus HD100                                     putative two-component sensor histidine kinase [Bdellovibrio bacteriovorus HD100]
46578674 1.7e-07: _1_[122..161]    TMRRRLREYTENLEDM---VAKQSA---RLVAAERQLAALQVMDGI        46578674       REC+SHELIX+PAS+PAS+HISKIN                                                                                    657    Desulfovibrio vulgaris subsp. vulgaris str. Hildenborough            sensory box histidine kinase/response regulator [Desulfovibrio vulgaris subsp. vulgaris str. Hildenborough]
53804207   0.011: _1_[111..150]    RARIRAGERILRLEQQ---LDQRGR---LLEDMNRELKQAYQTIRN        53804207       REC+SHELIX+pp2cSIG                                                                                           391    Methylococcus capsulatus str. Bath                                   response regulator [Methylococcus capsulatus str. Bath]                          
55379044   0.058: _1_[398..437]    AANMEAALAAVERDKQ---LRDREQ---ELSTQNDRLEQFATVVSH        55379044       REC+PAS+GAF+SHELIX+HISKIN                                                                                    644    Haloarcula marismortui ATCC 43049                                    HTR-like protein [Haloarcula marismortui ATCC 43049]                             
39995121   0.091: _1_[408..447]    ANQMAVLIENHTLYDR---MKEKNE---ELVRSRKALKENLEEVKR        39995121       REC+PAS+GAF+SHELIX+HISKIN                                                                                    727    Geobacter sulfurreducens PCA                                         sensory box histidine kinase/response regulator [Geobacter sulfurreducens PCA]   
11499078 3.1e-09: _1_[524..563]    KMRQELEKYTQELEKL---VEERTK---QLAESEKRYRLLVESPIV        11499078       REC+PAS+GAF+PAS+SHELIX+PAS+SHELIX+HISKIN                                                                  908    Archaeoglobus fulgidus DSM 4304                                      signal-transducing histidine kinase [Archaeoglobus fulgidus DSM 4304]            
16331424 2.2e-10: _1_[502..541]    LAEIQLKTINENLEVL---IEERTS---ELESSNSQLLQEIIEKEQ        16331424       REC+PAS+PAS+PAS+SHELIX+PAS+PAS+PAS+HISKIN                                                                    1178   Synechocystis sp. PCC 6803                                           hybrid sensory kinase [Synechocystis sp. PCC 6803]                               
58038745    0.66: _1_[295..334]    ALTLEAARAKAEMAEA---LAQANM---ELARTNERLLQAQSKLVQ        58038745       REC+REC+SHELIX+HISKIN                                                                                        592    Gluconobacter oxydans 621H                                           Sensory Transduction Protein Kinase [Gluconobacter oxydans 621H]                 
56478892 6.1e-07: _1_[552..591]    YIALSNAGHTAAIQRE---VAEQTA---ELTRALDARLAAEDERDR        56478892       T(7TM)M+TM+TM+TM+TM+TM+CHASE+SHELIX+PAS+SHELIX+PAS+HISKIN+REC+REC+HPT                                          1519   Azoarcus sp. EbN1                                                    putative sensory transduction histidine kinase of highly complex domain structure [Azoarcus sp. EbN1]
22298910 1.6e-11: _1_[381..420]    ESFAELHHFNQRLEEK---VKQRTA---ALEAANQQLSEKERVLHD        22298910       TM+CACHE+TM+HAMP+SHELIX+GAF+HISKIN+REC                                                                       1060   Thermosynechococcus elongatus BP-1                                   two-component hybrid sensor and regulator [Thermosynechococcus elongatus BP-1]   
27375470 2.2e-14: _2_[561..599]    VQTEQLKDWNKSLEER---VEKQLG---EIERI-RKLERFLAPQVA        27375470       TM+CACHE+TM+HAMP+SHELIX+GAF+SHELIX+acyc                                                                   815    Bradyrhizobium japonicum USDA 110                                    hypothetical protein blr0359 [Bradyrhizobium japonicum USDA 110]                 
27377973 1.7e-16: _1_[376..415]    EMGARLQESYADLENK---VEQRTA---ELSESLQQQTATADVLKV        27377973       TM+CACHE+TM+HAMP+SHELIX+GAF+SHELIX+HISKIN                                                                 860    Bradyrhizobium japonicum USDA 110                                    two-component hybrid sensor and regulator [Bradyrhizobium japonicum USDA 110]    
51246144 6.8e-14: _1_[332..371]    LQFRNNALRTSEIEWQ---VAEKTA---QLRKSELRAEAIIDKAVI        51246144       TM+CHASE+SHELIX+PAS+SHELIX+HISKIN+REC+HPT                                                                 1102   Desulfotalea psychrophila LSv54                                      similar to two-component system sensory/regulatory protein (hybrid family) [Desulfotalea psychrophila LSv54]
15806193 9.7e-14: _1_[877..916]    DAQHELRQLNQSLEER---VQRRTL---ELEEANRELEAFSYSVSH        15806193       TM+CHASE+PAS+PAS+PAS+GAF+SHELIX+HISKIN                                                                       1131   Deinococcus radiodurans R1                                           sensory box sensor histidine kinase [Deinococcus radiodurans R1]                 
39996138   9e-19: _1_[283..322]    ALFFSTQRQPALLRRL---VAERTK---ELERTNRELTSKVTELEQ        39996138       TM+CHASE+TM+SHELIX+SHELIX+HISKIN                                                                          579    Geobacter sulfurreducens PCA                                         sensor histidine kinase [Geobacter sulfurreducens PCA]                           
28869512 0.00096: _1_[364..402]    LLYSLISQRQRALQ-R---VAQRTR---ELRQREQQLRAAHGQLRN        28869512       TM+CHASE+TM+SHELIX+PAS                                                                                       507    Pseudomonas syringae pv. tomato str. DC3000                          CHASE domain/PAS domain protein [Pseudomonas syringae pv. tomato str. DC3000]    
26988822  0.0077: _1_[334..372]    LLYSLFSQRQRALA-L---VAQRTA---ELQVSEQSLRGTHNQLRS        26988822       TM+CHASE+TM+SHELIX+PAS+PAS+GGDEF                                                                             800    Pseudomonas putida KT2440                                            sensory box protein [Pseudomonas putida KT2440]                                  
32475880    0.13: _1_[469..508]    AIIVRDVTEQRRVDAL---IRQQIA---ELSRSNRDLDAFAYVASH        32475880       TM+CHASE+TM+PAS+SHELIX+HISKIN                                                                                734    Rhodopirellula baltica SH 1                                          signal-transducing histidine kinase [Rhodopirellula baltica SH 1]                
34497076   5e-11: _1_[468..507]    RIKAELQVLSENLEQR---VADRTA---ELDQAWRTLQTVLDAVPF        34497076       TM+CHASE+TM+PAS+SHELIX+PAS+PAS+PAS+PAS+HISKIN+REC+REC+HPT                                                    1748   Chromobacterium violaceum ATCC 12472                                 probable sensor/response regulatory hybrid protein [Chromobacterium violaceum ATCC 12472]
32472027   0.096: _1_[376..415]    QETTQLLIRSETLSEE---LAKKAD---ALVESNKELDNFAYIASH        32472027       TM+CHASE3+HAMP+SHELIX+HISKIN                                                                                 643    Rhodopirellula baltica SH 1                                          TM+HAMP+SHELIX+HISKIN+cyanobacterial phytochrome B [Rhodopirellula baltica SH 1]
21242098   0.041: _2_[203..242]    ALLMRELRHRAQAEQL---AAQANR---ELGQSIDALQRSTADLNL        21242098       TM+CHASE3+SHELIX+TM+SHELIX+GAF+GGDEF                                                                      569    Xanthomonas axonopodis pv. citri str. 306                            sensor histidine kinase [Xanthomonas axonopodis pv. citri str. 306]              
15807871   0.012: _1_[255..294]    TELDRLSAQFHQMAEA---VQQREA---ALAESARSLERTNASLQR        15807871       TM+CHASE3+TM+HAMP+SHELIX+HISKIN                                                                              532    Deinococcus radiodurans R1                                           sensor histidine kinase [Deinococcus radiodurans R1]                             
17545008 0.00067: _1_[210..249]    RERDKLEEESQKLDRM---VRERTR---QLSDLAAHLQRVTEDEKT        17545008       TM+CHASE3+TM+SHELIX+HISKIN                                                                                   474    Ralstonia solanacearum GMI1000                                       TRANSMEMBRANE SENSOR KINASE VSRA TRANSCRIPTION REGULATOR PROTEIN [Ralstonia solanacearum GMI1000]
39935034 5.4e-08: _1_[370..409]    GMAGQVAAREEYLDSM---VKQRTE---ELETRNRSLEGLSAALSK        39935034       TM+HAMP+SHELIX+acyc                                                                                          662    Rhodopseudomonas palustris CGA009                                    putative Adenylate/Guanylate cyclase [Rhodopseudomonas palustris CGA009]         
51891788 2.3e-08: _1_[93..132]     DLAEQLEAVTRNLEEQ---VAVRTA---ALARKADQLRAVGQVGQQ        51891788       TM+HAMP+SHELIX+GAF+HISKIN                                                                                    500    Symbiobacterium thermophilum IAM 14863                               two-component system sensor kinase [Symbiobacterium thermophilum IAM 14863]      
39995706 4.1e-09: _1_[244..283]    KVHGELEDWGKNLEVK---VEERTQ---ELKNIQAQLVHSEKLASL        39995706       TM+HAMP+SHELIX+HISKIN                                                                                        516    Geobacter sulfurreducens PCA                                         sensor histidine kinase [Geobacter sulfurreducens PCA]                           
29829504    0.12: _1_[442..481]    GQRQTMADDREQLRAE---IATATA---HLEQVRQSIHGTFVNLAL        29829504       TM+HAMP+SHELIX+HISKIN                                                                                        974    Streptomyces avermitilis MA-4680                                     putative sensor-like histidine kinase [Streptomyces avermitilis MA-4680]         
39998082 2.8e-10: _1_[414..453]    KSRRDLDSFARLLEQR---VYDRTV---QLEEANEDLRQFSSSLSH        39998082       TM+HAMP+SHELIX+HISKIN                                                                                        677    Geobacter sulfurreducens PCA                                         sensor histidine kinase [Geobacter sulfurreducens PCA]                           
53803111 3.5e-09: _1_[255..294]    RSHEQLEEYNRTLEDK---VRVRTE---ELVEAYRKQKELENEILQ        53803111       TM+HAMP+SHELIX+pp2cSIG                                                                                       543    Methylococcus capsulatus str. Bath                                   HAMP domain protein [Methylococcus capsulatus str. Bath]                         
42525675 2.7e-05: _1_[783..822]    LRFRALQHRAQELDKK---VKEKTK---ELADEKEKSDKLLKNTLP        42525675       TM+SHELIX+acyc                                                                                               1075   Treponema denticola ATCC 35405                                       adenylate/guanylate cyclase catalytic domain protein [Treponema denticola ATCC 35405]
51244166 6.8e-11: _1_[243..282]    QANKELLRHKQHLEIK---IRQRTA---ELTEANQKLEHISLTDGL        51244166       TM+SHELIX+GGDEF                                                                                              449    Desulfotalea psychrophila LSv54                                      sensory transduction system regulatory protein [Desulfotalea psychrophila LSv54] 
53714254 2.8e-05: _1_[632..671]    WRMRSLLKEKERLEAL---VGQRTK---QLVQQKNEIEEKSLKLEK        53714254       TM+SHELIX+HISKIN                                                                                             945    Bacteroides fragilis YCH46                                           hypothetical protein BF2964 [Bacteroides fragilis YCH46]                         
11499355  0.0024: _1_[258..297]    AVLLAAIALNAYLSRV---VAKRTE---EVRKNEAFLRAIFNTIQD        11499355       TM+SHELIX+PAS+PAS+HISKIN                                                                                     595    Archaeoglobus fulgidus DSM 4304                                      TM+SHELIX+PAS+PAS+HISKIN+ amino-acid ABC transporter, periplasmic binding protein/protein kinase [Archaeoglobus fulgidus DSM 4304]
15964171   0.046: _1_[54..93]      EQMSALIAAQTEMQGR---IAAMTE---VLGARQAELNQSLSQRID        15964171       TM+SHELIX+TM                                                                                                 401    Sinorhizobium meliloti 1021                                          hypothetical protein SMc01102 [Sinorhizobium meliloti 1021]                      
56962163     0.8: _1_[45..86]      HQKELLHLYRNELDNQMK-VLEETA---VVISRNADLQAFINNRHE        56962163       TM+SHELIX+TM+HAMP+HISKIN                                                                                     581    Bacillus clausii KSM-K16                                             two-component sensor histidine kinase [Bacillus clausii KSM-K16]                 
46580686 7.9e-08: _1_[309..348]    AARNAVERARNELETR---VAERTA---LLEDSNLHLREAKEKADN        46580686       TM+MCP-N+CACHE+REC+SHELIX+HISKIN+REC                                                                         733    Desulfovibrio vulgaris subsp. vulgaris str. Hildenborough            sensor histidine kinase/response regulator [Desulfovibrio vulgaris subsp. vulgaris str. Hildenborough]
54309156 5.4e-10: _1_[418..457]    RLNALLEKQNQKLEER---VQARTR---SLTEANQKLQQLAYYDPL        54309156       TM+MCP-N+CACHE+TM+HAMP+SHELIX+GGDEF                                                                          620    Photobacterium profundum SS9                                         hypothetical protein PBPRA1970 [Photobacterium profundum SS9]                    
17228138   9e-10: _1_[419..458]    EFFTALETSKAELEDR---VEQRTA---DLKTALTELQRTQAQVIQ        17228138       TM+MCP-N+CACHE+TM+HAMP+SHELIX+HISKIN                                                                         735    Nostoc sp. PCC 7120                                                  two-component sensor histidine kinase [Nostoc sp. PCC 7120]                      
34499590 1.3e-10: _1_[382..421]    ARERQLADVNANLEQM---VADRTE---QLRESNRLLVQKAAQLAQ        34499590       TM+MCP-N+TM+HAMP+SHELIX+GGDEF                                                                                585    Chromobacterium violaceum ATCC 12472                                 hypothetical protein CV4135 [Chromobacterium violaceum ATCC 12472]               
16330590 1.4e-08: _1_[414..453]    EAQISLEHLNEKLEQR---IQERTQ---ELENSQESLLEAKLVAEG        16330590       TM+PAS+GAF+PAS+SHELIX+HISKIN+REC+HPT                                                                         950    Synechocystis sp. PCC 6803                                           hybrid sensory kinase [Synechocystis sp. PCC 6803]                               
54308632 5.5e-07: _1_[373..412]    KYNRQLINFNDKLEKK---VEEKTE---ELTLSLEREERRRDILKS        54308632       TM+TM+HAMP+SHELIX+GGDEF                                                                                      700    Photobacterium profundum SS9                                         hypothetical protein PBPRA1439 [Photobacterium profundum SS9]                    
39934371 0.00047: _1_[383..423]    GMAHAVRDHTRILEDL---VDERTE---KLRRLANIDLLTEIPNRR        39934371       TM+TM+HAMP+SHELIX+GGDEF                                                                                      587    Rhodopseudomonas palustris CGA009                                    putative diguanylate cyclase (GGDEF) with HAMP domain [Rhodopseudomonas palustris CGA009]
17232078 3.9e-11: _1_[351..390]    EGQKKLEAYNQTLEQR---VEERTQ---ALQQKNISLRNTLRELKL        17232078       TM+TM+HAMP+SHELIX+HISKIN                                                                                     688    Nostoc sp. PCC 7120                                                  two-component sensor histidine kinase [Nostoc sp. PCC 7120]                      
16330815   9e-09: _1_[394..433]    LAQKQLAVANSCLEEK---VQQRTE---ELENTVKALELASSEAEA        16330815       TM+TM+HAMP+SHELIX+HISKIN                                                                                     674    Synechocystis sp. PCC 6803                                           sensory transduction histidine kinase [Synechocystis sp. PCC 6803]               
17229043 0.00047: _1_[290..332]    QANEQLTNYNETLENRNQEISEKNN---QLQQVLEELQRSQLKMVQ        17229043       TM+TM+HAMP+SHELIX+HISKIN                                                                                     604    Nostoc sp. PCC 7120                                                  two-component sensor histidine kinase [Nostoc sp. PCC 7120]                      
17231988 7.7e-07: _1_[302..341]    DEIGLLAKSLNRLIER---VSERTK---ELEQA-KELAEAASKAKS        17231988       TM+TM+HAMP+SHELIX+HISKIN                                                                                     575    Nostoc sp. PCC 7120                                                  two-component system sensory histidine kinase [Nostoc sp. PCC 7120]              
56459603 1.2e-08: _1_[243..282]    RAEEEASQLNTELERQ---VTQRTQ---ALKESNSELLKTLEQLHQ        56459603       TM+TM+HAMP+SHELIX+HISKIN                                                                                     539    Idiomarina loihiensis L2TR                                           Signal transduction histidine kinase (contains HAMP domain) [Idiomarina loihiensis L2TR]
34558245 8.1e-07: _1_[435..474]    SMIETLEQDIQTLDSK---VLERTQ---ALESLLEEKEALLKEVHH        34558245       TM+TM+HAMP+SHELIX+HISKIN                                                                                     664    Wolinella succinogenes DSM 1740                                      SIGNAL TRANSDUCTION HISTIDINE KINASE [Wolinella succinogenes DSM 1740]           
16126760 3.9e-09: _1_[446..485]    RQRERNEELLRSLEQR---VAVRTA---ELERANQAKSVFLANMSH        16126760       TM+TM+HAMP+SHELIX+HISKIN+REC                                                                                 839    Caulobacter crescentus CB15                                          sensor histidine kinase/response regulator [Caulobacter crescentus CB15]         
20806576   0.088: _1_[119..161]    RQNEELQEFNAELEASYEQLEALTR---ELELSERKYRLLVENIRD        20806576       TM+TM+HAMP+SHELIX+PAS+GAF+HDGYP                                                                              632    Thermoanaerobacter tengcongensis MB4                                 Response regulators consisting of a CheY-like receiver domain and an HD-GYP domain [Thermoanaerobacter tengcongensis MB4]
30248080 9.7e-10: _2_[539..574]    RDMREHVKIRQDLEQA------RR----DAELANQAKSAFLAAMSH        30248080       TM+TM+HAMP+SHELIX+PAS+SHELIX+HISKIN+REC+HPT                                                               1229   Nitrosomonas europaea ATCC 19718                                     Sensory transduction histidine kinase [Nitrosomonas europaea ATCC 19718]         
46579825 3.1e-12: _2_[381..420]    EAQQALRDAYDKLEAE---VASRTR---DLRRANAQLLLENAERRS        46579825       TM+TM+HAMP+SHELIX+PAS+SHELIX+PAS+GGDEF+EAL                                                                997    Desulfovibrio vulgaris subsp. vulgaris str. Hildenborough            sensory box/GGDEF domain/EAL domain protein [Desulfovibrio vulgaris subsp. vulgaris str. Hildenborough]
46581464   0.012: _1_[199..238]    VELSHIAENFARMAHE---VELRER---ELHAINDRLQQEIAVRRS        46581464       TM+TM+HAMP+SHELIX+PAS+PAS+HISKIN                                                                             764    Desulfovibrio vulgaris subsp. vulgaris str. Hildenborough            sensory box histidine kinase [Desulfovibrio vulgaris subsp. vulgaris str. Hildenborough]
51246243 0.00041: _1_[301..340]    GMSQKFAHSQDSLEAL---LKERNE---QLLELREQTQAYLDIAAV        51246243       TM+TM+HAMP+SHELIX+PAS+PAS+HISKIN+REC                                                                         963    Desulfotalea psychrophila LSv54                                      two-component system sensory/regulatory protein (Ntr family) [Desulfotalea psychrophila LSv54]
56477118   8e-13: _1_[376..415]    KTEAALLRLNATLEAR---VVERTG---QLEAANRELESFSYAISH        56477118       TM+TM+HAMP+PAS+SHELIX+HISKIN                                                                                 625    Azoarcus sp. EbN1                                                    putative two component sensor [Azoarcus sp. EbN1]                                
46581465 3.2e-06: _1_[229..268]    QRNRLLGEQRRRLEME---VSERTA---EFVGARERAERESKAMAD        46581465       TM+TM+HAMP+REC+SHELIX+HISKIN+REC+REC+HPT                                                                     1080   Desulfovibrio vulgaris subsp. vulgaris str. Hildenborough            sensor histidine kinase/response regulator [Desulfovibrio vulgaris subsp. vulgaris str. Hildenborough]
51244217   0.036: _1_[220..259]    MTEKALLRSQHKLENR---VSVQTR---EIEINKKVSMEAMAVLAE        51244217       TM+TM+SHELIX+HDGYP                                                                                           469    Desulfotalea psychrophila LSv54                                      hypothetical protein DP0365 [Desulfotalea psychrophila LSv54]                    
15643926 7.3e-07: _1_[259..298]    ISFGIALTFIYSLRRK---VEERTL---QLKEANEELKAQNEEIED        15643926       TM+TM+SHELIX+HDGYP                                                                                           618    Thermotoga maritima MSB8                                             TM+TM+SHELIX+HDGYP ABC transporter, periplasmic substrate-binding protein/conserved hypothetical protein [Thermotoga maritima MSB8]
16265097    0.15: _1_[241..279]    RGSRTIEKQRHALGQR---VDE-LS---ALLAQNEALRARLRQASQ        16265097       TM+TM+SHELIX+HISKIN                                                                                          490    Sinorhizobium meliloti 1021                                          putative two-component sensor histidine kinase protein [Sinorhizobium meliloti 1021]
29833244     0.5: _1_[61..100]     AEAVRRGRTVRELRAE---VARRTA---DLEERVAAHDAEFVRLGQ        29833244       TM+TM+SHELIX+HISKIN                                                                                          527    Streptomyces avermitilis MA-4680                                     putative sensor-like histidine kinase [Streptomyces avermitilis MA-4680]         
34558099 2.6e-08: _1_[122..161]    TLIATLQELNEELEGR---VQEKTE---SLKKRNQELKEMVLSQDR        34558099       TM+TM+SHELIX+HISKIN                                                                                          263    Wolinella succinogenes DSM 1740                                      HYPOTHETICAL PROTEIN-Signal transduction histidine kinase [Wolinella succinogenes DSM 1740]
34557782    0.65: _1_[291..330]    LLKNVINDMIDNLESK---IKEEIE---KRSEQEKLLIQQSKLASM        34557782       TM+TM+SHELIX+HISKIN                                                                                          548    Wolinella succinogenes DSM 1740                                      PUTATIVE TWO-COMPONENT SENSOR [Wolinella succinogenes DSM 1740]                  
39997849 1.2e-05: _1_[300..339]    VILGGTVLWSHSLRRQ---VAQRTE---SLSRALEEVQLNQQQLLQ        39997849       TM+TM+SHELIX+HISKIN                                                                                          590    Geobacter sulfurreducens PCA                                         periplasmic substrate-binding protein/sensor histidine kinase [Geobacter sulfurreducens PCA]
17232397 1.1e-08: _1_[227..266]    IAQITLRDLNHELEMR---VEKRAR---ELRYKNDQLTQTLQELQQ        17232397       TM+TM+SHELIX+HISKIN                                                                                          551    Nostoc sp. PCC 7120                                                  two-component sensor histidine kinase [Nostoc sp. PCC 7120]                      
34557419 1.1e-08: _1_[244..283]    LILKTIENLHYTLEEK---VAEKTK---ELQELNATLERCIACEVQ        34557419       TM+TM+SHELIX+HISKIN                                                                                          545    Wolinella succinogenes DSM 1740                                      PUTATIVE TWO-COMPONENT SENSOR [Wolinella succinogenes DSM 1740]                  
26989663 4.5e-09: _1_[296..335]    RVNNALQSANDVLEQR---VEERTL---ALRNAQSELFNAARQAGM        26989663       TM+TM+SHELIX+HISKIN                                                                                          604    Pseudomonas putida KT2440                                            sensor histidine kinase [Pseudomonas putida KT2440]                              
24374817 4.4e-07: _1_[794..833]    EKNRHQQDYSRKLEHE---VQIRTN---ELAQKKEEAEEANTAKTR        24374817       TM+TM+SHELIX+HISKIN                                                                                          1171   Shewanella oneidensis MR-1                                           sensor histidine kinase [Shewanella oneidensis MR-1]                             
32265520 0.00054: _1_[193..232]    SIRQSINTNHLLLEQL---VQSKTK---ELQSLNVNLQKSIEYEVE        32265520       TM+TM+SHELIX+HISKIN                                                                                          491    Helicobacter hepaticus ATCC 51449                                    two-component sensor histidine kinase family protein [Helicobacter hepaticus ATCC 51449]
53718372     0.3: _1_[272..313]    KTIKRLQNSAASLEAQ---VKDLTDYI-KLRSTNRSVQSEIVEVAP        53718372       TM+TM+SHELIX+HISKIN+REC                                                                                      602    Burkholderia pseudomallei K96243                                     putative two-component regulator histidine sensor kinase [Burkholderia pseudomallei K96243]
39997539 3.5e-16: _1_[327..358]    RLEQELTRLNEQLEER---VRERTA---EL---ERQV-----ADRR        39997539       TM+TM+SHELIX+SHELIX+HISKIN                                                                                610    Geobacter sulfurreducens PCA                                         sensor histidine kinase [Geobacter sulfurreducens PCA]                           
15598066 5.5e-08: _1_[215..254]    QLAGHLIELNATLEKR---VGERTR---QLSEGKALLHFILEASPS        15598066       TM+TM+SHELIX+PAS+GGDEF                                                                                       525    Pseudomonas aeruginosa PAO1                                          hypothetical protein PA2870 [Pseudomonas aeruginosa PAO1]                        
16331853 5.3e-11: _1_[221..260]    IARQEVLKANSQLEIK---VAERTA---DLAKANEAITGLNAQLKQ        16331853       TM+TM+SHELIX+pp2cSIG                                                                                         510    Synechocystis sp. PCC 6803                                           hypothetical protein slr0114 [Synechocystis sp. PCC 6803]                        
39998446 1.6e-13: _1_[529..568]    EAQEEILRLNVALEER---VRERTA---QLESSNRELESFCYSVSH        39998446       TM+TM+PAS+SHELIX+HISKIN                                                                                      794    Geobacter sulfurreducens PCA                                         sensory box histidine kinase [Geobacter sulfurreducens PCA]                      
39996152  0.0032: _1_[209..244]    RSEEEIKSLNAALE-R------RAR---DLAANNRELEAFSHSLSH        39996152       TM+TM+PAS+SHELIX+HISKIN                                                                                      452    Geobacter sulfurreducens PCA                                         sensory box histidine kinase [Geobacter sulfurreducens PCA]                      
39995120 1.4e-13: _1_[234..273]    KADEEVRRLNAELEER---VEARTQ---ELQRANRELESFSYSVSH        39995120       TM+TM+PAS+SHELIX+HISKIN                                                                                      499    Geobacter sulfurreducens PCA                                         sensory box histidine kinase [Geobacter sulfurreducens PCA]                      
16331091 1.8e-05: _1_[557..596]    IIVAIITNSQANLTHQ---IRQRTK---ELRATLGQLRERVKEMDC        16331091       TM+TM+TM+CHASE+TM+SHELIX+PAS+GGDEF+GAF+PAS+GGDEF+EAL                                                         1578   Synechocystis sp. PCC 6803                                           hypothetical protein sll0267 [Synechocystis sp. PCC 6803]                        
15641101 8.3e-09: _1_[230..269]    DYQHQLEQWNLELEDK---VNQRTE---TLLAKNQQLEEMNTKLTL        15641101       TM+TM+TM+HAMP+SHELIX+HISKIN                                                                                  534    Vibrio cholerae O1 biovar eltor str. N16961                          sensor histidine kinase [Vibrio cholerae O1 biovar eltor str. N16961]            
39995118 1.2e-08: _1_[388..427]    NLNLKLTDMNEFLERR---VEERTA---ALQVEMGRLEAILTSMAE        39995118       TM+TM+TM+HAMP+SHELIX+PAS+HISKIN                                                                              762    Geobacter sulfurreducens PCA                                         sensory box histidine kinase [Geobacter sulfurreducens PCA]                      
21220827     0.8: _1_[182..221]    AVNDERRRIERDLHDG---VQQRLV---ALGMLLGRARRSQDADRR        21220827       TM+TM+TM+SHELIX+HISKIN                                                                                       372    Streptomyces coelicolor A3(2)                                        putative two-component system sensor kinase [Streptomyces coelicolor A3(2)]      
32472613 8.2e-06: _1_[198..237]    VRQTEIEFAKRSVDQQ---VAERTK---ELSEAYDLLAEKTAETEK        32472613       TM+TM+TM+SHELIX+PAS+HISKIN                                                                                   651    Rhodopirellula baltica SH 1                                          probable sensor protein fixL [Rhodopirellula baltica SH 1]                       
16329986 4.3e-12: _1_[147..186]    TAEIALQELNNNLEKR---VEDRTT---QLAKINQQLEQEIEDKTR        16329986       TM+TM+TM+SHELIX+PAS+PAS+GAF+HISKIN                                                                           844    Synechocystis sp. PCC 6803                                           ethylene response sensor protein [Synechocystis sp. PCC 6803]                    
32476784 1.4e-06: _1_[203..242]    KLQSSLTQQRDLLEQR---VRNRTS---EIEQQRRILDTVLQRIPA        32476784       TM+TM+TM+SHELIX+PAS+PAS+HISKIN                                                                               769    Rhodopirellula baltica SH 1                                          probable two-component sensor [Rhodopirellula baltica SH 1]                      
32477267  0.0022: _1_[149..188]    GRLRTRLKHQRTLQHR---VHRRSL---QIRRVNRALRNEVTRRQE        32477267       TM+TM+TM+SHELIX+PAS+PAS+HISKIN+REC+REC+HPT                                                                   1182   Rhodopirellula baltica SH 1                                          histidine protein kinase homolog GacS [Rhodopirellula baltica SH 1]              
39997761 2.6e-13: _1_[222..261]    RVEEEIRRLNTELEQR---VSERTA---ELAASTRELEGFCYSVSH        39997761       TM+TM+TM+PAS+SHELIX+HISKIN                                                                                   498    Geobacter sulfurreducens PCA                                         sensory box histidine kinase [Geobacter sulfurreducens PCA]                      
39934628 1.1e-07: _1_[222..260]    QAEREVHDLNAALERQ---VAERTC---QLEKT-LTLQTAILERAA        39934628       TM+TM+TM+PAS+SHELIX+PAS+HISKIN+REC+HPT                                                                       903    Rhodopseudomonas palustris CGA009                                    sensor histidine kinase with multiple PAS/PAC and a response regulator receiver domain [Rhodopseudomonas palustris CGA009]
46579134 1.7e-12: _1_[637..676]    EAQEQLASFNRELEER---VKQRTR---ELREANQALEFSLDAVRR        46579134       TM+TM+TM+PAS+PAS+SHELIX+HISKIN                                                                               936    Desulfovibrio vulgaris subsp. vulgaris str. Hildenborough            sensory box histidine kinase [Desulfovibrio vulgaris subsp. vulgaris str. Hildenborough]
19553214    0.96: _1_[143..182]    VMGTCFQLLAQALKEL---VDARAS---AIRASKSAGEQAERARIA        19553214       TM+TM+TM+TM+TM+SHELIX+HISKIN                                                                                 377    Corynebacterium glutamicum ATCC 13032                                two-component system, sensory transduction histidine kinase [Corynebacterium glutamicum ATCC 13032]
17547455    0.48: _1_[175..214]    NELGRFQLFQLK-EE----LAEKNE---QLEAINHSLTEIKGQLLQ        17547455       TM+TM+TM+TM+TM+SHELIX+HISKIN                                                                                 442    Ralstonia solanacearum GMI1000                                       TRANSMEMBRANE SENSOR HISTIDINE KINASE TRANSCRIPTION REGULATOR PROTEIN [Ralstonia solanacearum GMI1000]
16330151 3.4e-09: _1_[542..581]    NQFKIIKQAKDNLEMR---VAERTH---TLIVINKQLAGEIEERQR        16330151       TM+TM+TM+TM+TM+MCP-N+TM+SHELIX+HISKIN                                                                        998    Synechocystis sp. PCC 6803                                           sensory transduction histidine kinase [Synechocystis sp. PCC 6803]               
51891631   2e-06: _1_[225..264]    QEFHRMNRLLDTLEDK---VADRTD---QLAQTVRALERRLAESEA        51891631       TM+TM+TM+TM+TM+TM+SHELIX+GAF+HISKIN                                                                          633    Symbiobacterium thermophilum IAM 14863                               two-component sensor histidine kinase [Symbiobacterium thermophilum IAM 14863]   
24374087 2.7e-07: _1_[522..561]    STMNEVKLLNEALEER---VEKRTK---ELEQERDKANQLAAVKAR        24374087       TM+TM+TM+TM+TM+TM+MCP-N+TM+HAMP+SHELIX+HISKIN+REC+HPT                                                        1019   Shewanella oneidensis MR-1                                           sensor histidine kinase/response regulator [Shewanella oneidensis MR-1]          
27378095 3.4e-11: _1_[258..297]    RAEAQLARLNEELEVR---VEERTA---QLAASNLALESEILVRRH        27378095       TM+TM+TM+TM+TM+TM+TM+SHELIX                                                                                  456    Bradyrhizobium japonicum USDA 110                                    Real hypothetical protein blr2984 [Bradyrhizobium japonicum USDA 110]                 
56479368   8e-06: _1_[454..493]    AREHADRQAYAALDRR---VGDRTR---ALERTMSVLRESTTLLRT        56479368       TM+TM+TM+TM+TM+TM+TM+PAS+SHELIX+GAF+PAS+HISKIN+REC                                                           1015   Azoarcus sp. EbN1                                                    two-component regulatory system protein containing hybrid kinase and response regulator domains [Azoarcus sp. EbN1]
15678384  0.0087: _1_[354..393]    TDITDLIGIQRSLEST---VSERDA---LLAEVHHRVKNNLQIIMS        15678384       TM+TM+TM+TM+TM+TM+TM+PAS+SHELIX+HISKIN                                                                       567    Methanothermobacter thermautotrophicus str. Delta H                  sensory transduction histidine kinase [Methanothermobacter thermautotrophicus str. Delta H]
39995583   0.007: _1_[380..415]    RAARQIERLNESLAS-------RAM---DLEVANGDLEAFNYSVSH        39995583       TM+TM+TM+TM+TM+TM+TM+PAS+SHELIX+HISKIN                                                                       622    Geobacter sulfurreducens PCA                                         sensory box histidine kinase [Geobacter sulfurreducens PCA]                      
56751584 1.9e-08: _1_[418..457]    HTDRQLRDLSENLEHL---VQIRTT---ELESIRDQLETAQRVAKV        56751584       TM+TM+TM+TM+TM+TM+TM+PAS+SHELIX+PAS+PAS+GAF+GGDEF                                                            1068   Synechococcus elongatus PCC 6301                                     hypothetical protein syc1575_c [Synechococcus elongatus PCC 6301]                
39996145   0.021: _1_[510..559]    ELEDRAEVLEETNCEL------ETSVE-QLEAVNRELEDANEELEA        39996145       TM+TM+TM+TM+TM+TM+TM+PAS+PAS+SHELIX+HISKIN                                                                   767    Geobacter sulfurreducens PCA                                         sensory box histidine kinase [Geobacter sulfurreducens PCA]                      
53718888 1.7e-10: _1_[301..340]    EKTVQLNEANELLEQR---VAERTR---ELQAANDELRREIVERER        53718888       TM+TM+TM+TM+TM+TM+TM+TM+SHELIX+HISKIN                                                                        575    Burkholderia pseudomallei K96243                                     putative integral membrane protein/sensor kinase [Burkholderia pseudomallei K96243]
15893919 3.9e-06: _1_[313..352]    TLLTYEKDINLILEEK---IKERTK---ELLLKNQELEYISNHDPI        15893919       TM+TM+TM+TM+TM+TM+TM+TM+TM+SHELIX+GGDEF+EAL                                                                  772    Clostridium acetobutylicum ATCC 824                                  Signal transduction protein containing diguanylate cyclase/phosphodiesterase domain (GGDEF) and domain (EAL) [Clostridium acetobutylicum ATCC 824]
56475528   0.024: _1_[644..686]    DEASQVRAYSHELEEKSRALESATA---ELRAANERLKELDRLKDD        56475528       TM+TM+TM+TM+TM+TM+TM+TM+TM+TM+TM+TM+SHELIX+HISKIN                                                            918    Azoarcus sp. EbN1                                                    fusion protein of probable Na-dependent permease domain and histidine kinase domain of putative two-component sensor [Azoarcus sp. EbN1]
46579568   5e-09: _1_[794..833]    HSEERLKELNESLESQ---VRLRTE---ELERSFESVRQADKMASL        46579568       TM+TM+TM+TM+TM+TM+TM+TM+TM+TM+TM+TM+TM+PAS+SHELIX+HISKIN                                                     1085   Desulfovibrio vulgaris subsp. vulgaris str. Hildenborough            sensory box histidine kinase [Desulfovibrio vulgaris subsp. vulgaris str. Hildenborough]
51473653    0.33: _1_[665..704]    DQEMLINLKNTHLGNR---VNYREQ---ELEKLLDLKHEFLRNINH        51473653       TM+TM+TM+TM+TM+TM+TM+TM+TM+TM+TM+TM+TM+TM+TM+TM+TM+TM+SHELIX+HISKIN                                          916    Rickettsia typhi str. Wilmington                                     two-component sensor histidine kinase [Rickettsia typhi str. Wilmington]         


EUKARYOTES
# 76
30178957       STYkin+SHELIX+acyc                                           875    Anopheles gambiae str. PEST           ENSANGP00000013324 [Anopheles gambiae str. PEST]                                                                                                                                                                                                            
21298041       STYkin+SHELIX+acyc                                           1106   Anopheles gambiae str. PEST           ENSANGP00000014144 [Anopheles gambiae str. PEST]                                                                                                                                                                                                            
4505437        STYkin+SHELIX+acyc                                           995    Homo sapiens                          natriuretic peptide receptor B isoform a precursor [Homo sapiens]                                                                                                                                                                                           
4580422        STYkin+SHELIX+acyc                                           1047   Homo sapiens                          natriuretic peptide receptor B isoform b precursor [Homo sapiens]                                                                                                                                                                                           
72008591       STYkin+SHELIX+acyc+STYkin+SHELIX+acyc                     1042   Strongylocentrotus purpuratus         PREDICTED: similar to Atrial natriuretic peptide receptor A precursor (ANP-A) (ANPRA) (GC-A) (Guanylate cyclase) (NPR-A) (Atrial natriuretic peptide A-type receptor) [Strongylocentrotus purpuratus]                                                       
48098407       STYkin+SHELIX+acyc                                           1292   Apis mellifera                        similar to CG31183-PA [Apis mellifera]                                                                                                                                                                                                                      
6981280        STYkin+SHELIX+acyc                                           1057   Rattus norvegicus                     natriuretic peptide receptor 1 [Rattus norvegicus]                                                                                                                                                                                                          
47207435       STYkin+SHELIX+acyc                                           549    Tetraodon nigroviridis                unnamed protein product [Tetraodon nigroviridis]                                                                                                                                                                                                            
72014291       STYkin+SHELIX+acyc                                           1178   Strongylocentrotus purpuratus         PREDICTED: similar to Atrial natriuretic peptide receptor A precursor (ANP-A) (ANPRA) (GC-A) (Guanylate cyclase) (NPR-A) (Atrial natriuretic peptide A-type receptor) [Strongylocentrotus purpuratus]                                                       
72006640       STYkin+SHELIX+acyc                                           796    Strongylocentrotus purpuratus         PREDICTED: similar to Atrial natriuretic peptide receptor B precursor (ANP-B) (ANPRB) (GC-B) (Guanylate cyclase B) (NPR-B) (Atrial natriuretic peptide B-type receptor) [Strongylocentrotus purpuratus]                                                     
72004807       STYkin+SHELIX+acyc                                           705    Strongylocentrotus purpuratus         PREDICTED: similar to Atrial natriuretic peptide receptor A precursor (ANP-A) (ANPRA) (GC-A) (Guanylate cyclase) (NPR-A) (Atrial natriuretic peptide A-type receptor) [Strongylocentrotus purpuratus]                                                       
51771626       STYkin+SHELIX+acyc                                           1100   Mus musculus                          PREDICTED: guanylate cyclase 2g [Mus musculus]                                                                                                                                                                                                              
48112650       STYkin+SHELIX+acyc                                           1420   Apis mellifera                        similar to ENSANGP00000009147 [Apis mellifera]                                                                                                                                                                                                              
47220620       STYkin+SHELIX+acyc                                           510    Tetraodon nigroviridis                unnamed protein product [Tetraodon nigroviridis]                                                                                                                                                                                                            
47208213       STYkin+SHELIX+acyc                                           993    Tetraodon nigroviridis                unnamed protein product [Tetraodon nigroviridis]                                                                                                                                                                                                            
47205438       STYkin+SHELIX+acyc                                           471    Tetraodon nigroviridis                unnamed protein product [Tetraodon nigroviridis]                                                                                                                                                                                                            
34328514       STYkin+SHELIX+acyc                                           1057   Mus musculus                          natriuretic peptide receptor 1 [Mus musculus]                                                                                                                                                                                                               
30179077       STYkin+SHELIX+acyc                                           1194   Anopheles gambiae str. PEST           ENSANGP00000012934 [Anopheles gambiae str. PEST]                                                                                                                                                                                                            
24656567       STYkin+SHELIX+acyc                                           1076   Drosophila melanogaster               CG3216-PA, isoform A [Drosophila melanogaster]                                                                                                                                                                                                              
28916675       STYkin+SHELIX+acyc                                           1047   Mus musculus                          natriuretic peptide receptor 2 [Mus musculus]                                                                                                                                                                                                               
28574512       STYkin+SHELIX+acyc                                           1163   Drosophila melanogaster               CG33114-PA [Drosophila melanogaster]                                                                                                                                                                                                                        
17540902       STYkin+SHELIX+acyc                                           613    Caenorhabditis elegans                Guanylyl CYclase family member (gcy-27) [Caenorhabditis elegans]                                                                                                                                                                                            
24666940       STYkin+SHELIX+acyc                                           1525   Drosophila melanogaster               CG8742-PA, isoform A [Drosophila melanogaster]                                                                                                                                                                                                              
24663843       STYkin+SHELIX+acyc                                           1172   Drosophila melanogaster               CG10738-PA, isoform A [Drosophila melanogaster]                                                                                                                                                                                                             
24663839       STYkin+SHELIX+acyc                                           1272   Drosophila melanogaster               CG10738-PB, isoform B [Drosophila melanogaster]                                                                                                                                                                                                             
24644018       STYkin+SHELIX+acyc                                           975    Drosophila melanogaster               CG9783-PA [Drosophila melanogaster]                                                                                                                                                                                                                         
21287856       STYkin+SHELIX+acyc                                           1127   Anopheles gambiae str. PEST           ENSANGP00000009149 [Anopheles gambiae str. PEST]                                                                                                                                                                                                            
20514776       STYkin+SHELIX+acyc                                           1100   Rattus norvegicus                     guanylate cyclase 2g [Rattus norvegicus]                                                                                                                                                                                                                    
17568387       STYkin+SHELIX+acyc                                           1057   Caenorhabditis elegans                Guanylyl CYclase family member (gcy-11) [Caenorhabditis elegans]                                                                                                                                                                                            
47213880       STYkin+STYkin+SHELIX+acyc                                    1001   Tetraodon nigroviridis                unnamed protein product [Tetraodon nigroviridis]                                                                                                                                                                                                            
40254426       STYkin+STYkin+SHELIX+acyc                                    1061   Homo sapiens                          natriuretic peptide receptor A/guanylate cyclase A (atrionatriuretic peptide receptor A) [Homo sapiens]                                                                                                                                                     
32562827       TM+STYkin+STYkin+SHELIX+acyc                                 1276   Caenorhabditis elegans                guanylyl cyclase (144.9 kD) (1F75) [Caenorhabditis elegans]                                                                                                                                                                                                 
32563666       TM+STYkin+SHELIX+acyc                                        1146   Caenorhabditis elegans                Guanylyl CYclase family member (gcy-17) [Caenorhabditis elegans]                                                                                                                                                                                            
21287855       TM+STYkin+SHELIX+acyc                                        1154   Anopheles gambiae str. PEST           ENSANGP00000009147 [Anopheles gambiae str. PEST]                                                                                                                                                                                                            
72043227       TM+STYkin+SHELIX+acyc                                        969    Strongylocentrotus purpuratus         PREDICTED: similar to Atrial natriuretic peptide receptor A precursor (ANP-A) (ANPRA) (GC-A) (Guanylate cyclase) (NPR-A) (Atrial natriuretic peptide A-type receptor), partial [Strongylocentrotus purpuratus]                                              
17561798       TM+STYkin+SHELIX+acyc                                        1025   Caenorhabditis elegans                Guanylyl CYclase family member (gcy-13) [Caenorhabditis elegans]                                                                                                                                                                                            
24647268       TM+STYkin+SHELIX+acyc                                        1417   Drosophila melanogaster               CG31183-PA [Drosophila melanogaster]                                                                                                                                                                                                                        
24656571       TM+STYkin+SHELIX+acyc                                        1141   Drosophila melanogaster               CG3216-PB, isoform B [Drosophila melanogaster]                                                                                                                                                                                                              
72011180       TM+STYkin+SHELIX+acyc                                        656    Strongylocentrotus purpuratus         PREDICTED: similar to Atrial natriuretic peptide receptor A precursor (ANP-A) (ANPRA) (GC-A) (Guanylate cyclase) (NPR-A) (Atrial natriuretic peptide A-type receptor) [Strongylocentrotus purpuratus]                                                       
72010207       TM+STYkin+SHELIX+acyc                                        908    Strongylocentrotus purpuratus         PREDICTED: similar to CG10738-PA, isoform A [Strongylocentrotus purpuratus]                                                                                                                                                                                 
17534649       TM+STYkin+SHELIX+acyc                                        1137   Caenorhabditis elegans                Guanylyl CYclase family member (gcy-1) [Caenorhabditis elegans]                                                                                                                                                                                             
47226131       TM+STYkin+SHELIX+acyc+acyc                                   878    Tetraodon nigroviridis                unnamed protein product [Tetraodon nigroviridis]                                                                                                                                                                                                            
17540898       TM+STYkin+SHELIX+acyc+TM                                     1139   Caenorhabditis elegans                guanylyl cyclase (gcy-23) [Caenorhabditis elegans]                                                                                                                                                                                                          
17534651       TM+STYkin+SHELIX+acyc                                        1118   Caenorhabditis elegans                Guanylyl CYclase family member (gcy-2) [Caenorhabditis elegans]                                                                                                                                                                                             
17540896       TM+STYkin+SHELIX+acyc                                        1112   Caenorhabditis elegans                Guanylyl CYclase family member (gcy-18) [Caenorhabditis elegans]                                                                                                                                                                                            
17540894       TM+STYkin+SHELIX+acyc                                        1206   Caenorhabditis elegans                Guanylyl CYclase family member (gcy-8) [Caenorhabditis elegans]                                                                                                                                                                                             
51467872       TM+STYkin+SHELIX+TM+acyc+TM                                  1090   Homo sapiens                          PREDICTED: similar to guanylyl cyclase receptor G [Homo sapiens]                                                                                                                                                                                            
17534653       TM+STYkin+SHELIX+acyc                                        1140   Caenorhabditis elegans                Guanylyl CYclase family member (gcy-3) [Caenorhabditis elegans]                                                                                                                                                                                             
32566183       TM+STYkin+SHELIX+acyc                                        1016   Caenorhabditis elegans                ODoRant response abnormal ODR-1, guanylyl cyclase (115.7 kD) (odr-1) [Caenorhabditis elegans]                                                                                                                                                               
17534665       TM+STYkin+SHELIX+acyc                                        1119   Caenorhabditis elegans                Guanylyl CYclase family member (gcy-21) [Caenorhabditis elegans]                                                                                                                                                                                            
38085380       TM+STYkin+SHELIX+acyc                                        1072   Mus musculus                          PREDICTED: guanylate cyclase 2c [Mus musculus]                                                                                                                                                                                                              
72004443       TM+STYkin+SHELIX                                             806    Strongylocentrotus purpuratus         PREDICTED: similar to Atrial natriuretic peptide receptor A precursor (ANP-A) (ANPRA) (GC-A) (Guanylate cyclase) (NPR-A) (Atrial natriuretic peptide A-type receptor) [Strongylocentrotus purpuratus]                                                       
47204849       TM+STYkin+SHELIX+acyc                                        659    Tetraodon nigroviridis                unnamed protein product [Tetraodon nigroviridis]                                                                                                                                                                                                            
17534661       TM+STYkin+SHELIX+acyc                                        1000   Caenorhabditis elegans                Guanylyl CYclase family member (gcy-15) [Caenorhabditis elegans]                                                                                                                                                                                            
47210702       TM+STYkin+SHELIX+acyc                                        1071   Tetraodon nigroviridis                unnamed protein product [Tetraodon nigroviridis]                                                                                                                                                                                                            
17534659       TM+STYkin+SHELIX+acyc                                        1280   Caenorhabditis elegans                Guanylyl CYclase family member (gcy-12) [Caenorhabditis elegans]                                                                                                                                                                                            
4504217        TM+STYkin+SHELIX+acyc                                        1103   Homo sapiens                          guanylate cyclase 2D, membrane (retina-specific) [Homo sapiens]                                                                                                                                                                                             
47210257       TM+STYkin+SHELIX+acyc                                        1128   Tetraodon nigroviridis                unnamed protein product [Tetraodon nigroviridis]                                                                                                                                                                                                            
47207887       TM+TM+STYkin+SHELIX+acyc                                     1154   Tetraodon nigroviridis                unnamed protein product [Tetraodon nigroviridis]                                                                                                                                                                                                            
17534657       TM+TM+STYkin+SHELIX+acyc                                     1122   Caenorhabditis elegans                Guanylyl CYclase family member (gcy-5) [Caenorhabditis elegans]                                                                                                                                                                                             
47222531       TM+TM+STYkin+SHELIX+acyc                                     1037   Tetraodon nigroviridis                unnamed protein product [Tetraodon nigroviridis]                                                                                                                                                                                                            
47551057       TM+TM+STYkin+SHELIX+acyc                                     1125   Strongylocentrotus purpuratus         guanylate cyclase [Strongylocentrotus purpuratus]                                                                                                                                                                                                           
32563954       TM+TM+STYkin+SHELIX+acyc                                     1034   Caenorhabditis elegans                C04H5.3 [Caenorhabditis elegans]                                                                                                                                                                                                                            
4504219        TM+TM+STYkin+SHELIX+acyc                                     1108   Homo sapiens                          guanylate cyclase 2F [Homo sapiens]                                                                                                                                                                                                                         
17561800       TM+TM+STYkin+SHELIX+acyc                                     1170   Caenorhabditis elegans                Guanylyl CYclase family member (gcy-14) [Caenorhabditis elegans]                                                                                                                                                                                            
51761722       TM+TM+STYkin+SHELIX+acyc                                     1421   Mus musculus                          similar to Olfactory guanylyl cyclase GC-D precursor (Guanylate cyclase, olfactory) [Mus musculus]                                                                                                                                                          
30174226       TM+TM+STYkin+SHELIX+acyc                                     993    Anopheles gambiae str. PEST           ENSANGP00000006391 [Anopheles gambiae str. PEST]                                                                                                                                                                                                            
51772455       TM+TM+STYkin+SHELIX+acyc                                     1152   Mus musculus                          similar to guanylate cyclase 2F, retinal [Mus musculus]                                                                                                                                                                                                     
17540900       TM+TM+STYkin+SHELIX+acyc                                     1091   Caenorhabditis elegans                Guanylyl CYclase family member (gcy-25) [Caenorhabditis elegans]                                                                                                                                                                                            
17559160       TM+TM+STYkin+SHELIX+acyc                                     1093   Caenorhabditis elegans                abnormal DAuer Formation family member (daf-11) [Caenorhabditis elegans]                                                                                                                                                                                    
16758694       TM+TM+STYkin+SHELIX+acyc                                     1047   Rattus norvegicus                     natriuretic peptide receptor 2 [Rattus norvegicus]                                                                                                                                                                                                          
17561794       TM+TM+STYkin+SHELIX+acyc                                     1161   Caenorhabditis elegans                Guanylyl CYclase family member (gcy-6) [Caenorhabditis elegans]                                                                                                                                                                                             
17561796       TM+TM+STYkin+SHELIX+acyc                                     1130   Caenorhabditis elegans                guanylyl cyclase (gcy-7) [Caenorhabditis elegans]                                                                                                                                                                                                           
72011791       TM+TM+STYkin+SHELIX+acyc                                     1040   Strongylocentrotus purpuratus         PREDICTED: similar to CG10738-PA, isoform A [Strongylocentrotus purpuratus]                                                                                                                                                                                 
16758684       TM+TM+STYkin+SHELIX+acyc                                     1108   Rattus norvegicus                     guanylate cyclase 2f [Rattus norvegicus]                                                                                                                                                                                                                    
17561802       TM+TM+STYkin+SHELIX+acyc                                     1108   Caenorhabditis elegans                Guanylyl CYclase family member (gcy-20) [Caenorhabditis elegans]                                                                                                                                                                                            
13242283       TM+TM+STYkin+SHELIX+acyc                                     1108   Rattus norvegicus                     guanylyl cyclase 2e [Rattus norvegicus]                                                                                                                                                                                                                     
6680135        TM+TM+STYkin+SHELIX+acyc                                     1108   Mus musculus                          guanylate cyclase 2e [Mus musculus]                                                                                                                                                                                                                         
4826752        TM+TM+STYkin+SHELIX+acyc                                     1073   Homo sapiens                          guanylate cyclase 2C (heat stable enterotoxin receptor) [Homo sapiens]                                                                                                                                                                                      
47211414       TM+TM+STYkin+SHELIX+acyc                                     2022   Tetraodon nigroviridis                unnamed protein product [Tetraodon nigroviridis]                                                                                                                                                                                                            
18543337       TM+TM+STYkin+SHELIX+acyc                                     1110   Rattus norvegicus                     guanylate cyclase 2d [Rattus norvegicus]                                                                                                                                                                                                                    
47209256       TM+TM+STYkin+SHELIX+acyc                                     1103   Tetraodon nigroviridis                unnamed protein product [Tetraodon nigroviridis]                                                                                                                                                                                                            
17568383       TM+TM+STYkin+SHELIX+acyc                                     1118   Caenorhabditis elegans                Guanylyl CYclase family member (gcy-9) [Caenorhabditis elegans]                                                                                                                                                                                             
48124391       TM+TM+TM+STYkin+SHELIX+acyc                                  969    Apis mellifera                        similar to CG3216-PB [Apis mellifera]                                                                                                                                                                                                                       
17561804       TM+TM+TM+STYkin+SHELIX+acyc                                  1012   Caenorhabditis elegans                Guanylyl CYclase family member (gcy-22) [Caenorhabditis elegans]                                                                                                                                                                                            
32563952       TM+TM+TM+TM+TM+STYkin+SHELIX+acyc                            1246   Caenorhabditis elegans                guanylyl cyclase (gcy-19) [Caenorhabditis elegans]                                                                                                                                                                                                          
# 33
48101339       HNOB+HNOB+HNOBA+SHELIX+acyc                                  633    Apis mellifera                        similar to soluble guanylyl cyclase beta-3 [Apis mellifera]                                                                                                                                                                                                 
17540904       HNOB+HNOB+HNOBA+SHELIX+acyc                                  583    Caenorhabditis elegans                predicted CDS, guanylyl cyclase (gcy-37) [Caenorhabditis elegans]                                                                                                                                                                                           
6980996        HNOB+HNOBA+SHELIX+acyc                                       619    Rattus norvegicus                     guanylate cyclase 1, soluble, beta 3; Guanylate cyclase soluble beta 1 (GTP pyrophosphate - lyase); guanylate cyclase 1 soluble beta 3; Guanylate cyclase soluble beta 1 (GTP pyrophosphate - lyase) see also D2Mgh17; Guanylate cyclase, soluble, beta 1 (GTP pyrophosphate - lyase) see also D2Mgh17; Guanylate cyclase, soluble, beta 1 (GTP pyrophosphate - lyase); Gucy1b3 protein [Rattus norvegicu
4504213        HNOB+HNOBA+SHELIX+acyc+HNOB                                  717    Homo sapiens                          guanylate cyclase 1, soluble, alpha 3 [Homo sapiens]                                                                                                                                                                                                        
8393507        HNOB+HNOBA+SHELIX+acyc                                       617    Homo sapiens                          guanylate cyclase 1, soluble, beta 2 [Homo sapiens]                                                                                                                                                                                                         
8567358        HNOB+HNOBA+SHELIX+acyc                                       620    Mus musculus                          guanylate cyclase 1, soluble, beta 3 [Mus musculus]                                                                                                                                                                                                         
17507861       HNOB+HNOBA+SHELIX+acyc                                       751    Caenorhabditis elegans                guanylyl cyclase (85.3 kD) (gcy-35) [Caenorhabditis elegans]                                                                                                                                                                                                
6980998        HNOB+HNOBA+SHELIX+acyc                                       682    Rattus norvegicus                     guanylate cyclase 1, soluble, beta 2 [Rattus norvegicus]                                                                                                                                                                                                    
4504215        HNOB+HNOBA+SHELIX+acyc                                       619    Homo sapiens                          guanylate cyclase 1, soluble, beta 3 [Homo sapiens]                                                                                                                                                                                                         
17561808       HNOB+HNOBA+SHELIX+acyc                                       947    Caenorhabditis elegans                guanylyl cyclase (gcy-33) [Caenorhabditis elegans]                                                                                                                                                                                                          
17561810       HNOB+HNOBA+SHELIX+acyc                                       686    Caenorhabditis elegans                Guanylyl CYclase family member (gcy-34) [Caenorhabditis elegans]                                                                                                                                                                                            
17568389       HNOB+HNOBA+SHELIX+acyc                                       752    Caenorhabditis elegans                predicted CDS, guanylyl cyclase (gcy-31) [Caenorhabditis elegans]                                                                                                                                                                                           
21288869       HNOB+HNOBA+SHELIX+acyc                                       720    Anopheles gambiae str. PEST           ENSANGP00000018455 [Anopheles gambiae str. PEST]                                                                                                                                                                                                            
21297018       HNOB+HNOBA+SHELIX+acyc                                       670    Anopheles gambiae str. PEST           ENSANGP00000003998 [Anopheles gambiae str. PEST]                                                                                                                                                                                                            
21355729       HNOB+HNOBA+SHELIX+acyc                                       669    Drosophila melanogaster               CG14886-PA [Drosophila melanogaster]                                                                                                                                                                                                                        
24646993       HNOB+HNOBA+SHELIX+acyc                                       947    Drosophila melanogaster               CG4154-PC [Drosophila melanogaster]                                                                                                                                                                                                                         
24646995       HNOB+HNOBA+SHELIX+acyc                                       940    Drosophila melanogaster               CG4154-PA [Drosophila melanogaster]                                                                                                                                                                                                                         
24647455       HNOB+HNOBA+SHELIX+acyc                                       667    Drosophila melanogaster               CG14885-PA [Drosophila melanogaster]                                                                                                                                                                                                                        
24651096       HNOB+HNOBA+SHELIX+acyc                                       676    Drosophila melanogaster               CG1912-PA [Drosophila melanogaster]                                                                                                                                                                                                                         
27370208       HNOB+HNOBA+SHELIX+acyc                                       824    Mus musculus                          guanylate cyclase 1, soluble, beta 2 [Mus musculus]                                                                                                                                                                                                         
30178012       HNOB+HNOBA+SHELIX+acyc                                       587    Anopheles gambiae str. PEST           ENSANGP00000023905 [Anopheles gambiae str. PEST]                                                                                                                                                                                                            
31981219       HNOB+HNOBA+SHELIX+acyc+HNOB                                  691    Mus musculus                          guanylate cyclase 1, soluble, alpha 3 [Mus musculus]                                                                                                                                                                                                        
32566105       HNOB+HNOBA+SHELIX+acyc                                       646    Caenorhabditis elegans                guanylyl cyclase (gcy-32) [Caenorhabditis elegans]                                                                                                                                                                                                          
32566352       HNOB+HNOBA+SHELIX+acyc                                       663    Caenorhabditis elegans                Guanylyl CYclase family member (gcy-36) [Caenorhabditis elegans]                                                                                                                                                                                            
34857867       HNOB+HNOBA+SHELIX+acyc+HNOB                                  690    Rattus norvegicus                     hypothetical protein XP_346628 [Rattus norvegicus]                                                                                                                                                                                                          
38089705       HNOB+HNOBA+SHELIX+acyc                                       567    Mus musculus                          PREDICTED: similar to guanylate cyclase 1, soluble, alpha 2 [Mus musculus]                                                                                                                                                                                  
48096192       HNOB+HNOBA+SHELIX+acyc                                       807    Apis mellifera                        similar to soluble guanylyl cyclase alpha-1 subunit [Apis mellifera]                                                                                                                                                                                        
48096194       HNOB+HNOBA+SHELIX+acyc                                       720    Apis mellifera                        similar to soluble guanylyl cyclase beta-1 subunit [Apis mellifera]                                                                                                                                                                                         
4504211        HNOB+HNOBA+SHELIX+acyc                                       732    Homo sapiens                          guanylate cyclase 1, soluble, alpha 2 [Homo sapiens]                                                                                                                                                                                                        
72007659       HNOB+HNOBA+SHELIX+acyc                                       645    Strongylocentrotus purpuratus         PREDICTED: similar to guanylate cyclase 1, soluble, beta 2 [Strongylocentrotus purpuratus]                                                                                                                                                                  
72011887       HNOB+HNOBA+HNOB+SHELIX+acyc                                  780    Strongylocentrotus purpuratus         PREDICTED: similar to guanylate cyclase 1, soluble, beta 2 [Strongylocentrotus purpuratus]                                                                                                                                                                  
47218455       HNOB+HNOBA+SHELIX+acyc+HNOB+HNOBA+SHELIX+acyc             1287   Tetraodon nigroviridis                unnamed protein product [Tetraodon nigroviridis]                                                                                                                                                                                                            
72053333       HNOB+HNOBA+SHELIX+acyc                                       604    Strongylocentrotus purpuratus         PREDICTED: similar to Guanylate cyclase soluble, beta-1 chain (GCS-beta-1) (Soluble guanylate cyclase small subunit) (GCS-beta-3) [Strongylocentrotus purpuratus]                                                                                           
47213713       HNOB+HNOBA+HNOBA+SHELIX+acyc                                 860    Tetraodon nigroviridis                unnamed protein product [Tetraodon nigroviridis]                                                                                                                                                                                                            
30178013       HNOB+HNOBA+SHELIX+acyc                                       652    Anopheles gambiae str. PEST           ENSANGP00000012727 [Anopheles gambiae str. PEST]                                                                                                                                                                                                            
24651577       HNOB+HNOBA+SHELIX+acyc                                       787    Drosophila melanogaster               CG1470-PA [Drosophila melanogaster]                                                                                                                                                                                                                         
21294357       HNOB+HNOBA+SHELIX+acyc                                       632    Anopheles gambiae str. PEST           ENSANGP00000012438 [Anopheles gambiae str. PEST]                                                                                                                                                                                                            
72025854       HNOBA+SHELIX+acyc                                            552    Strongylocentrotus purpuratus         PREDICTED: similar to Guanylate cyclase soluble, beta-2 chain (GCS-beta-2), partial [Strongylocentrotus purpuratus]                                                                                                                                         
72007704       TM+TM+TM+HNOBA+SHELIX+acyc+TM+HNOB+HNOBA+SHELIX+acyc      1018   Strongylocentrotus purpuratus         PREDICTED: similar to Guanylate cyclase soluble, beta-1 chain (GCS-beta-1) (Soluble guanylate cyclase small subunit) (GCS-beta-3) [Strongylocentrotus purpuratus]                                                                                           
# 5
21289692       SHELIX+acyc                                                  160    Anopheles gambiae str. PEST           ENSANGP00000013833 [Anopheles gambiae str. PEST]                                                                                                                                                                                                            
34859646       HNOBA+SHELIX+acyc                                            329    Rattus norvegicus                     soluble guanylyl cyclase alpha2 subunit [Rattus norvegicus]                                                                                                                                                                                                 
72053335       TM+HNOBA+SHELIX+acyc                                         289    Strongylocentrotus purpuratus         PREDICTED: similar to CG1912-PA [Strongylocentrotus purpuratus]                                                                                                                                                                                             
72074053       TM+SHELIX+acyc                                               265    Strongylocentrotus purpuratus         PREDICTED: similar to CG5719-PA, partial [Strongylocentrotus purpuratus]                                                                                                                                                                                    
72131266       HNOBA+SHELIX+acyc                                            405    Strongylocentrotus purpuratus         PREDICTED: similar to CG4154-PC, isoform C [Strongylocentrotus purpuratus]                                                                                                                                                                                  
72148968       HNOBA+SHELIX+acyc                                            155    Strongylocentrotus purpuratus         PREDICTED: similar to Guanylate cyclase soluble, beta-2 chain (GCS-beta-2), partial [Strongylocentrotus purpuratus]                                                                                                                                         
51783984       HNOBA+SHELIX+acyc                                            247    Mus musculus                          PREDICTED: similar to Guanylate cyclase soluble, beta-1 chain (GCS-beta-1) (Soluble guanylate cyclase small subunit) [Mus musculus]                                                                                                                         
47215559       HNOBA+SHELIX+acyc                                            103    Tetraodon nigroviridis                unnamed protein product [Tetraodon nigroviridis]  
# 5
21297031       TM+TM+SHELIX+acyc                                            461    Anopheles gambiae str. PEST           ENSANGP00000011644 [Anopheles gambiae str. PEST]                                                                                                                                                                                                            
21297032       TM+SHELIX+acyc                                               546    Anopheles gambiae str. PEST           ENSANGP00000003987 [Anopheles gambiae str. PEST]                                                                                                                                                                                                            
24654868       TM+SHELIX+acyc                                               513    Drosophila melanogaster               CG5719-PA [Drosophila melanogaster]                                                                                                                                                                                                                         
72062946       TM+SHELIX+acyc                                               650    Strongylocentrotus purpuratus         PREDICTED: similar to CG5719-PA [Strongylocentrotus purpuratus]                                                                                                                                                                                             
72076183       TM+TM+SHELIX+acyc                                            688    Strongylocentrotus purpuratus         PREDICTED: similar to CG10738-PB, isoform B [Strongylocentrotus purpuratus]                                                                                                                                                                                 
51468848       SHELIX+acyc                                                  147    Homo sapiens                          PREDICTED: similar to Olfactory guanylyl cyclase GC-D precursor (Guanylate cyclase, olfactory) [Homo sapiens]                                                                                                                                               
72164868       SHELIX+acyc                                                  476    Strongylocentrotus purpuratus         PREDICTED: similar to CG10738-PB, isoform B [Strongylocentrotus purpuratus]                                                                                                                                                                                 
72008904       TM+SHELIX+acyc                                               591    Strongylocentrotus purpuratus         PREDICTED: similar to CG5719-PA [Strongylocentrotus purpuratus]                                                                                                                                                                                             
48099072       TM+TM+SHELIX+acyc                                            1259   Apis mellifera                        similar to CG5719-PA [Apis mellifera]                                                                                                                                                                                                                       
# 4
6322044        TM+TM+HAMP+SHELIX+HISKIN+HISKIN+REC                          1220   Saccharomyces cerevisiae              Histidine kinase osmosensor that regulates a MAP kinase cascade; transmembrane protein with an intracellular kinase domain that signals to Ypd1p and Ssk1p, thereby forming a phosphorelay system similar to bacterial two-component regulators; Sln1p [Saccharomyces cerevisia
45198802       TM+TM+HAMP+SHELIX+HISKIN+REC                                 1103   Eremothecium gossypii                 AFR284Wp [Eremothecium gossypii]                                                                                                                                                                                                                            
50289301       TM+HAMP+SHELIX+HISKIN+HISKIN+REC                             1169   Candida glabrata CBS138               unnamed protein product [Candida glabrata]                                                                                                                                                                                                                  
50302185       TM+TM+HAMP+SHELIX+HISKIN+HISKIN+REC                          1155   Kluyveromyces lactis NRRL Y-1140      unnamed protein product [Kluyveromyces lactis]                                                                                                                                                                                                              
50555538       TM+TM+TM+HISKIN+HAMP+SHELIX+HISKIN+HNOBA+HISKIN+REC          1229   Yarrowia lipolytica CLIB122           hypothetical protein [Yarrowia lipolytica]                                                                                                                                                                                                                  
50549233       TM+TM+HAMP+SHELIX+HISKIN+REC                                 1129   Yarrowia lipolytica CLIB122           hypothetical protein [Yarrowia lipolytica]                                                                                                                                                                                                                  
21622508       TM+HAMP+SHELIX+HISKIN+REC                                    1266   Neurospora crassa                     related to protein histidine kinase [Neurospora crassa]                                                                                                                                                                                                     
42547515       TM+TM+HAMP+SHELIX+HISKIN+REC                                 1148   Gibberella zeae PH-1                  hypothetical protein FG10042.1 [Gibberella zeae PH-1]                                                                                                                                                                                                       


# 76
30178957                          TMFQMLEKYSNNLEELIRERTEL-LDIERKKTEQLLNRMLP       STYkin+SHELIX+acyc                                           875    Anopheles gambiae str. PEST           ENSANGP00000013324 [Anopheles gambiae str. PEST]                                                                                                                                                                               
21298041                          DLLRRMEQYANNLESLVEEKTEQ-LSMEKRRTEELLYQVLP       STYkin+SHELIX+acyc                                           1106   Anopheles gambiae str. PEST           ENSANGP00000014144 [Anopheles gambiae str. PEST]                                                                                                                                                                               
21287856                          NMMAMMEKYANNLEQLVDERTDQ-LQEEKKKTEALLLEMLP       STYkin+SHELIX+acyc                                           1127   Anopheles gambiae str. PEST           ENSANGP00000009149 [Anopheles gambiae str. PEST]                                                                                                                                                                               
21287855                          QMMEMMEKYANNLEEIVQDRTRL-LCEEKRKTEDLLHRMLP       TM+STYkin+SHELIX+acyc                                        1154   Anopheles gambiae str. PEST           ENSANGP00000009147 [Anopheles gambiae str. PEST]                                                                                                                                                                               
30179077                          NLLQRMEQYANNLEALVDERTQD-YFEEKRKCEELLYQLLP       STYkin+SHELIX+acyc                                           1194   Anopheles gambiae str. PEST           ENSANGP00000012934 [Anopheles gambiae str. PEST]                                                                                                                                                                               
30174226                          NMLAIMEKYAYNLEGIVQERTNQ-LSEEKKKTESLLLRMLP       TM+TM+STYkin+SHELIX+acyc                                     993    Anopheles gambiae str. PEST           ENSANGP00000006391 [Anopheles gambiae str. PEST]                                                                                                                                                                               
48124391                          DLLRRMEQYANNLEALVEEKTEQ-LSLEK------------       TM+TM+TM+STYkin+SHELIX+acyc                                  969    Apis mellifera                        similar to CG3216-PB [Apis mellifera]                                                                                                                                                                                          
48112650                          QMMDMMEKYANNLEDLVSERTRL-LFEEKQKTEDLLHRMLP       STYkin+SHELIX+acyc                                           1420   Apis mellifera                        similar to ENSANGP00000009147 [Apis mellifera]                                                                                                                                                                                 
48098407                          NLLSRMEQYATNLETLVEERTAD-YLEEKRKCEELLYQLLP       STYkin+SHELIX+acyc                                           1292   Apis mellifera                        similar to CG31183-PA [Apis mellifera]                                                                                                                                                                                         
32563954                          SMMRMMEEYANNLEKLVGERTKL-AEEANLRAERLLFQLLP       TM+TM+STYkin+SHELIX+acyc                                     1034   Caenorhabditis elegans                C04H5.3 [Caenorhabditis elegans]                                                                                                                                                                                               
17534649                          HVFNMLEEYTSTLEEEIEERTKE-LTLEKKKADILLSRMLP       TM+STYkin+SHELIX+acyc                                        1137   Caenorhabditis elegans                Guanylyl CYclase family member (gcy-1) [Caenorhabditis elegans]                                                                                                                                                                
17534651                          HVFNMLEEYTSTLEEEIEERTKE-LTLEKKKADILLSRMLP       TM+STYkin+SHELIX+acyc                                        1118   Caenorhabditis elegans                Guanylyl CYclase family member (gcy-2) [Caenorhabditis elegans]                                                                                                                                                                
17534653                          HVFNMLEEYTSTLEVEVEERTKE-LTLEKKKADLLLSRMLP       TM+STYkin+SHELIX+acyc                                        1140   Caenorhabditis elegans                Guanylyl CYclase family member (gcy-3) [Caenorhabditis elegans]                                                                                                                                                                
17534657                          HVFNMLEEYTSTLEVDIEERTKE-LTLEKKKADILLSRMLP       TM+TM+STYkin+SHELIX+acyc                                     1122   Caenorhabditis elegans                Guanylyl CYclase family member (gcy-5) [Caenorhabditis elegans]                                                                                                                                                                
32566183                          QMIRMSEKYADELEQMVAIRTAD-LADAQMQTMRLLNEMLP       TM+STYkin+SHELIX+acyc                                        1016   Caenorhabditis elegans                ODoRant response abnormal ODR-1, guanylyl cyclase (115.7 kD) (odr-1) [Caenorhabditis elegans]                                                                                                                                  
17534661                          NMVSMIEKYTDKLEKDIAERNEE-LEAEKAKSEALLKMMLP       TM+STYkin+SHELIX+acyc                                        1000   Caenorhabditis elegans                Guanylyl CYclase family member (gcy-15) [Caenorhabditis elegans]                                                                                                                                                               
17534665                          NMVSMIEKYTDKLEKDIAERNEE-LEGEKAKSEALLKMMLP       TM+STYkin+SHELIX+acyc                                        1119   Caenorhabditis elegans                Guanylyl CYclase family member (gcy-21) [Caenorhabditis elegans]                                                                                                                                                               
17540894                          QMTRMMEQYANNLEKLVAERTGM-LEEANQRADRLLSQLLP       TM+STYkin+SHELIX+acyc                                        1206   Caenorhabditis elegans                Guanylyl CYclase family member (gcy-8) [Caenorhabditis elegans]                                                                                                                                                                
17540896                          QMMKMMEQYANNLEKLVAERTGM-LEEANIRADQLLTQLLP       TM+STYkin+SHELIX+acyc                                        1112   Caenorhabditis elegans                Guanylyl CYclase family member (gcy-18) [Caenorhabditis elegans]                                                                                                                                                               
17540898                          QMMRMMEQYANNLEKLVAERTGM-LEEANVRADKLLGQLLP       TM+STYkin+SHELIX+acyc+TM                                     1139   Caenorhabditis elegans                guanylyl cyclase (gcy-23) [Caenorhabditis elegans]                                                                                                                                                                             
17540900                          QMIEMIDEYSANLEQIVAERTRE-LEQDMSVTENLLYQLLP       TM+TM+STYkin+SHELIX+acyc                                     1091   Caenorhabditis elegans                Guanylyl CYclase family member (gcy-25) [Caenorhabditis elegans]                                                                                                                                                               
17534659                          HMVLMMEKYQTQLEDLVDERTIE-LKDEQRRSQHLLQRMLP       TM+STYkin+SHELIX+acyc                                        1280   Caenorhabditis elegans                Guanylyl CYclase family member (gcy-12) [Caenorhabditis elegans]                                                                                                                                                               
17559160                          LMIKNLTAYTQGLNETVKNRTAE-LEKEQEKGDQLLMELLP       TM+TM+STYkin+SHELIX+acyc                                     1093   Caenorhabditis elegans                abnormal DAuer Formation family member (daf-11) [Caenorhabditis elegans]                                                                                                                                                       
17561794                          ----VLESYASTLEDEVAERMKE-LVEEKKKSDVLLYRMLP       TM+TM+STYkin+SHELIX+acyc                                     1161   Caenorhabditis elegans                Guanylyl CYclase family member (gcy-6) [Caenorhabditis elegans]                                                                                                                                                                
17561796                          HVFNMLESYASSLEEEVSERTKE-LVEEKKKSDVLLYRMLP       TM+TM+STYkin+SHELIX+acyc                                     1130   Caenorhabditis elegans                guanylyl cyclase (gcy-7) [Caenorhabditis elegans]                                                                                                                                                                              
17561798                          HVFSVLEKHASSLEDEVQERMKE-LVEEKKKSDILLYRMLP       TM+STYkin+SHELIX+acyc                                        1025   Caenorhabditis elegans                Guanylyl CYclase family member (gcy-13) [Caenorhabditis elegans]                                                                                                                                                               
17561802                          HVFNMLETYASTLEEEVSDRTKE-LTEEKKKSDVLLYRMLP       TM+TM+STYkin+SHELIX+acyc                                     1108   Caenorhabditis elegans                Guanylyl CYclase family member (gcy-20) [Caenorhabditis elegans]                                                                                                                                                               
17561804                          HVFNVLEQYASNLEDEVQARMKE-LTEEKKRSDVLLYRMLP       TM+TM+TM+STYkin+SHELIX+acyc                                  1012   Caenorhabditis elegans                Guanylyl CYclase family member (gcy-22) [Caenorhabditis elegans]                                                                                                                                                               
17568383                          QMMKMMEEYTANLENMVRDRTAL-LEEAQKQADRLLNSMLP       TM+TM+STYkin+SHELIX+acyc                                     1118   Caenorhabditis elegans                Guanylyl CYclase family member (gcy-9) [Caenorhabditis elegans]                                                                                                                                                                
17568387                          NIMNLLDRYRNNLEDVIKERTEQ-LEDERKRNESLLLQLLP       STYkin+SHELIX+acyc                                           1057   Caenorhabditis elegans                Guanylyl CYclase family member (gcy-11) [Caenorhabditis elegans]                                                                                                                                                               
32563952                          HVFNILEDYTTNLEVEVEDRTKE-LTAEKKKADVLLGRMLP       TM+TM+TM+TM+TM+STYkin+SHELIX+acyc                            1246   Caenorhabditis elegans                guanylyl cyclase (gcy-19) [Caenorhabditis elegans]                                                                                                                                                                             
32563666                          HVFNMLETYASTLEEEVNERTKE-LVEEQKKSDVLLYRMLP       TM+STYkin+SHELIX+acyc                                        1146   Caenorhabditis elegans                Guanylyl CYclase family member (gcy-17) [Caenorhabditis elegans]                                                                                                                                                               
32562827                          NLLKRMEQYANNLEGLVEERTQE-YLAEKKKVEELLHQLLP       TM+STYkin+STYkin+SHELIX+acyc                                 1276   Caenorhabditis elegans                guanylyl cyclase (144.9 kD) (1F75) [Caenorhabditis elegans]                                                                                                                                                                    
17561800                          HVFNMLETYASTLEEEVSDRTKE-LVEEKKKSDVLLYRMLP       TM+TM+STYkin+SHELIX+acyc                                     1170   Caenorhabditis elegans                Guanylyl CYclase family member (gcy-14) [Caenorhabditis elegans]                                                                                                                                                               
17540902                          QMIRMNEKYADELETLVAARSAD-LALAQMQTMRLLNEMLP       STYkin+SHELIX+acyc                                           613    Caenorhabditis elegans                Guanylyl CYclase family member (gcy-27) [Caenorhabditis elegans]                                                                                                                                                               
24647268                          NLLKRMELYANNLEELVEERTQD-YHEEKKKCEKLLYQLLP       TM+STYkin+SHELIX+acyc                                        1417   Drosophila melanogaster               CG31183-PA [Drosophila melanogaster]                                                                                                                                                                                           
24656571                          DLLNRMEQYANNLESLVEEKTRQ-LSLEKQRTEELLYQILP       TM+STYkin+SHELIX+acyc                                        1141   Drosophila melanogaster               CG3216-PB, isoform B [Drosophila melanogaster]                                                                                                                                                                                 
24663839                          NMMAMMEKYANNLEALVDDRTDQ-LQEEKKKTDALLHEMLP       STYkin+SHELIX+acyc                                           1272   Drosophila melanogaster               CG10738-PB, isoform B [Drosophila melanogaster]                                                                                                                                                                                
24663843                          NMMAMMEKYANNLEALVDDRTDQ-LQEEKKKTDALLHEMLP       STYkin+SHELIX+acyc                                           1172   Drosophila melanogaster               CG10738-PA, isoform A [Drosophila melanogaster]                                                                                                                                                                                
24666940                          QMMEMMEKYANNLEDIVTERTRL-LCEEKMKTEDLLHRMLP       STYkin+SHELIX+acyc                                           1525   Drosophila melanogaster               CG8742-PA, isoform A [Drosophila melanogaster]                                                                                                                                                                                 
28574512                          NMLSIMEKYAYNLEGLVQERTNL-LYEEKKKTDMLLYQMLP       STYkin+SHELIX+acyc                                           1163   Drosophila melanogaster               CG33114-PA [Drosophila melanogaster]                                                                                                                                                                                           
24656567                          DLLNRMEQYANNLESLVEEKTRQ-LSLEKQRTEELLYQILP       STYkin+SHELIX+acyc                                           1076   Drosophila melanogaster               CG3216-PA, isoform A [Drosophila melanogaster]                                                                                                                                                                                 
24644018                          TMFQMLEKYSNNLEELIRERTEQ-LDIERKKTEQLLNRMLP       STYkin+SHELIX+acyc                                           975    Drosophila melanogaster               CG9783-PA [Drosophila melanogaster]                                                                                                                                                                                            
51467872                          SVMSKLEVYANYLEEVVQERTSQ-LTAEKRKVEKLLSTKVP       TM+STYkin+SHELIX+TM+acyc+TM                                  1090   Homo sapiens                          PREDICTED
4504219                           SMLRMLEQYSSNLEDLIRERTEE-LEIEKQKTEKLLTQMLP       TM+TM+STYkin+SHELIX+acyc                                     1108   Homo sapiens                          guanylate cyclase 2F [Homo sapiens]                                                                                                                                                                                            
4505437                           NLLLRMEQYANNLEKLVEERTQA-YLEEKRKAEALLYQILP       STYkin+SHELIX+acyc                                           995    Homo sapiens                          natriuretic peptide receptor B isoform a precursor [Homo sapiens]                                                                                                                                                              
4580422                           NLLLRMEQYANNLEKLVEERTQA-YLEEKRKAEALLYQILP       STYkin+SHELIX+acyc                                           1047   Homo sapiens                          natriuretic peptide receptor B isoform b precursor [Homo sapiens]                                                                                                                                                              
4826752                           TLIRRLQLYSRNLEHLVEERTQL-YKAERDRADRLNFMLLP       TM+TM+STYkin+SHELIX+acyc                                     1073   Homo sapiens                          guanylate cyclase 2C (heat stable enterotoxin receptor) [Homo sapiens]                                                                                                                                                         
4504217                           SMLRMLEQYSSNLEDLIRERTEE-LELEKQKTDRLLTQMLP       TM+STYkin+SHELIX+acyc                                        1103   Homo sapiens                          guanylate cyclase 2D, membrane (retina-specific) [Homo sapiens]                                                                                                                                                                
40254426                          NLLSRMEQYANNLEELVEERTQA-YLEEKRKAEALLYQILP       STYkin+STYkin+SHELIX+acyc                                    1061   Homo sapiens                          natriuretic peptide receptor A/guanylate cyclase A (atrionatriuretic peptide receptor A) [Homo sapiens]                                                                                                                        
38085380                          TLIRRLQLYSRNLEHLVEERTQL-YKAERDRADHLNFMLLP       TM+STYkin+SHELIX+acyc                                        1072   Mus musculus                          PREDICTED
6680135                           SMLRMLEQYSSNLEDLIRERTEE-LEQEKQKTDRLLTQMLP       TM+TM+STYkin+SHELIX+acyc                                     1108   Mus musculus                          guanylate cyclase 2e [Mus musculus]                                                                                                                                                                                            
28916675                          NLLLRMEQYANNLEKLVEERTQA-YLEEKRKAEALLYQILP       STYkin+SHELIX+acyc                                           1047   Mus musculus                          natriuretic peptide receptor 2 [Mus musculus]                                                                                                                                                                                  
51772455                          SMLRMLEQYSSNLEDLIRERTEE-LEIEKQKTEKLLTQMLP       TM+TM+STYkin+SHELIX+acyc                                     1152   Mus musculus                          similar to guanylate cyclase 2F, retinal [Mus musculus]                                                                                                                                                                        
51771626                          SMMGKLETYANHLEEVVEERTRE-LVAEKRKVEKLLSTMLP       STYkin+SHELIX+acyc                                           1100   Mus musculus                          PREDICTED
51761722                          SMLRMLEKYSESLEDLVQERTEE-LELERRKTERLLSQMLP       TM+TM+STYkin+SHELIX+acyc                                     1421   Mus musculus                          similar to Olfactory guanylyl cyclase GC-D precursor (Guanylate cyclase, olfactory) [Mus musculus]                                                                                                                             
34328514                          NLLSRMEQYANNLEELVEERTQP-YLEEKRKAEALLYQILP       STYkin+SHELIX+acyc                                           1057   Mus musculus                          natriuretic peptide receptor 1 [Mus musculus]                                                                                                                                                                                  
20514776                          SMMGKLEMYASHLEEVVEERTCQ-LVAEKRKVEKLLSTMLP       STYkin+SHELIX+acyc                                           1100   Rattus norvegicus                     guanylate cyclase 2g [Rattus norvegicus]                                                                                                                                                                                       
18543337                          SMLRMLEKYSQSLEGLVQERTEE-LELERRKTERLLSQMLP       TM+TM+STYkin+SHELIX+acyc                                     1110   Rattus norvegicus                     guanylate cyclase 2d [Rattus norvegicus]                                                                                                                                                                                       
16758694                          NLLLRMEQYANNLEKLVEERTQA-YLEEKRKAEALLYQILP       TM+TM+STYkin+SHELIX+acyc                                     1047   Rattus norvegicus                     natriuretic peptide receptor 2 [Rattus norvegicus]                                                                                                                                                                             
6981280                           NLLSRMEQYANNLEELVEERTQA-YLEEKRKAEALLYQILP       STYkin+SHELIX+acyc                                           1057   Rattus norvegicus                     natriuretic peptide receptor 1 [Rattus norvegicus]                                                                                                                                                                             
13242283                          SMLRMLEQYSSNLEDLIRERTEE-LEQEKQKTDRLLTQMLP       TM+TM+STYkin+SHELIX+acyc                                     1108   Rattus norvegicus                     guanylyl cyclase 2e [Rattus norvegicus]                                                                                                                                                                                        
16758684                          SMLRMLEQYSSNLEDLIRERTEE-LEIEKQKTEKLLTQMLP       TM+TM+STYkin+SHELIX+acyc                                     1108   Rattus norvegicus                     guanylate cyclase 2f [Rattus norvegicus]                                                                                                                                                                                       
47551057                          NMIAIMERYTNNLEELVDERTQE-LQKEKTKTEQLLHRMLP       TM+TM+STYkin+SHELIX+acyc                                     1125   Strongylocentrotus purpuratus         guanylate cyclase [Strongylocentrotus purpuratus]                                                                                                                                                                              
72010207                          NMVMIMEKYANNLEEIVEDRTQQ-LVEEKKKTENLLHRMLP       TM+STYkin+SHELIX+acyc                                        908    Strongylocentrotus purpuratus         PREDICTED
72008591                          -LLNRMEQYASNLEALVEERTAA-FLEEKKRSETLLYEVLP       STYkin+SHELIX+acyc+STYkin+SHELIX+acyc                     1042   Strongylocentrotus purpuratus         PREDICTED
72043227                          -LLNRMEQYATNLESLVEERTAA-FLEEKKRSETLLYEVLP       TM+STYkin+SHELIX+acyc                                        969    Strongylocentrotus purpuratus         PREDICTED
72014291                          NLLSRMEQYATNLETLVEERTAA-FLEEKKRSETLLYEVLP       STYkin+SHELIX+acyc                                           1178   Strongylocentrotus purpuratus         PREDICTED
72011791                          NMIVIMEKYANNLEDIVEDRTHQ-LIEEKKKTDNLLHQMLP       TM+TM+STYkin+SHELIX+acyc                                     1040   Strongylocentrotus purpuratus         PREDICTED
72004807                          LMMQLMEKYSRHLESIVAERTQD-LLLEKQKTDRLLYSMLP       STYkin+SHELIX+acyc                                           705    Strongylocentrotus purpuratus         PREDICTED
72006640                          NMLSKMEKYTENLEKVVADRTGQ-LLEEKKKTDALLFRMLP       STYkin+SHELIX+acyc                                           796    Strongylocentrotus purpuratus         PREDICTED
72004443                          NLLSRMEQYATNLEALVEERTAA-FLEEKKRSETLLYEVLP       TM+STYkin+SHELIX                                             806    Strongylocentrotus purpuratus         PREDICTED
72011180                          TLLSRMEQYASNLESLVQERTEA-FYEEKRKAEELLYQILP       TM+STYkin+SHELIX+acyc                                        656    Strongylocentrotus purpuratus         PREDICTED
[truncated: 32,440 more chars]
